# Identification of cytochrome P450 monooxygenase genes and their expression in response to high temperature in the alligatorweed flea beetle *Agasicles hygrophila* (Coleoptera: Chrysomelidae)

**DOI:** 10.1038/s41598-018-35993-1

**Published:** 2018-12-14

**Authors:** Hong Zhang, Meiting Zhao, Yiran Liu, Zhongshi Zhou, Jianying Guo

**Affiliations:** State Key Laboratory for Biology of Plant Diseases and Insect Pests, Institute of Plant Protection, Chinese Academy of Agricultural Sciences, Beijing, China

## Abstract

Cytochrome P450 monooxygenases (P450s) are a large class of enzymes that play essential roles in metabolic processes such as hormone synthesis and the catabolism of toxins and other chemicals in insects. In the present study, we identified 82 P450 genes using comprehensive RNA sequencing in the flea beetle *Agasicles hygrophila*, and all of the sequences were validated by cloning and sequencing. Phylogenetic analysis showed that the P450 genes in *A*. *hygrophila* fell into the mitochondrial clan, CYP2 clan, CYP3 clan and CYP4 clan and were classified into 20 families and 48 subfamilies. Most *A*. *hygrophila* P450 genes had high sequence homology with those from other coleopteran insects. To understand the effects of high temperatures on the metabolic processes of female and male adults, we studied the effects of two temperature regimes (constant temperature of 28 °C for 20 h with a 4-h period of high temperatures of 30 °C and 39 °C) on the expression levels of P450 genes in *A*. *hygrophila* using RT-PCR and qRT-PCR. The results showed that there were no differences in expression in 30 P450 genes between the control and high-temperature-treated *A*. *hygrophila* adults, while 22 P450 genes showed up-regulated expression and 19 P450 genes were down-regulated in *A*. *hygrophila* female adults after high-temperature treatment. For *A*. *hygrophila* male adults exposed to high temperatures, we found that 8 P450 genes had higher expression levels and 12 P450 genes had lower expression levels under the same conditions. The P450 genes are candidates that showed significantly different expression levels after high-temperature treatments in *A*. *hygrophila* adults, and further studies are needed to determine their possible roles in metabolic processes during the response to elevated temperatures.

## Introduction

Insects are an ancient group of organisms that evolved from one group of crustaceans over 450 million years ago during the Ordovician period. Extant insects are extremely diverse and are represented by nearly one million species, making them one of the most evolutionally successful group of metazoans on the earth^1,2^. The various insect species include both agricultural pests and beneficial insects^1^. The characteristics of insects that allow them to live in an environment exposed to harmful chemicals or life-threatening conditions have contributed to their evolutionary success^3^. To adapt to a challenging environment containing natural or synthetic toxins, insects have evolved diverse metabolic mechanisms to defend against toxins.

One of the multiple enzyme species involved in detoxification is cytochrome P450 monooxygenases (P450s)^4^. P450s are remarkable because they have multiple clades and various functions^5^. With an increasing number and diversity of insect transcriptomes and genomes being reported, the diversity of insect P450 genes can be better identified. At present, insect P450 genes group into four major phylogenetic clades: CYP2, CYP3, CYP4, and the mitochondrial P450 clade^1^.

It has been reported that P450s have various enzymatic activities that can catalyse more than 60 different chemical reactions, such as aromatic and aliphatic hydroxylations, epoxidations, dealkylations, etc^5^. Insect P450 enzymes may function in the biosynthesis and catabolism of important endogenous compounds (such as juvenile hormone) and also in the degradation of harmful xenobiotics^2^. P450 enzymes (CYP15A1) have been shown to convert methyl farnesoate to juvenile hormone by epoxidation in cockroaches, and to be involved in the control of many fundamental physiological processes involving juvenile hormone in insects such as development, growth, metamorphosis, and reproduction^6,7^. In adapting to the harmful chemical stresses that they encounter, insects have proven their ability to detoxify xenobiotics. For example, some P450 genes (*CYP6BQ8*, *CYP6BQ9*, *CYP6BQ10*, and *CYP6BQ11*) are involved in the degradation of deltamethrin in *Tribolium castaneum*^8,9^.

Alligator weed [*Alternanthera philoxeroides* (Mart.) Griseb., Amaranthaceae] is a globally invasive plant that originated in South America and is usually found in wetlands, but can also occupy terrestrial habitats^10^. *A*. *philoxeroides* is an invasive weed in many countries around the world, such as the USA, Australia, India, and China^10^. *A*. *philoxeroides* was purposefully introduced for livestock fodder crop in China in the 1930s^11,12^ and now occurs widely throughout Hunan, Hubei, Fujian, Hainan, and Yunnan provinces in China^13^. *Alternanthera philoxeroides*, as a pernicious invasive plant, has diverse social, economic, and environmental impacts^14^. In an effort to control the damage caused by *A*. *philoxeroides*, *Agasicles hygrophila* (Selman & Vogt) (Coleoptera: Chrysomelidae), a flea beetle native to Florida in the US, was introduced for biological control of alligatorweed in China in 1987^15^. *Agasicles hygrophila* is effective at controlling *A*. *philoxeroides*, and the beetle has been introduced into many countries to control the spread of this invasive weed^16^. However, field surveys showed that the population density of *A*. *hygrophila* decreases sharply during July and August in Hunan province, China; therefore, it could not control the alligator weed efficiently, which resulted in agricultural product loss and ecological damage during this time^17,18^.

In Hunan province, the average daily temperatures were above 33 °C in July and August, and the frequency of high ambient temperatures exceeding 39 °C was 42.5% in July and 32.1% in August, respectively^17^. This suggests that high temperature may be the main factor that caused the fecundity and the population density of *A*. *hygrophila* to decline in July and August. Elevated temperature conditions can have profound effects on insect survival, development and reproduction^19,20^. Many studies investigating the influence of temperature on the survival of insects have shown that high temperatures have a negative effect on survival and may lead to rapid death^21^. In addition, previous studies have confirmed that high temperatures significantly inhibit tissue development in insects. For example, high temperatures have an impact on ovarian development and oogenesis in *Drosophila*^22^, which directly decreases their fecundity and population density^22,23^. The reason for the above results is that the reproductive physiology of insects needs a constant energy supply, while high temperatures influence the energy transfer process in insects.

High temperatures have also been shown to influence the physiological metabolic processes in insects. For example, juvenile hormones (JHs) are important hormones in insects that control insect metamorphosis, development and many other physiological functions^7^. Research has shown that the rate of synthesis of JH by the corpus allatum in honey bees is high at 35 °C but is extremely low at 25 °C^24^. High temperatures can not only influence JH synthesis, but also affect its degradation, which then influences physiological functions in insects^25^. In summary, the energy and substance metabolism processes are influenced by environmental temperatures. Many kinds of enzymes are involved in the metabolic process in insects, and P450 monooxygenases are remarkable because “there are so many of them and they do so many things”^5^. Currently, most studies on P450 monooxygenases have focused on their functions during the degradation of insecticides, but their expression levels and functions in responding to high temperatures are seldom reported, which prevents us from clarifying the molecular regulation of the mechanisms of P450 in insects. In addition to the well-studied ability of P450 enzymes to degrade insecticides, previous analyses of CYP450 function have indicated that the Halloween family of cytochrome P450s are involved in the synthesis of 20-hydroxyecdysone (20E), which is is a major modulator of the developmental processes that lead to molting and metamorphosis^26^. Moreover, CYP15A1 has been shown to catalyze the epoxidation of methyl farnesoate to juvenile hormone^6^. As an important insect hormone, juvenile hormone can regulate development, metamorphosis, reproduction, and many other physiological functions in insects^6^. It is well known that ecdysone and juvenile hormone act together to regulate the reproductive process in insects, and a decrease in reproductive ability is one of the important reasons for population fluctuation. Our previous studies have shown that high temperatures have a significant impact on the fecundity and population size of *A*. *hygrophila* adults^17^. Our purpose in this study is to explore the molecular mechanism by which high temperature affects population fluctuation. The ecological phenomena we have observed indicate that high temperatures have a profound influence on larval pupation and adult eclosion, and most of the larve and pupa were found to be abnormal after high temperature treatment (unpublished data). Furthermore, we found that the development of *A*. *hygrophila* ovaries were abnormal, and fecundities were sharply decreased after exposure to a temperature of 39 °C^17^. Because of the observed ecological effect of high temperature on *A*. *hygrophila* and the various reported functions of CYP, we suspect that the CYP gene family is one of the reasons to explain the decline in fecundity and population size in *A*. *hygrophila* at high temperatures.

Transcriptome sequencing technology provides a rapid and cost-effective means to produce a large amount of sequencing data for genomics research, and is widely used to (1) quantify gene expression in different tissues and developmental stages, (2) investigate phylogenetic relationships, (3) analyze metabolic pathways, and (4) to discover novel genes and describe their functions^27^. The use of next-generation sequencing (NGS) technology is now routine because it can provide useful genetic information and facilitate the exploration of gene functions, cellular responses, alternative gene splicing, and gene fusion in nonmodel organisms^27^. For *Agasicles hygrophila*, which is a non-model insect, a genomic sequence dataset has not been reported, making transcriptome data especially valuable for further research on this species^28^.

We used Illumina DNA sequencing to produce a transcriptome dataset for *A*. *hygrophila*. In our study, we successfully identified a total of 82 *A*. *hygrophila* CYP genes from the *A*. *hygrophila* transcriptome library using sequences from GenBank as queries in homology searches. It has been reported that insect genomes normally contain from 10 to >100 P450 genes (http://drnelson.uthsc.edu/CytochromeP450.html)^2^. At present, genome and transcriptome annotations have identified 143 CYP genes in *Tribolium castaneum*^2^, 74 CYP genes in *Leptinotarsa decemlineata*^29^, 90 CYP genes in *Drosophila melanogaster*^30^, 36 CYP genes in *Cnaphalocrocis medinalis*^3^, 48 CYP genes in *Apis mellifera*^31^, 84 CYP genes in *Bombyx mori*^32^, and 111 CYP genes in *Anopheles gambiae*^33^. However, no CYP gene sequences from *A*. *hygrophila* have been submitted to GenBank at present, which hampered our exploration into the functions of P450 genes in *A*. *hygrophila*. The 82 CYP genes that we identified in *A*. *hygrophila* grouped into the CYP2, CYP3, CYP4, and mitochondrial clans. We performed phylogenetic analyses with all *A*. *hygrophila* CYP proteins against those from several other species of insects, and we also determined the expression profiles of female and male *A*. *hygrophila* adults exposed to high temperature. The results presented here will enable a more detailed understanding of the evolution of CYPs in insects, and will facilitate further research to investigate the functions of P450 genes in *A*. *hygrophila*.

## Results

### Analysis of the *A*. *hygrophila* transcriptome

We constructed two transcriptome libraries from female and male adult flea beetles from the control treatment and sequenced them using an Illumina HiSeq. 2500 instrument. There were 88,042,749 and 97,496,934 raw reads from 150 to 200 bp in length generated from the female and male adult libraries, respectively. The raw reads were trimmed to remove adaptor sequences, and after discarding low-quality reads and contaminating sequences, 87,345,512 (99.2%) and 96,754,220 (99.2) clean reads were retained in the female and male adult libraries, respectively. All of the clean reads were then combined and assembled, which gave 101,557 transcripts with a mean length of 1,297 bp. Subsequent merging and clustering produced 69,328 unigenes in which the mean length was 833 bp and the N50 wasf 1853 bp (Table 1). Of these, 13,012 unigenes had lengths > 1,000 bp, representing 18.77% of all the unigenes (Table 2).

**Table 1:** Summary of the *Agasicles hygrophila* adult transcriptome assembly.

Parameter	Transcripts	Unigenes
Minimum length	201 bp	201 bp
Mean length	1297 bp	833 bp
Median length	488 bp	354 bp
Max length	56520 bp	56520 bp
N50	3102 bp	1853 bp
N90	433 bp	283 bp
Total nucleotides	131695627	57724095
total number	101557	69328

**Table 2:** Length distribution of the assembled transcripts and unigenes from the *Agasicles hygrophila* adult transcriptome.

Length distribution (bp)	Transcripts		Unigenes	
	Number	Percentage	Number	Percentage
200–300	30446	29.98%	27663	39.90%
300–500	21106	20.78%	17421	25.13%
500–1000	17096	16.83%	11232	16.20%
1000–2000	13588	13.38%	6324	9.12%
2000+	19319	19.02%	6688	9.65%

**Figure 1:**
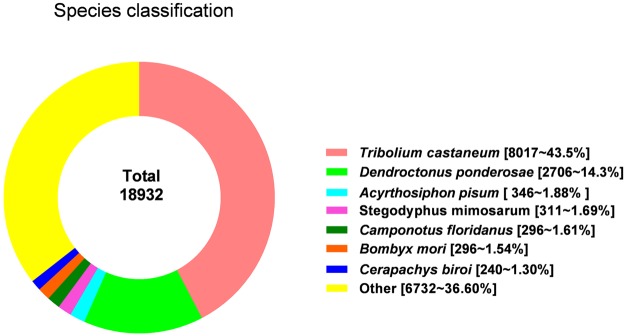
Species distribution of the unigenes based on best-hit annotation terms in the nr database.

The 69,328 *A*. *hygrophila* adult unigenes were used as queries in BLASTx and BLASTn searches against other insect species using an E-value < 1.0E-5^34^. There were significant BLASTx hits in the non-redundant (nr) protein database for 18,932 of the 69,328 unigenes (27.3%), and there were also significant BLASTn hits in the non-redundant nucleotide sequence database for 5,135 of the 69,328 unigenes (7.4%). The most hits for the annotated unigenes were with Coleoptera insect genes; this included 8,107 unigenes homologous to *Tribolium castaneum* sequences and 2,706 unigenes that were homologous to *Dendroctonus ponderosae*. The species distribution for all of the best-hit annotated genes is shown in Fig. 1.

**Figure 2:**
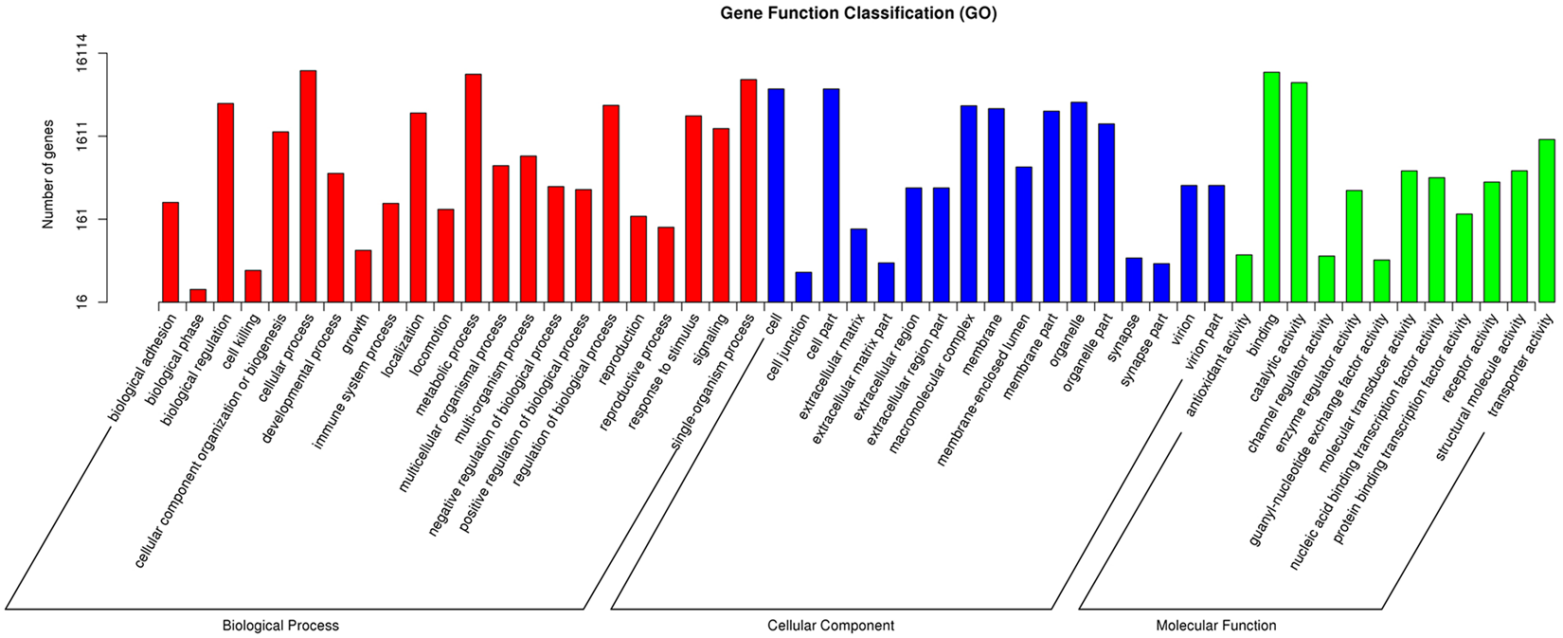
Gene ontology (GO) classifications of the *Agasicles hygrophila* unigenes.

We used GO (Gene Ontology) assignments in an effort to functionally classify the predicted *A*. *hygrophila* proteins. After analysis, we found that 16,114 of the 18,932 unigenes (85.12%) could be annotated in the GO database and classified into the three functional GO categories: “molecular function”, “biological process”, and “cellular component” (Fig. 2). In the GO classification system, the three main functional categories are divided into 57 detailed sub-categories. For the “biological process”, the unigenes expressed in *A*. *hygrophila* adults were mainly linked to the “cellular process” (9,928; 61.6%) and “metabolic process” (8,991; 55.8%) sub-categories. In the “cellular component” term, “cell” (5975; 32.5%) and “cell part” (5974; 32.5%) were found to be the most abundant categories. In “molecular function”, the transcripts were primarily distributed among “binding” (9,500; 58.9%) and “catalytic activity” (7,120; 44.2%).

### Identification of candidate *A*. *hygrophila* CYP genes

We identified 82 candidate *CYP* genes with amino acid sequences based on the *A*. *hygrophila* transcriptome data; of these, 30 sequences had full-length ORFs with lengths of approximately 1,500 nucleotides (Table S5). We verified all 82 sequences by PCR amplification of the cDNAs and Sanger sequencing. The individual names for the *A*. *hygrophila* P450 genes were assigned by the Cytochrome P450 Nomenclature Committee (D Nelson, University of Tennessee, Memphis, TN, USA)^3^. All of the *CYP* genes were first identified in *A*. *hygrophila* and were then submitted to GenBank under accession numbers. MH158318–MH158399. In summarizing our results, we found that the P450 genes in *A*. *hygrophila* grouped into four clans; the CYP2, CYP3, CYP4, and mitochondrial clans, which were further classified into 20 families and 48 subfamilies (Table 3).

**Table 3:** Number of CYP families, subfamilies, and genes in each of the four P450 clans in *Agasicles hygrophila* and other insect species.

Insect Species	CYP2 clan			Mitochondrial clan			CYP3 clan			CYP4 clan			Total
	Family	Subfamily	All genes	Family	Subfamily	All genes	Family	Subfamily	All genes	Family	Subfamily	All genes	
*Agasicles hygrophila*	6 (CYP15, 18, 303,305, 306, 307)	6	7	6 (CYP12, 49, 301, 302, 314, 315)	6	6	4 (CYP6, 9, 345, 347)	21	39	4 (4, 411, 412, 349)	15	30	82
*Tribolium castaneum*	7 (CYP15, 18, 303–307)	8	8	8 (CYP12, 49, 301,302, 314, 315,334, 353)	9	9	6 (CYP6, 9, 345–348)	27	79	5 (CYP4, 349–352)	15	47	143
*Leptinotarsa decemlineata*	4 (CYP18, 305, 306, 307)	4	4	7(CYP12, 301, 302, 314, 315, 334, 353)	9	11	4 (CYP6, 9, 345, 347)	14	39	4 (CYP4, 350, 412, 413)	8	20	74
*Drosophila melanogaster*	6(CYP18,303–307)	6	7	6(CYP12,49,301,302,314, 315)	10	12	7(CYP6,9,28, 308–310, 317)	17	40	6 (CYP4, 311–313, 316, 318)	16	32	91
*Apis mellifera*	8 (CYP15, 18, 303,305, 306, 307, 342, 343)	8	8	5 (CYP301, 302, 314, 315,334)	6	6	3 (CYP6, 9, 336)	11	30	1 (CYP4)	4	4	48
*Culex quinquefasciatus*	7 (CYP15, 18, 303–307)	9	16	6 (CYP12, 49, 301, 302, 314, 315)	6	12	3 (CYP6, 9, 329)	23	89	3(CYP4, 325, 326)	29	82	204
*Bombyx mori*	8(CYP15,18, 303, 305–307)	7	7	7(CYP49,301, 302, 314, 315, 333, 339)	8	11	8(CYP6,9,324,332,337,338,354,365)	17	30	5 (CYP4, 340, 341, 366, 367)	16	36	84
*Aedes aegypti*	7(CYP15,18,303–307)	9	11	6(CYP12,49,301,302,314,315)	6	10	3 (CYP6, 9, 329)	23	84	2(CYP4,325)	25	59	164
*Cnaphalocrocis medinalis*	5(CYP18,304,305,306,307)	5	5	4(CYP301,314,333,339)	5	6	8(CYP6,9,321,324,337,338,345)	12	16	3(CYP4,341,367)	7	9	36
*Trialeurodes vaporariorum*	4(CYP18,304,305,306)	4	5	6 (CYP301, 302, 314, 315,353, 404)	7	9	4 (CYP6, 9, 401, 402)	17	49	3(CYP4, 380, 403)	9	15	78
*Acyrthosiphon pisum*	6 (CYP15, 18,303,305,306,307)	7	10	5(CYP301,302, 314, 315, 353)	6	8	1 (CYP6)	6	25	2 (CYP4, 380)	7	24	50
*Tetranychus urticae*	2 (CYP307, 392)	6	50	4 (CYP302, 314, 315, 381)	4	5	4 (CYP319, 383, 384, 385)	6	11	6 (CYP4, 386–390)	12	26	92
*Pogonomyrmex barbatus*	8(CYP15,18,303,305,306,307,343,369)	8	8	5 (12, 302, 314, 315, 334)	5	6	3 (CYP6, 9, 336)	11	61	1 (CYP4)	7	40	115
*Nasonia vitripennis*	6 (CYP15, 18, 303,305, 306, 307)	6	6	6(CYP12,301,302,314,315, 334)	7	7	3 (CYP6, 9, 336)	11	59	1(CYP4)	5	34	106
*Danaus plexippus*	7 (CYP15, 18, 303–307)	7	8	6(CYP49,301, 302, 314, 315, 333)	8	12	9 (CYP6, 9, 321, 324, 332, 337, 338, 354, 365)	14	36	7 (CYP4, 340, 341, 366, 367, 405, 421)	13	30	86
*Plutella xylostella*	6 (CYP15, 18, 303,304, 305, 307)	7	10	8(CYP49,301, 302, 314, 315, 333, 339, 428)	10	13	7 (CYP6, 9, 321,338,354, 365, 429)	16	26	5 (CYP4, 340, 341, 366, 367)	19	36	85

### Phylogenetic analysis of AhCYP sequences

**Figure 3:**
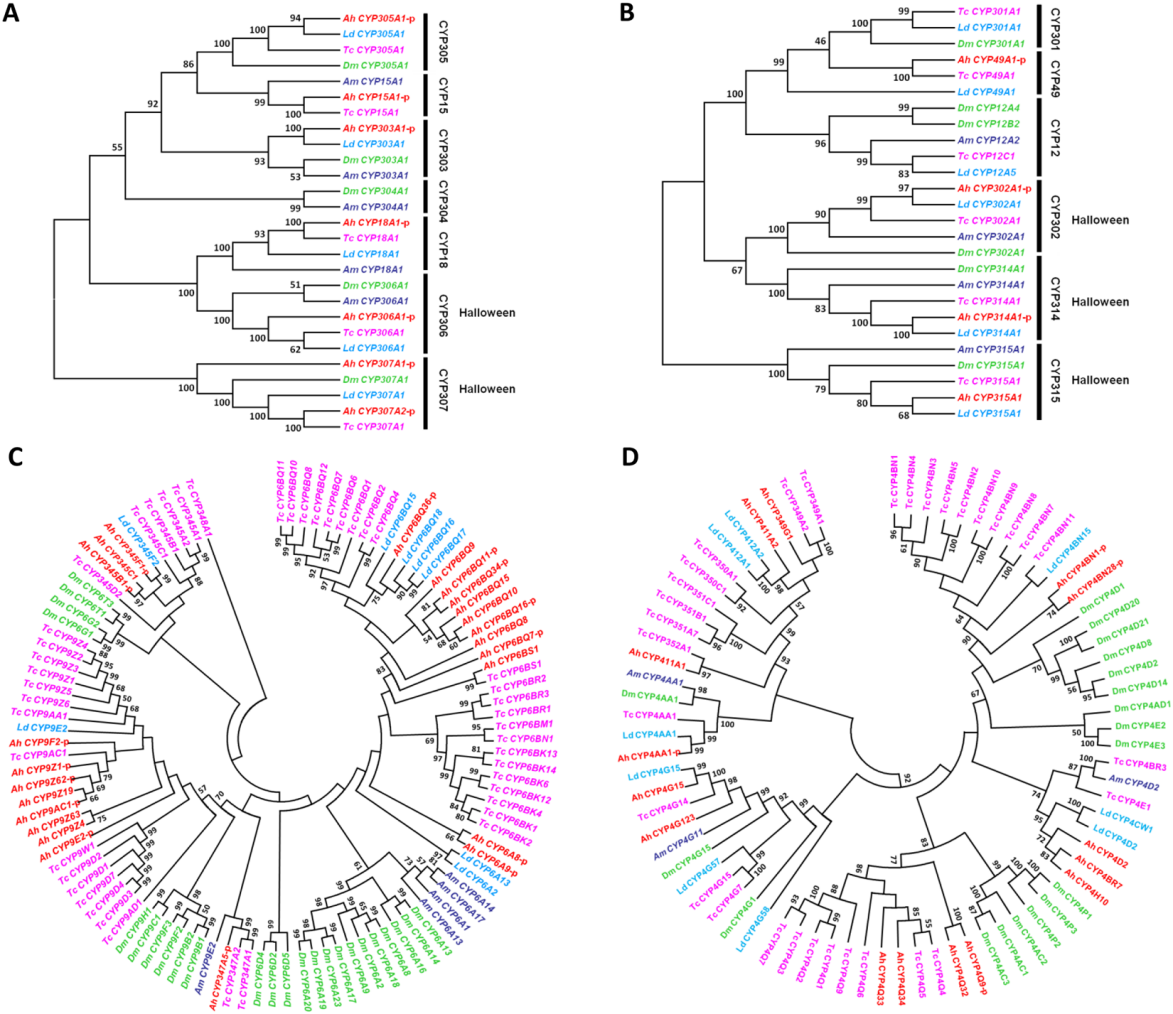
Unrooted neighbor-joining consensus trees of the four P450 clans. (**A**) CYP2 clan; (**B**) mitochondrial clan; (**C**) CYP3 clan; and (**D**) CYP4 clan. “AhCYPxx-p” represents “AhCYPxx-partial”. The phylogenetic trees were generated by MEGA 5 using amino acid sequences from *Agasicles hygrophila* (Ah), *Tribolium castaneum* (Tc), *Drosophila melanogaster* (Dm), *Leptinotarsa decemlineata* (Lc), and *Apis mellifera* (Am).

The evolutionary relationships of the *A*. *hygrophila* CYPs were visualized by constructing four phylogenetic trees using CYP sequences from *T*. *castaneum*, *D*. *melanogaster*, *L*. *decemlineata*, and *A*. *mellifera* (Fig. 3A–D) in order to provide insight into their genetic distance and function. The *A*. *hygrophila* CYP2 clan, except for CYPs in the CYP307 family, showed a high degree of 1:1 orthology with CYP proteins from other insect species, suggesting functional conservation of these CYPs^1^. Within the CYP2 clan, CYP 303 A1 showed precise 1:1:1:1 orthologies (Fig. 3A). There are six *A*. *hygrophila* CYPs in clan 2 that belong to the CYP15, CYP18, CYP303, CYP305, CYP306 and CYP307 families. Of these, CYP306A1 and CYP307A1 are encoded by the *Phantom (Phm)* and *Spook (Spo)* genes, respectively. In *Helicoverpa armigera*, a previous study found that these two CYPs are induced by 2-tridecanone, and are known to play a role in the biosynthesis or metabolism of 20-hydroxyecdysone^35,36^. In the mitochondrial clan, the CYPs in *A*. *hygrophila*, except for those in the CYP12 family, show high 1:1 orthology with proteins from other insects (Fig. 3B). Three CYPs from *A*. *hygrophila*, CYP302A1, CYP314A1, and CYP315A1, encode othologs of the Halloween genes *Disembodied (dib)*, *Shade (shd)*, and *Shadow (sad)*, respectively, and this result was consistent with the CYPs in *D*. *melanogaster* and *T*. *castaneum*^2,26^.

The *Agasicles hygrophila* transcriptome contains highly expanded CYP3 (21 subfamilies, 39 individual genes) and CYP4 (15 subfamilies, 30 individual genes) clans, most notably in family 4 and family 6 (Fig. 3C,D). These results show that there is a high frequency of species-specific CYP genes in the CYP3 and CYP4 clan, evidence of CYPome “blooms”^30^. Other insect species also have expanded CYP3 and CYP4 clans; examples are 27 CYP3 clan subfamilies and 15 CYP4 clan subfamilies that form several species-specific clades in the *T*. *castaneum* CYP phylogenetic trees^2^. Similarly, 39 CYP genes in the CYP3 clan and 20 genes in the CYP4 clan were identified in *L*. *decemlineata*^29^. In *A*. *hygrophila*, we identified four CYP families (CYP6, CYP9, CYP345, and CYP347) and 15 subfamilies in the CYP3 clan (Table 3, Fig. 3C). Of these four CYP families, the CYP6 family is evolutionarily related to the CYP3 and CYP5 families in vertebrates^1,37^. The *A*. *hygrophila* CYP6 family has 13 subfamilies, including CYP6A, CYP6AS, CYP6BQ, CYP6BR, CYP6BS, CYP6BW, CYP6CR, CYP6DG, CYP6EF, CYP6FP, CYP6M, CYP6J, and CYP6K. Among them, all CYP6BQs group together in one cluster. Similarly, the CYP9 and CYP345 families also formed one cluster. Although all of the CYP6 families group to a single cluster, they have various functions. For example, transcription of CYP6BQ4 and CYP6BQ8 increased significantly during the first 2 h of exposure to UV-A radiation in *T*. *castaneum*, which could indicate that these two genes play a role in defense against the effects of harmful UV wavelengths^38^. In *Periplaneta americana*, the transcript of CYP6BS1 was more abundant than were those of the other P450 genes in the midgut, suggesting that CYP6BS1 could participate in the degradation of insecticides or other xenobiotics, in addition to other physiological processes and biochemical reactions in *P*. *americana* midguts^39^. Other genes from the CYP6 family also showed multiple functions. In *Cimex lectularius*, CYP6A2 and CYP6A13 were found to be putatively responsible for the resistance of *C*. *lectularius* to pyrethroids^40^. CYP6M7 was confirmed to contribute to pyrethroid resistance in *Anopheles funestus* and was also shown to be important in insecticide metabolism^41^. In the *A*. *hygrophila* CYP4 clan, we found four families and 15 subfamilies (Table 3, Fig. 3D), with the CYP4 family being the largest. The *Agasicles hygrophila* CYP4 family comprised 10 subfamilies, including CYP4AA, CYP4BN, CYP4BQ, CYP4BR, CYP4C, CYP4D, CYP4E, CYP4E, CYP4G, and CYP4Q (Fig. 3D).

CYP4 is the most-studied family in Clan 4 in other insects. CYP4 family members in other insect species are known to be involved in the metabolism of endogenous compounds such as ecdysteroids^42,43^ and pheromone metabolism processes^44,45^, and it has been shown that they have both ω-hydroxylation and ω-1 hydroxylation activity^46^. These results strongly indicate that the CYP4 genes in *A*. *hygrophila* could have multiple biochemical functions and are therefore worthy of further exploration.

### Transcripts expression levels of *A*. *hygrophila CYPs*

**Figure 4:**
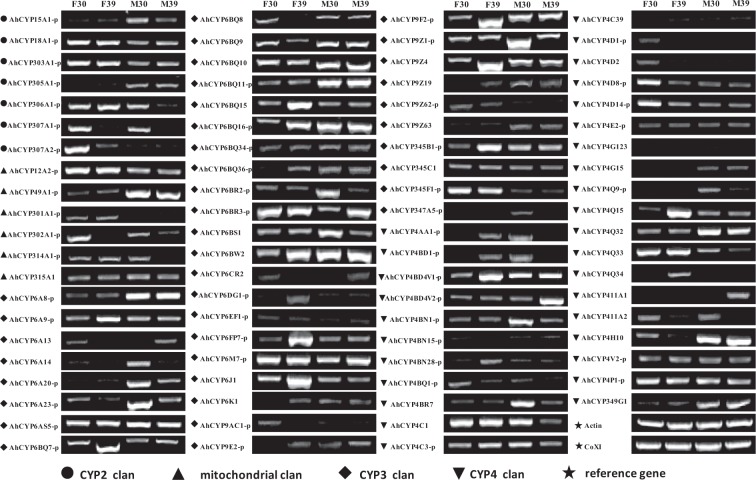
*Agasicles hygrophila* CYP transcript levels in different samples as evaluated by RT-PCR. F30: female adults treated at 30 °C; F39: female adults treated at 39 °C; M30: male adults treated at 30 °C; M39: male adults treated at 39 °C. “AhCYPxx-p” represents “AhCYPxx-partial”. The grouping of gel lanes cropped from different gels, and the full-length gels for each of the *AhCYP* genes are shown in Supplementary Fig. S1.

To determine whether high temperatures can impact the expression of P450 genes in *A*. *hygrophila* adults, we examined the expression profiles of 82 CYP genes that we identified after high temperature treatment. We used RT-PCR to analyze the expression profiles of the *AhCYP* genes in the four samples (female 30 °C, female 39 °C, male 30 °C, male 39 °C). The results showed that the *AhCYPs* have distinct expression profiles in the four samples, and that all of the *AhCYP* genes showed expression in both female and male adults. Based on the analysis, 22 *AhCYP* genes were found to be highly expressed, and 19 *AhCYP* genes had low levels of expression in female adults after high-temperature treatment (Fig. 4). In *A*. *hygrophila* male adults, eight *AhCYP* genes (*AhCYP306A1*, *AhCYP314A1*, *AhCYP6BR3*, *AhCYP6M7*, *AhCYP6J1*, *AhCYP4BD4V2*, *AhCYP411A1*, *AhCYP411A2*, *AhCYP6CR2*) were up-regulated and 12 *AhCYP* genes (*AhCYP307A1*, *AhCYP6A14*, *AhCYP6A20*, *AhCYP6A23*, *AhCYP6BR2*, *AhCYP9Z1*, *AhCYP345A5*, *AhCYP4BN1*, *AhCYP4BR1*, *AhCYP4C1*, *AhCYP4AA1*, *AhCYP4BD1*) were down-regulated significantly (Fig. 4). We can speculate from these results that the highly expressed *AhCYP* genes (22 *AhCYP*s in female adults and eight *AhCYP*s in male adults) may participate in the defense against high temperature, and the metabolic processes involving the *AhCYP* genes (19 *AhCYP*s in female adults and 12 *AhCYP*s in male adults) showed low expression levels that may have been impacted by high temperature.

**Figure 5:**
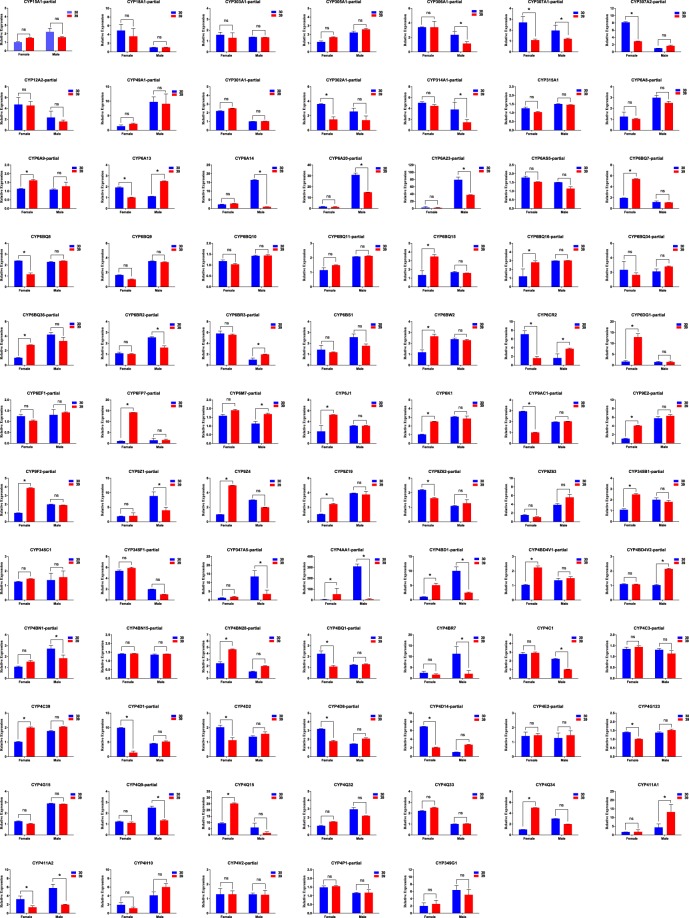
*Agasicles hygrophila CYP* gene mRNA levels in different samples as evaluated by qRT-PCR. 30: female or male adults treated at 30 °C; 39: female or male adults treated at 39 °C. “AhCYPxx-p” represents “AhCYPxx-partial”.

The RT-PCR results were confirmed by performing quantitative real-time PCR (qRT-PCR) assays to measure and quantify the expression levels of the 82 *AhCYPs* in female and male adults at both 30 °C and 39 °C (Fig. 5). The results showed that expression levels of 22 *AhCYP*s were higher in the high-temperature treatment than they were in the control in female adults; among these gene, the expression levels of 12 *AhCYP* genes (*AhCYP6BQ7*, *AhCYP6BQ15*, *AhCYP6BQ16*, *AhCYP6BQ36*, *AhCYP6DG1*, *AhCYP6FP7*, *AhCYP9E2*, *AhCYP9F2*, *AhCYP9Z4*, *AhCYP4Q15*, *AhCYP4Q34*, and *AhCYP4AA1*) were significantly higher (3-fold) in the high-temperature treatment. In the *A*. *hygrophila* male adults, eight *AhCYP* genes were shown to be more highly expressed in the high-temperature treatment group compared to the control. The qRT-PCR results were consistent with the RT-PCR results. Furthermore, 19 and 12 *AhCYP* genes were confirmed to show low expression after high-temperature treatment in female and male adults, respectively. It is worth mentioning that the expression of four *AhCYP*s (*AhCYP9AC1*, *AhCYP4D1*, *AhCYP4D14*, *AhCYP6CR2*) in female adults and four *AhCYP*s (*AhCYP6A14*, *AhCYP347A5*, *AhCYP4BD1*, *AhCYP4AA1*) in male adults were reduced by 3-fold in the high-temperature treatment compared to the control group. The qRT-PCR results also showed that there were no differences in expression in 30 *AhCYP* between the high-temperature and control groups in both *A*. *hygrophila* female and male adults.

## Discussion

The cytochrome P450 monooxygenases are a large and diverse class of enzymes found in virtually all organisms in all kingdoms of life. In insect tissues, CYPs function in the synthesis and degradation of ecdysteroids and juvenile hormones^6^ and the detoxification of extraneous chemicals of natural or synthetic origin, and are crucial for growth, development, and reproduction in insects. Uncovering additional information about the functions of P450s may help us to understand more about the lives of insects, so the functions of P450s that have not been clarified in insects are perhaps of even greater interest.

As increasing numbers of P450 genes are discovered and their functions elucidated, the study of P450 functions will be a research hot spot. In our research, we identified 82 *CYP* genes in adult *A*. *hygrophila* using RNA-seq, transcriptome assembly and bioinformatic analysis. These included members of four *CYP* clans; the mitochondrial clan, and the CYP2, CYP3, and CYP4 clans. Amplification of these *CYP* sequences with specific primers and Sanger DNA sequencing showed that our transcriptome data is reliable. The number of P450 genes found in *A*. *hygrophila* is greater than the number of P450s identified in other insect species such as *Cnaphalocrocis medinalis* with 36 CYP genes^3^, *Apis mellifera* with 48 CYPs^31^ and *Pediculus humanus humanus* with 38 *CYP* genes^47^, and our *CYP* dataset of 82 genes showed an expansion of the *AhCYP* family, which could provide diversity to the P450 monooxygenases that are involved in many complex metabolic processes in *A*. *hygrophila*. However, the number of P450 genes in *A*. *hygrophila* is relatively low compared to the numbers identified in other model insect species including *D*. *melanogaster* (90 *CYP* genes)^37^, *Anopheles gambiae* (111 *CYP* genes)^48^ and *Tribolium castaneum* (143 *CYP* genes)^2^.

The molecular phylogenetic analysis of 236 CYP proteins from five different insect species (*A*. *hygrophila*, *T*. *castaneum*, *D*. *melanogaster*, *L*. *decemlineata* and *A*. *mellifera*) indicated that the insect CYPs have differentiated over time into several groups (Fig. 3), consistent with the results of a previous study^1^. Some coleopteran CYP genes from *A*. *hygrophila*, *T*. *castaneum*, and *L*. *decemlineata* showed a high degree of sequence homology and were located on the same branch of the phylogenetic tree with high bootstrap support (Fig. 3). Examples are CYP305A1, CYP18A1, and CYP306A1 from the CYP2 clan and CYP49A1, CYP302A1, CYP314A1, and CYP315A1 from the mitochondrial clan that were clustered into one clade for three Coleoptera species (*A*. *hygrophila*, *T*. *castaneum* and *L*. *decemlineata*). This finding indicates that the *CYP* genes from these three beetle species may have originated from a single ancestor gene and then differentiated during speciation.

From previous studies, we have concluded that the CYPs in the CYP2 and mitochondrial clan were mostly associated with the ecdysteroid metabolism pathway, such as *CYP18A1*, *CYP307A1* and *CYP306A1* in *Helicoverpa armigera*^35^, *CYP301A1* in *Drosophila melanogaster*^49^ and *CYP314A1* and *CYP315A1* in *Nilaparvata lugens*^50^. Therefore, we speculate that these genes from mitochondrial and CYP2 clans in *A*. *hygrophila* may be involved in the metabolic pathway of ecdysteroid, so the specific function of these genes needs further study. The phylogenetic analysis showed that CYP3 and CYP4 are the two largest of these four clans in *A*. *hygrophila*, and that these two clans contain a high frequency of species-specific CYP gene “blooms”. For example, genes in the CYP6BQ, CYP6BS, CYP6BR, CYP6A, CYP9Z, CYP9D and CYP4D families were mostly clustered in one species-specific clade. This phenomenon revealed that the *CYP* genes might have one ancestral gene, and functional diversity appeared after a long time to adapt to the changing ecological environments.

Fluctuating temperatures are important factors that affect the growth, development, and reproduction of insects, especially high temperatures, which can hinder the metabolic processes in insects and thus are considered to be a significant environmental stress. High temperatures affect the activity of enzymes and the rate of enzymatic reactions, which affect the metabolism process of insects under heat stress^24^. Under the influence of high temperature, many gene families are involved in heat stress^24,31^. Among them, CYP is a typical family involved in metabolism. To gain a more complete understanding of the function of these 82 *AhCYP* genes in responding to high temperatures in *A*. *hygrophila* adults, we evaluated their expression levels using RT-PCR and qRT-PCR. The results showed that the expression patterns of the *AhCYP* were different in *A*. *hygrophila* adults after treatment with high temperature. Some of these CYP genes such as *AhCYP6A9*, *AhCYP6BQ7*, *AhCYP6DG1* and *AhCYP4Q34* were up-regulated in *A*. *hygrophila* female adults, while *AhCYP307A1*, *AhCYP6A13*, *AhCYP6BQ8* and *AhCYP4D8* were down-regulated in female adults. In *A*. *hygrophila* male adults, some AhCYP genes such as *AhCYP306A1*, *AhCYP307A1*, *AhCYP6BR3* and *AhCYP4BD4V2* had relatively high expression levels after high-temperature treatment, while the *AhCYP* genes such as *AhCYP6A20*, *AhCYP6A23*, *AhCYP4C1* and *AhCYP4BR7* had relatively low expression levels (Fig. 5). We can speculate that the differential expression of the CYP genes after heat treatment may be one of the harmful consequences caused by heat stress. We can also speculate that one of the reasons for the decline of the *A*. *hygrophila* population caused by high temperature may be that the expression of some CYP genes changed under high temperature, which partly affected the metabolic process of *A*. *hygrophila*, and eventually maybe affected its reproduction and population propagation.

In the *A*. *hygrophila* CYP2 and mitochondrial clans, we can conclude from previous studies that *CYP15A1* can catalyse epoxidation of methyl farnesoate into juvenile hormone III^6^ and that *CYP18A1*, *CYP303A1*, *CYP305A1*, *CYP306A1*, *CYP307A1*, *CYP307A2*, *CYP301A1*, *CYP302A1*, *CYP314A1*, and *CYP315A1* are involved in ecdysone synthesis^49,51–53^. Ecdysteroids regulate many endocrine processes in insects and are critical for successful development, moulting, and reproduction^54^. Our results show that *AhCYP 306A1* and *AhCYP 302A1* are expressed at low levels in male adults and that *AhCYP 307A2* and *AhCYP 314A1* are also expressed at low levels in female adults, while *AhCYP 307A1* shows a low level of expression in adults of both sexes. These results indicate that elevated temperatures can affect transcription of *AhCYP306A1*, *AhCYP302A1*, *AhCYP307A2*, *AhCYP314A1*, and *AhCYP307A1* in *A*. *hygrophila*. From the fact that *AhCYP 307A1* was down-regulated in both female and male adults, we can speculate that high temperature may affect the process of ecdysone synthesis, but the mechanism needs further study.

In the CYP3 and CYP4 clans, we found some genes in *A*. *hygrophila* such as *AhCYP6CR2*, *AhCYP4AA1* and *AhCYP4BD1* that showed sex-specific differences, and the differences in female and male adults were not consistent after high temperature treatments. For example, the expression levels of *AhCYP4AA1* increased significantly after high temperature treatment in female adults, while it decreased significantly in male adults. From this phenomenon, we speculate that a different metabolic mechanism exists in *A*. *hygrophila* female and male adults when they respond to high temperatures. From the qRT-PCR results, we found many unigenes that showed sex-specific differences (Fig. 5), which needs further investigation. In addition, we found 30 *AhCYP* genes in which there were no differences in expression levels between the high-temperature and control treatments in both *A*. *hygrophila* female and male adults, which illustrates that these *AhCYPs* are probably not involved in the resistance of *A*. *hygrophila* to high temperatures, and that they could function in in other aspects of diverse physiological processes.

In conclusion, based on the transcriptome analysis, we identified 82 *AhCYP* genes in female and male adults of *A*. *hygrophila* in our study. Our work represents an important first step in understanding how CYP genes function in the high temperature response in *A*. *hygrophila*. We performed a comprehensive analysis of the expression patterns of the *AhCYP* genes in different samples using RT-PCR and qRT-PCR. Our results show that the *AhCYP* genes have multiple expression levels in female and male adults after high temperature treatments. The changes in the relative expression of *AhCYP* genes in response to high temperatures will require further study by RNAi or CRISPR/Cas9. In our study, we also constructed phylogenetic trees of the AhCYPs with other insect CYP proteins to understand the diversity of *A*. *hygrophila* P450 genes and the evolutionary relationships of CYPs among insects. The results presented here provide a starting point for further functional studies of *AhCYP* genes at the molecular level.

## Materials and Methods

### Host plants and insect rearing

The host plants of *Alternanthera philoxeroides* were collected from a pond in the Institute of Plant Protection, Hunan Academy of Agricultural Sciences (IPP, HAAS), Changsha, Hunan province in summer 2017. The plants were grown in plastic boxes (40 cm in length, 18 cm in width, and 15 cm in height) containing sterilized nutritional soil in the greenhouse at Langfang Experimental Station, Chinese Academy of Agricultural Sciences (LF, CAAS). The plants were used for experiments when they had four- to six-internodes with leaves and stems.

*Agasicles hygrophila* adult beetles were collected from the wild in Changsha, Hunan province. The *A*. *hygrophila* adults were reared in the laboratory on *A*. *philoxeroides* plants under controlled conditions: a temperature of 28 ± 2 °C, relative humidity of 75 ± 5%, and a photoperiod of 12 h light and 12 h dark. The beetles were reared for three generations on potted *A*. *philoxeroides* plants prior to conducting the experiments^55^. Five pairs of newly emerged (<12 h) *A*. *hygrophila* adults were selected and placed in a plastic bottle (8.5 cm in diameter, 10 cm in height, with absorbent wool and wet filter paper at the bottom to provide humidity) containing fresh *A*. *philoxeroides* stems. The experiment was conducted in an environmental growth chamber (Prx-450D-30, Ningbo Saife Instruments, Ningbo, China). Two environmental growth chambers were set up with the same relative humidity of 75 ± 5% and a 12-hour photoperiod, and the temperature conditions were as described in Table 4.

**Table 4:** Temperature regimes used in the heat-shock treatments.

Treatment Temperature (°C)	Time of day (hours)			Temperature Mean (°C)
	0:00–10:00 (10)	10:00–14:00 (4)	14:00–24:00 (10)	
Control	28	30	28	28.3
Treatment	28	39	28	29.8

### RNA extraction, cDNA library construction and Illumina sequencing

After treatment for 7 days, we collected samples from the *A*. *hygrophila* female and male adults; each sample contained 10 individuals (5 individuals from the control group and 5 individuals from the high temperature-treated group) of a single gender with three replicates. Total RNA was extracted from control female and male *A*. *hygrophila* adults using TRIzol reagent (Life Technologies, Carlsbad, CA, USA) following the manufacturer’s protocols. Construction of the cDNA libraries and Illumina RNA sequencing were performed by Novogene Bioinformatics Technology Co. Ltd (Beijing, China). The RNA sequencing libraries were prepared with 3 μg mRNA that was purified from total RNA using oligo-dT paramagnetic beads. Fragmentation of the purified mRNA was performed using divalent cations atn elevated temperature in NEBNext First Strand Synthesis Reaction Buffer (5X). First-strand cDNA was synthesized using M-MuLV reverse transcriptase (RNaseH) and random hexamer primers, and second-strand cDNA synthesis was then performed using DNA polymerase I and RNase H. The cDNA fragments were amplified by PCR and purified using the AMPure XP system (Beckman Coulter, Beverly, USA) following end repair and Illumina adaptor ligation. The index-coded samples were clustered on a cBot Cluster Generation System using TruSeq PE Cluster Kit v3-cBot-HS (Illumina) as directed by the manufacturer. After cluster generation, the cDNA libraries were sequenced on an Illumina Hiseq 2500 instrument and paired-end sequencing reads were generated.

### *De novo* assembly and functional annotation

After removing short reads, low quality reads, and adaptor sequences, the remaining clean reads from *A*. *hygrophila* female and male adults were assembled using the short read assembly program Trinity (r20140413p1)^56^. The resulting unigenes were further clustered using TGICL to remove redundant sequences and to obtain the longest possible non-redundant unigenes^57^. The assembled transcripts were used as queries in searches against the nr protein database (NCBI non-redundant protein sequences), Nt (NCBI non-redundant nucleotide sequences), Swiss-Prot (a manually annotated and reviewed protein sequence database), KO (KEGG Ortholog database), and GO (Gene Ontology) to identify putative protein functions using the BLAST algorithm with an E-value cut-off of 10^−5^ ^58^.

### Transcript abundance analysis of the AhCYP genes in the transcriptome data

The read number for each unigene was converted to RPKM (Reads Per Kilobase per Million mapped reads) to calculate the transcript abundance of these unigenes^59^ using the following formula: RPKM (A) = (1,000,000 × C × 1,000)/(N × L), where RPKM (A) is the expression of CYP gene A, C is the number of reads that are uniquely aligned to CYP gene A, N is the total number of reads that are uniquely aligned to all unigenes, and L is the number of bases in CYP gene A. The RPKM method eliminates the influence of gene length and sequencing depth on the calculation of gene expression.

### Identification of the cytochrome P450 genes by cloning and sequencing

All of the CYP gene sequences identified in the *A*. *hygrophila* adult transcriptome were verified by gene cloning and Sanger sequencing. We designed gene-specific primers to amplify and clone the complete or partial sequences of each CYP gene using Primer Premier 5 software (PREMIER Biosoft International, CA, USA) (Supplementary Table 1). The cDNAs were synthesized from 2 µg of female and male adult RNA using the TransScript II One-Step gDNA Removal and cDNA Synthesis SuperMix Kit (TransGen, Beijing, China). PCR amplification was performed using EasyTaq DNA polymerase (TransGen, Beijing, China) in 20 μl reactions containing 200 ng of template cDNA. The PCR cycling conditions were an initial denaturation at 94 °C for 4 min, followed by 40 cycles of 94 °C for 30 s, 55 °C for 30 s,and 72 °C for 2 min, with and a final extension step at 68 °C for 10 min. The amplified DNA fragments were gel-purified and cloned into the pEasy-T3 vector (TransGen, Beijing, China), and the inserts were sequenced using standard M13 primers.

### Phylogenetic analysis of the AhCYP genes

The candidate AhCYP gene fragments were identified by homology searches using the Blastx and Blastn tools in NCBI. The annotated CYP sequences from the representative insect species *T*. *castaneum*, *D*. *melanogaster*, *L*. *decemlineata* and *A*. *mellifera* were downloaded from the NCBI reference sequences (RefSeq) database and the cytochrome P450 monooxygenase homepage (http://drnelson.uthsc.edu/CytochromeP450.html). The insect CYP amino acid sequences were aligned using the program ClustalW and edited using the BioEdit Sequence Alignment Editor 7.1.3.0^60^. A total of 236 CYP protein sequences (Supplementary Table 2) from *T*. *castaneum*, *D*. *melanogaster*, *L*. *decemlineata* and *A*. *mellifera* were included in the phylogenetic analysis this included the 82 *A*. *hygrophila* CYPs identified in this study. A phylogenetic tree was constructed using the neighbour-joining method as implemented in MEGA 7.0 as described in Kumar *et al*.^61^. The statistical bootstrap support of each branch was assessed by re-sampling the amino acid positions 1,000 times.

### Expression analysis by RT-PCR

The expression of *AhCYP* transcripts in female and male adults after response to different temperatures (male adults at 30 °C and 39 °C, female adults at 30 °C and 39 °C) was examined by RT-PCR. The *Actin* (GenBank accession number: KF792064.1) and *CoxI* (GenBank accession number: FJ977926.1) genes of *A*. *hygrophila* were used as internal control genes to assess cDNA integrity. Specific primers for the target and control genes were designed with Primer Premier 5 software (Supplementary Table 3). Each reaction (25 μl volume) contained 200 ng of cDNA template. Amplification conditions were 95 °C for 4 min, followed by 30 cycles of 94 °C for 30 s, 55–60 °C for 30 s (depending on annealing temperature), 72 °C for 1 min with a final extension step of 10 min at 72 °C. The amplified DNA fragments were examined on a 1.2% agarose gel and confirmed by DNA sequencing.

### qRT-PCR measurement

The relative mRNA abundance of *AhCYP* genes in female and male *A*. *hygrophila* treated at different temperatures were further determined by qRT-PCR. The *A*. *hygrophila* reference genes *Actin* (GenBank accession number: KF792064.1) and *CoxI* (GenBank accession number: FJ977926.1) were used for normalization of gene expression. Specific primer pairs for the qRT-PCR were designed using Beacon Designer 7.9 software (PREMIER Biosoft International, CA, SA) and are given in Supplementary Table 4. qRT-PCR assays were conducted using an ABI Prism 7500 system (Applied Biosystems, Carlsbad, CA, USA). Each qRT-PCR reaction and amplification conditions were performed as described in the instruction book of TransStart Tip Green qPCR SuperMix Kit (TransGen, Beijing, China). Negative controls containing no template DNA were included in each experiment. Each qRT-PCR experiment was performed with three biological replicates, and each biological replicate was repeated three times. The relative transcript levels in each sample were calculated by the comparative 2^−ΔΔCT^ method^62^. All data were analysed using the ANOVA and Duncan’s new multiple range test (P < 0.05) to compare the expression of each target gene between the various samples by using SPSS Statistics 18.0 software (SPSS Inc., Chicago, IL, USA).

## Electronic supplementary material

Supplementary information
