# Supplementary material for: Identification of cytochrome P450 monooxygenase genes and their expression in response to high temperature in the alligatorweed flea beetle Agasicles hygrophila (Coleoptera: Chrysomelidae)
Source: Sci Rep. 2018 Dec 14;8:17847. doi: 10.1038/s41598-018-35993-1 (PMC6294762; doi:10.1038/s41598-018-35993-1)
Supplement: Supplementary file 1 — Supplementary information [file 41598_2018_35993_MOESM1_ESM.doc]

Supplementary Information for

**Identification of cytochrome P450 monooxygenase genes and their expression in response to high** **temperature in** **the alligatorweed flea beetle *Agasicles hygrophila* (****Coleoptera: Chrysomelidae)**

Hong Zhang , Meiting Zhao, Yiran Liu, Zhongshi Zhou, Jianying Guo*

State Key Laboratory for Biology of Plant Diseases and Insect Pests, Institute of Plant Protection, Chinese Academy of Agricultural Sciences, Beijing, China

* Correspondence should be addressed to Jianying Guo, PhD. Email: guojianying@caas.cn

E-mail addresses of authors:zhanghong19900301@126.com

Supplementary Figures

Supplementary Figure S1. The gels for each AhCYP genes presented in Figure 4.

Supplementary Tables

Supplementary Table 1. Primers used for intact ORF or partial sequences validation of each *Agasicles hygrophila* CYP genes.

Supplementary Table 2. The protein names and sequences of the 236 CYPs used in Figure 3.

Supplementary Table 3. Primers used in RT-PCR for determination expression levels of *Agasicles hygrophila* CYP genes.

Supplementary Table 4. Primers used in real-time PCR for determination expression levels of *Agasicles hygrophila* CYP genes.

Supplementary Table 5. Cytochrome P450 monooxygenase genes identified in the *Agasicles hygrophila* adult transcriptome.

Supplementary Figure S1. The gels for each AhCYP genes presented in Figure 4.

*AhCYP15A1*-partial  *AhCYP18A1*-partial *AhCYP303A1*-partial


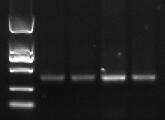


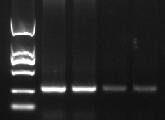


**291 bp**


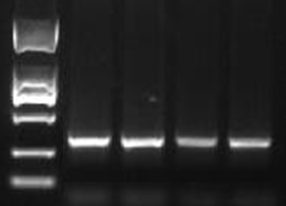


**288 bp**

**2000 bp**

**500 bp**

**250 bp**

**355 bp**

*AhCYP305A1*-partial *AhCYP306A1*-partial *AhCYP307A1*-partial


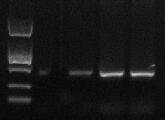

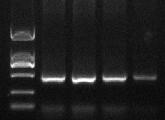

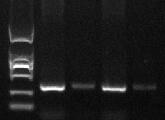


**2000 bp**

**500 bp**

**250 bp**

**505 bp**

**482 bp**

**298 bp**

*AhCYP307A2*-partial *AhCYP12A2*-partial *AhCYP49A1*-partial


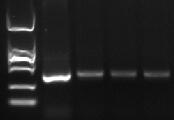

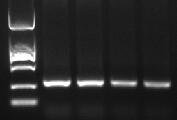

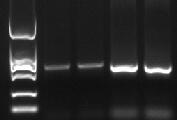


**2000 bp**

**500 bp**

**250 bp**

**436 bp**

**279 bp**

**537bp**

*AhCYP301A1*-partial *AhCYP302A1*-partial *AhCYP314A1*-partial


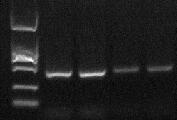

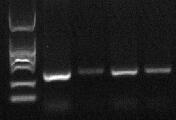

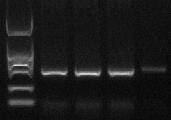


**2000 bp**

**500 bp**

**250 bp**

**439 bp**

**284 bp**

**503 bp**

*AhCYP315A1 AhCYP6A8*-partial *AhCYP6A9*-partial


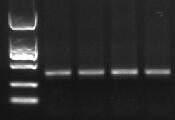

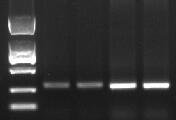
 **
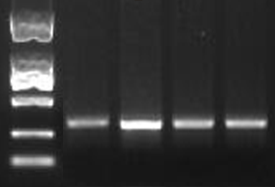
**

**2000 bp**

**500 bp**

**250 bp**

**464 bp**

**293 bp**

**280 bp**

*AhCYP6A13 AhCYP6A14 AhCYP6A20*-partial


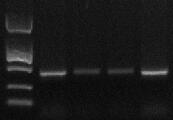

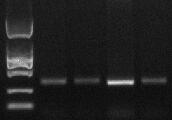

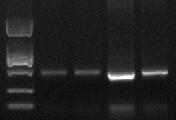


**2000 bp**

**500 bp**

**250 bp**

**376 bp**

**365 bp**

**508 bp**

*AhCYP6A23*-partial *AhCYP6AS5*-partial *AhCYP6BQ7*-partial


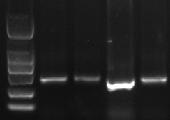

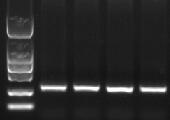

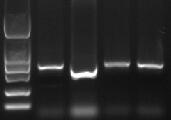


**2000 bp**

**500 bp**

**250 bp**

**534 bp**

**290 bp**

**619bp**

*AhCYP6BQ8 AhCYP6BQ9 AhCYP6BQ10*


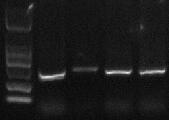

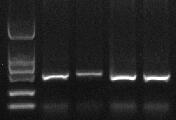

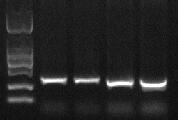


**2000 bp**

**500 bp**

**250 bp**

**507 bp**

**527 bp**

**295 bp**

*AhCYP6BQ11*-partial *AhCYP6BQ15 AhCYP6BQ16*-partial


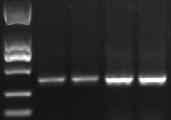

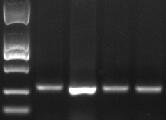

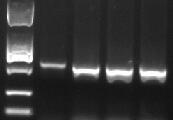


**2000 bp**

**500 bp**

**250 bp**

**288 bp**

**512 bp**

**510 bp**

*AhCYP6BQ34*-partial *AhCYP6BQ36*-partial *AhCYP6BR2*-partial


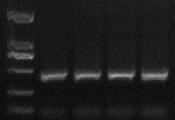

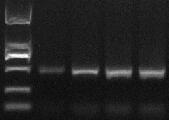

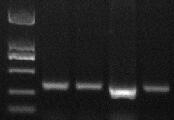


**2000 bp**

**500 bp**

**250 bp**

**455 bp**

**420 bp**

**295 bp**

*AhCYP6BR3*-partial *AhCYP6BS1 AhCYP6BW2*


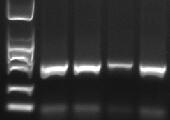

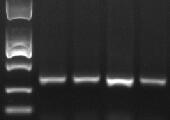

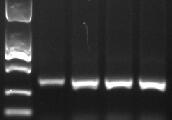


**2000 bp**

**500 bp**

**250 bp**

**522 bp**

**288 bp**

**290 bp**

*AhCYP6CR2 AhCYP6DG1*-partial *AhCYP6EF1*-partial


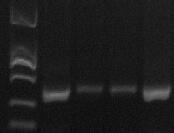

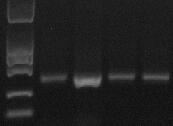

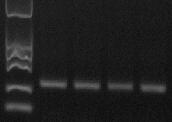


**2000 bp**

**500 bp**

**250 bp**

**299 bp**

**285 bp**

**311 bp**

*AhCYP6FP7*-partial *AhCYP6M7*-partial *AhCYP6J1*-partial


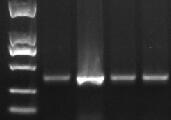
 **
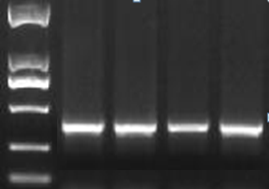
**
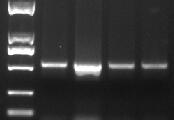


**2000 bp**

**500 bp**

**250 bp**

**417 bp**

**352 bp**

**526 bp**

*AhCYP6K1 AhCYP9AC1*-partial *AhCYP9E2*-partial


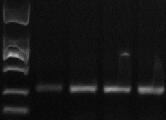

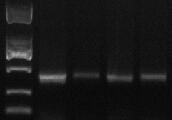

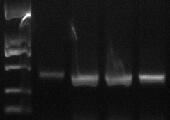


**2000 bp**

**500 bp**

**250 bp**

**505 bp**

**448 bp**

**446 bp**

*AhCYP6F2*-partial *AhCYP9Z1*-partial *AhCYP9Z4*


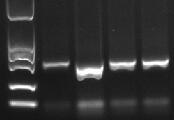

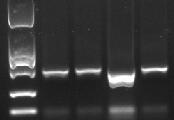

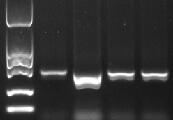


**2000 bp**

**500 bp**

**250 bp**

**504 bp**

**515 bp**

**522 bp**

*AhCYP9Z19 AhCYP9Z62*-partial *AhCYP9Z63*


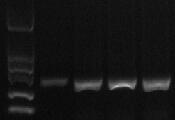

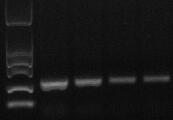

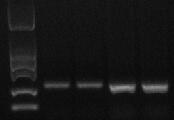


**2000 bp**

**500 bp**

**250 bp**

**489 bp**

**275 bp**

**295 bp**

*AhCYP345B1*-partial *AhCYP345C1 AhCYP345F1*-partial

**2000 bp**


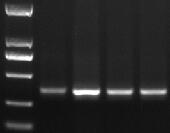

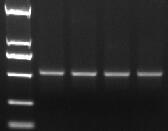

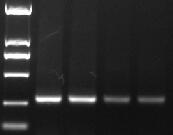


**500 bp**

**250 bp**

**340 bp**

**518 bp**

**289 bp**

*AhCYP347A5*-partial *AhCYP4AA1*-partial *AhCYP4BD1*-partial

**2000 bp**


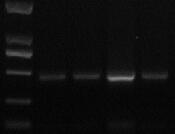

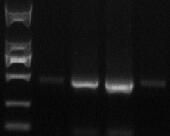

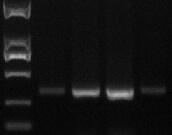


**500 bp**

**250 bp**

**503 bp**

**484 bp**

**289 bp**

*AhCYP4BD4V1*-partial *AhCYP4BD4V2*-partial *AhCYP4BN1*-partial

**2000 bp**


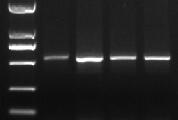

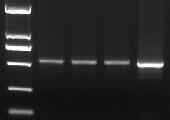

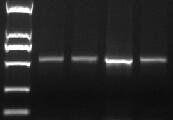


**500 bp**

**250 bp**

**514 bp**

**515 bp**

**545 bp**

*AhCYP4BN15*-partial *AhCYP4BN28*-partial *AhCYP4BQ1*-partial

**2000 bp**


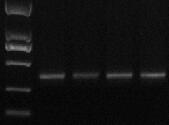

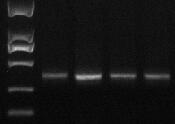

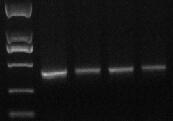


**500 bp**

**250 bp**

**295 bp**

**311 bp**

**351 bp**

*AhCYP4BR7 AhCYP4C1 AhCYP4C3*-partial

**2000 bp**


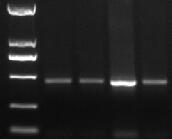

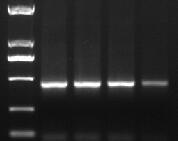

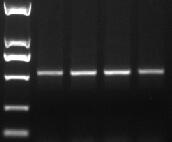


**500 bp**

**250 bp**

**456 bp**

**482 bp**

**546 bp**

*AhCYP4C39 AhCYP4D1*-partial *AhCYP4D2*

**2000 bp**


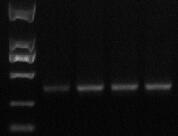

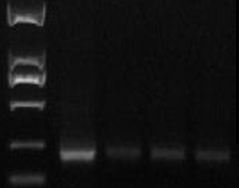

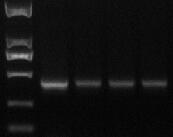


**500 bp**

**250 bp**

**351 bp**

**201 bp**

**357 bp**

*AhCYP4D8*-partial *AhCYP4D14*-partial *AhCYP4E2*-partial

**2000 bp**


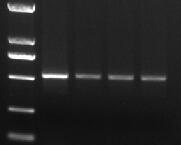

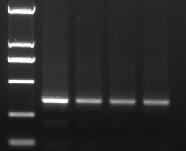
 **
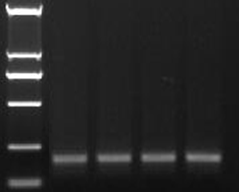
**

**500 bp**

**250 bp**

**521 bp**

**424 bp**

**189 bp**

*AhCYP4G123 AhCYP4G15 AhCYP4Q9*-partial

**2000 bp**


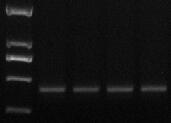

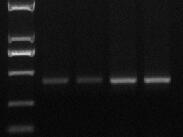

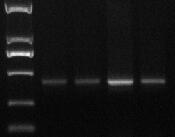


**500 bp**

**250 bp**

**350 bp**

**357 bp**

**344 bp**

*AhCYP4Q15 AhCYP4Q32 AhCYP4Q33*


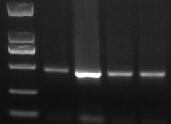

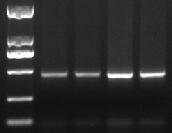

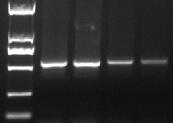


**2000 bp**

**500 bp**

**250 bp**

**417 bp**

**523 bp**

**526 bp**

*AhCYP4Q34 AhCYP411A1 AhCYP411A2*


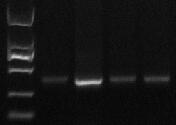

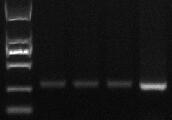

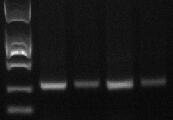


**2000 bp**

**500 bp**

**250 bp**

**434 bp**

**285 bp**

**299 bp**

*AhCYP4H10 AhCYP4V2*-partial *AhCYP4P1*-partial


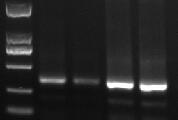

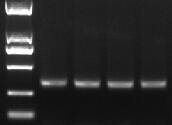


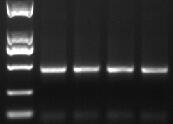


**448 bp**

**2000 bp**

**500 bp**

**250 bp**

**324 bp**

**295 bp**

*AhCYP349G1 Actin CoxI*


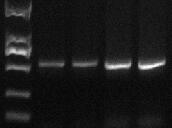


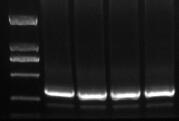


**274 bp**


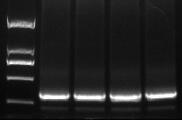


**282 bp**

**2000 bp**

**500 bp**

**250 bp**

**543 bp**

**Supplementary Table 1. Primers used for intact ORF or partial sequences validation of each *Agasicles hygrophila* CYP genes.**

| Gene name | Forward primers (5'-3') | Reverse primers (5'-3') | **Amplicon size (bp)** |
| --- | --- | --- | --- |
| *AhCYP15A1* | AGAGAAGACTTCGATG | AGAGATAGTTTTTCTAT | 507 |
| *AhCYP18A1* | ATGTTTGTGTACAGTGCC | GAAACCATTTAATTTCG | 1203 |
| *AhCYP303A1* | GATGTTTTAGTAATAGCCA | TGTTATTCTAGCTTTAA | 309 |
| *AhCYP305A1* | ATGGTGATAGAACTGCTGA | TTATAATAAA AAATGTCT | 1341 |
| *AhCYP306A1* | ATGATGATTACTTATCTAGTGG | CTAACCTGCTTGAAAAGGCA | 1299 |
| *AhCYP307A1* | GCACATACATTTCCACGAGC | TTATCTTTGTGCAAATTT | 1056 |
| *AhCYP307A2* | ATGCTCGCGCTCGTTGTG | TCAAGATCTGGGAGTTAGC | 1455 |
| *AhCYP12A2* | AAAATCAAAACTGCAAATAAC | TCATTTTGTA ACAGATA | 825 |
| *AhCYP49A1* | ATGATAACGATGTTTGTCC | CTAAAATGAAGACAAAT | 708 |
| *AhCYP301A1* | TTGTGTTTGAAAAATATCATGC | CTAAATGATA AAATTGTA | 672 |
| *AhCYP302A1* | CACAGTTTTGGAAAAATACC | TTACTTTTCCATATATTC | 783 |
| *AhCYP314A1* | ATGATAGACGAAAGCCTGTAC | CTAAACTCTTTCTCTAA | 1476 |
| *AhCYP315A1* | ATGTATATGAGACCGCG | TTAATCGATTTTACGAA | 1410 |
| *AhCYP6A8* | ATGGCTTTCACCTTCTCTTTAG | GTACAATCTTTTTAAATT | 1504 |
| *AhCYP6A9* | TTTAAAGTGGTGGGTAAAG | CATAAATTGTCCATTTAAG | 397 |
| *AhCYP6A13* | ATGTTTATAATTGTAGTG | TTACATTGTAGAATCGTCC | 1509 |
| *AhCYP6A14* | ATGTCTATAATAACAGGAGAC | CTATATCCTCTCGACATCC | 1533 |
| *AhCYP6A20* | AAGGATTTTATGCATTTG | ATCTCTTTTTACTAATT | 777 |
| *AhCYP6A23* | ATGGCTAAGGACTTTAATTA | TTAGGAAACCTTATCGAG | 1239 |
| *AhCYP6AS5* | GCTTACCAAGAAACTTTAAG | GTGCTTTTTAACAAAAAT | 381 |
| *AhCYP6BQ7* | CAATGGATTTCAACGTATTGG | TAAATTACCTTTAAG | 1020 |
| *AhCYP6BQ8* | ATGTTGACAGTATTGTTG | TCACTTCTGAATTTTCTCC | 1536 |
| *AhCYP6BQ9* | ATGCTGATAATTTTAATAG | TTAATTAATTTTTTCC | 1518 |
| *AhCYP6BQ10* | ATGTTTTTATATTCACTGATAG | TTACACTTCTTTAAGATT | 1527 |
| *AhCYP6BQ11* | ATTGTTCGTCAAGA | TTAATTTTGTATTTTTTC | 750 |
| *AhCYP6BQ15* | ATGCTGTTGCCTGTAAC | TTATTCAATAGCTTC | 1551 |
| *AhCYP6BQ16* | GCTGCAGTAATATTATTG | TAACAAATGCATAAAATCC | 798 |
| *AhCYP6BQ34* | ATGTTATGTATTACAATA | AACGTCATCAGAAACCTCC | 861 |
| *AhCYP6BQ36* | TCAGGAAAAATGAAAATG | TCATCTCTCAGTTATATC | 1122 |
| *AhCYP6BR2* | ATGCTGTTACTATTTGG | AGTTTCTTGGTAAGCTTG | 1058 |
| *AhCYP6BR3* | ATGGCTAAGTTTACGACC | TTATAACGCTGTTGCTTC | 963 |
| *AhCYP6BS1* | ATGGGCTTTTTAACCATTTC | TCATCTGGGACAAAAGTT | 1512 |
| *AhCYP6BW2* | ATGTTGCTCTACATAATAA | TTATTTTACTTCTTTAATG | 1530 |
| *AhCYP6CR2* | ATGATTGACTTTTCCAGC | TTAGTTGTTTTCGATT | 1551 |
| *AhCYP6DG1* | ATGTTGTTCATTTTACTTA | CTACAACCTTTCCGC | 1497 |
| *AhCYP6EF1* | ATGTCAGCCGCGATATAC | TTACTCTTTATGTAGC | 1470 |
| *AhCYP6FP7* | TGTTTAGCTCTTTATGG | ATGCACCTTTAGTAAC | 597 |
| *AhCYP6M7* | TCCTCTGCTAAAATAAA | ATAGCTTAAATTTTCC | 426 |
| *AhCYP6J1* | ATGCTTATTACTTCATC | TTAGGTTAATCGTACAATCC | 1500 |
| *AhCYP6K1* | ATGTCTCTTTTTGCTC | TTAGCTTTTAACAAGTGG | 1512 |
| *AhCYP9AC1* | CTTTTCGTTCTTAAAGG | ATCAGAGCTTGAGCTGC | 633 |
| *AhCYP9E2* | GGGCAAAGATGGAA | TTAATAATGTAAAGGC | 1179 |
| *AhCYP9F2* | ATGTTAAGTGCGGTATT | CTTGGCTTCCATCATCAA | 840 |
| *AhCYP9Z1* | ATGCTACTCAGGATATAC | TTATTTAACTGTACTAT | 1446 |
| *AhCYP9Z4* | ATGTTCTGGATATTGATAC | TTAACATTTTTCCAATCCC | 1566 |
| *AhCYP9Z19* | ATGTGGGGAATACTTCT | TTATTTAAGTTTCTTCAG | 1566 |
| *AhCYP9Z62* | CAAGGATCGGTATTGTATTGG | TGTTAACACCATATCC | 1023 |
| *AhCYP9Z63* | ATGATTCTTGTTTGTT | TTAGGAAGAGCTAATT | 1578 |
| *AhCYP345B1* | ATGCTGCTGACCAC | TTATTTGATCTTCTTAA | 1497 |
| *AhCYP345C1* | ATGCTGCTGACCCAA | TTATAATTTCTTAAATCG | 1521 |
| *AhCYP345F1* | ATGCACATCAACGTC | TTACAACTTTGTGACG | 1494 |
| *AhCYP347A5* | ATGTGGTTGATATTGG | TCAGTTTATTTTCTTAA | 1497 |
| *AhCYP4AA1* | ATGGACTACTACCTAGGC | CTATGAAATAAGATTACC | 1452 |
| *AhCYP4BD1* | CAGGAAAAAGTATTGGC | TTAATATATCCTATGC | 513 |
| *AhCYP4BD4V1* | TATTTACAAGACGTACCG | AAGTGAAATATGGACCCC | 1386 |
| *AhCYP4BD4V2* | ATGTTCGCGGTAATAGG | TGGATACCGAGACAAACAGT | 987 |
| *AhCYP4BN1* | ATGTATTTACTTACATT | TTAAGTTCTTTTCTTTAA | 1458 |
| *AhCYP4BN15* | GGATTAAGATGGAAAC | AAACATAAAAGTATCG | 561 |
| *AhCYP4BN28* | TGGAAGAAAAACCG | TCAGATCTTCCTTTTCTTC | 1128 |
| *AhCYP4BQ1* | CCAACTGTGCCAAGATGGC | CTACCATTTTCTTTTAACC | 1446 |
| *AhCYP4BR7* | ATGTTAACTGCATTGTTAG | TCATCTTTTTCTTTTTTC | 1458 |
| *AhCYP4C1* | ATGGCGTTCTTGGGTGTGA | TTAGTCCAATTTTCTTAAAG | 1521 |
| *AhCYP4C3* | GAATCCTTTTTGCAATTGC | TCATCTTTTTCTAATGG | 906 |
| *AhCYP4C39* | ATGATACTAGCAGGG | CTACGTTCTTCTTTGAAGCC | 1476 |
| *AhCYP4D1* | GGATTTGTTCCTATGTTGC | AGAGCTCTTAAATACTCC | 201 |
| *AhCYP4D2* | ATGTCGATATTAGTT | CTATTGTATCTTTGTTTG | 1485 |
| *AhCYP4D8* | TTTGACGAAATTATA | CCCATTTTTGATGTTCAGC | 696 |
| *AhCYP4D14* | ATGTTTGTGTTAAGTG | TGGTTTTAACTTAATTTTG | 1500 |
| *AhCYP4E2* | AAAATTGTACTTGGG | AAAGTCGTAAGTCAA | 192 |
| *AhCYP4G123* | ATGACACAGATGCTGATTG | TTAAACTTTTAAATTAC | 1701 |
| *AhCYP4G15* | ATGTCAGCAACAGTGATACC | CTAAGCAGCCCTAAGCTGG | 1686 |
| *AhCYP4Q9* | ATAATTTATTTGTTAC | ATGTCCCTCAAACATAA | 909 |
| *AhCYP4Q15* | ATGTCACAACCTACTTTG | TCATACTCTTTCTGAC | 1677 |
| *AhCYP4Q32* | ATGACCATTTTACTTCAAG | TTATTGATGAATGATAT | 1521 |
| *AhCYP4Q33* | ATGTTAATTATTGCTGCTG | TCAAATTAAGCTATTTAA | 1518 |
| *AhCYP4Q34* | ATGATTGAATTGTTGTGG | CTATCTTGGAACAAATTTAAC | 1482 |
| *AhCYP411A1* | ATGCCTTTACTTTTAATAGC | CTAGAACCATTCTCTCTTC | 1479 |
| *AhCYP412A2* | ATGTTAACAGGAAAC | TTATCTCTTTTCTACCC | 1554 |
| *AhCYP4H10* | ATGATATTTTTAATTTTTG | CTAACGCTGTCTTTTTTG | 1467 |
| *AhCYP4V2* | TTAACACCTACTCTAC | ATTTGAAAAACTATGC | 372 |
| *AhCYP4P1* | AAAAACGATACTTTTAATT | GGGAAACTGATGCTTTT | 453 |
| *AhCYP349G1* | ATGGAGGCCTTATG | TTACTTTCTATATGTAAG | 1560 |

Supplementary Table 2. The protein names and sequences of the 236 CYPs used in Figure 3.

>Ah CYP15A1

KRLGIVFSDGQFWQNQRKFSIQHLRSFGFGRREMEEKIQEETLSLIEIFMKKHTEPIYMHDAFDVPVLNGLWAMMAGQRFDIDDKRLRKLLQILHDAFRLVDISGGMLNQMPFLRYIAPNASGYTRIKQILERMWDFLEETISDHRKTIS

>Ah CYP18A1

MFVYSAMVLWNLAIKEFSTHILLVFLTVLFVVRALQKLKEAFSLPPGPWGLPILGSLPFLKSDLHLHYRDLTQKYGSLISTRLGSQLIVVLSDYKMIRDIFRKEDFTGRPDTEFSNILGGYGVINIAGKLWKDQRRFLHDGLRHFGMSYLSSRKTQMESRIMSEVEDFLLVLKAGQGQSVDLNPVFAVSISNVICDVLMSVRFSHNDKKFIRFMNLIDEGFRLFGSLEAAIFIPILRYLPGHTLTRQKIAKNREEMGKFLQETINEHKCTFDPSHMRDLLDTYLYEIQKADEEGTGHHLFEGRDHDRQMQQIMGDLFSAGMETIKSSLQWAVLFMLHHPDKMLAVQEELDQIIGRQRLPKLEDLTYLPVTESTIYEVLRISSIVPMGTTHAPIRDTKLNGF

>Ah CYP303A1

DVLVIANLHSCMLEKGSDYKNPEQFKPERFLQDGKIVLPDSFMPFGIGKHRCLGESLARANVFIFLASLLQKFDFSIVPDHPPTTEWLDGITPGPKPFKARIT

>Ah CYP305A1

MVIELLIGVITALLLYYVVFSTRKPRNFPPGPPWLPFLGSSVVVKRLARSLGGQQKAFGKLSEIYNSDVIGLKLGKEQIIVVQSYETVKKILTGDEYIGRPDNYFWRLRCMGKISGITSTDGETWRQQRHFVTTHLRNLGFGKSPMERMIRDEVDDLLDYFKKTEEVNISNVLSTSVLSVLWSLVSGYRLDRSDSRLLRLLDILDARGRAFDMSGGTLNQFPWLRFVAPEKTGFNIIQNLNSEMKKLLIEIITQHKSISKDDRSDDLVYSYIRQMKEEDNTFTEEHLMMVIVDIFIAGAQSTSNTLTFAIMVMIMYPDLQKKVKRSLEDAFTWTEKISYSERSRIPYVEAVLCEVERYYNIIPIGGPRRVLRDTTLNNYFIPQNTTVLINLHSVHHDKDYWGDPEIFRPERFLDSNGKLMYHDRLIPFGLGKYPKPLRCTRRHFLL*

>Ah CYP306A1

MMITYLVAVVILLLVFMYWRNRDMPPGPWGLPVIGYLPWLDANNPYLSLSELTKKFGHVYGLRLGSIYTIVLSDPKIIKNALSKDATTGRAPLKLTHGIFQGYGLICAEGERWREHRKFSANFIRQISVKNSLKKDKLESLILHHCSEFTNYLASLDDISSINVFPALKHGLGSVINTLVFGKSWDRNNELWKYLLYLQEEGPKYIGVAGPLNFFPFLRFIPKYKKIISFIKDGQKNTHKEYRKIIEEQTALMDKLNTNSSPDNIIQAYVAEKKKKKPQIAEIYSDKQLCYLLADLFGAGLDTTAVTLRWFLVYLAKYPAIQQEIRNELNEVLEGRKPSIEDLALLPLMEASIWETMRIRPVIPTGLPHGTLEDCYIEGYKIPKGSMLLPLIWHLHNNPEVWEDPNKFNPKRFIDKEGKVVKSEYIMPFQAG*

>Ah CYP307A1

AHTFPRAFTNNFFNLETVILTETQNMFSLVTPNEITNLKQILQKCCGNIFWTHFCSKSFKYNDPKFIEVTENFDEVFYEVNQGYAADFLPFLSPLLTKNIKRMNRLTHRIRKFVEEEVIQDKFEQFDVESEPNDYVETLIKNAKSEDIDLSWNTALFALEDIVGGHSAVSNFLVKVFAYLVKEPEVQRKIQKEIDDNVGINKDVSLSDRNSMPYTEATIYEAIRLIASPIVPRVANRDSSINGYKIEKDSVIFMNNYELNMSNELWKDPKKFMPERFLSNGTLVKPEHFLPFGGGRRGCLGYRMVQLISFGVIGGFLQNFTILPVKGEDYKVPVGSLALKKDTFKFKFAQR*

>Ah CYP307A2

MLALVVLCIVLFVCWFTRRNKKDIANYPPGPKPWPVIGSLHLLGQHSTPFEGFTALSKIYGDIFSISLGSTPCVVVNNFELIKEVLITKGGDFDARPNFIRFHKLFGGDRNNSLALCDWSDLQRTRRSIARTYCSPRFTSLQYDMVANVGTLEIPVFLDEINKLPRNSPIDIKPLVLSACANMFTQYMCSTSFPYHDKEFQKVVRYFDEIFWEINQGYAVDFLPWLLPFYKNHMQKITNWSTEIRQFILSRIIDNHRATLDYNSPPRDFTDALLMHLEEDPNMNWQHIIFELEDFIGGHSAIGNLIMVTLASLIKYPEIAAKVQKEIDEVTGASRLPNLLDKSSMPYTESVIWETLRMASSPIVPHVASTDTEILGYNVPKGTMVFINNYDLNVSPNYWNKPDNFMPERFLSLAGHVVKPNHFIPFSTGKRTCIGQRLVQCFSFVIITTLLQRYDVKAGGNLNIKPGCVAVPPDCFKVLLTPRS*

>Tc CYP15A1

MLFFVTLVISLVLLFLILDTIKPRRYPPGPKWLPIVGNFLEFRRRLSEIGYHHLVWKEFSEEYGDVVGLKMGRNLVVAVFGAEAVKEVLTREEFDGRPDGFFFRLRTFGKRLGIVFSDGQFWQKQRKFSMQHLRNFGFGRKEMEEKIEEETKDLIAVFKKQCSEPIWMHTAFDVSVLNVLWAMMAGERFNINDERLRKLLKIVHDAFRLTDMSGGMLNQLPFLRFIAPETCGYNQLVDVLVRMWEFLQETISEHRKTLCSSHARDLIDAFLQKMDIQSDSSFTDDQLMSLCLDLFMAGSETTSNTLGFSVVYMLQFPEVQKKVQDEMDEVVGRNRWPTLQDRIKLKYTEAVLMEIQRRANIPPLGIAHRATRDVSLFDYRIPEGTIVLTSLYSVHMDHKFWKDPLAFRPERFLNKEGNLEVDEKYFAPFGYGKRRCLGESLAKANYFLFFTALLHNFYLEKDCDGPEPQLEGYDGVTISPKPFRAKLIPRTD

>Tc CYP18A1

MFVYSGLVLWNFLAEELSTKVLAVFLMVLFLVRLVQMLVQMLKEASALPPGPWGLPILGSLPFLKGDLHLHFRDLTHKYGSLISTRLGSQLIVVLSDYKMIRDAFRKEEFTGRPITEFTTLLDGYGVINTAGKLWKDQRRFLHDGLRHFGMSYIGSRKTQMENRIMREVEEFLSVLTARKDTPIDLNPVLAVSLSNVICDILMSVRFSHNDERFKRFMFLIDEGFKLFSSLEASFFIPILKYLPGQRQTREKIAKNRAEMAQFLQETIEEHRKSFDPSHLRDLLDTYLYEIQKADEEGTGDHLFEGKDHDRQMQQIMGDLFSAGMETIKSSLQWAVLFMLHHPEVMKAVQEELDQVVGRRRLPKLEDLPYLPVTESTMLEVLRISSIVPMGTTHAPTRDLKLNGFHLPRHAQIVPLLHSVHMDPSLWHEPERFNPSRFINAEGKVVKPEYFLPFGVGRRMCLGEILARMEIFSFFSSLLHSFDICVPTGETLPSLKGVAGVTISPNAFRVCLKPRPMEWDSMGTIRPAGSH

>Tc CYP305A1

MWLAVLLFFVFVVTYVIQQVKKPPNFPPGPYWLPFVGNLPQLKKLSKKLGGQHLALSHLAKEYNTNVLGLKLGKDYVVTVFTYPLIRDVLISEEFEGRPDNFFLRLRCMGQKRGITCTEGEEWNTLRNFVNRHLRNLGFGKKPMEKMIQDEICDILTILKKDGSDIQVDKFLAPCVLSVIWTLITGEKISRENNQLDELLDLFNLRSKAFDMTGGTLTQYPWLRYFLPEWSGFNLIKSVNTSLKNLFMKYIREHKDGWFEGRNEDLIYRYISEMKTNTEASKYFTDDHLVMVCVDLFIGGAQTTSRTLGFAFLMMIMYPEVQKKVQEQIDKHYDKNSSIEYSDRYKLPYVEAVLLETIRYRYVVPIGGPRRVTKNTTLDGYYLPKNTTVLISFYSINNDPENWQNPEIFNPERFLDEKGSLLPDEKLIPFALGRRRCVGEILAKNCIFLLFVEILRRYNVSLAPGSKPPTGKPIAGITLSPESYRVKFTERSL

>Tc CYP306A1

MAPLWSVLLSFLLFIIAYTWLRRSRNLPPGPWNLPVIGYLAWLNPQFPYLTLTSLSKKYGPIYGLYLGSIYTVVISDAKLIKKTFNKDASSGRAPLHLTHGIMKGFGLICAQGDLWKDQRKFVHNTLRTLGASKVSPNRPTMEALILHHVSDLVQHIKSLGESVTLDPLDSLRHSLGSAINQMVFGKCWSRDDATWKWLQHLQEEGTKHIGVAGPLNFLPLLRFLPKSKQTINFLTEGIRDTHQIYREIIEEQKNSSNETVSNVIQAFLAEKEKRKNEDSVKFYNDQQFHYLLADIFGASLDTTLTTLRWYVLYMAVHQDVQKKVRSLLNDLTLEQIAMVPYFEATIAEVQRIRPVVPVGIPHGSVEELEIAQYKVPPGTMIVPLQWAVHMDANIWDEPEVFKPERFINEEGKFFKPEAFIPFQAGKRMCVGDELARMFLYLFGAALVKNFAISCMGEVDLTGDCGITLTPKPHELIFTSL

>Tc CYP307A1

MLALLCLCVVLLVWWFSRPKKSPSTIPGPRPWPLIGSMHLLAGHETPFQAFTALSRVYGDIFSIHLGSASCVVVNNFKLIKEVLIAKGGDFGGRPDFARFHKLFGGDRNNSLALCDWSSLQKTRRSIARTYCSPRFTSLQYDRVNNVGEEELKSFLHQLDQLPHGQPCNVKPAVLMVCANMFTQYMCSTSFAYEDKGFQKIVRYFDEIFWEINQGYAVDFLPWLLPVYTGHMKKISNWATEIRQFILSRIIDKHRATLDTNSPPRDFTDALLMHLEEDPNMNWQHIIFELEDFLGGHSAIGNLVMVTLAAVVDHPEVAKRIQEEVDQVTGGTRCPNLFDKAAMPYTEATILETLRTASSPIVPHVASKDTEIDGHEVSKGTIVFINNYELNQGDAYWDEPGLFKPERFLSSTGNIVKPAHFIPFSTGKRTCIGQRLVQCFSFVVLATLLQYYDVSTKESVKVQPGCVAVPPDCFKLVLTPRK*

>Dm CYP303A1

MFYTVIWIFCATLLAILFGGVRKPKRFPPGPAWYPIVGSALQVSQLRCRLGMFCKVIDVFARQYVNPYGFYGLKIGKDKVVIAYTNDAISEMMTNEDIDGRPDGIFYRLRTFNSRLGVLLTDGEMWVEQRRFILRHLKNFGFARSGMMDIVHNEATCLLQDLKDKVLKSGGKQTRIEMHDLTSVYVLNTLWCMLSGRRYEPGSPEITQLLETFFELFKNIDMVGALFSHFPLLRFIAPNFSGYNGFVESHRSLYTFMSKEIELHRLTYKNYDEPRDLMDSYLRAQDEGNDEKGMFSDQSLLAICLDMFLAGSETTNKSLGFCFMHLVLQPEIQERAFQEIKEVVGLERIPEWSRDRTKLPYCEAITLEAVRMFMLHTFGIPHRAVCDTRLSGYEIPKDTMVIACFRGMLINPVDFPDPESFNPDRYLFDGHLKLPEAFNPFGFGRHRCMGDLLGRQNLFMFTTTVLQNFKMVAIPGQVPEEVPLEGATAAVKPYDIMLVAREQ

>Dm CYP304A1

MITETLLTICAAVFLCLSYRYAVGRPSGFPPGPPKIPLFGSYLFMLIINFKYLHKAALTLSRWYKSDIIGLHVGPFPVAVVHSADGVREILNNQVFDGRPQLFVAAMRDPGQDVRGIFFQDGPLWKEQRRFILRYLRDFGFGRRFDQLELVIQEQLNDMLDLIRNGPKYPHEHEMVKSGGYRVLLPLLFNPFSANAHFYIVYNECLSREEMGKLVKLCQMGIQFQRNADDYGKMLSIIPWIRHIWPEWSGYNKLNESNLFVRQFFADFVDKYLDSYEEGVERNFMDVYIAEMRRGPGYGFNRDQLIMGLVDFSFPAFTAIGVQLSLLVQYLMLYPAVLRRVQNEIDEVVGCGRLPNLEDRKNLPFTEATIREGLRIETLVPSDVPHKALEDTELLGYRIPKDTIVVPSLYAFHSDARIWSDPEQFRPERFLDADGKLCLKLDVSLPFGAGKRLCAGETFARNMLFLVTATMCQHFDFVLGPNDRLPDLSQNLNGLIISPPDFWLQLQDRH

>Dm CYP305A1

MSALIFLCAILIGFVIYSLISSARRPKNFPPGPRFVPWLGNTLQFRKEASAVGGQHILFERWAKDFRSDLVGLKLGREYVVVALGHEMVKEVQLQEVFEGRPDNFFLRLRTMGTRKGITCTDGQLWYEHRHFAMKQMRNVGYGRSQMEHHIELEAEELLGQLERTEEQPIEPVTWLAQSVLNVLWCLIAGKRIARQEDGTLRRLLDLMNRRSKLFDICGGLLAQFPWLRHVAPDRTGYNLIQQLNTELYGFFMDTIEEHRRQLAKDPSPAESDLIYAYLQEMKDRSAGGESSTFNETQLVMTILDFFIAGSQTTSNTINLALMVLAMRPDVQEKLFSQVTASVAAASTDAFPHLSRREAFDYMDAFIMEVQRFFHITPITGPRRALWATKLGGYDIPKNATILISLRSVHLDKEHWKDPLEFRPERFIDSAGKCFKDEYFMPFGMGRRRCLGDALARACIFSFLVRIVQHFSVVLPAGESPSMVLLPGITLTPKPYKVQFVKRT

>Dm CYP306A1

MSADIVDIGHTGWMPSVQSLSILLVPGALVLVILYLCERQCNDLMGAPPPGPWGLPFLGYLPFLDARAPHKSLQKLAKRYGGIFELKMGRVPTVVLSDAALVRDFFRRDVMTGRAPLYLTHGIMGGFGIICAQEDIWRHARRETIDWLKALGMTRRPGELRARLERRIARGVDECVRLFDTEAKKSCASEVNPLPALHHSLGNIINDLVFGITYKRDDPDWLYLQRLQEEGVKLIGVSGVVNFLPWLRHLPANVRNIRFLLEGKAKTHAIYDRIVEACGQRLKEKQKVFKELQEQKRLQRQLEKEQLRQSKEADPSQEQSEADEDDEESDEEDTYEPECILEHFLAVRDTDSQLYCDDQLRHLLADLFGAGVDTSLATLRWFLLYLAREQRCQRRLHELLLPLGPSPTLEELEPLAYLRACISETMRIRSVVPLGIPHGCKENFVVGDYFIKGGSMIVCSEWAIHMDPVAFPEPEEFRPERFLTADGAYQAPPQFIPFSSGYRMCPGEEMARMILTLFTGRILRRFHLELPSGTEVDMAGESGITLTPTPHMLRFTKLPAVEMRHAPDGAVVQD

>Dm CYP307A1

MLAALIYTILAILLSVLATSYICIIYGVKRRVLQPVKTKNSTEINHNAYQKYTQAPGPRPWPIIGNLHLLDRYRDSPFAGFTALAQQYGDIYSLTFGHTRCLVVNNLELIREVLNQNGKVMSGRPDFIRYHKLFGGERSNSLALCDWSQLQQKRRNLARRHCSPREFSCFYMKMSQIGCEEMEHWNRELGNQLVPGEPINIKPLILKACANMFSQYMCSLRFDYDDVDFQQIVQYFDEIFWEINQGHPLDFLPWLYPFYQRHLNKIINWSSTIRGFIMERIIRHRELSVDLDEPDRDFTDALLKSLLEDKDVSRNTIIFMLEDFIGGHSAVGNLVMLVLAYIAKNVDIGRRIQEEIDAIIEEENRSINLLDMNAMPYTMATIFEVLRYSSSPIVPHVATEDTVISGYGVTKGTIVFINNYVLNTSEKFWVNPKEFNPLRFLEPDNEKLQLKRNIPHFLPFSIGKRTCIGQNLVRGFGFLVVVNVMQRYNISSHNPSTIKISPESLALPADCFPLVLTPREKIGPL

>Ld CYP18A1

MFVHDSTVLWKLVSEAYSTQVLLATSLVLLVMKLIQVIKQEWNLPPGPWGLPIVGSMWSIKGSELHLYYQDLAKKYGPIFSVRLGSQLCVVLSDPKQIREVFRKEEFTGRPTNEFTNILGGYGVINIAGDLWKQQRRYLHASFRRFGMSYFNARKDLMEKRIMDEVNSCIGYLRTIGGKPVDTSEVFAVNVSNVICGMMMSVKFSHNDVQFIRFMNLIEEGFRLFGSLEVANFIPTLRILPFCFGGVVAKIEQNRAEMAEYMQEKIDDHKKTFDPGNPRDLLDMYVNDVSLAMKEGNVDRLFHGKDPDRQIQQIIGDLFSAGMETIKSSLQWSILFMLHYPDEMKAVQEELDEVVGRGRLPALDDISYLPITNATLNEILRIANIVPLGTTHAPTRVIKLGKYTLPKHAQVVPLLHYVHMNPDLWDEPEKFNPSRFISSVGKLLNPECFLPFGEGRRKCLGEVMARMEIFLFFSSILHSFDLSVPEDHPLPSLKGIAGVTLNPNAFKVCLKDRPLIGYAN

>Ld CYP305A1

IVTVVSLVIAYLIKSIRRPPNFPPGPIWLPIIGNLHQVKKLSIAQGGQHLAFSKLSQDYNSNVIGLKLGGKYTIVIASYPLVKTVLTSEEYDGRPQNFFMILRSLGTGKGITGTDGELWRIQKHFVSVHLRQLGLGKTAMDAQIQAEASEILVIIEENVNKELQIGQILPKSVLNILWMLMSGLRLSRNDDRLIKLLNLLHKRAKAFDMAGGTLNQMPWLRFVTPEWTGYNLIRNINEEFMTLLMEPIREHFKKWTDDRNDDLIYSFITEMRKAEGEKSTFTEDQLLMVCLDIFLAGAQTTSNTLDFAFLMMLLYPEIKNKVHACLDEAFQKSEEITYSERKRVPYVEAVLYETERFCTIAPIIGPRRVLRKTTLNGYDIPKDTTVLIHIHSVHNDVDYWQDPEIFRPDRFLDPKENLMYHDRFLPFGLGKRRCLGEILAKNRIFTFFSQIMKSYQIELPPNAKKPTGVPQPGITLSPE

>Ld CYP306A1

MGAFVPSYRMVLVVLILVTALTLLVTYYIYKRRGLPPGPWGLPIIGFLPWLDPIAPYLTLTKLSKKYGSIYGLSLGNVYAVVLTDPKIIKSLFSNDSTTGRAPLYLTHGIMKGYGLICAEGELWREQRRFVHNCIRQFGGSKIGQQRTKMEKLIMTHANEFVNYIGNSGEDTALDPLEPLRHSLGSAINLFVFGISWSRNDDIWIWLQEIQEEGTKHIGVAGPLNFLPFLRFLPKYQRTMKFLLDGKHKTHELYQKIIDEQVGDLDSDKSATNILQAFLLEKTRKSEEIVNRFYNDQQFYHLLADLFGAGLDTTLTTLRWYLMYLAKNKSIQENIRSEISDVLQGRPPTLEDMPHLPLLEASICEAQRIRPVVPVGIPHGTMAELVIEGYRIPKGTMIVPLQWAMHMNEEVWNDPEKFDPYRFLDDEGKVVKNAHFMPFQIGKRMCVGDELARMILFLFITTIIQNFSFVLENEEVDFSGECGITLTPKSHKIKFSKIMK

>Ld CYP307A1

MGIDQLNFSADMLAIALLCVVLCIFYCKKRKSDSLPPGPAPWPLIGSLHLLGQHETPFQAFTALSKIYGDFFSITLGSSRCIVVNNFDLIREVLITKGKDFGDCPNFQRFHRLFGGDRNNSLALCDWSDLQKKRRSIARSYCDPKCTSLKFDNANVVAINEMDLFMNELQKLPMNEPVDLKTVVLQACANMFTQYMCSTRFSYQDEDFRRAVRHFDEIFWEINQGYAVDFMPWLKPFYEGHMKKLSFWAQEIRTFILERIIDSHKRTIDYNSTPRNFTDALLMQLQEDSTLNWEHIMFELEDFIGGHSAVGNLVMVTLAALVKYPHVAKRIRKEVDTVTKNERIPNLYDRANMPYTDCVLWETLRKSSSPIVPHVASDKTELQGYDIPKGTTVFINNYELNVGKQYWKNPEEFMPERFLSPTGVVVKPTHFIPFSTGKRTCIGQKLVQSFSSVIIASLLQKYDITGGSDVELRVGSMAVPPDCFKLILTPRKAPTIPEEE

>Ld CYP303A1

MWLAIILFSILLCILAFLDTRKPKNFPPGPKWWPIFGSAPQIAQLQKKTGHLLYATSEMSKKYGPILGIKVGRETIVVVHGTRANKEFLFSDDMAGRPLGDFYDLRTWGKRKGILLVDGKFWQDQRRFFVKQLREFGFGTKNMSTLVEEEAQELVEYIFRTIRNNDSTLFNIQSLLTVHILNSLWKMLAGVRYSAEDGKMRELQDILSELFRSLSMVGTTFSHLPILRYLAPERSGYNLYVNSHNRIWEFLSKEIAHHKQTHNTHEPRDVIDVYLNVLESLDNTNDEFSEEQLLATCMDMFMAGSETTSNTLSFCFLYLILNQDVQRKAQEEIDRVIGKYRNPRLDDRPNMPYVECVVMESLRMFGGRCFTVPHRALKDTELFGYSIPKDVLVIGNLYGAMLEEGGSFEEPAMFKPERFLKNGKISLPEDFLPFGIGKRRCLGESLARANVFLFIAALLQKFTFSISPEHPPTTEWKDGITPTSKILTD

>Am CYP15A1

MLYVVISLLLALYCIFCIYDCVKPHNFPPGPKWLPLIGCFLTFRRLKLKHKYTYVAFQELSKTYGPILGLKLGSQKLVVISTHDLVKKVLLQDEFNGRPDGFFFRVRAFGKRKGILFTEGSMWSQCRRFTMRHLRSFGLGQSTMEKYLTVEAENLVNYLRRVSTKGPVPMHTAFDIAVLNSLWCMFAGHRFDYENEKLAEILEIVHDSFRLMDTMGGIISQMPFLRFIIPELSGYNNLMEILRKLWNFLDEEINNHEKHLSGNQPQDLIEAFLLEISSRNGVQNDSIFDRENLLILCLDLFLAGSKTTTDTLSTSILFLSLHSEWIKILQEELDNVVGRSRSPTLEDYSSLPIMESFLAEIQRFLILAPLGVPHKTTKDVILNGYNIPKDTTVLLDFHSAHNDPAYWDHPEEFRPQRFLDANGRFCQNNANIPFGLGKRRCPGEMLARTSLFLYFAYVIHYFDIEISPEHGKPDLNGHDGFTISPKSYYLKITARSDVTNCSTI

>Am CYP18A1

MLVEHAAQWAWQAMGGTRIEVLCTFLVFLGVLLVARCLQWLRYVRSLPPGPWGVPVFGYLPFLKGDVHLRYGELAKKYGPMFSARLGTQLVVVLSDHRTIRDTFRREEFTGRPHTEFINILGGYGIINTEGAMWKDQRKFLHDKLRGFGMTYMGGGKKIMESRIMREVKTFLRGLASKRGTPTDVSASLGMSISNVICSIIMGVRFQHGDARFKRFMDLIEEGFKLFGSMAAVNFIPVMRYLPCLQKVRNKLAENRAEMAGFFQETVDQHRATFDEGTMRDLVDAYLLEIEKAKGEGRATTLFQGKNHDRQMQQILGDLFSAGMETVKTTLEWAIILMLHHPDAAIAVQEELDQVVGKSRMPVLEDLPFLPITEATILEVLRRSSVVPLGTTHATTRDVTLHGYTIPAGSQVVPLLHAVHMDPELWEKPEEFRPSRFLSAEGKVQKPEYFMPFGVGRRMCLGDVLARMELFLFFSSLMHTFELRSPQGSSLPSLRGNAGVTVTPDPFDVCLLPRNLDLIEDNDMISTGAILRNIGSH

>Am CYP303A1

MMSIIIEESQLLVEYIKKLINDEHNIQINKSKIYCNINEFNDGQIYKLVKKYKKDETKLINNYIINEKELKASDLYMNANEYTGNNVTQSQLGTIISMHNIFGITVLNSLWKMLAGKRYNIDDKELIYFQRILSITLNEIDMLGAPFSHFPLLRFIAPEISGYKSFVKIHEELWKFFKDEVNNHKNTFNSDSPGNLIDIYLTILNSENYGKTFSEPQLVAICVDLFMAGSETTSKVLGFCFLYLVLFPHVQKKAHEEIDRVIGRNKLPTAEDKAKMTYMNAIVLESLRMFAGRSLNLPHRVQRDTKISDYKIPKNTIIITNFNGILMDESWGDPENFRPERFIDGSGNIVTPSRFLPFSAGKHRCMGENLAKTNIFIIATTLLQAFTFSEIPGEKPTIEHFIDGTTISPKPYRVNVSLRI

>Am CYP304A1

MISFLFIIFLLLIIYKIYNSVIHVSSNTPPCLPRLPIIGSYWHLLWHDYEYPYNGIIHYVNKLQSKIVTCYFGSHKTIIANDYKSIKEVLTKQEFNGRPINVDIVLQRAFGKSLGIFFTEGTLWHEQRRFALRHMRDFGFGRRHEIFETNVMEEIAILVDMLKEGPINDEEKKFLKNGYACFPDILYPYVANVILNIMFGERFDRSQYHKLIYFCESSMMFQKSLDTSGGAIFQFWFLKYFGNIFGYTNAIKATYQMINFIEEYIDNKKDLDDYDKGLIGRYLKILKEKNNITSTFSQKQLIMTLVDFMFPATSALPSALVHAIKLVMHHPRVVNNIQEEIDRVVGTGRLVTWSDRKNLPYIEATIRESLRYETLTPLSVFHKTLKKTTLCDYDIPKDTLVVTNLVALNTDPDLWGDPENFRPERFLDENNELRKDFTFPFGFGHRVCPGETYSRYNMFEVFAVLMQNFNFSFVEGEPTGLDDKESGLIVTPKKTWIQVKARNMK

>Am CYP306A1

VIRSIVDGKDKTHEIYRQILDEHRARVDSGNGCKIDSFLAAFDEQMRKKDGAESGYFTEPQLYHLLADLFGAGTDTTLTTLRWFLLFMAAHPMEQEKIQSEMDLCLREGEQPTLNDRIVMPRLEAAIAEVQRIRSVTPLGIPHGTSEDVEIGGYDIPCGAMIVPMQWAIHTDPAYWRDPLEFRPDRFLSEDGTFFKPESFLPFQNGKRVCVGEELA

>Am CYP307B1

NKENSLAFCNWSDVQKARREMLRAHTFPRAFSTRFNELNGIIGDEMEFMVNHLDSLSGTSVHAKPLILHCCANIFITYLCSKNFHLEHDGFRNMVENFDKVFFEVNQGYAADFLPFLMPLHHRNMARMAHWSHEIRRFVIKNIIADRVNSWNDVVPEKDYVDCLINHVKSGTEPQMSWNTALFVMEDIIGGHTAIGNLLVKVLGFLATRPEIQRLAQD

>Ah CYP12A2

KIKTANNNFMDGIFKTLIGVPFWRYFENEAYKQLRVSHEFLFITLKDLIESSRKLYITNPEVLKWSQPFLYSLFSNTRLSEDDCNMLVIETFLGGIDAVATTLALTLHYLALNKTIQEKAHASLSDKSYLRACIKETLRMSPTAGANSRVLVKDTVFDDYLIPKNTLVSVFSSVISRNEEYFPDADRYWPDRWLKGTPNCIHDKFASLPFGFGARTCPGIRIAEAEMVLFLKAVLSNFIIETEDKSEIPMVFRMNRVPERPINLKFIPTISVTK*

>Ah CYP49A1

MITMFVRTSRSISRRCVSAQIHILRMYATDRPYSTTIGILPEAVEHEINDSGITVKDYSLIPGPRELPLIGNSWRFAPIIGHYKIHELDKVMWSLRRDYGRIVKVSGLIGHPDLLFVFDGDEIKKVLKNEEAMPHRPSMPSLHYYKQEFQKKFFEGNEGVIGVHGPKWEAFRKQVQQYLLPPLTAKKYIEPLDMIAMDFLDRMENMLDKNRELPDHFLSEIYKWALECKLHLSSF*

>Ah CYP302A1

HSFGKIPGPLSLPGVGTLYQYFPLVGKYRFDELQHNGFKKYNAFGSLVREEIVPGVNLIWLFDPDDIETLFRNEGKFPLRRSHLALEKFRLDRPYVYNSGGLLPTNGAEWHRIRRNFQKGLTSTLAVKKFITGSNEIIQEWLIEVDNMRNSNTDFLPELSRLFLELTGQTTFNVRLNSFSKSERNKNSRSSRIIECAYSTNSCILKTDNGPQLWRKFDTPLYRKLKRSQEYMEK*

>Ah CYP314A1

MIDESLYSFNSFNYIILIIVFIIFGYRPPWFSRKSVDWVQREIPEIPGPLALPILGTTRPRFLGGFNINKIHEYYLELYKKYGPIVKEEALFNVPVISVFEKSDIEKVLKSCGGKYPMRPPCEAQMVYRRSRPDRYVSTGLVNEQGPVWHHLRTSLTTDLTSPKTISGFLPQAEEISEDWCNLLRQTRTNGDIVEHLEDLVNKLGLETSCSLVLGRRLGFLLPGQETNLAKRLADAVHSHFIATRDTYYGLPFWKLYNTSAFKKLADSEETIYKLASELIQSADDATAESSVFQSIIKADIDEREKTAAIIDFIAAGIHTLKNSLVFILYLVGRNAGVQQKILEDKTKSYLKACIRESFRIYPTAYSLARITQEDLDLSGFKVKGGSVVICHTGVAGKNAQYFKDPLEFRPERWLGAEKSSTASNATFLVTPFGAGKRICPGKRLMESVLPIFLENTVKNFEIDVVNPMEIQFEFLLAPKGPTSMIFRERV*

>Ah CYP315A1

MYMRPRFIKVVAARVPQNGFSTKALDFGSIPTSKWRLPIIGSTLDLILSGETAKYHKYMDRKHRELGPIFKDNIGPTTAIFVSDAHEIRNIFAKEGVYPMHYLPEAWAIYNKKHNFVRGLLFMDGPEWLHFRRIVNPILLGRDLAWLEQACDISTNDLIHRFELENGREIANIEHTLYRWSIDVIVGVLVGGSNYKKCAEEVGDLLQTLAETVHRVLDDTVVLQFINPKLAEKFNLKMWRKFEESVTTSTYLTRQLLGILINKYQGEDGLLHKLIQGNIGKDLDRIIIDLIMSAGDTTSYTMTWILYLLCKNQDVIQAIRESEPKSHLLRNSIKEALRLHPVAPFINRYLPKDVTICGYNIPAKTLVTMSFYSCGRNPEYFKHPDRFLPNRWVRGSEEYAALPLASIPFGLGARSCIGRRIAENQLKMTTEKLIRKFEIQLLNADEIDDVMRMISKPSKPLRLVFRKID*

>Tc CYP12C1

MSTGSENVISQKKLDHYKNLNALENSDVMPEGWDKAKPFESLPGPKPIPFFGNIWRFLVPKIGDFYGIDFLDLHKIFYEQYGDISILKGMINDPIVVIFNPKDYETLFRNEGIWPIRKGLQSFNEYRKSRKDIFVNAGLLLEEGENWFKIRSVVNPILMQPRNISQYTDKMNSVADELVNNITNLLEESENGEMPENFHNELYKWSLESVGLVTLDTHLGCLKNDLDEDSEPQKLIRSTLEMFKLMHKLDVLPSFHNYVSTPSWRKFVQVMDFIVETNMKYVNQVLDKLEKGETTSTEIPSVLEKLLKVDRNIAVTMSIDMMIAGIDTTGRILGAALYFLGKNHEAQEKLRSEAISLLQTKDQAVTSEVLKKAPYLKAVIKETTRLAPIGIGNLRTTVKNLVLGGYQIPKGTDVVTSNLVLCTNDEYFSRAKEFIPERWLSTTSGELSKKNTNPFIFAPFGYGPRSCVGKRLASLELEVALLKIIRNFELDWPHEDMVFKTKMLYGMTEPLKIHVRSV

>Tc CYP49A1

MSLSRKLLVPKRALAVAQRAYSTDRPYSTAIMPELLDELAIPEHVERVEATPRPYSAIPGPKELPLIGNAWRFAPIIGQYKIQELDKVMWSLNRDYGRIVKVGGLIGHPDLLFVFNGDDIEKVFRMEEAMPHRPSMPSLHYYKQILKKDFFDGNAGVIGVHGPKWEEFRKKVQHALLPPQIAKKYIEPLDVIAGDFLHRMEDMLDENQELPNHFLSEIYKWALESVARVSLDTRLGCLEPNLSQNSESQRIINSINTFFWNVAEVELKMPVWRVYKNRSFKKYIGALEDFRTLCLKHIHKSMEKMQEKNFDEIKEENISIVERILLKTDNPKLAAVLALDLLLVGVDTTSIAAASTIYQLSQNPEKQQKLFDELQQVLPENDSKIDVSIQDKMPYLKACIKETLRMYPVIIGNGRSLQTDTVIAGYKVPKGTHVIFPHLVVSNSEDYFHEPHRFLPERWLKTENACPMHKKIHPFVTLPFGYGRRSCLGRRFAEAELQILLAKIFRKYKVEYNYGPLSYKITPTYVPEQPLKFKLVKRE

>Tc CYP301A1

MVATSKFPQAAKIVKNITRSRSQTVTPAFCPHLENLNEAKPYSEIPGPKPIPILGNTWRLLPIIGQYDVSDVAKLSELFYKEYGKIVKLSGLVGRPDLLFIYDANEIEKIYRQEGPTPFRPSMPCLVRYKSVVRKDFFGDVGGVVGVHGEPWRRFRSTVQKPILQVQTVKKYIEPIENVTNYFIQRMMEMQNDDQEMPSDFDNEIHKWALECIGRVSLDARLGCLDPNLPPDSEPQKIIEAAKYALRNVAILELKFPFWRYFPTSLWTRYVQNMDYFVEICMKHIDAAMERLKSKTVLDEKDLSLVERILANEPDPKTAYILALDLILVGIDTISMAVCSILYQLATRPEQQEKMYQELKRVMPDPNTPLNTKLLDQMDFLKAFVKEVLRVYSTVIGNGRTLQQDTIIHGYRIPKGVQVVFPTLITGSMEEFVSQSNEFIPERWMKKSSSDYKIHPFASLPYGYGARMCLGRRFADLEIQVLLAKLIRSYKLEYHHEPLEYKVTFMYAPDGPLRFKVIKREE

>Tc CYP302A1

MCLFSKKSVHYLLTIRTRNYYYRGRFLTTAVDPPKSFKEIPGPLSLPLVGTLYLYFPFIGRYQFDRLHKNALKNFQLYGPIIREEIVPGEHIVWLGDPDDIAKMFHTEGTYPYRKSHLTLEKYRLDRPHIYNSGGLLPTNGPEWSRIRKVFQKGLSGPTEALSFIKGSDDVISEWLDTRFKKIHKETSNMDFLQELSRLFLELIGVAAFDIRFQSFHDDELDPCSKSTKLLESAFVTNSTILKTDNGPQLWRKFETPAYRRLRKAQELMESVAIDLVALKLSTFKEKTSNPPTLLERYLASASLDFKDIIGVVCDFLLAGMDTTTYSSSFLLYHLATNPSTQDALYEEACRLLPNPAAPLTTEKYKQAEYAKCAVKESLRLRPISIGVGRQLTTDVVFSGYKVPSGTVVVTLNQVLSRMEKYFPEPDSFKPERWMKNDPSYVQTHPYLVIPFGHGQRSCIARRFAEQNMVILILKLARKYKLRWNGSEIDSKSLLINKPDGPILLSFEPR

>Tc CYP314A1

MFEKIFQSLDVTSLLIIAIFFLFLEYRPPWWYRNNDCKKGVKLIPGPLALPGLGTTWIFFFGGFSFNRLHLYYENMYKRYGPVMKEEYWCNIPVINLFEKREIVKVLKAGGKYPLRPPVEAVAHYRRSRPDRYASIGLVNEQGEAWYDLRSTLTPALTSPKTITSFLPEAQQIADDWCNLLKLSRDKNGRVSNLNYIADRLGLELTCALVLGRRMGFLLPGAETETGEKLAEAVRQHFLGTRDTYFGFPFWKLFPTPAYKTLIKSEASIYELALELINSANESTKESAVFQSVIQAEIDEREKIAAIIDFISAGIHTMKNSLLFLLHLIGQDLQIQKKIIEDSTKSYSKACVTETFRLLPTANALGRILEEEMELGGYRLSAGTVVVCHFGIACRDERNFPDASKFKPERWLDDDKVQTATNSLFLLTPFGAGRRICPGKRFIEHILPLLLESTVNSFEIQAEEELELQFEFLVTPKGQMPMVFKDRV

>Tc CYP315A1

MLFRRVLAQLSTRPKSSLVSANTVLHFDEIPSPKGLPLVGTTLALIAHGSTPKLHDYVDKRHKQLGPIFKEKLGPVSAVFVADPDEIRAVFDHEGKHPLHVLPDAWVAYNQMYGCPRGLFFMDGANWWYHRRIMNRLLLKGDFRWIEGACECVSDKLIDSLMGESDYCGNLEATLYKWSLDVIVSILLGSGSYSQLCGELEPKVQKLAQVTHLVFQTSAKLALIPASFASKFKIPQWRRFVDSVDNALAQANALVDTLIEKKPHSDGLLPKLLAEQITLEDIKRIIVDLVLAAGDTTAVAMEWMLYLVAKSPQIQEKLRRNPDFVKHVFKETLRLYPVAPFLTRILPEDAILGGYGVPKGTLVVMSIYTSGRDGRYFKNPGLFQPERWDRKDEFYSSEMKKASLPFAMGLRACVGRKVAETQLQMTLLKIVNKFEVELGNQREVEIVLKMVAVPSEPLLLKFKKL

>Tc CYP12C1

MSIIASWLRYNKFHTQLINIKYSPVNKSFKNLPTLKSYPVIGHSYLFFPRGKYKSERLTEAFVDISKTLGPIFKLNLGGSAMVVTLDPDHTRILFQNEGTRPERPPFPALLHFRRKRFSSVGVVPGNGEEWYKMRKGVTPLLKLQLIEPYKRQQEDIAKTFVEYVKTHRDENFVLRDIFSHLLKFTIEGHRFHCLFSDNLETEQIIKASVDFMDGLYGTLIEPPFWKLWKTPSYKKLESSHNTIYKILERHLEQIKFQFSENPESVKESQPYMYSLFSNDQLSWDDKIMLAMEIFLGGIDATATTISFTLHYLSQNPEIQKMARSQNTDFLKACIRETLRLSPTAGGNSRFLSNNTVIGGYLIPKGTLLLSLNSGMARDERYFKDAQKYRPQRFVRATREDFHRYASLPFGHGPRMCPGKRVAENEIILKNFALESAGSSDVGMVFRMNRIPDKPISVRFVDTNH

>Dm CYP12A4

MLKVRSALSLIQSQKATLSLATQKRWQTNVATAEAREDSEWLQAKPFEQIPRLNMWALSMKMSMPGGKYKNMELMEMFEAMRQDYGDIFFMPGIMGNPPFLSTHNPQDFEVVFRNEGVWPNRPGNYTLLYHREEYRKDFYQGVMGVIPTQGKPWGDFRTVVNPVLMQPKNVRLYYKKMSQVNQEFVQRILELRDPDTLEAPDDFIDTINRWTLESVSVVALDKQLGLLKNSNKESEALKLFHYLDEFFIVSIDLEMKPSPWRYIKTPKLKRLMRALDGIQEVTLAYVDEAIERLDKEAKEGVVRPENEQSVLEKLLKVDRKVATVMAMDMLMAGVDTTSSTFTALLLCLAKNPEKQARLREEVMKVLPNKNSEFTEASMKNVPYLRACIKESQRLHPLIVGNARVLARDAVLSGYRVPAGTYVNIVPLNALTRDEYFPQASEFLPERWLRSPKDSESKCPANELKSTNPFVFLPFGFGPRMCVGKRIVEMELELGTARLIRNFNVEFNYPTENAFRSALINLPNIPLKFKFIDLPN

>Dm CYP12B2

MWKYSNKIIYRNVSGNQLWFNRNSSVGGTLSQQVRSWQKEQELLKSRNLFTNNGYICSQTQLELADSRIDEKWQQARSFGEIPGPSLLRMLSFFMPGGALRNTNLIQMNRLMREMYGDIYCIPGMMGKPNAVFTYNPDDFEMTYRNEGVWPIRIGLESLNYYRKIHRPDVFKGVGGLASDQGQEWADIRNKVNPVLMKVQNVRQNLPQLDQISKEFIDKLETQRNPETHTLTTDFHNQLKMWAFESISFVALNTRMGLLSDNPDPNADRLAKHMRDFFNYSFQFDVQPSIWTFYKTAGFKKFLKTYDNITDITSNYIETAMRGFGKNDDGKTKCVLEQLLEHNKKVAVTMVMDMLMAGIDTTSSACLTILYHLARNPSKQEKLRRELLRILPTTKDSLTDQNTKNMPYLRACIKEGLRITSITPGNFRITPKDLVLSGYQVPRGTGVLMGVLELSNDDKYFAQSSEFIPERWLKSDLAPDIQACPAARTRNPFVYLPFGFGPRTCIGKRIAELEIETLLVRLLRSYKVSWLPETPIEYESTIILSPCGDIRFKLEPVGDLM

>Dm CYP301A1

MNNLSLKAWRSTVSCGPNLRQCVPRISGAGSRRAQCRESSTGVATCPHLADSEEASAPRIHSTSEWQNALPYNQIPGPKPIPILGNTWRLMPIIGQYTISDVAKISSLLHDRYGRIVRFGGLIGRPDLLFIYDADEIEKCYRSEGPTPFRPSMPSLVKYKSVVRKDFFGDLGGVVGVHGEPWREFRSRVQKPVLQLSTIRRYLQPLEVITEDFLVRCENLLDENQELPEDFDNEIHKWSLECIGRVALDTRLGCLESNLKPDSEPQQIIDAAKYALRNVATLELKAPYWRYFPTPLWTRYVKNMNFFVGVCMKYIQSATERLKTQDPSLRAGEPSLVEKVILSQKDEKIATIMALDLILVGIDTISMAVCSMLYQLATRPVDQQKVHEELKRLLPDPNTPLTIPLLDQMHHLKGFIKEVFRMYSTVIGNGRTLMEDSVICGYQVPKGVQAVFPTIVTGNMEEYVTDAATFRPERWLKPQHGGTPGKLHPFASLPYGYGARMCLGRRFADLEMQILLAKLLRNYKLEYNHKPLDYAVTFMYAPDGPLRFKMTRV

>Dm CYP302A1

MLTKLLKISCTSRQCTFAKPYQAIPGPRGPFGMGNLYNYLPGIGSYSWLRLHQAGQDKYEKYGAIVRETIVPGQDIVWLYDPKDIALLLNERDCPQRRSHLALAQYRKSRPDVYKTTGLLPTNGPEWWRIRAQVQKELSAPKSVRNFVRQVDGVTKEFIRFLQESRNGGAIDMLPKLTRLNLELTCLLTFGARLQSFTAQEQDPRSRSTRLMDAAETTNSCILPTDQGLQLWRFLETPSFRKLSQAQSYMESVALELVEENVRNGSVGSSLISAYVKNPELDRSDVVGTAADLLLAGIDTTSYASAFLLYHIARNPEVQQKLHEEARRVLPSAKDELSMDALRTDITYTRAVLKESLRLNPIAVGVGRILNQDAIFSGYFVPKGTTVVTQNMVACRLEQHFQDPLRFQPDRWLQHRSALNPYLVLPFGHGMRACIARRLAEQNMHILLLRLLREYELIWSGSDDEMGVKTLLINKPDAPVLIDLRLRRE

>Dm CYP314A1

MAVILLLALALVLGCYCALHRHKLADIYLRPLLKNTLLEDFYHAELIQPEAPKRRRRGIWDIPGPKRIPFLGTKWIFLLFFRRYKMTKLHEVYADLNRQYGDIVLEVMPSNVPIVHLYNRDDLEKVLKYPSKYPFRPPTEIIVMYRQSRPDRYASVGIVNEQGPMWQRLRSSLTSSITSPRVLQNFLPALNAVCDDFIELLRARRDPDTLVVPNFEELANLMGLEAVCTLMLGRRMGFLAIDTKQPQKISQLAAAVKQLFISQRDSYYGLGLWKYFPTKTYRDFARAEDLIYDVISEIIDHELEELKKSAACEDDEAAGLRSIFLNILELKDLDIRDKKSAIIDFIAAGIETLANTLLFVLSSVTGDPGAMPRILSEFCEYRDTNILQDALTNATYTKACIQESYRLRPTAFCLARILEEDMELSGYSLNAGTVVLCQNMIACHKDSNFQGAKQFTPERWIDPATENFTVNVDNASIVVPFGVGRRSCPGKRFVEMEVVLLLAKMVLAFDVSFVKPLETEFEFLLAPKTPLSLRLSDRVF

>Dm CYP315A1

MTEKRERPGPLRWLRHLLDQLLVRILSLSLFRSRCDPPPLQRFPATELPPAVAAKYVPIPRVKGLPVVGTLVDLIAAGGATHLHKYIDARHKQYGPIFRERLGGTQDAVFVSSANLMRGVFQHEGQYPQHPLPDAWTLYNQQHACQRGLFFMEGAEWLHNRRILNRLLLNGNLNWMDVHIESCTRRMVDQWKRRTAEAAAIPLAESGEIRSYELPLLEQQLYRWSIEVLCCIMFGTSVLTCPKIQSSLDYFTQIVHKVFEHSSRLMTFPPRLAQILRLPIWRDFEANVDEVLREGAAIIDHCIRVQEDQRRPHDEALYHRLQAADVPGDMIKRIFVDLVIAAGDTTAFSSQWALFALSKEPRLQQRLAKERATNDSRLMHGLIKESLRLYPVAPFIGRYLPQDAQLGGHFIEKDTMVLLSLYTAGRDPSHFEQPERVLPERWCIGETEQVHKSHGSLPFAIGQRSCIGRRVALKQLHSLLGRCAAQFEMSCLNEMPVDSVLRMVTVPDRTLRLALRPRTE

>Ld CYP301A1

FGELAGVVGVHGEAWRKFRTKVQKPILQLRTVKKYIEPIEVVTEDFIKRMLEMKDQNDEMPGDFDNEIHKWALECIGRVSLDVRLGCLDPNLSPDSEPQKIIDAAKYALRNIALLELRFPFWRYFPTSLWTRYVKNMDYFIEICTKYIDEAMKRLNTKSVKDDKDLSLVERILANEPDPKTASILALDLILVGIDTLSMAVCSILYQLATRPDEQEKLYQELKRISPDPAEPLNSRKMEEMLYLKAFVKEVFRVYSTAIGNGRTLQEDMVLMGYQVPKGVQVVFPTVVTGSMEEYVTEADQFRPERWLKQESGNFKFHPFSSLP

>Ld CYP302A1

MNYSYFRFSHLDKYLQKATWISFSNLYRYSHNFSNIPGPISLPVVGTLYHYVPIIGDYKFDELHHNGFKKYYKFGPAVHEKHVPDVNIVWLFDPVDIETMFRNEGKYPQRRSHLALEKYRLDKPAVYNSGGLLPTNGSEWFRIRSIFQRGLSSPQSVKKFLGDTDEVIREWIVSLEEISSKPGLDYLPELSRLFLELTGKAILDIRLNSFAKHERSKHSRSSKLIEAALVTNSSVLKTDNGPQLWKKFDTPLYKKLKRAQGYMEEVAIDLLSLKMSLFSEREKNTPSTLLETYLSSPELDFKDIIGVICDFLLAGIDTTTYTTSFLLYHLANNQHTQDNLFREAHKLLPQKDTPVTEKILQEAVYSKAVLKESLRLRPVSVGVGRVLQSDAEFSGYSVPSGTVVVSQNQISCRLHKYFPSPDEFIPERWLKKDPLYKQPNPFLVLPFGHGPRSCIGRRLAEQNMLVLVLKIMRNFHVEWLGGNLDSKSLLINKPNGPILLKFRKRLC

>Ld CYP314A1

MGYIIIDMFKEILQSLSTVNYVLVLVFFLIVGYRPPWWSRKICNFLPKTVSDIPGPLSLPFFGTSWLFLSRIYTYDKFHEFAEDIYARYGPIVKEEALFSVPVIHIFEKSDIEKVLKSNTKYPIRPPTEAVAVYRKTRPDRYASVGLVNEQGIKWHELRTSLTKKLTSPKTVAGFLPQVQEIVEDWCNLISQQRTDNTITNLEDIVGPLGLEISCALVLGRRMGFLLPGAESETAQKLAETIHQLFIATRDTYYGLPFWKIFETPAYKKLAESEDTIYQLVTNLISTADQAANRSPVFQSVLDANINQKEKTAAIVDFIAAGIHTLKNSLVFLLYLVAKNPGTQEKILEDSSKAYLRACIMETFRIYPTANCLARITEEELELSGYKVKPGSVILCQTGIACKNDRYFADANQFRPERWLTEEKSLTSTNATYLVIPFGAGRRICPGKRFIEQVLPVILENTLKKFIIDVEVTRPIELQFEFLLSPKGPTSLIFEDRIGSEEDSTASE

>Ld CYP315A1

MLSRQVRPFFCKFRVRCASSSGALEFEDIPSPKRLPLIGTTLSLLAAGGAKNLHKYVDRRHKELGLIFKDNVGPVSCVFLADPEGMRHVFSKEGKYPVHIKPESWLLYNEKHGYTRGLFFMDGEEWLKFRRIMNSLLLKGSLSWLEESCDAAGEVLVKKLEGFKNIEVPNLEQEMYKWSMDVIVSVLVGKRTYASHQEELDVLVYELAGKVSKIFETTSKLQLISAKLAQKYQLGYWKRFENSVQEGLDSAYSIVNHITANYKDADGLMAKMLSENIKSDDLNRIIVDLILGAGDTTAYTMIWILYVLSKNPNVQKELRNQLLQNKKTPFLKNVVKETLRLYPVAPFITRFLPESVTLCNYRIPANTLVVMSIYTSGRNEKYFIEPELFQPNRWLRNNHEMNMALQQASLPFGIGSRSCIGRKIAEHQLHGTLAKIVDKFQVEMCNREEVHDILQMIIKPSQNMRFKFNRIQD

>Ld CYP49A1

MKGFSKCARLMSVSSAQAQDVISSADKLVEAGRARPYQSIPGPKPLPFLGNTWRFLPLIGDYEIEHIDKVSEKLYKEYGKIVKLEGLMGRPDMLFVFDPDNIEQVFRQEDILPHRPSMPSLNYYKHVLRKDFFGEYGGVIAVHGEKWQSFRSKVNQIMLQPRVAKMYVSSISSTAQEFVDRCYMKIPSKLIGFLFVSRICMKYINKSLKRNTEDVLENNEISILQRILRLENDPKIATILALDMFLVGIDTTSNAVASILYQLAVHPKKQEKLRKEVFRVLPNISSAVTHEKLEEMVYLKACIKETMRMYPVVIGNGRQTSCDTVIAGYHIPKGVQVIFQHYVISNLEQYFPRSKEFLPERWLKSCPFSKEQHPFASLPFGFGKRMCLGRRFADLEMQTALAKYKVEYHHEKLDYFVHPMYTPNGPLKLKFIKHGS

>Ld CYP12A5

MLIRCASRPLALGARAVSASSSKPCAGAAAVSKREDYYKTLGEHVDESKPPGWDSARPFEEIPGPKPLPIVGNLLRFMPFVGELYKTPILGMFQKFREQYGDVVILKGIPGRSDYVYIYDTKDIENLLRNSGTFPVRRVLDVFVYYRTVARKDIFQGIGGVLTVQGEDWFKIRTVVNPILMQPKTVQQYMSKLDGVAGELAENMRHFSRLNETHQMPDDFVNELYKWALESVGIMALNKHFGCLDLTTPQDSDPKKLVTAVLNMFKHLYLIETSPPLWKFISTPLYKKFVKNLDFITSIIVKTIDEALAEPIDESVPEHQLSVLHRLGRKDKRIAFTMVMDMLIAGLDTTGKTLGAILYFLAKNPDKQAKLREEIFEVLPEKDSVVTAEGLGRMPYLKAVIRESNRIAPVAVGTVRTTVKELVLGGYQIPKGTNVTVVSICTSNSSEHFKDPEKYMPERWLRDTNDEYSSKNVHSFASLPFGFGPRMCVGMRFASLELELVLVKIIRNFELSWEHPDMEFASHLLYGINNPLKLTVKELTR

>Ap CYP12A5

MLYVVISLLLALYCIFCIYDCVKPHNFPPGPKWLPLIGCFLTFRRLKLKHKYTYVAFQELSKTYGPILGLKLGSQKLVVISTHDLVKKVLLQDEFNGRPDGFFFRVRAFGKRKGILFTEGSMWSQCRRFTMRHLRSFGLGQSTMEKYLTVEAENLVNYLRRVSTKGPVPMHTAFDIAVLNSLWCMFAGHRFDYENEKLAEILEIVHDSFRLMDTMGGIISQMPFLRFIIPELSGYNNLMEILRKLWNFLDEEINNHEKHLSGNQPQDLIEAFLLEISSRNGVQNDSIFDRENLLILCLDLFLAGSKTTTDTLSTSILFLSLHSEWIKILQEELDNVVGRSRSPTLEDYSSLPIMESFLAEIQRFLILAPLGVPHKTTKDVILNGYNIPKDTTVLLDFHSAHNDPAYWDHPEEFRPQRFLDANGRFCQNNANIPFGLGKRRCPGEMLARTSLFLYFAYVIHYFDIEISPEHGKPDLNGHDGFTISPKSYYLKITARSDVTNCSTI

>Ap CYP315A1

MKESGNLQRRGKRLLDNGIVNVLSLDWIQVIKHDSRKRKQEEMPEPRGIPVFGTLFSFILSGGPKKQHEYVDKRHKELGPVYKERIGPTTAVFVNSIHEFRKIFRLEGSTPKHFLPEAWTLYNEIRKCRRGLLFMNGEEWVYFRKILNKVMLLPDPTNLMIAPCQEVAIELKRKWQKQIKTNNIISNLQVQLYQWSIEAMMATLMGSYWYSYKHQLSRDFEILAETLHEIFEYSAKLSIIPVKLAMNLRLPVWKKFVASADTAFEIVRMLVPEMAKLGGNGLLKKMMDEGIRAEDAICIVTDFILAAGDTTATTLQWILLLLCNHPEKQEELFKHLKDLSQEDILRLPLLKGIIKESLRLYPIAPFISRYLPEDSVIGNYFVPKGELLVLSLYSSGRDAANFPQPNEFRPERWIRTQKGIYQGVVHPHASLPFALGARSCIGRKLAEIQISFALAELIKSFKIECINKNQVKLILHLISVPSQSIKLKLMERN

>Ap CYP302A1

MCTLLKKCNQSIRKKLFIKFYSNEFTKSKIKINHSQPKAFYDIPGPKSLPIIGTLYKYLPFIGEYSFTNLYESGKKKLKCFGPLVREEIIPNVNVIWIYRPEDIAEIFKAESGLHPERRSHLALLKYRKDRPNIYNTGGLLPTNGSEWWRLRKEFQKVSSKPQDVINYLKETDCVIQEFVELCNNEKFADFLPLLSRLFLELTCLVVFDIRLNSFSKEERCENSISSKLIKAAFATNSAILKLDNGLQLWRLFETPLYRKLRKAQTYMEMIALELVSRKKNNMKIRYNKSFLDAYLENPVLDIKDIVGMACDMLLAGIDTTSYSTAYILYHLAKNQNIQEKLRIEATQLLKNHNEPISINILRNASYTKAVIKESLRLNPISIGIGRILQTDVVLSGYRVPKGSVVVTQNQIICRLPEYFEEPNLFIPERWLREYSENNNKINYKKTVHPYVLLPFGHGPRSCIARRFAEQNMQILLLRICRRLKISWHGDDLGMISLLINKPNALLKFNFHDILNNNSV

>Ap CYP314A1

MLLSSAWFEVIAAVLLTILIFVTSHRPAWWFWTATSHEASAPAEGKFKTVSKVPGPFSLPIFGTRWIFSCIGYYKLNKIHDAYKDLNQRYGALCKEEALWNFPMISVFSRQDIETIIRRNSRYPLRPPQEVISHYRRTRRDRYTNLGLVNEQGQTWHDLRVALTSELTAASTVLGFFPALNIVADSFIELIRRQRVGYKVTGFEELAYKMGLESTCTLILGRHLGFLKPDSSSELATRLAEAVRIHFTASRDAFYGLPLWKLLPTCAYKQLIESEDAIYNIISEIIETTIQEKRDDAKDESVEAIFQSILRQKNLDIRDKKAAIVDFIAAGIHTLGNTLVFLFDLIGRNPTVQNKLYEETYALAPAGCDLTIDNLRKAKYLRACITESLRLIPTTTCIARILDEPIELSGYRLTAGTVVLLHTWIAGLNEENFKDAKKYLPERWTTPTTPHSPLLVAPFGAGRRICPGKRFVDLALQLILAKIIREFEIIVEEELDLQFEFILAPKGPVSLGFRDRS

>Ap CYP12A2

MMQPKVVTQYINIIDSIANDFMEMIQEYVDKNPQMPDNFLELYNKWALESIAAIGLDTRLGCLEKNLSPDSAQMKYINSVLETFDLFYKLDILPSLWKYYSTPNFKRLVKLLDFITFTNVNHIEEALKRIESNPNKLESEMSILEKLAKKDKAVALAMVTDMLLAGVDTTSRTLAATTYHLAKHPDKQAILREESIKLLPTKDTPVTKDVLNNASYLRACIKESMRIAPIAIGTARKLAKDVVIAGYQVPKGTTVVISNIYVTNSEEYVKQPKEYIPERWLRNIKSDLAFKNRFMYMPFGFGVRSCIGQRFANLELETGLLKMIRNFEISWPYKEDMPFKSKLLYGIDAPLRLYIRKIEN

>Ah CYP6A8

MAFTFSLVLEFLLVICLILATAFLYIKYKYNYWKRKGVPQLIKPTFPFGNYLKTLPEGINIGAHSKKFYDVYKKAGYTLGGVYLGLDPHLVIVDPLYAKDVLANDFQYFTDRRIYSNKKVPITLSLLSQQGDEWKITRAKFTSIFTASKMKYFFNTVDRCHREMIENFNYLEEKGSDINIFEMMACYTTDVISSVIFGVEANSFKMPNAIFRKLGRELFSKFSISIRLQLFLTICYPKLAKLLDVSNIQSYINDFFKSFVKDALEYRQSNNIVRQDFLQLLIELKNSEVKLSIDEIASQSFLFFSAGFETSSASSSFALLELGLNQDIQNKVRTEINDVIKKYDGKITYESLSEMHYLGQILQETMRKYPLLTALTRQCVKDYTFKNSDVTVKAGTGIILPVLGYHRDPEIFTNPEVFDPDRFADKDVKYEGYLPFGNGPRICIGARFGTMQVTLALAAIVKEFQIFVCPKTRSELPIELSQNTFVTRPIQTIYLKFKKIV

>Ah CYP6A9

FKVVGKAIFDASSLGHRLRMNIVNYFPDLALLFSVTNMSSSVLDFFKQFLKATMKYREKNNIFRKDLVQTLMKLKQESNIGEDELAAQAPS

>Ah CYP6BQ7

QWISTYWKRRGIYVPKCSFLFGHLNDLITFKKSVADLYVDIYRDIKKKGHRYGGIFFCKSPQFIPVDLELIKSIMQTDFKHFHSRGLYHEEATDPLAANLIFTDGLKWKDSRKKFTPTFSPGKLKTMCPIIVDLSHRFSDLLEQSAKSGPVDVKAIINRFTVEVIGCLIFGLDMNTIKNENSDFVKYAMVPFEPSLGGLMRKMIQFVLPNNILKMINYSHVPKDCTEFYFNTIGSTITFREKNNFVRNDFMNLLIDLKSNGIITTKEIVAQCYIFLVAGFDSSASIVAFAAYELADNQDMQTKLRNEITSVLKDHDNQITFEAIMEMEYLDKVLKGNL

>Ah CYP6BQ8

MLTVLLYLLIGLPLLFQLYKKWRLNYWQRKGVYCVEPEYPYGNYKDVRQGKISEFDFLKKYFYDLKKKGLKYGGLYQEMKPLFIPVEQDFIKQLLLKDFQYFTDHGFFSNPKVDPLSGSLFQLDGEQWRNHRTKLTPTFTSGKMKMMFPTIVEKTKGLEKLLAKESNTGEPVMIKEAVARFTTDIIGAIAFGLDCNCMEVPNNEFRTYCNKALGSTSKWVVFQMKYFPHWLLRAIRFKRGDPETEAFFFNLVESTIKYREENGIVKKDFMQLLIELNNTGSVTNDETLKKNIKKSSDSPVFTLDELTGHALSFFVAGFETSSTLMSFALFELGQRPEIQETLRKEINEVLAKHNGQLTYEALSQMTYLDGVIMEALRKHPPFPVLYRICTKSYQIPDSNITIDKGTHVRFILHAIHNDPELYSEPEAFMPERWNDGSIDKSKYQFLTFGEGPRMCIGMRLGKIQAKCGLLTIIKNYKVTLSNKTMLPLKYVPFVVTGVQGGVWLNMEKIQK*

>Ah CYP6BQ9

MLIILIVICASVYCYIKRKQSYWQRKNVDYIEPSFFVGNTSANTTGKIHKGELYQQFYEHFKSNGKTFGGVYSNLTPVLVPTEPALIKAILQTDFAHFMNHEGFVNEEDDPLSGNLFSLKDEKWRNIRSKLTPTFTTGKMRMMFDTMVECTVGLQKLLDPYAEMHDPVDIKKYLLLFTTDIISSVAFGLDVKSLENPNTEFRTRFNKIFEPNLRMIIKQIIIQSVPHWLLKKLRFSLSNREMLKFFVKVVKDIVEYREKNNVYRKDFMHLLIQIKNFGKTTDDEMLKGNSEGTHLTLNQMTAQCITFFIAGFETSSTTMSFALLELAQKMEIQEKIRDEIETVLKRHDGKITYDAIMEMEYLEQALDEALRKHSPATTLSRVCNKEYKIPGTDTIIDPDVHVMVPVYALHRDPKYFPNPEKYDPDRFSKENKQNIVPYTYLPFGEGPRSCIGLRFGKLQSKVGLCSIIRTYKITLNEKTKVPVKYAQARIPTVQGGIWLNLEKIN*

>Ah CYP6BQ10

MFLYSLIVLILASYLYIKWKYTYWKRKGVLYAEPTFLLGFMDTLVKRHHMITPMAKLYDQAKAEKRKYCGHYEFLTPVFTPIDLELIKTIMQTGFGHFDRHWDPRADENADPLSRHLFNLGGEKWKMIRSKMAPTFTSGKIKMMFETMVSCTQVLEDILRQSPKTKPIEITDVLSRFTMDIIGSVGFGLDINSMKNPDTDFQKYGRKSFKFPVSKVFRLILINMLPLWLVHKLKIPSTEPDITSFFKNLVKTTVSYREKNNIFRKDFLHMLIQLKNFGRLLDEEKVVPDKTLEGVTPSLTLNEITAQVFVFFAAGFDTSAITMSFALFELAQHKDIQNKARNEVRRIFGKYNGKITYDGIIEMEYLDQIVMETLRKYPPVGVVPRECTKDFHVPDSDLVIYKGQRVQIPAYSIQRDSEYFPNPELFDPERFNNENKATIPPMAYIPFGEGPRICIGQRFGKLQVKVGLCCVLKDHEVSLNDKTKIPLQYESAFILSVAGGIWLNLKEV*

>Ah CYP6BQ11

IVRQDFLHLLIQLKNTGCVEVSDDVTLPIKQNKEEGMSIEEVSAQAMVFFLAGFETSSTTMTFALLELSQNQDIQNKLRKEIRNVLKSHNDQITYDGIMEMQYLEMVISETLRKHPPLAVLPRGCIADYKVPGSDLIIKAGTKIDIPVLSLHMNPKYFGNPEKFDPERFSPENKNNILNYTYMPFGEGPRTCIGLRFGKLQAKVGLCTILKDYKVTLNKKTNYPIQYDPRAQVTTVKGGVWINIEKIQN*

>Ah CYP6BQ15

MLLPVTGLFFIILISVYVYSKWKYLYWKTRNVFHLEPEFFYGNIRPMLRRIKPMSDVVNEVYVEAKAKGNKYCGLYFFFTPQFVPMDLELLKCILQKDFSYFTNHGFYVNEEKDPLTGNLFNLEDDKWRNLRAKLTPTFTSGKMKMMFHTMVACTNGLKEALKESAAINEPADIKDILCRFTTDIIASVAFGLETNSLKNPDNEFRKYGKQILAHTFRGQLISSLINLLPKSVMKLLPIKIIRQDMENFFINVVKKTVLYREKNNIYRKDFMHLLIQLKNIGKVLEGTEESALLFKTGNSSCVRGMTIEEMAAQVFVFFLAGFDTSATTVAFALLELSENQELQDRAREEVVRVLQKHNNQMTYEAIKEMEYIEKVICETLRKHPPLPIIPRKCNKDYKVPNSDLVIKAGTRVPIPVWSIHRDPQYYKDPEKFDPERFNDENKASRPEFSYLPFGEGPRTCIGLRFGKLQAKVGLCSVLQSYRVIFNDKTAKPIKYKNSVIAEVKGGVWLNLEAIE*

>Ah CYP6BQ16

AAVILLLFFYVKYCQTYWARRNIYTVKPTFLLGSIDTIFKNQHMVVPLSEGYDAAKAKGERYCGLYEFLRPAFVPIDLELIKLILQNNFANFESHFDVIVDEKDDPLIGHLFNLSGEKWRSLRAKLTPTFTSGKMKMMFETMTSCTDGLEEMLNSYAKTNELVDIKDILSCFTTDIIGSVAFGLEINSMKNPDNEFRKHGKSIFKIRFPMICRMVLINMLPRWLVHKLKIAWIDNDVTSFFINLVEKTVAYREKNNIYRKDFMHLL

>Ah CYP6BQ34

MLCITIFLVILGTLYVYSKNKFSYWKTKNVFYKEPVFFFGNITDVITGRRDIGQVMKEIYITANEKGLRYFGIYKLWEPVLVVFDLELIKRILQTDFSHFISHGFFIETKKPGIHSNLFIVEGEYWRKLRNKMTPAFTSGKMKMMFQTMVGCTDGLKDLLQDESKSDEGMEIKEVLSRFTTDIIGSVVYGVEINSMKNPNTDFKKIADVVFKPSLRNTVQGIIFAVIPKKLNLLFRYLLKTGVTDFFFKLLKETIDYREQNNIVRQDFLHLLIQLKNTGCVEVSDDV

>Ah CYP6BQ36

SGKMKMMFSTMVACTQGLEDILRESARIQDVVEIKEVMSRFTTDVIGSVAFGIDCNSLKNPDSEFRQFGKKMFDFSLTRFFKSTMSRITPKPLLNVLGYKQTPQDIEDFFMGVVQSTVNYREKNRIYRKDFMHLLLQLKNRGKVVDDENIFDDKDVTKGEYFLTFNEVAAQCFVFFAAGFETSATTMTFALLELALNQDIQENLRKEIKSVLEKYDDKVTYEAVMNMRYLDKVVHETLRKHPPFGALPRKCNTSYLVPNTNVIIEAGTIVQIPVQAIHRDPDYYPNPEKFDPERFNEENIAARHPCAFLPFGEGPRICIGSRFGMLQAKVGLISIIKNFQVSLNEKTKTPIEYITKGFVTGVKGGVWLDITER*

>Ah CYP6BS1

MGFLTISLLSILILLYIYYKNAFRYWKNINVPQLDPSFPFGDMYETIFRKQNMGDKIKEIYDKMKGHRYVGLYFFSRKALLPLDPILIKKILNSDFHHFYDRGIYYDEINDPLSAHLFSISGTKWKNLRAKLTPAYSPGKLKYMFGTILKCGEEMTNKLEEISKADGEIEIKEILARYTTDVIGTCAFGLECHCMTNPTAEFRVMGKRAFTQTIGDVLRMIIIRSFPPLARLLRFGVFTGTVTSFFNKVVRETIAYREQNSITRPDFLQLLIQLRKNGKLEEDNLDDHPPGTCLTIEEAAAQAFIFFLAGFETTSTTISFALFELASNQSIQNKARKEVDKVFEKYQSFTYESVMEMHYLDTVIYETMRKYPPAPVFLRKCTEAYQIPDTNIILEKGLSVLIPCYGLHRDPEYFPEPELFDPDRFSEENKSNIWDGTYIPFGDGPRNCIGMRFAMIQAKIALSLTLKNFHFDLSKKTRLPLKMETKGIILSPIGGLWLNFCPR*

>Ah CYP9AC1

LFVLKGQKWRYMRPILSPSFTSSKMRTIFELISECTENFIGHFQEKNDDIIEVELKETFTRFTTDVIATTAFGLKTNSLKDPTNDFFVMGQMLSKFASGFRNLWKFMVLALSPTLFKILNLSIFDKKVMSFFTDIIDQTIKVRKEQGIIRPDMIHLLIEAQKSIAKEEQVEDLSFAAHTESDLGQQNVAEKKELTNMDIAAQALIFFFCRF

>Ah CYP9E2

GQRWKYMRSILSPAFTSNKMRSIYNLMAETSDAMVKYFLEKKQDHIDVAFKDLAARFANDIIATTAFGVKVDSLNNPENEFFYMGKQLTGGNRSAWKILKSVLVLLCRPLAKVLKVKIFDEPGVSFFVNLLRDTIKIRKERNIVRPDMLQLLLEAQKDANSNSKDHSGSPQKNIVSEVDILAQALVFFFAGFESVSSLIAFMAYEIALNQDVQEQLREEIRNAHEKCNGKLIYEVLFQMKYLDMVVSETLRKWTNFSTTDRICTSDFTIPAELPHEKDFVIKKGTEITIPIRAIQYDDKYYPNPQKFDPERFNTENKKNINASTYLPFGLGPRTCIGNRFALMEAKVFFSYLLMKFEIVPNAKTVIPIVLSPPKFNVHPADKLNLTLKPLHY*

>Ah CYP9F2

MLSAVLFGCVILLLSFLFYKYFVKPFSYWSDRGVKQTNPKWVFGDNWKIITKQECMFDTLKRLYRELPNNRYVGMYQFTQPILMIKDVELIKQITVKEFEHFVDHRPWVPETSDYYWSQNLFALRGDKWKEMRTFLTPAFTGRKMRAMYGLMRETAQRFVQHFEEKIEGTLELDFKDICNKFSCDVIATTAFGVEVDSLKYPHNEFYVYGKEAGNFTGIKMLIFFVGYFLPSFLMQLLDIKVFSKKVSDFFSSLVEDTITTREKNNIYRPDMINLMMEAK

>Ah CYP9Z1

MLLRIYKDAGNERYYGFYQFSTPTLLIKDPELIKQIGVKDFEHFLDHSTFIPENADPLWGKNLFALKGQRWRDMRPILSPSFTSSKMKVMFQLMHDCANNFTDFFLKKNENVIEVKLKDIFTRYTNDVIGTTAFGIKTNSLENPDNEFYRMGKEATDLTSLRKNLKFFGYLIFPRLLEFLKVKLFESHVAAFFTNLIDNAIKVRREKGIIRPDMIHLLMEAQKLGANSKEEHNVDNGFATVKESDLGKPNIHHRALTNLDITAQALIFFFAGFDSVSSVMGFMAYELAVNPDIQNRLREEILETAEECKEELTYDVILKMKYLDMVLSETLRKWPNAIAVDRVCTKPYIIEPATPNEKPLYIKEGTLIWFPIYAIHHDPEYYSDPDKFDPERFSDENKGNIDPYKYIPFGIGPRNCIGNRFALLEIKVIMFHILSHFEIVPIANSQIPVIPSKKQFNLIPDDGFQFGLKHIGTKKVYSTVK*

>Ah CYP9Z4

MFWILILILLATLFYFYVFKPLTIFREIGVPQESLLKCIPTNYFAFIKGITLPDFMLEVYNKFPGSRYHGFYNLNRPVLVLKDLDLIKQMLVKDNEHFNEHKEFVPAEADPLFARNLFALKGNQWKNMRSALSPSFTGSKMKGIFLLMDEYSRNFINFFLNKDEDLIEIDMKDVSSRYCNDIIASTAFGTAVDSFINPDNEFFLSGKKLTDFRSFFFKVRLLICMAFPSVSKAFNIPLMDPTVTQFFENLIKDVIKYRRESGDTRQDMIQILLETQKGCVKTEEQTENIEFASVEEHIKTSDVPVITDDDIVSQAIIFFFAGFESSSTMMCFMAYELCVNQDIQEKLREEILETSARCGDKITYEHLTRMKYLDMVVTESLRKWPAQVGVERVCIKPYTIEPVLPNEQQAHLYKDLTVFLPIYGIHHDPNNFPDPSKFDPERFNDENKRLVKSLALQPFGVGPRNCIGSRFALLEAKMMFYYLLKHFKIVPVEKTVVPIQLGKNIFSILPYDGFWMGLEKC*

>Ah CYP9Z19

MWGILLLTAIAAVIYYFYDKVIYWKKKNVPQEDIINAVKTIVTFIMGKSVFADFVVDGYKLFPNARYTGIYQSTRPLLMIKDLDLIKDIAVKNFDHFTDHSNFLSDVSEPLWARNLFALKGQKWRDMRPILSPSFTSSKMKMIFHLISECAENFSNFFSNKEEEVIEVEFKDVFTRFTNDVIATTAFGVKTDSLKDRENDFYVMGNKLTKFTSVWLGIKLFIIVLAPKIADLFKLSFFDKEVTAFFTKTINSTIETRKEKGIVRPDMIHLLMQAQKEQEEHSINDKSFAAQEESELGKGGGQHVQLSNLDIAAQAFIFFFGGFDSVSTAMSFMAYELAINPAEQSRLREEILETLQNCNGHLTYEALLKMEYMDMVVSESLRKWPAQMATDRQCTKPYVIQPESPDEKPLQIEENSIVWIPIYAIHNDPKYYENPEKFDPERFSEENKGNIAPYTYLPFGLGPRNCIGSRFALLELKTVFFYLLLKFEIVPTKKSQIPIKYAPGFSVASDKGFWFALKKLK*

>Ah CYP9Z62

QGSVLYWLKDHISTIRGTKCFPDFLMEIYNMAPEARYFGLYQLTQPFLLIKDPQLLKEMVVKNFEHFTNRSTLIAESADEPLWAKNIFALQGQQWRQMRPLLSPSFTSARMREIFKLVSDCANNFVIHFQNKSRETTEVDLKDLFTRITTDVIATTAFGLEINSLKNPKNEFYMTAQNLGAFSTGVWSIWNIVMLNCFPKLCKLLNISLFPSEATSFFTDSILQTIKTRRKQGIIRLDMIQLLMEAQDDANAQTKQAFSKTNVNQKKELTDLDIAAQAFVFFLAGFDTVSTQMSFMAYELVVNTDVQNRLREEIKQTLEDCDGKLTYEALNKMRYMDMVLT

>Ah CYP9Z63

MILVCLSILIVFVLLYYHLVYSPLTLWRRAGVAQKSVLHNFINSWFLFVKGQSVVDLIKGFYDEFPGVRYSGTYQFNSPILVIRDPVLIKKIVIKDFEHFVDRRTYIPEDADPMFGKSLFSLRGDKWRNMRSTLSPAFTSSKMKSMFVLMKECSKNFVKFFSEKNQEITEVQFKDISTRFCADVIATVAYGISVNSLENPENEFYIRGKSSTNFGTFSKMIKFIGNLAAPKLYKLLDVKLADKDEREFFTNLIEETIRAREEGITRPDVIQLLVDTRKGLKIHEDHTTEDTGFATVQEDKRAKGIAIENLTNEDITAQAFIFFFAGFESVSTLVCIMAHELATNPDIQDKLRSEIIEVGGSDLTYDVLVGMKYMDMVVSETLRKWPAQAGFERICTKPYEIAPVHPNEKMVKLEKGAVVIFPTVGIHRDPEYFPDPDRFDPERFNDANKHNIVPCSYTPFGGGPRGCIASRMAILETKILFYYLLSSFELVPVEKTCIPLTMAKNALLISVEGGCWLGLKKISSS*

>Ah CYP345B1

MLLTTSIICDFVILLGTLFVALYLFFARHANYWKKRGVPFVKPVPFFGTMLDVFTSKKVIGLLFQDLYKEMKTPYLGIFIMDRPGLLTSDLNLVKNVLIQNFNNFVDRDFAYSKNTLGAYMMSLMRGDDWKLYRKKLSPGFSKAKANFDLLKVVNQDYVRYLEREVEKNELISVKDLGQMYGTEAITYLGLGTKADCFVNKNSIFTKMAKQMFDLDNFVRAFAMSAYLLLPSLVPIFKFNIMDPEAEAFLYGLFTGAVEHRQRTKQHKNDFVDVLLKLQKDERDMHLDMPTLGSQAIAYWIGGYETTSILISTCLYELGKHPDIQTKVREEINTALEKTDGEFTYEIITEKLKYFDAVVKETLRKYPPLPVINRVPSKDFPIPGTNIVLEKGVPIYIPSYAIHMDPKYFPDPEEFIPERFLGDNTILPYTYMPFGIGPRMCPGEKIAMVADVLALAHTVKNFEFTLRDDSQRIMEFYTKTTILSPKPDKMILRFKKIK*

>Ah CYP345C1

MLLTQCICADLLIISASLVLLIYIFLTRNFNYWKKQGIPFIEPLPLFGTFKEVFLRKNNMSEALSEIYFKSKDPYVGLFVVDKPVLLINDRELIKSILVRDFSNFSDRSFGTREGDLSEYMLPFMKGKTWKTYRKFNTPAFSRATLKSVYFRTIQAAGQEAVEYLETTLKTSDLVNCKLLASMFTVEMLTCITLGVAPKCFRDEHSKYLKMGHGMFAVDKLKRDIAIASYLLYPSLVNMFKFNLVDPDSENYFRKVFFETIRRRKATASAEDLIDILLKWRHDNQFAKEYVELDDDKLVAQALAFFLAGHEGTTSVIAFTLYELAANPDVQERLRAEIKDYLHSSENNSLNFETLFSEMKYFDMVLKEVLRKYPPVSMMDRRCLNDYKIPGSSHIIKGGTTLFIPSLAVHRDPQYWLNPEKFDPERFTEENMRDIVPGTYFPFGDGPRVCIGEKIGQISVKLCVARIIERYQIELRDDTQKIMKIPVRAQTLIPEDDVVMLRFKKL*

>Ah CYP345F1

MHINVINIPTIALLGFTLILVYRFLTRRHNYWKNRGVPYEEPFYLAGNFWEVFSGKSQIGKHLGELYSRYKDPYFGIYILGKPYLVLRDPDIIKNVVIRDFKNFEDRTFACDKNADKMAANSLFIMRNPDWKYIRTKLTPIFTSGKLKGMMTLVKKSAKDMELYLTKFNKQTVEIKSISCKYMTDVVASCFFGFEAESFQNENSEFYTYTQTMFSSKAGFFSLFSYFFAPLFVSIFKLKFMKYEILQRAFISTLKYRQENNFRRNDFIDLLLQLKDRCNEDKMQFDFGPDRMLAQSITFFVAGFETTSNAVAFTLYELCLRKDCQEKLRDEINAFIKDEDDITFEKIQKMKYLDMVLSETLRRYPFGPFLNRHCKEDYVIEQTGLKVDKGTPVLIPLDGLHHDPEYFPEPDIFNPERFADDKKQNLAQACVYMPFGMGPRNCIGDRFGAICAKIGLVYILRKYKVERCEETPIPLVLNPRSPFMVPLDGLKMIVTKL*

>Ah CYP347A5

MWLILVVFILTIVYYLFEKQYSFWSKNGVPGPKPVFLVGNFGMNVLGRKSMGDVISDIYKKYENYPFVGVYKSTTPVLLVRDPEFVKNIVIRDFKHFYDNDIIIDKDVEPLFGRNPFCLKGQEWKQKRAQLSHCFTSGKIKGMYVFLEKNASRMIKYIKEETNASPTLELREVCIRFTLDNVAACAFGLEGKCFDEPLSQFRDIANRFLSPGSISAFKFTILSLCPWLGGYMKIKFVPNDVEQQLMNIVRSSIKYRKENNVVRNDFLDAMLQISSSTDVFTDIDIVANSASFFGDGYETSSRVMAFALFELALNVEVQEKLRKEIQNAAEKNNNILSYDVVNELPYLDACLNESLRKNSIIASLGKTCTEDYTYTTTDPDLKPLTVHVKAGSTIMIPIEALQNDPKYFENPDKYIPERFLEKNANSFNKYTFMPFGEGPRSCIGQRFGLTQIKVGIANIINNFKLTFNAKTKFPLKFDPHYIMLDAIGGLWVDFKKIN*

>Tc CYP345A2

MLLTPYLPLDTVVLLSVLALLLYKYFSRNFDHWEKKNVFYFKPIPFFGNFVDISLFRTTIGEHLAKLYNQTTEPFFGIFVFDKPHLIIKSPELVKTILVRDFNNFDDRCIASPHHDPLVKNMLFLNKNPEWKNVRVKMTPVFTTGKLKGMIPLINDVGETMTKYIAQKIPNFSLEAKEICAKFSTDVIAKCAFGINANSFKNEDAEFRKIGRRIFDFRWSTAIQQTSYFFLPGLVNLLKFRMLDKDASDFLRETFWHTIKLREEKNLKANDLIDAIIALKDNQEFCKNMNFEGDKVVAQAAQFFVAGFETTSSTMAFTLYELCLQPQFQRRVRAEIATCLKEHNGLTYEALQSMKYLNMCVCETLRKYPVLPFLDRTCKEDYKLPNSNVVIEKGTPVFIPMFGLHYDPQYFPNPQKYDPERFSDENMQNITPFSYIPFGEGPRNCIGERFGLIGTKLGLIHILSEFEVEKSSDTPVPLEFEPKSFVLASKVGLPMKFKKVMTSAA

>Tc CYP345A1

MLLTSYLPLDTFLLLSVFTLLLYKYFSRNYDHWEKKNVFFFKPTPFFGNILDISLFRTTIGEHLAKLYNQTTEPFFGIFVFDKPHLIIKSPELVKTILVRDFNNFDDRGVASPHHDPLVKNMLFFNKNPEWKNVRVKMTPVFTTGKLKGMIPLINDIGETLTKYIAQKTSNLSLEAKEISAKYTTDVIAKCAFGINANSLKNEDAEFRNLGRRFFDFRWSNAIQQTSYFLLPGLVNVLKLRVMDKKDSNFLRETFWQTIKLRQENNSKAKDLIDAIIAMKENKEFCKNFNFEGDKVVAQAAQFFIAGFETTSATMAFTLYELCLQPQIQSKVRTEIMTCVKEHNGLTYEALQDMKYLNMCVCETLRKYPVLPFLDRTCKEDYKLPNSNVVIEKGTPVFIPMFGLHYDPQYFPNPQKYDPERFSDENMQNITPFSYIPFGEGPRNCIGERFGLISTKLGLIHVLSNFEVERSSDTPVPLEFEPKSFVLASKVGLPMKFKKVMTSAA*

>Tc CYP345B1

MGLELQFSIINLLILLITFWTTLYLYFTRKFNHWKSKNVPQVAPIPFFGNAFEVFTWRKNIGEFARQIYNSTTKPFIGFFICDEPYLLIRDPELVKSILVKDFAVFSNRSISENKENDPMGSHLLFLLKTPDWRDMRRKITPVFTSGKMKNMYSLISEAGNDMIQHMRKEVSKSDQLEMREVAARYTTDAITSTSFGINANCFKNEKAEFREVSRRVFNWAIWERSISTTCYFIAPNLVKLFKLKFIDSASATFLREAFWRTMTDREEKKFVRNDLLDILIDIKKQEDINDPYKLDGDKLVAQATQFFVAGFETTSSTICFTLYELAINKDLQNKLKSEIRDVVRKHGEISYNSLKDMEYLDMCIKETLRKYPVLPFLDRKCDTDYRIPGSDVVLEKGSPVFISVSGLHYDPQYFPDPDKYDPLRFTEENIKSRPQFTYLPFGEGPRNCIGARFGSVSSKSGVAKIISEFEVDLCEKTQHPIQIDPKGFLMAPVSDLVLKFKRLDD

>Tc CYP6BM1

MAFISLTILAILLLLAVTLFYKWKFQYWKTRQVPFLPPKIPFGNIPNPFTLKEKPGIFIKQFYQEMKRNGWKHGGFYFFACPVYMVVDPDYIKQVLIKDFQYFIDRGIYSNEKDDPISHNLFSANETKWKYLRTLLNPSFTSKKIKAMFTILLACEKFLTQNVENNQVIDANHIFANFTIDVITTCAFGLDCNGNQNLALKHFGEKTINYSKLKMFVLMLASIFPKLAKKFGCVIMHKNTQEFFVQFVADIIKFREENNYTRDDFMQFLIKLKNKSAKQVFTLEEVASQCVLFFIAGFETSHATMSFALYELAKHQDIQQKVREEFWAVVDSQESEITYEAVQKLKYLDQVLNETLRKYPVVMYLTRRCIKDYKVPEDGAVIEKGTTVIIPTVALNYDENYYPNPKKFDPQRFSEENKKLRHPCIHLPFGLGPRFCIGRRFALIQVKVGLVLLLKNYSFTVNPNTREPIKLKIHSFISAPEDKILLDVHKL

>Tc CYP6BK1

MFSVLVFLIIVVVFLYYKWAFTYWHRRNIPYLEPSFPFGNLPNLFQRKENIGLTVEKFYNEMKRRKWKHGGVYFLVSPVYCVIDLEYVKNIMNRDFEYFTNRSIYYNEKDDPLSAHLFAIGGQKWRNLRTKLTATFTSGKMKAMFQTLLECETNLLKQIEDCNNPINIKEILSCFTTDVIGSVGFGLECKTFEEKNSPFRIYGKKVFKDSVLRSLKRAFLTSNFPELARKLGILAFPSEITDFFLKVVADTVKYREENNRSRNDFLQLLIDLKNSGTLSLEEIAAQCFLFFLAGFETSSTTMTFALYELAQHQDIQDKVREEIDAVLKKYGGKITYEAIHDMKYMNQVIDETLRKYPAASIITRTCVKDYKIPDQDIVIEKGTSVIIPVLGIHHDEKFYPNPEKFDPERFTEENKAARHHYAHLPFGEGPRICIGMRFGLVQSKVGLTSLLKNYIFKVNKKTIEPLKMQARSLILAAEGEIWLDVYKL

>Tc CYP6BN1

MNTTIEILTIIILTIFCFYKWKFSYWKSRKIPYLDPKFPFGNVSNPFFMKEKVGMTLARFYNDMKQRGWKYGGFYFGVSPICLIVDPDLIKNIVTRDFEHFVDRGVYFNEKDDPLSGHLANLDGQKWKNMRRKLNPTFSASKTKNMFPIVKFCVENMMEKVKSCYENAHKCCNINEVFPAFSINVICSCAFGLQEENALFKAYADECVSWTKYKYLVQILATFFDKTAKRMGITTVPRNAAKFFMKVVTDSVKYREENDYVRSDFMQLLIEMKKQNQLTIEEIAAQCFVFFIAGFETSSATLTFALYELAKNQNLQNVARDEILANCPNEITYDSLADLKFLDQVVDETLRKYPPLLYVTRQCVKNYKIPDEDVVIEKGTLVAIPISALHSDEEFYPKPETFEPERFSKTEKSLRHPYTFLPFGEGPRMCIGKRFGLLEVKLALTCLLKNYKFSVNCKTKEPLAMRVDTVVLAPKDEIWLDLEKI

>Tc CYP6BK2

MLFSDSLTKDLFGCVLTFAILIIAYFKWSYQYWKRKNLPYLKPTFPFGNITGLLTRKETFGVIIQKVYQEMKNCNWNHGGIYLTLNPAYCAIDLEYVKNIMTKDFQHFVDRGSYYNEKDDPLSAHLFAIGGQKWRNLRTKLTPTFTSGKMKQMFQTLVDCESNLAKKIDMEYEKKHPIDIKEVLGCFTTDIIGSCAFGLDCKTFEHENSPFRVYGKKLFKGSTLRNIKRSLLTQNFPDLAQKLGVLALPEDISNFFMKVVKDTVRYREKNGFTRSDFLQLLINLKNSKVAEEEGYKHDGNTLTLEEIAAQSFVFFIAGFETSSTTMTFALYELAKNQEIQSQVREEIDAVLKKHDGKITYDSIQDMKFMNQVIDETLRKYPPIPFVTRKCVNNYQIPGENIVIEKGTTVIIPILGIHYDKDYYPNPEKFDPERFTEKNKNARHHYAHIPFGEGPRICIGARFGLMQTKVGLISLLRNYKFTVNNMTREPLRMQVDSFVLAAEGEIWLDAHKL

>Tc CYP6BR1

MNIPSPYFEILGTVLALLVAIIFYYHHAFQFWARKNIPFIPPRFPLGNTNVVLPRGLTIGLLSKTFYDYFKQRGHKIGGVFLVTEPNLMILDPDYIKNILLKDFHHFVDRGFYFNEKTDPISANLFTIDGDQWRNLRTKLTPTFTSSKMKIMFQTVKNCCNNMLEAIASNTDLDKDLKEFLGRYTVDVVGSCAFGIECNSFKHPDAEFRKMGLRAFHFSFLRSIRAFFIAYLPKLSRQMGLPSNNKDIREFFYKVVMETIQKREETQIKRNDFLQILMDLRKTEILSLDEVAAQVFLFFTAGFETSSSTMAMCLYEIAKEPEYQEKLRQEICEITNGEITYENLFEMKYLDQVFSETLRKYPPGQTLNRRCVKDYTLPGTSTIIEKGTPILISAIGVHRDPEYYPDPEKFDPERFSEENKKLRHPFVYLPFGDGPRNCIGMRFGTMQSKLGIASVVKNFKVSVSPITKRVELDPNTFLLNTIDKIYFRVEKIVK

>Tc CYP6BR2

MGLFLEDFWTEVFFLLTTLAILVLSYFHNAYKYWEKRGVPTIKPTFPLGNGDTLLPEGFSVGMLSRKFYEELKKRGEKFGGVYLVTQPNLVLVDPEYVKDILATDFQYFVDRGFYFNEKTDPLSAHLFALDGTSWRNLRIKLSPTFSSGKMKMMFKTVLECCGYMRDYIEDILDKDLDIKEIFSRYTTDVIGSCAFGIECNSFKYPDAEFRKMGKKVFSFGLWRSICSFCIIYFPNLSYHLGVPTNEKDVQNFFRGIVAESVNTRKEQNIKRNDFLQLLIDLKDHSALSLNEMAAQVFLFFAAGFESSSTTMTFVLYELAKHPDIQQELRNEIRKTLDKHQGQLTYEAVMEMKYLQQVIDETLRLYPPLATLNRRCIKDYTLRNTGIKIEKGTSVIIPSLGLHMDPELYPDPEIFNPDNFSEENKKKRPSFVHLPFGDGPRNCIGLRFALMQSKCGIATVVNNFRFSLSPATKPLRLNPNSFVLNTYDKIYLRAERLAM

>Tc CYP6BR3

MSVFTEFWPQILVFLALFLLVLVNYFNYCYRYWSKKGVATIKPRFPLGNTNIILPQGLSIGILSKQFYDEFKAKGHKFGGVYLVTEPNLLIVDPEYVKDIMSKDFQHFVDRGFYYNKEKDVLSAHLFAIDGSDWRNLRIKLTPTFTSGKMKMMFEAVVQCSKHMIENLDKNLGSDIEIKEVLGRFTTDVIGTCAFGIECNSFKYPDAEFRMMGKKIFEYDLWKSVKAFFAVNVPKVALQLGLTSTPDDVIDFFTRIVADAVKMRESNNIRRNDFLQILIDLKNTTSLTLDEMSAQVFLFFVAGFETSSTTMTFALYELARNEKMQEKLRFEICQVLDKTGGQITYESLIEMKYLQQVIDETLRMYPPLPTLNRRCTKDYVLRDTNIIIEKDTPILISALGLHMDPEFFPKPEKFDPERFTEEKKKERHPFVHLPFGDGPRNCIGLRFGVMQSKIGIITIIKNFKLTVSSNMKPVQFNPYVFLLNINDKVLLQVGKL*

>Tc CYP6BK4

MLITTSLTGDLLAVFLTLVLIIVTYCKWSYQYWERKHLPYLEPQIPFGNVGDSIKGKTNFGITIKKIYEEMKHNGWKHGGLYVFTEPNYMVLDLEYVKNIMTKDFQYFVDRSTYYNKKEDPLNAHLLNLGGTKWRNLRTKLTPTFTSGKMKMMFQTLADCQTGLLKRMEKERLTRQPIDIKEVLACFTTEIIGSCAFGLNCKAFEDENSPFRVYGRKVFATSKLQRLKRTIATIFPTLGYLFNISITPKDITNFYLNVVKDTVEYREKNNYNRNDFMQLLIDLKNNKLEGGTSNGGFTLKEVAAQSFVFFLAGFETSSTLMTFALYELARHQEIQDIVREEINEVLRKHNGNVTYDSINDMKYLSQVIDETLRLYPPASLVNRKCIKDYQVPDCDLVIEKGTTVLIPIMGIHYDKDYYPDPEKFDPERFTEENKNARHNYAHIPFGEGPRICIGMRFGLMQTKVGLSCLLKHYKFTVNKRTQEPLKMQPSPLILSAEGEIWLDAEKL

>Tc CYP6BK6

MFRFLDLVGVFIALFAILFAYFKWTYQYWSQKNVPFIQPTIPFGNSNLFRQKESGGIRMKNFYDEMKAQGWKHGGLYSILKPSYMVIDLDYLKNIMTKDFDYFSDRGFYYNEKDDPLSAHLFAIGGEKWRNLRIKLTPTFTSGKMKMMFQTLVDCAPGLVKQIDRKMESIDIKEVLGCFTTDIIGSCAFGLDFNTSNDNNSPFREYGRKAFVPTKFDIVKIIFAMSFPRLAQLFRLTLTRKDVSEFFLKVVRETVEYREKNNYRRKDFIQLLIDLKGEDGKTLTINEIAAQSFVFFIAGFETSSTTMAFVLYELSRRPDLQQKLRDEINTVLSRYEGSITYEATQEMKYMDQVINEALRMYPPVPMLGRKCVKDYKIPDQDVIIEKGTSILIPVLGIHYDQEYYPDPKTFDPERFNEENRKARHHYAHLPFGEGPRICIGMRFGLMQTKVGLATLLKNYKFKVGERTQEPLKFKVASFVLAAEGEIWLDAEKL

>Tc CYP6BK12

MPTLFDILGVSVALLVITYAYFKWRFQYWRQKNVPFLPPTVPFGNFNMLRQRDNGGILLKKFYDEMKSHGWKHGGFYSLFQPGYLVIDLDYLKNIMTKDFDHFTDRGFYYNEKDDPLSAHLFALGGEKWRNLRTKITPTFTSGKMKQMFQILVDCTPDLIERIDSFCREKKPIDIKELLGCFTTDIIGSCAFGLQFNTSKDKNSPFREYGRKAFTPTYFDIVKMTFAMAFPRLALALKLRMTRKDVSDFFLQLVRDTVKYRVENNYSRKDFIQLLIDLKSLTIEEIAAQCFVFFIAGFETSSTTMTFVLYELSRRHDLQQKLRNEINTVLAKYDGHMTYEAIQDMKYMDQVINEALRMYPPVPLLSRKCVKDYKIPDQEIIIDKGTTVCIPIWGIHYDKDYYPEPEIFDPERFNEDNKKSRHHYAHLPFGEGPRICIGLRFGLMQTKVGLATLLQNYNFRVAGRTQEPLKYKVASFVLAAEGEIWLDAEKL

>Tc CYP6BK13

MFSCFLTNIIGIFVAFFAVIIAFYKWSFGYWKQKNVPYLPPKIPFGNLANPLRPAENMGVQVQHFYAQMRAKKWKHGGLFFLANPTYMVVDLEYVKNILTKDFQHFVDRGVYYNEDVDPIGAHLFALGGTKWRNLRTKLTPTFTSGKMKMMFQTLIDCESNLHKKIQAQKNKPIDIKDVLGCFTTDIIGSCAFGLDCNTFEDEDSPFRKYGKKVFVATPLERLKIAIFMNFPKVGRFFKMRQVSKDISDFYSKVVKDTVEYREKHNYSRKDFMQLLIDLKNDGNALTLDEITAQSFIFFLAGFETSSTTMTFALYELAKNSEVQEKVREEVLAVLGKHGGKITYEAIQDMKYMNQVLNETLRKYPPVPFITRQCIKEYKIPDQEIIIETGTRVIIPILGIHYDPEYYPDPQKFDPERFSEENVNKRHHYAHLPFGEGPRICIGLRFGLMQSKVGLASLLSKYRFKLNERTKQPMKFQVKSFVLAAEGEVWLDAESL

>Tc CYP6BK14

MEMLFSAIILILALNYFYYKWKFTYWQCKKIPTLAPKIPFGDLPNPLKQAEATGVHIKRLYDQIKGKGWKHAGLYFLARPVYMVVDLEYVKNILTKDFHHFVDRGFYFNEKDDPISAHVFAVGGQKWRNLRTKLTPTFTSGKMKMMFQTLLECEANLLKRFEKEHKSAIDIKDILGCFTTDIIGSCGFGLDFNTFEDENSPFRKYGKKVFASNLTRRLQIFFFTHFPSMARALKVRHFSKDVADFFTKVVKDTVEFRERNNYKRKDFMQLLIDLKNCNSGLTLNEITAQSLIFFLAGFETSSTTMTFALYELAKNEKIQERVRDEIFSILGEEKITYEALQKMKYLALVINETLRKYPPLSFLTRECVQDYKIPDQDVIIEKGTKVVISILGMHRDQEFYPDPETFDPGRFSEGNVVSRHQYAYIPFGEGPRICMGLRFGMMQTKVGLVSLLRKFKFTVNGRTKEPIKFDVRGFILAAEGDIWLNAQKVC

>Tc CYP6BQ11

MSLITDNLTFDLVAILATILVGIIVYFKWQLSYWDRLGVPSLNPVLFFGDIKDFILAKCTLGEQFKEFYGKFKSKGYKHGGIFLGPKPFYVPIDPELVKHIMQKDFQHFVNHGNYFDEDADPLSGHLFNLEDAKWKNMRIKLTPTFTSGKIKMMFQTLADCTRGLKEIMDHSALNHAPADIKDILGRFTTDIIGSVAFGIECNSLKDPDAEFRKYGRKVFEVGFIDRLKTICILSLPHPVLRFFKLKFTKSDVEKFFMSAIRDTVNYREKNNIYRKDFMHLLLQLKNRGFVADDGKVTDEKENVKEKALTLNELSAQAFVFFLAGFETSSTTMTWALYELATNQDVQEKLRNEINNVLSRHDNKLTYEAMMEMTYMEKVIHETLRKYPPLPILTRKCNKDYTIPNTSIKLSRGTAVGIPVLALHTDPEYYSNPEKFDPEHFSEENVKTRPGFTWLPFGDGPRVCIGLRFGMLQSKVGLTALLKNYKIKLSKKTELPIKLDPKSFITTAKGGIWLDVEKLD

>Tc CYP6BQ10

MTLITNNITLDLVAIIATFLVGIIVYFKWQLSYWDRLGVPSLNPTLVFGDFKNYIFAKCSLGEQFKELYDQFKSKGYKHGGVFVGPKPFYVPIDPEIVQHIMQKDFHHFMNHGNYFDENADPLSGHLFNLEDSKWKNMRVKLTPTFTSGKIKMMFQTLADCTRGLDEIMYNSALNHAPADIKDILGRFTTDIIGSIAFGIECNSLKNPDAEFRKYGRRVFEVGIIDRIKIICILALPDSVLRLLKLKFTKSDVENFFMNAIRDTVNYREKNNIYRKDFMHLLLQLKNRGSVTDDEKVTDDKDNVKEKALTLNELSAQAFVFFLAGFETSSTTMTWALYELATNQDVQEKLRNEINNVLSRHDNKLTYEAMMEMTYMEKVIHETLRKYPPLPILTRKCNKDYTIPNTCIKLRRGTTVAIPVLGLHTDPEYYSNPEKFDPEHFSEENVKSRPGFTWLPFGDGPRICIGLRFGMLQSKVGLTAILKNYKVTLSNKTKFPVTLDPKSFITTAKDGIWLDVKKLD

>Tc CYP6BQ8

MLLNNFTLNILAVFITVLVGVIVYFKWHLSYWDRLGVPSLSPVLFFGDTKNLILSKCTIGEQFRVFYNKFKSKGYKHGGIFFGPIPFYIAIDPEIIKHILQKDFQHFMNHGYYINEEDDPLTGHLLNLENVKWKNMRAKLTPTFTSGKMKIMFQTLADCTTGLKKVMDDSALNHTPVDIKDIFGRFTTDIIGSVAFGIECNSLENPDAEFRKYGKKVFEIDFFGRIKTLCTFAIPHPILRLFRFKFYNSDVATFFMDAIRETVNYREKNNIYRKDFMHLLLQLKNRGLVTDDEKITGDKDIVTEALTMNELAAQAFVFFLAGFETSSTAMTWALYELAINPDVQQKLRAEINDVLRKHNKLTYEAMMDMTYMEKVICETLRKYPPIPVLTRKCTKDYTIPNTSIQLQRGVSVSVPVLALHTDPEYYPNPEKFDPDRFNDENVKARPGFTWLPFGEGPRICIGLRFGLLQSKVGLTAVLKHYRIKLNHKTQLPVTLNPRSFITSAKGGVWLDVEKID

>Tc CYP345D2

MIFVYLCCFLLFLYYLYRKNYKYWESKGVLTEKPFFIFGSFYDVALRRKHLFHKVREIYDKFSTPYVGIYIFNQPTLVIRSPELLKKVLVKDFDKFINRKVAANESVDPVFFHTLFSAKNDNWRNLRAKISPVFTSGKIKLMFPLMKECGTDLLNYFKKRSGGVIEARNVTKKYAVDIISSCAFGINSYCLKDDNSEIMQIATQLVDFKSFIRSISIFCFFFMPKLVDIFRLTFADKKASEYLMNVFKTTINERRKKTIVRNDLIDMLHNLKENSSSSDFTFDDVKMAAQALSFFSAGNDTTSITITFALYELALNTDIQNRLREEIRKRYEAHGDFTYEAIQEMKYLEMVLCETLRKYPLTIFLNREAVSNYTLEESGLTIDKGTSIMIPVAGLHFDEEYFPNPEKFDPERFSDENKSKIVPYTYMPFGDGPRICIGQRFAMLVSKVALAYILKDFAVEKTDSTTVPMQLDPGAIFLMNKNGVNLRVVEVK

>Tc CYP6BQ7

MLFSDSLIVDLVTIISTIVVVSIAYCKWKLSYWNNTGLPTLSPVFPFGDTKELLLGQKSFGEQFQDLYKEIRAKGWKHAGVYLAIQPFYLPVDPKIIKHIWQVDFQHFVNHGNYINEEVDPLSGHLFNLEDAKWKEMRVKLTPTFTSGKMKMMFQTLADCTHGLKTIMNECATSGTPIEIKDVLGRFTTDIIGSAAFGLECNSLKDPDSTFRKYGKKVFELQGWERIKVLAQFALPHSFLRAIKFKQTQSDVEKFFMKVVRDTADYREKNKIYRKDFMHLLLQLKNRGKVSDDEKVTQENGKTGEKALTMNEVAAQAFVFFLAGFETSSTLMTFTLYELATNPDIQGKLREEINTVLAKHDGQMTYEAMMEMTYMEKVLNETLRKHPPIPFLFRRCTKDYTIPETSVKLRKGDDVGISIVGIHNDPEYYPNPEKFIPERFNEENKNARPPFTWIPFGEGPRICIGLRFGMLQSKVGLTALLKNYKISLSSKTKMPLEMEKSGLVTTVEGGMWLNVEKIN

>Tc CYP6BQ6

MTFMTESLIFDIVTIISTIVVLTIAFWKWKLSYWDRVGLPTLSPILFFGDTKDVLLGRLTFGEQFQELYKKLKAKGLKHGGVYLGTRPFYLPTDPEIIKHIWQKDFQHFVNHGNYFDEEADPLSGHLFNLEDAKWKNMRVKLTPTFTSRKMKMMFQTLADCTHGLKDIMDEGASSGSPVDIKEILGRFTTDIIGSVAFGLECNSLKDPNTLFRMYGKKIFELSTGRRLHLFLQFILPERVLHAMKFKVTPSDVETFFMKVIRDTVGYREKNNVYRKDFLHMLIQLKNRGTVTDDEKITEDNGKTGEKALTMNELAAQSFVFFAAGFETSSTTVTFALLELALNQDIQQKLREEINTCLAKSNGELTYQAIMEMTYMDKVLQETLRKYPPVPFLTRRCTKDYTIPETSIKLRKGDHVGISVVGIQNDPEYYPDPEKFVPERFNEENKNSRHPFTWMPFGEGPRICIGLRFGMLQSKVGLAALLKNYKITLNSKTKMPIEMEKSSFITSVNGGVWLNVEKLS

>Tc CYP6BQ12

MAIFTSSIFIDIITIITTVLALTIAFFKWKLTFWEKLGVPTLNPVLFFGDAKNILLGKRQVGVEFIDYYKQFKPRGLKHGGVYLGPRPFWVITDPGLIKHILQIDFPHFINHGTYADEENDPLSAHLFSLEGTKWKNMRTKMTPTFTSGKMKMMFQTLVDCTPGLKDIMDEHASKKKAVNIKNILERFTTDIIGSVGFGIECNSLKDPDSLFPKYGKKVFDLTPAEQLKQSLLFFLPNPVLKAMKFRITNKDVEDFFMKTVRETVLYREKNNIYRKDFMHLLLQLKNRGTVTDDEKIIDHSNQKQEPALTMNELTAQAFVFFLAGFETSSTTMTFALYELATNPDIQEKLRNEICTVLEKHNNELTYNAIAEMTYMDQVLHETLRKYPPLPILQRQCNKDYIVPETNIRIPKGVDVAITILALHRDPEFYPNPEVFDPERFNEENKNARTPFTWLPFGDGPRVCIGLRFGMMQSKVGLTALLKDYRVTLNKKTKVPLEMSKNSFIIAAEGGIWLDIEKINT

>Tc CYP6BQ1

MNYTFLALFVTFLLGVISYLKWKLRYWDKKGLPTIPPKLPFGNLTDVIFGRKSFGEQFVEFYQILKAKGYRHGGIYFGASPLYVPTDPEIVKHIMLVDFAHFSSHGGYVDEESDPLSGHLFNLDDIKWRNLRVKLTPTFTSGKMKMMFQTLAEVSYNLKNVLDESVINKTPVDARDVLSRFGVDVISSVAFGLECDSLKDPNVSFRKYGKKAVDFDIWVRIKLFMQVLLPHCVLRAIGHKFTKTEVEEFFMKSIRDVVEYREKNNIFRKDFMHLLIQLKNLGTVTDDGQILDETSGSKEVGLTMNQVAAQAFVFFMAGYETSSSTITFALYELAMNPPLQDKLRDEINTILAKHDNKLTYAAMMEMTYMEKVIQETLRKYPPLPIIMRLCTKDYVVPGTDIEIKKGVGVMIPVLGLQTDPEYFPDPDVFDPDRFSEEKKKERPGFTWLPFGDGPRICIGMRFGMLQSKVALTTFIRNYRVKLNEKTKIPLKIDKGSFTTRAEGGVWLNLEKLN

>Tc CYP6BQ2

MLLVNGSLTYDLVAILVTVLAGLVVYYKWAFTYWKKRGLEYLEPSIPFGNCASLFKAKYNFGEQFAEFYKQFREKNLRHGGVFMFARPFYIPVDPAIMKDIMQKDFQHFVDRGFYVNEEIDPLSGHLFSLEGNKWKNLRAKLTATFTSGKMKMMFETMSRCAEELDNYVNEASKRKETIDIKETLARFTTDIISSCAFGLESNCLKNPDTKFRYYGKRTFQNTTWENLKNLIQFMTPMYLLNLVKFKTTKQDVEDYMLNLVKKTVDYREKNNVYRKDFLHLLLQLKNKGKITDDETFLDNNNKSKEVALTINELAAQVFVFFAAGYETSSTAMTFTLYELCLNQKIQDKMREEIKTVLAKHNNKISYEALMEMTYMEQALNETLRKYPPVPFLNRKCTKAYDVAGTNLHLDEGTMVVLPILGLQHDPEYYPDPSKYDPDRFSEENKNSRPPFTWMPFGEGPRICIGLRFGMLQAKVGLATLLENYKVTLSQKTKTPLEMDCKTFITTTKEGIWLDFTRLN

>Tc CYP6BQ4

MLLVNGSLTYDLVAILATVLAGLVVYYKWAFTYWKRRGLEYLEPSIPFGNCASLLKLKYSFGEQFAEFYKQFREKNLRHGGVFMFARPFYIPVDPAIMKDIMQKDFQHFVDRGFYVNEEHDPLSGHLFSLEGNKWKNLRAKLTATFTSGKMKMMFETMSKCAEELDNYVNEAAKREETIDIKETLARFTTDIISSCAFGLESNCLKNPDTQFRYYGKRTFQNTTWENFKDVIQFITPTYLLDLVKFKITKPDVEDYMLNLVKKTVDYREKNNVYRKDFLHLLLQLKNKGKITDDETFLDHNNKSKEVALTINELAAQVFVFFAAGYETSSTAMTFTLYELCLNQKIQDKLREEIKTVLAKHNNKISYEAIMEMTYMDQALNETLRKYPPVPVLNRKCTKAYDVAGTNLHLDEGTMVVLPILGLQHDPEYYPDPSKYDPDRFSEENKNSRPPFTWMPFGEGPRICIGLRFGMLQAKVGLATLLGNYKVTLSQKTQTPLEMDCKAFITTTKEGIWLDFTRLN

>Tc CYP6BS1

MESALMEAGIIFLTIVSFVYLYFKHAYTYWKARNVPQLSPTFPFGDMASVIFRRHNMGDKIKEIYDETKARGHKYVGLYFFSRKAFLPIDPVLIKKILAPDFQHFHDRGIYYDEENDPISAHLFSLAGPKWKNLRARLTPAYSPGKLKFMFDTVLACGEEMADILKEIAKREGKVEIKEILARYSTDVIGCCAFGIDCNSMRNPTAEFRLMGRRAFTQTIGDVLKMIVIRSFPRLAKLLGFGVFSPTVTGFFQRVVNETIRYREENNVTRNDFMQLLIQLKNKGKLEGDGEEKDGAPAVGTSLTLDEAAAQAFIFFLAGFETTSTTISFALFEMARNESIRTKARTEINKVLGKHGGKLTYEATMEMEYLDTVISETLRKYPPAPVFLRKCTKTYRVPDTDVIIEEGLSVLIPAYGLHRDPEYFPDPETFDPDRFNEDNRSKIWDYTYIPFGDGPRICIGMRFAMIQIKIALALILKSFEFPLSEKTILPMKMENSGIILAPIGGIWLNLIPLE

>Tc CYP345C1

MMFVIFFAVLFLIYYYLTRNFKHWEKKNVPFIKPLPFFGSIYDGVLMRHSIGEVFYDLYYKSTKPFVGFFILDKPCLLIRDPKLIKKILVNDFQYFYDRNAVNNKRDDPISTHILFILKNPDWRELRTKMTPVFTSSKIKIMSELIENASHEMTNYLNNHIKDYNSVEMRDVCLKFTVDVIGSTIFGVQANSFKDENSQFSSVAKRLIDWDDIVTAFRFRCYLLAPLFVNLFRMKLFPPDCVNFLKNTFLDIMDKRSVSNKSRNDLIDILLQMKNDNRNFIEGDILVSQALMFFVAGFETTSSTMGFALYEFARNPDIQDKIRNEIKDISDKYGDIKYDSLKEMEYLDMCVKEVLRKYPVVPFLDRKCNTTYTIPDTNVTIDKDTPIFIPSLALHYDPQYFPNADIFDPERFSSNNKTGIDSFAYLPFGEGPRNCIGARFGLLTAKLGLVHILKEFVVSCNEKSNEKIKFNPKGMVLSALNGINLKLSKVEKAT

>Tc CYP348A1

MEQDWFFYSVTLFTFAVIFTFCYIRHVYTFFERKRIPRAGHPTFPFGNLYDVFFEKVPIFMQLWKFYNRFKRKKCKLGGFYLFLKPAIVLVDTTLVQLFFTDVFAKDERDDLDANFCGQVLASVVKSVEKVWPKLGPFAENEANFTQILGQELVGLCAREFFDSNDAKLCEVEGSRVFPLFRKCVSLSYPQFCKNENHRFRGKNFEKAAKNGVLQVLQKNGFSAKDRVDFIAELVDYLGKSSNLILFTLYELSLNREIQDDLRGEILRFRRNKDTFTYTELEQLTYLETVICETLRKYPMKPFVHKVCQKTFEQNGIKFDTNQTVFLSIFGIHHDVDNYINPELFDPDRFSEENEMFIDPIKYLPFGHQIKNDFDSLYAKLIMKMILIELLSKYEFALGKKSPKCITFDENLFEIKSREDMWLKIKKL

>Tc CYP347A1

MLPIVLFILACTICYFFVKINHAYWKKRGVVGPKPSFLVGNLGKSFILKSSPGEIYTEIYRKYKDASVVGLFRSETPVLLVRDPELLKEITTKSFQHFRNNDIDVDKKHDPLFGRNPFVLKGDEWKTVRAQLTPGFTSGKMKWLYSYLETNSKKMVEYILRVPEATNGQGYEAKELCIRYTLNNVGDAVFGIEGKCFEEKNSEFRKLSKEFLSPGSYGIIIMFLATLFPPITKIFPMRFVTKSVEKKLTNLVSDALKHRQVNNIVRNDFLHIISQLKKTNQDFTDVDVTAHAAGFFADGSETSSIVMSFVLFQLSINPDVQSKLRAEVTEAFNNNNNNMPYEVVQDLPYLEAVIQETLRIQPPIHSLQKLCTKSFTWTLKETNKPLVIEEGTPIIIPTHALQSDPQHFEDPESFQPERFLGENRENIKKCTHMPFGEGPRACLGQRFGLLQIKVGLAYIVKNFELSLNKKTKLPLKYDPINPLVTPTGGLWIDFKKIEKKKCNQIKSFHVMYLVIFPNIL*

>Tc CYP347A2

MIPIILTITVVTIVYYLLHLNYQYWKKRGIANPKPVFLLGNFGKCFFLRASPGQVYYDVYNKYKNEQVVGLYRATTPVLLVRDPELIKEVMVKSFSSFHNNDVYVYKKRDPIVGRNPFVLKGEEWKSVRQQLTPGFTSGKMKWLYPLLDEVAENLVKFIENQPKTLNAKDVCARFTLNNVASCAFGIEGKCFEEENNHFRQMAIEFLSPSSWSFIGLFLVLLNPSLLKVLPIKFVTKNCEKKLIDLVQQTVKYREDNNVVRNDFLHILTQLKKTSSEFTDIDVVAHAAGFFGDGYETTAGVMSFVLYCLAENLDIQTKLRELVDKVFENNEEKLPYEALQEIHFLDAVVNETLRLHPPALHFQKLCTQDFTFVLKNANKSVTIEKGTPVILPVYGLHHDPDYYESPDLFKPERFLPENKDKTVKCTFMPFGEGPRSCLGQRFGLLQVKVGVAHIIRHFELSVNGKTQRPIKYEPTNPVTAPVGGFWVNFRKIR*

>Tc CYP9D1

MWPFVVQKKHITEIIADIYRQFPTDRYVGYYHFMTPTLLLRDVELIKQIGVKEFDSFHDHVSVSNTNTEPLLSEALTNLPGEKWKEMRATLSPAFTSSKIKNMYSLISECAENFVDFFQGREQDVEIKEVFSRFSNDVIATAAFGIRVNSVQEPNNEFYAMGRSLTTFSAFQILKIFLAHLVPKIAEFFEYRVLPRKVTDFFEGLILDNMQKREAEKIVRPDLIHLLMEARKGKLKHDNSNEGGEGFATVEESEIGKNTKQVELTDEKIVAQALLFFFAGFETVSTGVSFMAYELATNPHVQKKLQKEIDLTLQENHGKISYNVLQSMKYLDQVVCESLRLWPPAPQTDRLCNKNFVIEASKPHERTFTVEKDTMVMISMFAIHRDPQYFPDPEKFDPERFSDENKAKIVPGTYMPFGVGPRNCIAKFDIVPNGKTVIPIKISPSTANMVPQEGIHLTFKPRL

>Tc CYP9D2

EFDSFHDHLPFVTEATEPLLSKSLLNLLGDEWRKMRATLSPAFTSSKMKHMYQLIYECAERFTQHFGNRTVDVEIKDVFSRFSNDVIASAAFGIQVDSLKEPGNEFYSMGKAVTSILNGFQMVKVFLAFLFHAIFQLVQFRAIPPKVTSFFYDIIIDNIKKRKNEGIVRPDMVHLLMEAQSGKLKHDNNTEQVDGFATVEESEIGKNSNKIQLTNELIVAQALIFLLAGFDSVSTGLSFMAYELATNPDVQKKLQEEIDSVLKKNQGEVTYIEIQSMKYLDQVLCETLRLWPPAPQTDRYCNKDFPIDANFTIEKGVMIEIPIFAIHRDPQYFADPDKFDPGRFSDENKSKIVPGTYIPFGVGPRNCIAPGSRFALLEIKILFWHLLSKFDILPNEKTVVPFKLCKRSSSLVPNEGIHLRFRPRK*

>Tc CYP9W1

MWYVYLSIFCGFLALFIYYKIIKPANFWKKRNVPHESTFALLQRKHPATILTQLYQKHKDQRYVGYLQLNHPFLMIRDLELIKQLTVKDFESFHDHYPFSYAKNDPILGKSLLMLNGQDWKQMRATLSPAYTTNKLKNMHQLMTECAQNFVGHFKGREKVAVDMKDIFTRFTTDVIASTAFGIKTDSLNQPNNRFFLMAKSLSKFGFIQVVKILISSFAKVGIFPSSVVNYFGTIIKSNIRERKQSCIVRPDMIHLMLEANKGQANQLTDDDIVAQAMTFFFAGFETVATACSFMAYELAIHPEIQQKLQAEIDSVGGVTYESLLSMKFLDQVVSESLRLWPPGFQIERMCTKSYKIEPKNHNEKGIVLEEGVSLIIPVIALHHDPDLFPEPDRFDPERFSEANRSQIVPGSYLPFGVGPRNCIASRFALLEIKTLFFHMLSKFDIVPIDTTQIPLKLSTKKMHLEPEKGFRLGIRPRKV

>Tc CYP9D3

MLTIILSVVLGALIYYKLIKPASFWEERNITHASAWPLIGSMWESVIQKKNFNEISADIYKKYPNDRYVGYMQFSNPALLIRDLNLIKQIGVKAFDHFHDHLAFVGVKAEPLFSKSLAILQGEEWKQMRATLSPVFTSSKMKNMYHLIYECAENFAKHFQGRKEDVEVKDIFSKFTNDVIATAAFGIQINSLEEPNNEFYLMGKLLTTFKGWQLINNNTILYRLIFEILFQLTKLNIFNQKVIKYFYDIIIDNIHKREKEGIIRPDLIHLLMEARKGKLKHDNSNEGGDGFATVEESDIGKNSKQLELTEDLMVAQALIFFFAGFETISTSFSFTAYELATNPDVQKKLQKEIDSAFQENHGKISYNVLQSMKYLDQVVSESLRLWPPAPQTDRFCNTDFVLEPTKPDERRFTIEKGVTTIIPIYGIHRDPQYFPNPDKFDPERFSDENKAKIVPGTYMPFGVGPRNCIGSRLALLELKTLFFHLLSKCDIVTNSKTVIPLKISPNNGNITPKEGIHLSFKPRKINGKM*

>Tc CYP9D7

MLTIILSVIFCALIYYKLMKPANFWKKRNITHVPSWPLVGNMLDSIIQKKHILEISTDIYKKYPDDRYVGYMQFTNPTLLIRDIDLIKQIGVKIFDSFHDHVTAAGIETEPLLNQSLISLHGEDWKRMRATLSPAFTSSKMKNMYHLIYECSENFAQHFQDGKQNVEVKDIFSKFTNDVIATAAFGIQVNSLKEPDNEFYSMGKALTTFQGFQLIKFFFVQLVPKIAEILKVRFLNPKVANFFTSIIINNMQKRERDGIIRPDLIHLLMEARKGKLKYDKGNEFGDGFATVEESEIGKNTKQVELTDDLIVAQALLFFFAGFDTVSTGASFLAHELATNPDVQKKLQEEIDMVLEENHGKISYTRIQSMKYLDQVISESLRLWPPAPQTDRFCNTDFVLEPTKPHERRFTIEKGVTTIIPIYGIHRDPQYFPNPDKFDPERFSDENKAKIVPGTYMPFGVGPRNCIGSRFALLELKTLFFHLLSKCDVIPNEKTVIPLKISPNNASITPKEGIHLTFRPRNFVQFKKSF

>Tc CYP9D4

MLTIILSVILGALIYYKLIKPASFWEERNITHASAWPLVGSMWESVIQKKNVNEVSTDIYNKYPNDRYVGYMQFTDPVLLIRDLDLIKQIGVKVFDNFRDHHAIAGFNAEPLFTKSLIFLEGEEWKQMRATLSPAFTSSKMKNMYHLIYECAENFAKHFQDKKQDVEVKDIFSRFTNDIIATVAFGIQINSLEERNNAFYLNGKSVTSFNGKQLLKFFVAQLAPQIFEFFKLCVFDQNSVKYFYNIIIDNMHKREKEGIVRPDLIHLLMEARKGKLKHDNSNNEGGDGFATVEESDIGKNFKKMELTDELMVAQALLFFFAGFETVSTGASFLAHELATNPDVQRKLQEEIDSVLEENQEKISYITIQSMKYLDQVISESLRLWPPAPQTDRLCNTDFVLEPTKPHERRFTIEKGVTIIIPFYGIHRDPEYFPNPDKFDPERFSDENKAKIVPGTYMPFGVGPRNCIGSRLALLELKTLFFHLLSKCDIVTNSKTVIPLKISPNNGNITPKEGIHLTFKPRNVA

>Tc CYP9AC1

MINMNYMITKHNKEQKNHGNKIQQHRKRITGTIYLGIFCAIFGYFYYYVKRQQNVWHQKGIKYCKPKFLLGSLEIIRSKSLAQIIQELYNAFPEERYHGFYQFLLPTLIIKDPDLIKQITIKDFDHFVNHRRFVNADDDPFWSRNLRGILVFARRDEKWRDMRATLSPSFTSSKMRIMFELMDECAREFVQHFKSQNSDTVELEMKDTLRRFTNDVIASTAFGLKINSLKDRKNEFLLMGAAATDFGYIRTMKFLLYTLSPKLCQLLKMKIIPTKVADFFTVIIKDTIKKRKEKNIIRPDMIHLLLEAQKGRQHPEEHLDIDEGFSSVSESKLTKPNEKLEITTEDIISQALIFFLAGFDTVSTLLNFLFYELAVNPDVQTRLRTEIQSVDEKITYETLLKLKYLDMVMSEALRKWPPAIATDREVAKNYTIEPTKPDEKPLLLEEGMLCSIPICAIHRDEKYYPEPEKFDPERFNDENKHKINPLTFIPFGAGPRNCIGSRFALLESKLLVFHVVKNFEIVPIDKTLIPLVMDKYQINWSVKGGTWLGLKNI

>Tc CYP9Z2

MFWILIVIAAIIAISYHQLRRYHKYWSEKGVQQGDPLPLFGHCLGTTLQKQSLSEFIQMSYNVAPDARYCGIYQFYSPILIVRDPELIKQITVKDFDHFVNRMAFIPEDCDPLWGKNLVALTDQRWRDMRSTLSPAFTSSKMKYMFSLISESAEQFAQYFANQDQDLITIEMKDIFTRFTNDVIANTAFGVKIDSLEERNNEFYLMGIEATDFRGFWRNMKIVIYFIFPTLFKSLGWRFFSKNVADFFVKLVRDNIAKREKHGIVRPDLINLFLEARKNGLKYEEQEHALDTGFATAEESEIGKNGMVRQTLTDVDIAAQALLFFFGGFDSVSSMMCFMSYELALNPDVQAKLLREVDETFESCDGKLTYEALIKMKYLDMVVSETLRLWPTNVSADRVCTKPYTIEPKNPNEKALHLEKNTNVIVPIFGIHRDAKNFPEPNRFDPERFSDENKANIRPYTYLPFGAGPRNCIGSRFALLETKALFFHLLSKFEITPVEQTQIPIQLNRKSFNMTAENGFWLGLKRRKNDN

>Tc CYP9Z4

MLWILVSIAIIALSYLFLIRPFKYWTNLGVEQGNPVPVFGHSWGTIVHTQSFADLVQMVYNIAPGARYSGMYQFFTPTLVLKDPDLIKQIAVKEFDSFLNHRSFIPEDVDPLWSKNLFSLTNQKWRDMRSTLSPAFTSSKMKYMFVLISESAEQFIGHFLKQKEDVITIEMKDTFTRFTNDVIANTAFGVKIDSLEERNNEFYLMGKEATDFKGFWRNLKFLILLSCPTLFKYLGINFFPKAVSSFFRRLVKDNINNREKHGIVRPDMINLLLEARKNVEESDIGKNGAIKQKITDDDITAQALLFFFGGFDSVSSLMCFMSYELGINPDVQEKLHQEIDETLEACKGKLTYEALMKMKYMDMVISETLRLWPTAVASDRLCNKPYTIEPKDPTEKPIHLEKNSVVWIPIFAIHRDPQYFPEPNRFDPERFSDENKNNINPYAYMPFGVGPRNCIGSRFALLETKALFFHILSKFEIVPIEKTEIPLQLNRKSFSMTAENGFWFGLKRRESNK

>Tc CYP9Z3

MIWFSTAVITIIALFYWLLIRPFKHWIKKGVAQGNPVPVFGHLLGTILHKQSFSEMIQTVYNIAPDSRYCGMYQFFQPMLVLRDPELIKQITVKDFDLFMNHKGFIPPDCEPLMGKVLILLTDQKWKDMRNTLSPAFTSSKMKYMFGLISESAQRFTGHFSKKNDNVITIEMKDIFSRFTNDVIANTAFGVEIDSLEEPNNEFYLMGLEITNFQGVVTFLKFLTFVILPRVAKFLKLTFFSKKVGKFFTKVIKENIANREKHGIVRPDMVNLLLEARKNSDTQKITDEDITSQAIIFFMGGFDPVSSLMSFMSYELAANPDVQTKLLREIDKTYERCDGKLTYEALVEMKFMDMVVSETLRLWPANGMTDRVCNTSYIIEPKDSTEKPLRLEKGDVVMVPIFGIQRDPKYFPEPNRFDPERFNEENKIKIKPYTYLPFGTGPRNCLGTRLALLETKALFFHLLSTFEIIPVEKTEIPLRLTRKSFNMTAENGFWLGLKRRSDKL

>Tc CYP9Z1

MILLCLIAVIVILSYRFLYRPHKYWLERGVIQGNPKPLLGHFMGTTFHKQSFADCIQMVYNMAPNSRYCGMYQFTMPTLVVRDPDLIKQITVKDFDYFLNHRTFIPEECDALWGKNLFALTNQKWRDMRSTLSPAFTGSKMKFMFPLISETAEYFIRHFLSKQEDVVTVEMKDAFTRFTNDVIANTAFGVSCDSLKERENEFYLMGKEATDLKGFWKNIKLLLFFLIPQVFKLCKVRFFSKSVANFFTNLIKGNITKREEFGIVRPDMIYLLMEARKNVSNEPISDEDITAQALIFFFAGFDTVSSAMCFMSYELATNPEIQEKLLQEIDSVEGKPTYDTLMNLKYLDMVVSETLRKWPISVATDRICNKPYTIEPKSPDKRPLLLEKNLAVWIPVYALHRDPNYFPDPNRFDPERFNEENKANIKPYTYLPFGLGPRNCIGSRFALLEMKILFFYILSHFEITPIPRTQIPLKINKTQFALTAEDGFWLGLKRRQK

>Tc CYP9Z5

MFWALILAAVLLVTYWLLSRPYKYWTERGVKQGKPVFIFGDYWGPMLRKQTNAEVVDMLYNQIDKEARYCGIYQFLTPVLLIRDPELIKKITVKDFEHFVDHRIVIPEESDPLSGQKWRDMRSTLSPVFTSSKLKYMFSLISERGEQFAQSLLKENKDVITLDIKNSFTRYTNDVMASTMFGITCDSLQEPKNEFYSLAQKSQDFSGFWKNVKLLGYFLFPEFCKSLEISFYSKEVNSFFEKLVTENIGSRQKHGIVRPDLIHLLMEAKNHAKKSTGYQESNKMKTEITDQDIATQALIFFFGGFDTVASLMSFMSYELATNPDCQEKLRQEVDEVMQNCNGKVTYEAIVNMKYMDMVTSETLRKWPNAPAVDRVCTKPYTIEPARPDEKPIHLKENDTVLLPIYALHRDPKYFPNPDRFEPERFSEENKANIEPYTYMPFGSGPRNCIGQRFALIETKLFFFYILANFELIPVERTQIPLKLTKNPFTMTAEKGFWLGFKKR

>Tc CYP9AA1

MWWLLIAAFTLLYALYIRLKKKNEYWIKKGVKQGSPVLIFGDKWRAIAHNESLADMVQKIYNVGPGDRYCGAYDFMSPALVLKDPELIKDILVKDFDHFVDHKRVIPEGSDPIWDKNLFFLTGQKWRDMRSTLSPVFTGSKMRFMFNLIAKSADQFVQHFVKQEETLIQIEMKDACARLTNDIIASTVFGFESDSVEDPDSKFYEMARKITDFSGLWQGLKILGFFIFPKYFENQNLSTEISNFFYNIVKENIRSRQLQNISRPDMISLLMEARENHKLIHGVEISDEDVTAQALVFFFAGFDSVSSLMTFMSYELALNPEIQKKLIEEIDTTTKTLDYDVVKSMKYLDMVVSETLRKWPITPFVNRVCTIPYTVQPKRPDENPIHLKKDAIVFLPIYGLHHDPKYFKDPERFDPERFSDENKAKIVPYTYVPFGAGPRKCIGYRFALLEIKILFFFLLSKFEIVTVDRTEIPVKICKKALNMTPEGGLWLGLKPRK

>Tc CYP9Z6

MFWILVGLALVALYWALSRPHSYWKRKGVVQGSPVFLLGDSWPMLFRKYSPPEMTELFYKASPNTRYFGIYEFLTPGLLLRDPDLIKQITVKDFDHFVDRRPFVPEKADPLFGKSLFSLKGPKWRAMRSTLSPVFTSSKMKYMFQLMSQTGERFINHFLNQKEEVVTVEMKEITTKFANDVIGNIVFGYECDSVKDPKNEFYVMGADLTNFRSLRFLLVFSAKSIFQLFKIPLFPKKVSDFFVRVVNDNIESREKHGIVRPDMIHLLLKMKSNNLKHEEVETVPDAGFASVEESELGKNAKTVTKITNEDIAAQALLFFFAGFDSVSSLISYMAYELAVNPDVQTKLLQEVDETRQKSDGKITYEALMSMKYMDMVVSEALRKWPNAIATERVCTKPYTIEPKLPDEKPLRLEIGDVVAIPMYAIQRDPKYFPEPERFIPERFSDENKSKVQPYTFMSFGTGPRSCIGSRFALLETKLLFYYFLTNFEFVPVEKTQIPIKFNRKALNMSPENGFWLGLKRRV

>Tc CYP9AD1

MLLLLLGTLIVILVLYLVNLSTKNFDHWEKRRVKYVKPFPFVGNLLPTVLKTKSTAELIQHLYKAFPNERFVGIFQFNTPILLIRDPELIKSIAIKNFDNFVDHYGFANDDIDPLWAKNLFASQGERWRDLRQTLSPVFTSSKMRTMFVLMDECVKQLTEYFKDQKQDVIDIELKDIFSRYTTDVIATTAFGIKVDSLRNRNNDFVVSGREFTDFSGLRGLAFFINGSYPRLAKFLNIKIVSDRLGNFFRTIIKETLTLREEKKIVRPDLIHSLMLARKGKLKYEELMELPEAGFAAVEESNLTKSNNNVNFLTDEDITAQALVFFLGGFDTTSSLMCFAGYELAINPHIQKRLKDEVLATDRECNGQITYEKLLNMKYLDMVVSETLRKWNQAVWLDRKCTKEIEIESETSGEPSVTLKVGDIIWMPAYAIHHDPKYYPNPELFDPERFSDENKDKIRTGTYLPFGVGPRNCIGSRFALLETKLLLYNLLLNFDLVTNHKTENPIKLRRDTPLLMPQNGFQVSLRKNDAFSEK

>Dm CYP6A2

MFVLIYLLIAISSLLAYLYHRNFNYWNRRGVPHDAPHPLYGNMVGFRKNRVMHDFFYDYYNKYRKSGFPFVGFYFLHKPAAFIVDTQLAKNILIKDFSNFADRGQFHNGRDDPLTQHLFNLDGKKWKDMRQRLTPTFTSGKMKFMFPTVIKVSEEFVKVITEQVPAAQNGAVLEIKELMARFTTDVIGTCAFGIECNTLRTPVSDFRTMGQKVFTDMRHGKLLTMFVFSFPKLASRLRMRMMPEDVHQFFMRLVNDTIALRERENFKRNDFMNLLIELKQKGRVTLDNGEVIEGMDIGELAAQVFVFYVAGFETSSSTMSYCLYELAQNQDIQDRLRNEIQTVLEEQEGQLTYESIKAMTYLNQVISETLRLYTLVPHLERKALNDYVVPGHEKLVIEKGTQVIIPACAYHRDEDLYPNPETFDPERFSPEKVAARESVEWLPFGDGPRNCIGMRFGQMQARIGLAQIISRFRVSVCDTTEIPLKYSPMSIVLGTVGGIYLRVERI

>Dm CYP6A8

MALTYILFQVAVALLAILTYYIHRKLTYFKRRGIPFVAPHLIRGNMEELQKTKNIHEIFQDHYNKFRESKAPFVGFFFFQSPAAFVIDLELAKQILIKDFSNFSNKGIFYNEKDDPISAHLFNLDGAQWRLLRNKLSSTFTSGKMKLMYPTVVSVANEFMTVMHEKVPKNSVLEIRDLVARFTVDVIGTCAFGIQCNSLRDEKAEFLYFGKRSLVDKRHGTLLNGFMRSYPKLARKLGMVRTAPHIQEFYSRIVTETVAVREKEHIKRNDFMDMLIELKNQKEMTLENGDVVRGLTMEEVLAQAFVFFIAGFETSSSTMGFALYELAKNPDIQDKVRAEVEEVIEQHDQNFTYECTKDLKYLNQVLDETLRLYTIVPNLDRMAAKRYVVPGHPNFVIEAGQSVIIPSSAIHHDPSIYPEPFEFRPERFSPEESAGRPSVAWLPFGDGPRNCIGLRFGQMQARIGLALLIRNFKFSTCSKTPNPLVYDPKSFVLGVKDGIYLKVETV

>Dm CYP6A9

MGVYSVLLAIVVVLVGYLLLKWRRALHYWQNLDIPCEEPHILMGSLTGVQTSRSFSAIWMDYYNKFRGTGPFAGFYWFQRPGILVLDISLAKLILIKEFNKFTDRGFYHNTEDDPLSGQLFLLDGQKWKSMRSKLSYTFTSGKMKYMFPTVVKVGHEFIEVFGQAMEKSPIVEVRDILARFTTDVIGTCAFGIECSSLKDPEAEFRVMGRRAIFEQRHGPIGIAFINSFQNLARRLHMKITLEEAEHFFLRIVRETVAFREKNNIRRNDFMDQLIDLKNSPLTKSESGESVNLTIEEMAAQAFVFFGAGFETSSTTMGFALYELAQHQDIQDRVRKECQEVIGKYNGEITYESMKDMVYLDQVISETLRLYTVLPVLNRECLEDYEVPGHPKYVIKKGMPVLIPCGAMHRDEKLYANPNTFNPDNFSPERVKERDSVEWLPFGDGPRNCIGMRFGQMQARSGLALLINRFKFSVCEQTTIPIVYSKKTFLISSETGIFLKVERV

>Dm CYP6A13

MLTLLVLVFTVGLLLYVKLRWHYSYWSRRGVAGERPVYFRGNMSGLGRDLHWTDINLRIYRKFRGVERYCGYFTFMTKSLFIMDLELIRDIMIRDFSSFADRGLFHNVRDDPLTGNLLFLDGPEWRWLRQNLTQVFTSGKMKFMFPNMVEVGEKLTQACRLQVGEIEAKDLCARFTTDVIGSCAFGLECNSLQDPESQFRRMGRSVTQEPLHSVLVQAFMFAQPELARKLRFRLFRPEVSEFFLDTVRQTLDYRRRENIHRNDLIQLLMELGEEGVKDALSFEQIAAQALVFFLAGFDTSSTTMSFCLYELALNPDVQERLRVEVLAVLKRNNQKLTYDSVQEMPYLDQVVAETLRKYPILPHLLRRSTKEYQIPNSNLILEPGSKIIIPVHSIHHDPELYPDPEKFDPSRFEPEEIKARHPFAYLPFGEGPRNCIGERFGKLQVKVGLVYLLRDFKFSRSEKTQIPLKFSSRNFLISTQEGVHLRMEGLERP

>Dm CYP6A14

MLFTIALVGVVLGLAYSLHIKIFSYWKRKGVPHETPLPIVGNMRGIVKKYHFRDINQRIYKKFKGQGPIAGMYTFFKRTALITDLDFIKQVMIKDFSYFQDRGAFTNPRDDPLTGHLFALEGEEWRAMRHKLTPVFTSGKIKQMSKVIVDVGLRLGDAMDKAVKEAKVEEGNVEIKDLCARFTTDVIGSCAFGLECNSLQDPSAEFRQKGREIFTRRRHSTLVQSFIFTNARLARKLRIKVLPDDLTQFFMSTVKNTVDYRLKNGIKRNDFIEQMIELRAEDQEAAKKGQGIDLSHGLTLEQMAAQAFVFFVAGFETSSSTMSLCLYELALQPDIQQRLREEIESVLANVDGGELNYDVLAQMTYLDQVLSETLRKHPLLPHLIRETTKDYQIPNSDIVLDKGILALIPVHNIHHDPEIYPEPEKFDPSRFDPEEVKNRHPMAYLPFGDGPRNCIGLRFGKIQAKIGLVSLLRRFKFSVSNRTDVPLIFSKKSFLLTTNDGIYLKVERV

>Dm CYP6A16

MDFTLLLLTSLLSFLLGYLRYRFTYWELRGIPQLRPHFLFGHFFRLQSVHYSELLQETYDAFRGSAKVAGTYVFLRPMAVVLDLDLVKAVLIRDFNNFVDRRSFHGDPLTANLFNLQGEEWRNLRTKLSPTFTSGKMKYMFGTVSTVAQQLGGTFDELVGSQGAVLELHDLMARYTTDVIGSCAFGTECSSLREPQAEFRQVGRRIFRNSNRSIRWRIFKMTYLSSLAKLGLPVRILHPDITKFFNRIVRETVELRERENIRRNDFMDLLLDLRRKGLTMEQMAAQAFVFFVAGFETSSSNMSYALFELAKNQDVQQKLRMEINDSIGKHGKLTYEAMMEMPYLDQTITETLRKYPALSSLTRLASEDYEIPSPDGGDPVVLEKGTSVHIPVLAIHYDPEVYPEPHEFRPERFAPDACRERHPTAFLGFGDGPRNCIGLRFGRMQVKVGLITLLRRFRFSLPPGSPTQLKVTKRNLILLPSDGVRLQVDPVESRLM

>Dm CYP6A17

MLLLALIVVILSLLVFAARRRHGYWQRRGIPHDEVHPLFGNIKDWPNKRHIAEIFRDYYFKYKNSDYPFAGFFFFFTRTAVVTDMELLKRVLIKDFNHFENRGVFYNEIDDPLSATLFSIEGQKWRHLRHKLTPTFTSGKMKNMFPIVVKVGEEMDKVFRSKTAADRGQVLEVVDLVARYTADVIGNCAFGLNCNSLYDPKAEFVSIGKRAITEHRYGNMLDIFLFGFPKLSRRLRLKLNIQEAEDFYTKIVRETIDYRLRTKEKRNDFMDSLIEMYKNEQSGNSEDGLTFNELLAQAFIFFVAGFETSSTTMGFALYELARNQDVQDKLREEIGNVFGKHNKEFTYEGIKEMKYLEQVVMETLRKYPVLAHLTRMTDTDFSPEDPKYFIAKGTIVVIPALGIHYDPDIYPEPEIFKPERFTDEEIAARPSCTWLPFGEGPRNCIGLRFGMMQTCVGLAYLIRGYKFSVSPETQIPMKIVVKNILISAENGIHLKVEKLAK

>Dm CYP6A18

MQLTYFLFQVAVALLAIVTYILHRKLTYFKRRGIPYDKPHPLRGNMEGYKKTRTVHEIHQEYYNKYRNSKAPFVGFYLFQKPAAFVIDLELAKQILIKNFSNFTDKGIYYNEKDDPMSAHLFNLDGPQWRLLRSKLSSTFTSGKMKFMYPTVVSVAEEFMAVMHEKVSENSILDVRDLVARFTVDVIGTCAFGIKCNSLRDEKAEFLHFGRRALLDSRHGNLVSGLMRSYPNLARRLGLCRNTAQIQEFYQRIVKETVTLREKENIKRNDFMDMLIGLKNQKNMTLENGEVVKGLTMDEIVAQAFVFFIAGFDTSSSTMGFALYELAKNPSIQDKVRAELGQVLEQHDQKFTYECIKDLKYLDQVINETLRHYTIVPNVDRVAAKRFVVPGNPKFVIEAGQSVIIPSSAIHHDPSIYPEPNEFRPERFSPEESAKRPSVAWLPFGEGPRNCIGLRFGQMQARIGLAMLIKNFTFSPCSATPDPLTFDPHSAILLGIKGGIQLKVEAI

>Dm CYP6A19

MAILLGLVVGVLTLVAWWVLQNYTYWKRRGIPHDPPNIPLGNTGELWRTMPLAGILKRTYLKFRKQTDGPFAGFYLYAMKYIVITDVDFVKTVLIRDFDKFHDRGVYHNEKDDPLTNNLATIEGQKWKNLRQKLTHTFTSAKMKSMFSTVLNVGDEMIRVVDEKISSSSQTLEVTDIVSRFTSDVIGICAFGLKCNSLRDPKAEFVQMGYSALRERRHGWLVDLLIFGMPKLAVKLGFQFLLPSVQKFYMKIVQDTIDYRMKRKVTRNDFMDTLIDMKQQYDKGDKENGLAFNEVAAQAFVFFLAGFEAGSTTMGFTLYELACNQDVQDKLRAEIDSVLERYNGKLEYDSMQDLFYMEKVINESLRKHPVVAHLARIATKPYQHSNPKYFIEAGTGVLVSTLGIHHDPEFYPEPEKFIPERFDEEQVKKRPTCAFLPFGAGPRNCIGLRFGRMQVIIGLALLIHNFRFELHPKTPVPMKYTINNLLLGSEGGIHLNITKVVRD

>Dm CYP6A20

MAVMIVLLIGVITFVAWYVHQHFNYWKRRGIPHDEPKIPYGNTSELMKTVHFADIFKRTYNKLRNKTDGPFVGFYMYFKRMVVVTDIDFAKTVLIREFDKFHDRGVFHNERDDPLSANLVNIDGQKWKTLRQKLTPTFTSGKMKTMFPTILTVGDELIRVFGETASADSDSMEITNVVARFTADVIGSCAFGLDCHSLSDPKAKFVQMGTTAITERRHGKSMDLLLFGAPELAAKLRMKATVQEVEDFYMNIIRDTVDYRVKNNVKRHDFVDMLIEMKLKFDNGDKENGLTFNEIAAQAFIFFLAGFETSSTTMGFALYELACHQDIQDKLRTEINTVLKQHNGKLDYDSMREMTYLEKVIDETMRKRPVVGHLIRVATQHYQHTNPKYNIEKGTGVIVPTLAIHHDPEFYPEPEKFIPERFDEDQVQQRPACTFLPFGDGPRNCIGLRFGRMQVIVGMALLIHNFKFEFHPTKTVVPLEYRTDDFLLSSKGGIHLKVTRV

>Dm CYP6A23

MSLLLTLIALLVSLLLFMARRRHGYWQRRGIPHDVPHPIYGNMKDWPKKRHIAMIFRDYYTKYKRSVYPFAGFYFFFTRSAVITDLELVKRVLIKDFNHFENRGIFYNEIDDPLSATLFSIEGQKWRHLRHKLTPTFTSGKMKNMFPIIVKVGEEMEKIFSAKTTTGEGQVLEIVDLVARYTADVIGNCAFGLNCNSLQNPNAEFVTIGKRAIIERRYGGLLDFLIFGFPKLSRRLRLKLNVQDVEDFYTSIVRNTIDYRLRTNEKRHDFMDSLIEMYEKEQAGNTEDGLSFNEILAQAFIFFVAGFETSSTTMGFALYELALDQDIQDQLRAEINNVLSKHNNEFTYEGIKEMKYLEQVVMETLRKYPVLAHLTRMTQTDFSPEDPKYFIAKGTTVVIPALGIHYDPEIYPEPEKFKPERFTDEAIAARPSCTWLPFGEGPRNCIGLRFGLMQACVGLAYLIRGYKFSVSTETQIPMKFVVKSILLSAENGIHLKVEKLSK

>Dm CYP6D2

MWTILLTILIAGLLYRYVKRHYTHWQRLGVDEEPAKIPFGVMDTVMKQERSLGMALADIYARHEGKIVGIYMLNKRSILIRDAQLARQIMTSDFASFHDRGVYVDEDKDPLSANLFNLRGASWRNLRQKLTPSFSSGKIKGMFGTIDDVGDKLVQHLEGALDQSDEVEIKDVMTTYAVDIIGSVIFGLEIDSFRNPKNEFREISSSTSRDESLLLKIHNMSMFICPPIAKLMNRLGYESRILTSLRDMMKRTIEFREEHNVVRKDMLQLLIRLRNTGKIGEDDDQVWDMETAQEQLKSMSIEKIAAQAFLFYVAGSESTAAASAFTLYELSMYPELLKEAQEEVDAVLMKHNLKPKDRFTYEAVQDLKFLDICIMETIRKYPGLPFLNRECTEDYPVPGTNHIIAKGTPILISLFGMQRDPVYFPNPNGYDPHRFDSNNMNYDQAAYMPFGEGPRHCIALRMGKVNSKVAVAKILANFDLVQSPRKEVEFRFDAAPVLVTKEPLKLRLTKRK

>Dm CYP6D4

MFSLILLAVTLLTLAWFYLKRHYEYWERRGFPFEKHSGIPFGCLDSVWRQEKSMGLAIYDVYVKSKERVLGIYLLFRPAVLIRDADLARRVLAQDFASFHDRGVYVDEERDPLSANIFSLRGQSWRSMRHMLSPCFTSGKLKSMFSTSEDIGDKMVAHLQKELPEEGFKEVDIKKVMQNYAIDIIASTIFGLDVNSFENPDNKFRKLVSLARANNRFNAMFGMMIFLVPSIAQFLFRIGFKNPVGLAMLQIVKETVEYREKHGIVRKDLLQLLIQLRNTGKIDENDEKSFSIQKTPDGHIKTISLEAITAQAFIFYIAGQETTGSTAAFTIYELAQYPELLKRLQDEVDETLAKNDGKITYDSLNKMEFLDLCVQETIRKYPGLPILNRECTQDYTVPDTNHVIPKGTPVVISLYGIHHDAEYFPDPETYDPERFSEESRNYNPTAFMPFGEGPRICIAQRMGRINSKLAIIKILQNFNVEVMSRSEIEFENSGIALIPKHGVRVRLSKRVPKLS

>Dm CYP6D5

MIGIYLLIAAVTLLYVYLKWTFSYWDRKGFPSTGVSIPFGALESVTKGKRSFGMAIYDMYKSTKEPVIGLYLTLRPALLVRDAQLAHDVLVKDFASFHDRGVYVDEKNDPMSASLFQMEGASWRALRNKLTPSFTSGKLKAMFETSDSVGDKLVDSIRKQLPANGAKELELKKLMATYAIDIIATTIFGLDVDSFADPNNEFQIISKKVNRNNIEDIIRGTSSFLYPGLEKFFVKIGWKQEATERMRELSNRTVDLREQNNIVRKDLLQLLLQLRNQGKINTDDNIWSAESTKNGVKSMSKDLIAGQLFLFYVAGYETTASTTSFTLYELTQNPEVMEKAKEDVRSAIEKHGGKLTYDAISDMKYLEACILETARKYPALPLLNRICTKDYPVPDSKLVIQKGTPIIISLIGMHRDEEYFPDPLAYKPERYLENGKDYTQAAYLPFGEGPRMCIGARMGKVNVKIAIAKVLSNFDLEIRKEKCEIEFGVYGIPLMPKSGVPVRLSLKK

>Dm CYP6G1

MVLTEVLFVVVAALVALYTWFQRNHSYWQRKGIPYIPPTPIIGNTKVVFKMENSFGMHLSEIYNDPRLKDEAVVGIYSMNKPGLIIRDIELIKSILIKDFNRFHNRYARCDPHGDPLGYNNLFFVRDAHWKGIRTKLTPVFTSGKVKQMYTLMQEIGKDLELALQRRGEKNSGSFITEIKEICAQFSTDSIATIAFGIRANSLENPNAEFRNYGRKMFTFTVARAKDFFVAFFLPKLVSLMRIQFFTADFSHFMRSTIGHVMEERERSGLLRNDLIDVLVSLRKEAAAEPSKPHYAKNQDFLVAQAGVFFTAGFETSSSTMSFALYEMAKHPEMQKRLRDEINEALVEGGGSLSYEKIQSLEYLAMVVDEVLRMYPVLPFLDREYESVEGQPDLSLKPFYDYTLENGTPVFIPIYALHHDPKYWTNPSQFDPERFSPANRKNIVAMAYQPFGSGPHNCIGSRIGLLQSKLGLVSLLKNHSVRNCEATMKDMKFDPKGFVLQADGGIHLEIVNDRLYDQSAPSLQ

>Dm CYP6G2

MELVLLILVASLIGIAFLALQQHYSYWRRMGVREIRPKWIVGNLMGLLNMRMSPAEFISQLYNHPDAENEPFVGIHVFHKPALLLRDPEMVRNILVKDFAGFSNRYSSSDPKGDPLGSQNIFFLKNPAWKEVRLKLSPFFTGNRLKQMFPLIEEVGASLDAHLRQQPLHNERMRCFDLEAKELCALYTTDVIATVAYGVSANSFTDPKCEFRRHGRSVFEFNLLRAAEFTLVFFLPHLVPFVRFKVVPAEATRFLRKTINYVMSEREKSGQKRNDLIDILIEFRRSTQLAKASGIKDQFVFEGDILVAQAVLFFTAGFESSSSTMAFAMYELAKDTDVQQRLREEIKDALVESGGQVTLKMIESLEFMQMILLEVLRMYPPLPFLDRECTSGRDYSLAPFHKKFVVPKGMPVYIPCYALHMDPQYFPQPRKFLPERFSPENRKLHTPYTYMPFGLGPHGCIGERFGYLQAKVGLVNLLRNHMITTSERTPHRMQLDPKAIITQAKGGIHLRLVRDALGV

>Dm CYP6T1

MIAVFSLIAAALAVGSLVLLPVVLRGGCLLVVTIVWLWQILHFWHWRRLGVPFVPAAPFVGNVWNLLRGACCFGDQFRELYESKEAAGRAFVGIDVLHNHALLLRDPALIKRIMVEDFAQFSSRFETTDPTCDTMGSQNLFFSKYETWRETHKIFAPFFAAGKVRNMYGLLENIGQKLEEHMEQKLSGRDSMELEVKQLCALFTTDIIASLAFGIEAHSLQNPEAEFRRMCIEVNDPRPKRLLHLFTMFFFPRLSHRVGTHLYSEEYERFMRKSMDYVLSQRAESGENRHDLIDIFLQLKRTEPAESIIHRPDFFAAQAAFLLLAGFDTSSSTITFALYELAKNTTIQDRLRTELRAALQSSQDRQLSCDTVTGLVYLRQVVDEVLRLYPPTAFLDRCCNSRTGYDLSPWNGGSPFKLRAGTPVYISVLGIHRDAQYWPNPEVFDPERFSAEQRQQHHPMTYLPFGAGPRGCIGTLLGQLEIKVGLLHILNHFRVEVCERTLPEMRFDPKAFVLTAHNGTYLRFVKNSL

>Dm CYP6T3

MLLIWLLLLTIVTLNFWLRHKYDYFRSRGIPHLPPSSWSPMGNLGQLLFLRISFGDLFRQLYADPRNGQAKIVGFFIFQTPALMVRDPELIRQVLIKNFNNFLNRFESADAGDPMGALTLPLAKYHHWKESRQCMSQLFTSGRMRDVMYSQMLDVASDLEQYLNRKLGDRLERVLPLGRMCQLYTTDVTGNLFYSLNVGGLRRGRSELITKTKELFNTNPRKVLDFMSVFFLPKWTGVLKPKVFTEDYARYMRHLVDDHHEPTKGDLINQLQHFQLSRSSNHYSQHPDFVASQAGIILLAGFETSSALMGFTLYELAKAPDIQERLRSELREAFISTATLSYDTLMTLPYLKMVCLEALRLYPAAAFVNRECTSSASEGFSLQPHVDFIVPPGMPAYISILGLHRDERFWPEPCVFDPERFGPERSRHIHPMTYIPFGAGPHGCIGSRLGVLQLKLGIVHILKQYWVETCERTVSEIRFNPKSFMLESENEIYLRFCRSSL

>Dm CYP9B1

MSFVEICLVLATIGLLLFKWSTGTFKAFEGRNLYFEKPYPFLGNMAASALQKASFQKQISEFYNRTRHHKLVGLFNLRTPMIQINDPQLIKKICVKDFDHFPNHQTLNIPNERLVNDMLNVMRDQHWRNMRSVLTPVFTSAKMRNMFTLMNESFAQCLEHLKSSQPIAAGENAFELDMKVLCNKLSNDVIATTAFGLKVNSFDDPENEFHTIGKTLAFSRGLPFLKFMMCLLAPKVFNFFKLTIFDSTNVEYFVRLVVDAMQYREKHNITRPDMIQLLMEAKKESKDNWTDDEIVAQCFIFFFAAFENNSNLICTTAYELLRNLDIQERLYEEVKETQEALKGAPLTYDAAQEMTYMDMVISESLRKWTLSAAADRLCAKDYTLTDDEGTKLFEFKAGDNINIPICGLHWDERFFPQPQRFDPERFSERRKKDLIPYTYLPFGVGPRSCIGNRYAVMQAKGMLYNLMLNYKIEASPRTTRDMWESARGFNIIPTTGFWMQLVSRK

>Dm CYP9B2

MALIEICLALVVIGYLIYKWSTATFKTFEERKLYFEKPYPFVGNMAAAALQKSSFQRQLTEFYERTRQHKLVGFFNMRTPMITLNDPELIKKVCVKDFDHFPNHQPFITSNDRLFNDMLSVMRDQRWKHMRNTLTPVFTAAKMRNMFTLMNESFAECLQHLDSSSKTLPGRKGFEVDMKVMCNKLSNDIIATTAFGLKVNSYDNPKNEFYEIGQSLVFSRGLQFFKFMLSTLVPKLFSLLKLTIFDSAKVDYFARLVVEAMQYREKHNITRPDMIQLLMEAKNESEDKWTDDEIVAQCFIFFFAAFENNSNLICTTTYELLYNPDVQERLYEEIVETKKALNGAPLTYDAVQKMTYMDMVISESLRKWTLAAATDRLCSKDYTLTDDDGTKLFDFKVGDRINIPISGLHLDDRYFPEPRKFDPDRFSEERKGDMVPYTYLPFGVGPRNCIGNRYALMQVKGMLFNLLLHYKIEASPRTIKDLWGSASGFNFTPRSGFWMHLVPRK

>Dm CYP9C1

MVFVELSIFVAFIGLLLYKWSVYTFGYFSKRGVAHEKPIPLLGNIPWSVLMGKESYIKHSIDLHLRLKQHKVYGVFNLRDPLYYLSDPELIRQVGIKNFDTFTNHRKGITEGFNDTSVISKSLLSLRDRRWKQMRSTLTPTFTSLKIRQMFELIHFCNVEAVDFVQRQLDAGTSELELKDFFTRYTNDVIATAAFGIQVNSFKDPNNEFFSIGQRISEFTFWGGLKVMLYILMPKLMKALRVPVMDMNNVDYFKKLVFGAMKYRKEQSIVRPDMIHLLMEAQRQFKAEQEGSAESAAQQDKAEFNDDDLLAQCLLFFSAGFETVATCLSFTSYELMMNPEVQEKLLAEILAVKEQLGEKPLDYDTLMGMKYLNCVVSESLRKWPPAFIVDRMCGSDFQLKDEEGEVVVNLREDDLVHINVGALHHDPDNFPEPEQFRPERFDEEHKHEIRQFTYLPFGVGQRSCIGNRLALMEVKSLIFQLVLRYHLKPTDRTPADMMSSISGFRLLPRELFWCKLESRGPA

>Dm CYP9F2

MLWEFFALFAIAAALFYRWASANNDFFKDRGIAYEKPVLYFGNMAGMFLRKRAMFDIVCDLYTKGGSKKFFGIFEQRQPLLMVRDPDLIKQITIKDFDHFINHRNVFATSSDDDPHDMSNLFGSSLFSMRDARWKDMRSTLSPAFTGSKMRQMFQLMNQVAKEAVDCLKQDDSRVQENELDMKDYCTRFTNDVIASTAFGLQVNSFKDRENTFYQMGKKLTTFTFLQSMKFMLFFALKGLNKILKVELFDRKSTQYFVRLVLDAMKYRQEHNIVRPDMINMLMEARGIIQTEKTKASAVREWSDRDIVAQCFVFFFAGFETSAVLMCFTAHELMENQDVQQRLYEEVQQVDQDLEGKELTYEAIMGMKYLDQVVNEVLRKWPAAIAVDRECNKDITFDVDGQKVEVKKGDVIWLPTCGFHRDPKYFENPMKFDPERFSDENKESIQPFTYFPFGLGQRNCIGSRFALLEAKAVIYYLLKDYRFAPAKKSCIPLELITSGFQLSPKGGFWIKLVQRN

>Dm CYP9F3

MLWEFLALFAIATALFYRWASANNDFFKDRGIAYEKPVLYLGNMAGMFLRKRAMLDIVCDLYTKGGSGKFFGIFAQRQPLLMVRDPDLIKQITIKDFDHFINHRNEFDTSSDDDPHDMSNLFSSSLFSMRDARWKDMRSTLSPAFTGSKMRQMFQLMNQVAKEAVDCLKQDDSRVQENELDMKDYCTRFTNDVIASTAFGLQVNSFKDRENTFYQMGKKLTTFTFLQNMKFILLFALKSLNKILKVEIFDRKSTQYFVRLVLDAMKYRQEHNIVRPDMINMLMEARGIIQTEKTKASAVREWSDRDIVAQCFVFFFAGFETSAVLMCFTAHELMENQDVQQRLYEEVQQVDQDLEGKELKYLDQVVSEVLRKWPPAIAFDRECNKEAKAVIYYLLKDYRFAPAKKSCIPLELISSGFQLSPKGGFWIKLVQRN

>Dm CYP9H1

MDQSMIALALFIILLVLLYKWSVAKYDVFSERGVSHEKPWPLIGNIPLKAMIGGMPVLKKMIELHTKHTGSPVYGIYALRDAVFFVRDPELIKLIGIKEFDHFVNHNSMHNNIQESILSKSLISLRDGRWKEMRNILTPAFTGSKMRIMYDLIQSCSEEGVIHIQEQLELSQDASIELEMKDYFTRFANDVIATVAFGISINSFRRKDNEFFRIGQAMSRISAWSVVKAMLYALFPRLMKVLRIQVLDTKNIDYFSSLVTAAMRYRQEHKVVRPDMIHLLMEAKQQRLADLSDKSKDELYYSEFTADDLLAQCLLFFFAGFEIISSSLCFLTHELCLNPTVQDRLYEEIISVHEELKGQPLTYDKLTKMKYLDMVVLEALRKWPPSISTDRECRQDIDLFDENGQKLFSARKGDVLQIPIFSLHHDPENFEDPEFFNPERFADGHALESRVYMPFGVGPRNCIGNRMALMELKSIVYQLLLNFKLLPAKRTSRDLLNDIRGHGLKPKNGFWLKFEARQ

>Ld CYP345F2

LLLLTVSIYLVYRYLTRNHTYWKDRGVPFEEPVFLAGNLWDVFTGKRQIGKHLGHLYSKYPRDTPYFGIYIMGRPYLVLRNPEIIKSVTIRDFSNFDDRTFACDVNADPMTGNSLFIMRNPDWKNIRNKLTPIFTSGKLKMMINIMKKCSKEMQTYLGGFDGQVVEVKEVAAKYMTDL

>Ld CYP6BQ16

MTFLLTVTYILGAVIVGIYLLLKWIYSYWYRKGVEYIEPDFFYGNVKEMIQRKVSLGGLFKKFYDNLKSRGLKYGGCYTFFSPVFIPVDLDVIKSILLKDFDHFVNRGMYYNEKVDPLSTHLFTLEDERWKSLRSKLTPTFSSGKLKMMFPTLVSCSFGLENVLNEYSAIQDAVDIKEVSSRFSTDAIGSVAFGIDCNSLKNPNSEFRQWGRQMFLGGIRGIIKAIMLMTLPNSFLHVIRFKMTSKSTEDFLMNVVRDTVDYREKNNVYRKDFMHLLLQLK

>Ld CYP6BQ15

MDIFTIACILSVFVLAIWLFSKWSFSYWKKVGLEYPQPEFLFGNTKGFLTRKLSFGDQMKVIYDELKSRGLKHGGFYMSFRPSYLPIDLDLIKTIMQKDFNHFVNHGLYFNEKSDPLSGHLFNLEDEKWRNLRARLTPTFTSGKIKMMFQTLVSCTSGLGNILTDHSIIGDAIDIKDVVSRFTTDVIGSVAFGIDCNSLKDPDSEFRHWGKRIFTFDFMRRIKNNITMLIPRDIVIKTGIKLMSRDLEDFFMNVVRSTVQFRETHNVHRKDFMHLLLQLKNKGQIAEDDSTDKEIEIKAPGQLLTFNEIAAQCFVFFLAGFETSATTMTFALLELALNQDVQKKLREEINTVLDASDGEVTYEAIMKMSYLDKVVHETLRKHSPVPGTPRVCNKAYKVPGTDIVLEVGTRVHIPFQAIHWDPEYYPEPQKFDPERFSEENKSKRHPFAFLPFGEGPRICIGARFGLLQVKVGLTAIIRNFKVTLNKKTKTPIKYATNVFITSVDGDVWLNVEK

>Ld CYP6BQ18

MMLSLTITYILAAVVASIYFFQKWIYSYWERRGVKYIEPEFFYGNVRDMIQTKIFSGDLFKRFYDDLKSRKVKYGGIYAFFTPVFIPIDLDLIKSIMLKDFHHFVNRGGYVNEEADPLSGHLFSLEDEKWRNLRTKLTPTFTSGKLK

>Ld CYP6BQ17

CIHDTRIIMTFLLTATFILGAVIAGVYFFIKRIYSYWCRKGVEYIEPDFFYGNIKEMVHGKISTGGLFKKFYDDLKSRGLKYGGCYTFFSPVLILVDLDLIKNILLKDFDNFINRGMYYNEKMDPLSAHLFSLEDERWKNLRAKLTPTFTSGKLKMMFPTLVACSFGLEDVLSEYSVTQDAVDIKEVCSRYATDAIGSVAFGIDCKSLKNPDSEFRQWGRQMFKEGIKGIIKA

>Ld CYP9E2

MIYNFLGEKWRKMRSILSPSFTSSKMRIMFVLISKCTENFVNHFLKKDEKCIEVEMKDTFTRFTNDVIATSAFGVEVDSLEEPNNEFYSKGKKSTTFEGVSKLFMFIRYPVPKLIEYLQIPFMDPVVYRFFCDLVDNTIEVREKNNIIRPDMIHLLMEARKRGPHKKENSGVDTGFATVEKANFGAGHASELTNLDITAQAVLFFFAGFDTVSSLMCFMSHELAVHPDIQTKLRDEIEETLAECGGEVTYEAILKMKYMDMVISGEKLISFFPGAGHASELTNLDITAQAVLFFFAGFDTVSSLMCFMSHELAVHPDIQTKLRDEIEETLAECGGEVTYEAILKMKYMDMVISESLRKWPSTISTDRVCTKPYTIEAKNAGEKPLHIEKGTLLMIPSVGIHYDPKYYPDPERFDPERFSDENKRKIDPFTYMPFGLGPRLCIGSRFALLETKLLFFHLLSHFELVPVEKTQIPLKLSKFTFNLMAENGFWLGLKRLKK

>Ld CYP6A13

MELLIFDYLTKTLVYSSILATFIYLYFSWCYGHWKRRNFPFLKPNFPKGNNPTYLARFPGPHLECRDHYLEIRKRGLKFAGIFSVIHPILVVVDPVIIKDILVKDFEHFVDRGFYSSENDPLSVSLLSMGGDKWKNSRGKLTNFFTIGKVKMLIPLMVGSAKPMVRSIGECALNNTDSNITLLTKRYAAETVGTCFLGIDCNNFGDAEDTFANMVGKFFRDQNFTHLLRVSCTNRFPALSRKVGLKLILSEVSEFFKSAVRDTLKYREEHNYFRPDVLQLLINLKKEMENTSDPITEEQVAAHIFSFFVAGIDTIADLISFTLYELCRNPDIQERVREEIMDIMKKHKNEFTPECLNDMKYLRQVISETLRIYPGLPSLERECVKDYKINGTNLTIEKGTTVWISIMGIHRDPEYHTDPEKFDPDRFIEDSAKSYSQFYFPFGLGPRMCIGVRFGLTVAQIGLIKILREHRMSLSDETCTPLKLNKQSFLLETLEPLLMRAEKI

>Ld CYP6A2

MEIYFLNLLLDLIIYASILLLVVYTYFSICFNYWRDRKFPYLIPKFPLGNYQNVLCKYPGATFENCNIYEEIRRKKHKFAGIFSITRPILVIADPEIAKDILVKDFDHFVDRGFYKTTHDPLSYTMFTMDGADWKKSRMAFSPSFTSGRVKAMIPLISSVSELMIKEIGDLATENADLDIKKLTTKFTINSLASCALGIEPGNSSNTDIFAKMARKQFHCENFTNSFRFTVLNKFPDFCSKLGLHLMDREVGMFFETILQDSIKYRKENNCTRSDMLELMVNLKKDTKLSSRPVTDNQLTAELLNFFIAALDTSPNTISFTLFELARNPDIQERVREEIEEVLIGNDGVLNTDCLKQMKYLLQVINETLRIYPPLPNVERKCSKDYKISGTDLTIERGTSIIIPIYGIQRDPENFSIPEKFDPERFSDQNKVNMKSYTFLPFGIGPRSCVGLRFGLTMVQIAVIDIIRRFKLSVSPTTKLPFEMDNRAFMLTVKCPILLKAEIIQNSAMQ

>Ap CYP9E2

MLNVVSKAAAGALRAVKPSILQNEITKISGALSVNSRDYAKAASSKGMKPVLILRDINLIKTVLQSNFSKFHENAVKIDPKLDPLLAKNPFFCYGELWQTGRKRLTYAFSNARLKILFAAVYEVCTKFRNFLDRRLESSKKYEVELKSLFLKFTSEVVANAGLGIEGFCFEDDKVQSMFTNLDNNDFLDTFLIGIIVHFPFLTKLLRIQFLPTKHDKFFRTVVKKNLELRKSDPIPRNDFIQLMIEMEQTGEKIDEEIVAAHAVSFYLDGVETSSVTLNFIGCQLAIHQDVQEKLRKEVRSTLEKHGGVLTFEALKDMTYMNQVISESQRYFSALGFLGKICTDEFELQGSDGLNYRAKPGTELLIPICGLHKDPKYWDNPEIFDPERFSDENKQRIEKMAFIPFGEGPRICVGMRMAMLQMKSCLATLMKDYKLEVSPKMQLPLKLSPTYFLSAPLGGGWVLISKA

>Ap CYP6A13

IKSNILLELTDALLTSQALVFFLAGFETSSTTISNALYELAQNPEMQDKLRKEIKEVYENNGGALSYTDVKEMKYLDKVFKETLRKYPVLAALSRQATENYTFKDTKIKISKGTRIWIPVYGIQHDPNIYPEPEVFDPERFEDDAFASRHPMTYLPFGDGPRNCIGARFAHYQSKVGLITILRDNKVEVCAKTLIPYKSEPRNILMIPKGGKVELGITKV

>Ap CYP6A1

MAYIEILCGIIVSMLVFYYYFTSVFNFWKIRGIPGPKPKFLFGNIRDIILSRISTPTFIKNIYDTYTNEPMVGLYMGRNPILLLKDPELIKDVLIRDFSKFADRGFNVHEKVEPLSQHLFNLEPKRWRPLRSKLSPMFTSKKLKEMFGLILECGHHFEKYVDGLAARRQPVDFCEVAAKYTTDVIGSCAFGINMNAMSSEGSEFREAGRKIFEPTWNSIIRLKFKITMPTLYDLLGPLVPEREVTPFFIKVVTDAMKYRKERNVFRPDFIDTLMKLRDDPESLSDIELTDAFLTAQAYVFFAAGFETGASTISNTLYELAQNQGMQDRLREEIREHCDKYGGELMYENIKEMEYLDKVFKETLRKYPPGTLIPRRSVSEYTFKNTNVTIPKGTMIWIPAFPIHRDPNIYPNPDDFNPENFTEDAINNRHPMNYLAFSNGPRNCIGARFANYQVKIGLIMILRNYKVEVCEKTVIPYQFDPNLFLLGPKGGIYLRVTKVE

>Ap CYP6A14

MPSLEIIFGIILLFPAIYYYFTSTFDFWKVRGVPGPKPIPIFGNIKNVMLLKTSMCHYLKKLCEEYKHEPMIGIFTRKTPILIIQDPDLIKDVLIRDFSKFANRGIPIHEKAEPLSPHLFNLEVERWRPLRTRLSPVFTSGKLKEMFPLILDCAKHLEQYLDKLVLREEFIECRELTAKYTTDVIGSCAFGIEMNALSDEESEFRRIGRKVFSNSFGQILRFRFRQIFPRIYNLLGFVLPPMEVTKFLTNIIVSTMKYRQENNIVRPDFVNMLIELKKHPDKLENIKLTDTLLTAQAFVFFIAGFETSSSAISNALYELALNPEVQNKLRQEIKEYFNKHNELKYEYIKNMIYLDLVFRETLRKYPPGPLILRKSITNYTFNNTKVSIPEESFVWIPLYAIHHDPKIYPNPDAFIPERFNDDAIATRHPMHYLPFGDGPRNCIGARFAVYQSKIGLITILWNYKVEVCDKTMIPYEINPAAFLLTPKGGIYLKFTKIKNNEEILN

>Ap CYP6A17

MLHHFHILTAFVAIFLALYYYLTSKFDFWKNRGVSGPRPVPFFGNAKDVLLRKIGIGSFIAELYKRYDNEAMFGIFIGRSPNLVLRDLDLIKDVLIKDFSIFDNRGLNIPERAEPFSVNLFSVDATRWRPLRMRLSPVFTSGKLKEMFPLILECAEHLEQCLEDAVKRGGPVDCFEIPARYTTDVIGSCAFGINMNALSDERSEFRKMGRNMFDQNMIKFTRNLLRDFFPRFYNLLGFVLPYTESTVFMTKLIKGTIKYREENDVVRPDFVNLLMELKKHPEKLKNIEITDTLLAAQASVFFAAGFETSSTTMAHALYEMALNPDIQDKLRNEMKEFHAKNNGNLKYEDIKEMKYLDKVFRETLRKYPPGMLLRRKCNSNYTFHGTKVSIPAGTSVIIPLYAIQIDPKFYENPDVFDPERFNEDAVAARHPMTYLPFGDGPRNCVGARFAVYQTKVGLIKILQNFRVDVCEKTMIPYVKKINSITLAPRDGIFLKIEKITD

>Ah CYP4AA1

MDYYLGHIFQILSAILLAIKNYARIFILAWKLPGPPGLPILGNVLTLRNQDELVHLGTYSSKLYGTIMRLWVSLLPTIYIMDPRHLKIILSTNKNNQKGLFYEVLHNFIGRGLITNSGYKWKKNRKLVQPYFHINILEKFLDTFIDCSQSFVSRLEGNETVKITTYINECVLDILHNGVLGVPLDLNSPYRQGELQALERFIKPWLLLEPIFQFTSSAKYEQKQRHNLHSYTKEILTQRLKTAKKSSRTCFLDMFIEISENSPEFTEEDIINETVTFMLAGQDSVGATLAFALYYIAKNQEVQEQIINEITLMDINDRVTLKDLNNMKYLEQVIKETLRLAPAVPMITRVLTEDVALDKTILPKGTNIFISPFTTHRLEEYFHDPLKFIPDRFEENNLEKIHPYAFLPFSLGPRNCIGSKFAILELKTLLYYFLKKFEIVIPPGREDLKFSYRATLRARGGIWLNLYSKMLQFVVQCYGNLIS*

>Ah CYP4BD1

QEKVLAEQKEVFGDVQNVSPTIGQLQDMKYLDLVIKESMRLYPPVPYFGRKLPEDLEWEGNTFPKGLDVLLFCYAMQRDPEYFPDPLSFIPERFLDNSRVNPYVYCPFSAGSRNCIGQKFAMLELKSAISKIVRNYELCPAKPIQGLQLAPDTILTSKNGVHISIKHRIY*

>Ah CYP4BD4V1

YLQDVPRPKPNILFGNTLDFITKPTSEYFNVLVGYKNRFGKIYLVQDGPLSCGLIVAEAAFAEHILGSTNITKKSSQYESLQSWLGEGLLTSEGLKWKQRRRMLTSSFHFQILENFIDTFHNASDKFVKKLEKQIGKDSFDISQMVSLLTLDIICEAAMNTKINAQDNETSEYVRAVKTVCAIFSERMASPIHKWFYPLTTNFYRERAALKVLHQHTNTVIDQRIANLKNNLIKNITENPNKKLTFLDLLLKSNENGYSLTKNDIKEEVDTFMFEGHDTTSAAINFALYSMANNPEVQNKVLAEQIEILGPNKNKRPTITELQEMKYLELVIKETLRLYPPVPYFGRELLKDINWDGKIYPKGLSLMIFCYGMQRDPDYFPDPLKFNPERFLESTQINPYVYCPFSAGPRNCIGQKFAMLELKTTLSKIIRNFELNPAKPQKYLQLAPEVILVSKNGVHISL

>Ah CYP4BD4V2

MFAVIGVICLLPLLMVFIIFIKHQRFIWKNREFMRTVPGQKPNPVFGNMLDFAGPTHEFLNRIQNYRNQYGRIFRVFDRPTRAMIIVSDEKLMEYIMSSPKFIEKSYHYDYFFKWLGTGLLTSTGLKWKKRRRMLTPAFHFNILENFVDIIDKTSDILVDKLENHAGKKSFNIYPYISLYTLDVICEASMGTSVHAQTSNNSEYVKSVKTMCTILNDRNFSPLDQRLFPYTLNAWRERRALKVLHNHTDSVIDKRIQEKFTDGTQVKAFNDVGQKRRLAFLDLLLESTIDGVPLAREDIREEVDTFMFEGHDTTSSAIAFAVYCLSRYP

>Ah CYP4BN1

MYLLTFILIVFSALFSYIIIWKQINKKYMKNLSGPEPNYFLGNLAYLFFIKPEESFEQLQILNDKYGTFIKCFTGPIHFILIVFDIKFLENILSSTKLIDKSVRYSFLHRWLGTGLLTSTGKKWKTRRRLISPSFHFSILETYIEIFDNVSEIFIQKLNKEVGQSSVDLSPLTTSCTLDIICEAAMDTKLNAQDDQNLEYVRAVKTMCEIFVKRIVVPLPDILYILTPNYYKERKALKVLHNHTNNIINKKIWENKNGIKSVPESKKRLSFMDLLLQSSLSKEEIREEVDTFMFEGHDTSSVVLGFAMYCLANNPEKQEKAFQEQKAIFDTKDDLKIETVDLQAMKYLEMVIKETLRLYPPVPMLGRRFSETFEFGGNIYPKDLEVLIPIYIVHRNPEHFPEPFKFIPERFEESKIIPYTYLPFSAGPRNCIGQKFAMLEIKTILSKVIRHFELIPATPFHEIQPFPSVVLQSKNGIRVALKKRT*

>Ah CYP4BN15

GLRWKQHRRMLTPSFHFSILHNFMEVFETNGNYFIEKLKKEVGKDSVDILPLVSLMTLDVICEAAMNTKINAQQNSESEYVKSVKVMCDIFTDRFYSTLHTSLYPFTWNNIREKKALKILHSHTEGVIEKRIDEYKTKGSRRSQNDNIDDVGLKKKLVFLDMLLESTIDGKPLTKLDIREEVDTFMF

>Ah CYP4BN28

WKKNRRLLTPAFHFSVLEKFIETFDSHSEVLVELLQKEVDKSYFDISPYTAMAALDIICETTMGVCINAQRNRKSNYVNAVHLMCRVMIARTYSAIKKFDFLYRFTSMYQEQQKAIKILHTYTSDVIKQRRQELENLETDEEDIDDLGRKRRLAFLDVLLKASTDQRKLTTEEIRQEVDTFMFAGHDTTSIALSFTLYCLSQNPDVQVKAVVEQRSIFNGDFYRVATMRDLQEMKYLEMVIKEALRMYPPVPYTGRCATKDVVYKDGKIIPAGTNFLLLIFCSNRNPKYFPDPDKFNPERFLDGKSIPQYAYIPFSAGPRNCIGQKFAMLEMKTVLSKLLRHFEIVSSGQELKLISDTVLKSENGINIKLKKRKI*

>Ah CYP4BQ1

PTVPRWPIVGNIMEFTDTSTILQDFTRCIPKKEKIVVLDMAGKINILSTDYDFLEFILSNQNILKKSHDYSYLINWLGTGLLISDGDKWRTRRRIITPAFHFAILEQFVDIFEANTSVLIKKLEDSIGDSIELFPYISLYTLDVICETSMGIKLNSQMGKNRDYVKNVDLMCKLFMKRSLSIFTSNAWIYHLTPNYYKEKAVLKKLHSFTENVIQARRMLIDDTNDDLVDDLGRRKKMAFLDILLKATVDGKRLSNEDIREEVDTVMFEGHDTTASSLTFTCYCLSTNPEVQVGALITSYSPFTTYVLFFANKEIAFKEQQEIFGDDKFRPTTYRDLQEMKYLECVVKEGLRLYPSVPLYGRNVHQDIYYKGNIIPKGCNLTIFPYAIHRDPDYYPDPERFNPSRFLDIDGSRPYQYVPFSAGPRNCIGQKFAMLQMKCALSKLLRSFEILPTGGVPKLVSETVLRSSNGVHVKMVKRKW*

>Ah CYP4BR7

MLTALLVGLLVIIISKWCFQIYRKNKCLSSLPGPLPIPFFGYKLPSDAYEVTHFLEDLIKAHGNTFKIYMGNDMHVFTADPKFVDGVLTSDNLSKTQMYDFLRPFWYDGLAISEGEKWKTRRTMCSPLFHRGSLKLFFERAKNLTDNLIQNLDNAINGTDVINAKDILFQYTMEVNFESIFGLKIDSQDVNGHKFPKYVDDFVECTMARNFSAWKRFDLPYKLFASEYEEYTEALNYLRSFTLKMMNAKLNLLKLTGKESNSGNCKQCLVEHLDLPNMSTADILEEVNVFVTAGYDTSGTNVGFFLLELSRHQEMQKQIFEEITAIIGDDFSKLNLQNLQAMRYLDRFYKEVLRLRSAVPIIERRIKKDCVINGVNLPKDTSVSCLLYYLHHNKEYYPDPEKFDPDRWLPEIQSSRPSYTFIPFSTGLRNCIGQKFATYTVKISVVRMLMEYQFLPVENMKLELGIGGILMSKCGFPVRIEKRKR*

>Ah CYP4C1

MAFLGVIPIVIEVIILTFPIFWFFDIFRKVYLCHKAPGYPGRSFILGDNLSSKDAATFFKRLRKWSKTCGPVYILSASFLFPSINISGPEAFENVASTTKNITKGQVYGFLRLWLGEGLLTSTGSKWHTRRKILTPAFHFTILQQFMSVFNNETQRLVEQLKKEVDKPWSNVVPLISQFTLYSISETSMGTALNLESTEDKNYVSAVYDIGKIFYHRLLRPWLYPDAIFYSMFPTGRHEMSLVKTLQEFTNKIIRKRASTFEKFEVPTNDDSATFGKRKRLVFLDLLINAKVTNAAIDDQGIQDEVNTFMFEGHDTTAMSICFTLMLLANNKNYQDQIYQEILNVLGDNINPDLNDLSELKLMERVIKESLRLYPSVPFIGRAIEEDTIVGGYVLPRNTPVNIHIFDIHRNEKHWPDPEKFDPDRFLPENCLNRHPFAYVPFSAGPRNCIGQRFALLEIKAVLAGIIRNFILEPIDTPETIQLVPDIVLRTAGETVKVKFTLRKLD*

>Ah CYP4C3

ESFLQLPLDTMKGKNMEYVESLKTLSRLVIYKNFLLFPKFYPLSKHYRIEKKATRIIRDHAAKGIDNYNKNQPEISKNHRVMDILHRGHADGEGLSRKDIEDEVNTFMFAAHHSVAATISYVLFFLAKHPNIQEKVLDEQRNIFGKLDSSDCPNLKQLQQMHYLDLVIKESLRLLPPFPQIGRKLKKDYIYDGTLFPKDLNLILFPYSNMLSAKYFSEPTKFNPERFLTNKESESHIYVPFSAGPRSCVGQKFAIAQIKFITSKLLKEFKFLPSDPLHDMKMAFEIAMTSSNGVCVSIRKR*

>Ah CYP4C39

MILAGILAVGVILFLNWCYEIHTKNKILSKIPGPPPIPYFGYRLPSTPFGFYKMFNELTCKYGKTFKLYIGSNLYVCTSDIKLLEAIMTSNVHLNKSKVYDILKAWLKNGLIVSFREYWRPRRKIVSPSFHFNIMKEFFKVFKEASSRLVEKLKQEADTGKVFNIIEYLQKCSLDVVCKSVLGVNMNFEKSEGKQFVKAINDMCEIAFERNFSPWKRFDILYRLFGSTYEDCRQSVQVINDLTMNVVKQRRADIKAGKVEVSRSSGNKYTKNVILLDALFEYPSLTNEDIQEELINFMFAGYDTISTSLSFAMLNLSLHKDIQEKLYNEINSITDHDINNLTVQSLNEMKYVDQFFRESLRTFTTVPFIERELAEDADLNGVYIPKGTTMSLLIHWMHHDSDVHSNPETFDIERFSSENQRSNSAFAFLPFSAGPRNCVGQKFALMEAKTLLVGILSEFEILSVEGFKIELGLATLLTSKNGFPVRLQRRT*

>Ah CYP4D1

GFVPMLHRCTEKYGSMVNIFTGPVSVGLLVTDEKFLTHILNSNEIIDKSDQYDFLHNWIGDGVFKSS

>Ah CYP4D2

MSILVLLLILILNFCFKIFKINSALSNIPGPPPIPFFGYKLPKDVYEIYTMYNELFLKYGATFKLYAGNTMSVMTSDVKLIEAILKSTTHITKSNTYDLFRPWLNDGLILSDGQKWKNRRKICTPSFHFKIIKNYLEIFKDAAENMANKLRDFADTGTEIDIMEFSSGLSLDVICESAFGVKMNIQLGHSKEYSEAINSVAEISYKRIFSMWKQCDMLFTMFSSDYSKFQKAVKLLNDTSMNIIKERRAIFSETKEKLKKSCKDSNIYKTQTILSDALFDNSELTDKDVQEELNNFLFAGHDTASVTISFMFLTLSKHFDIQQKIFQEISENVGDDIKNLKTNDIQNMKYLDQVIKEVMRYRTPIPFIGRHFKEDAVIGDYFFPKGVNVFLLLHWLHNDNKYYSNPEKFNPERFSTENQANRAPFLYLPFSAGPRNCIGQKYGMLELKTVIIRILWEFVLLPVNNFKEELGIAALLKSKNGFPIKLKLRQTKIQ*

>Ah CYP4D8

FDEIINKAIETKQGIEMDDLNFLDLLLEVQRENDLPKKYVRDELNTIIFAGHDTTATSIALTLDELSKHQDIQEKVFEEQVKIFGQGYQNAQTSYADLKAMKYLELVLKESLRLHPVVSLIGRHVSADTKLSENINICKDENVVVFIHGMHHDPKYFIDPCKFIPERFADAIVPFSYLPFGGGPRVCVGQKYAMLQLKSCISKMVRHFHLMPSVPEHKIELLPSITLNIKNG

>Ah CYP4D14

MFVLSVLLAVIIVVFSFLFNWYYSLWKYRSLKNVPGPEPMPIIGNANDIGKTTVDLLNSFMKLHLEYGNFYKLWLGPRLHLMIAKPEYLEELLTSNVHLAKSNGYDMFKPWLGDGLLVSTGQKWKNRRKMITPTFHFKILEEFMKVFNKQFDIMLEILKEDIAKSSNTSIEITQYTNLASLDTICETAFGMSINAQQKSNPEYVKSVTNFLEIFTLRFFSMSLRNPIIFRFSSLYTKYNKTLKILHDFTNNIVKNRRKEFEQIEVKSTKVSEDGIKRRAALLDMLLEVNVDGHGLSDEDIREEVDTFMFEGHDTSATAICYVLYAISQNQEVQHKVYEEILEVIGDEKSNEITISKINDMKYLDIVVKEAFRIYSPVPLIERQLEEDWLIDGITIPKETSISIFLYGMNHDPNVFPDPEKFDPERFLPGKQSSRHTYGYIPFSAGPRNCVGQKYAVYQVKTCIIKFLMEFQMEEDKDFKPEIGMCSVLKSRNGIKIKLKP

>Ah CYP4E2

KIVLGYDGPFSCGLIIADAKLAEHILSSTKIIEKGAQYNNLHSWLGQGLLTSKGKDSNILTYDF

>Ah CYP4G123

MTQMLIDPDFNHLNDIYSPNNAFYFLVLPVLILLLVYWKVSRRNLIKLGEQIPGPKGYPIIGSALEFVGTSPEIFKRIYAKTYEYGKTIRLWAGPRLLIFITDPRDVEIILSSHDHIDKSNEYKFFKPWLGEGLLISTGSKWKSHRKLIAPTFNLNVLKSFIDLFNANSRETVKKMQQEINKDFDCHDYMSEATVEILLETVMGVSKKTQDQSGYDYAMAVMRMCDILHLRHTKIWLRSDFIFTRTKHAKTQEKLLNTIHSLTKKVIKEKRAAYEKGIKGSTAEVPDEIKTQKCEKNENYNINNGQTVVEGLSYGQSAGLKDDIDVDDDVGEKKRMAFLDLLVEASQDGSIINDEEIKEQVDTIMFEGHDTTAAASSFFLSLMGIHKDIQERVVQELDDIFGDTDRPVTFADTLEMKYLERCLMETLRMFPPVPVIARELKYNVKLVSENYTLPAGATIIIGTYRVHRDPNTYPNPDNFDPDNFLPERSVNRHYYSFIPFSAGPRSCVGRKYAMLKLKILLSTILRHYRIHSDLKEEDFQLQADIILKRAEGFKVRIEPRKCNLKV*

>Ah CYP4G15

MSATVIPDTVAPGGVITASNVFYLLLLPATLLFYAYWKISRRHLHELSNNIPGPSGLPILGSALEFVGSSADIFKRMYAKSFEFGKVVKIWVGPKLLIFLTDPRDVEIILSSHVHIDKASEYRFFKPWLGDGLLISTGQKWRAHRKLIAPTFHLNVLKSFIDLFNANSREVVQKLKKENNKEFDCHDYMSEATVEILLETAMGVSKKTQDQSGYDYALAVMKMCDILHIRHTKIWLRADWIFNLTQYATKQKGLIHTIHSLTRKVIKRKRADFEKGIRGSTAEVPEDAKTLKYEKNVSSKTVVEGLSYGQSAGLKDDLDVDDDIGEKKRMAFLDLLIEASQNGVVINDEEIKEQVDTIMFEGHDTTAAGSSFFLSLMGIHQDIQDRVIQEIDEIFGDSDRPATFADTLEMKYLERCLMETLRMFPPVPIIARQLRQDVKLASGDYTLPTGATIVIGTFKIHRDPDTYANPDKFDPDNFLPERSANRHYYSFIPFSAGPRSCVGRKYAMLKLKILLSTILRHYRVQSDLKEKDFQLQADIILKRAEGFKIKLEPRKRQLRAA*

>Ah CYP4Q9

IIYLLLTAIFAYLIYKLIQWQRVLNLVFKLPGPKRLPIVHNLFDLLYKDSAGYFSTMRQWNKEYGPVSAGSILGTPFVNVCGPEAFETVASTMKHITKGPLYKLVHPWLGMGLLTSTGSQWQQRRKILTPAFHFKILQEFLLVFNTEIDHLVNQLKEETDKPCTNILPLLSHSTLNIIAETSFGTQLDPKNSEDMKYVDAIYDMGRVVLYRGVRPWFYDTTLFDLFSSMSKLHKKVLHILHSFTDTIIEKRIQTFTPFDVTEDSSTRKKLAFLDLLLNAKIKSGIIDDKGIRDEVNTFMFEGH

>Ah CYP4Q15

MSQPTLISDTGEPESAWYGNTVLIFLLGITTLLTLYEYWCQNQRHVKLAKDIPSIKRLPIVGNAHLVLGKSAAGIFRLATDLSKTIQADVAKVFLGNRLIVAIYNAEDAETILGSSVHLQKSPDYSLFEPWLGDGLLISYGEKWKSHRKMIAPTFHATILKSFIPTFNKNTNRILEKLKEEKGKIFDCHDYMSTATVDTLLETAMGVEKTNEDNTGFDYAMAVMKMCNIVHLRHTKFWLRPDLFFNLTSMSKIQVKLLDTIHTLTNTVIKRKKKNYTERLLKGEDSLYTEVMKSSEYNESKGGAVDSSSIKYIRDDLDENDENDVGEKKRLAFLDFLIEANETQGGLLSDKEVSEEVNTIMFEGHDTTAAASSFFLCLLGIHTDVQRKVYEELKEIFEGNMDRPITFNDTLKMKYLERVLLETLRMYPPVPLIARQVNEDVKLATENYVIPKGTTVIVTQYLIHRNEKYWKNPTVFDPDNFLPENCQKRPYYAYIPFSAGPRSCVGRKYAMLKLKILLAGIVRKFEIISLEKEEDFNLQGDIILKREEGFKIKLSERV*

>Ah CYP4Q32

MTILLQVVYLFISFFVVYVTYRILKWQRMLNMVFKLPGPKRIPVVHNLVEMLSKSPVELLELIRQWSKAYGPVYVGAIMGQAAVNVCGPEDFETLSSSMKHISKGYIYNFLHPWLGKGLLTSTGTQWQQRRKILTPAFHFSILQEFTSVFNNEVDDLIERIRGEIDKPYTNVLPLMSHSTLNIIAETSFGTKLDPGNSEDEKYKKAIHSMGDILSYRSLRPWFKIQMVFDLFSSRSKENKMALKTLHDFTNRIIKKKTKTFTPFKISEDGSSKKKLVFLDLLLNAKVQKGDIDDEGVRDEVNTFMFEGHDTTSMALALSLLLLAEHREYQDMLYEEIVGVTGDSDRHPDFRELNDMKLMDRVVKECLRLYPSVPFISRALDCDTILGGFLIPKEVPIHIWIYDIHRNPKHWPDPEKFDPDRFLPENCVERHPYAYVPFSAGPRNCIGQKFAMLEIKTMLCGILKNFVVEPVDTRETIHFVPDLVLRPHNGILKLKFRLRKTYIIHQ*

>Ah CYP4Q33

MLIIAAVVVLLIFLCLPRFLEWYKFCQILDKIPGPKDYPIIGNWTETKINPESLFNYDRQLITEFYPICRIKFFYQYLIKIVCPEDMEAILSNVAHNNKNKFYRFMTDWLGTGLLISKDKKWHNRRKLLTPAFHFTILPQFVDIFNNGTQRLTEYFENNCHKEYIDILHPVTNFALESMGESAMGVNISSNNTEWYRNAIHSCGRLLQERLVKPWLHSDFIYSMTQSYQTMHKNINVLHEFTKKLITERKQYWNDIDKNSSVSYSKQKRLALLDLMLKMSETEGQIDDEGIREEVDTFMFEGHDTTAIAVCTILLTLASEPEYQEEIYKEIISVLGESDRNPTYNDLCEMKFMERCIKECLRLYPSVPIIGRVIGETFVSHSGYTIPKGTQVFIYIYDMHRNPNIWKNPEKFDPDRFLPENTVNRNPFTFLPFSAGGRNCIGQKFAMLELKAVLCGILRKFSLEPFDTRSDLKFKTDIILRPINKEIRIKFLPRTQQKKSLNSLI*

>Ah CYP4Q34

MIELLWVFLILTIIYLYIQRQKRYEEYWKHIMAIPGPKSVPILGTNYVRHTSESLFLRDRQRTQELYPIYKMWSFNVASVNLMSPEDMELILNNQTHIEKGLLYSFLHQWLGTGLLTNTGKSWHQRRKILTPAFHFSILREFMDMLNNETEILVTNLRKLSDQPYVDVTKPITEFTLYSIGETSMGVQLRNEPNCGVYKKAIYDIGEAFIYRIVRPWLYVPFVYSLSPAGKSYSEIVKTLHNFSNNLISERKKLFQKEKEQKGSYSQRKRLALLDLMLQAKENGADIDDDGIRQEVDTFVFEGHDTTAVCLCYTLMVLANEQSIQDEMYQEIISVLVDPKKPTFNDLGELKFMERCIKESLRLYPSVPIISRVLAENITTRTGYIIPKGCNVNLLFFDMHRRPEIWEDPEKFDPDRFLPSNAAKRHPYSYLPFSAGSRNCIGQKFAMLELKTALCGILKNFKLEPVDTPADMRYKLDLVLRPLGEIRVKFVPR*

>Ah CYP411A1

MPLLLIALVVSLFVYYHWSRRRFYAAEANYHGPRGLPIIGNALEFMCNAEDFHEKLKKLFSQVKEKPFRFWLGPHLLLSFKNPIHLEKIMSAQKFAYKHDIYALLEEQIGDGLISASGLKPKYKVHRKTIIPMLDLKYVTSALLIIQHHIEICLKKLEKHVDMGTFDVVSAIGPCSMDIIGEIIMGQKVNSQKTDHCQFSHAVINMMDVTFKRILQIWLHPKFIFRWHPLYREMEKSAHIVNSFVNKAISDSYQRRRTTTDKEEFIPLIDSLVNVKENNPNVISMQDLRHHLMTLMLASEDTFGIVTSFTVMCLGMYPEYQKIAVEEIRRLVGEEKKEITLDDTYKLEYLDMCIKDVMRLFPIAPYIIRKAGEDYQIDQYKVPAGAGIIVPIFHLHRDSDFWENPEHFHPDHFLPEAVKKRHPYTYLPFSTGPRGCIGKLLANVQIKLFIARFLQEFAIEADGKVPDLQLKEDISIRPKFGYNIRLKKREWF*

>Ah CYP411A2

MLTGNIPLSYVKMENENLGLTILSVICIAMVIIWYLKYLWYRRYMYYYSSKIPGPFSLPFIGIGFQMLFSGETSDIMGILLKFQKAYPDLCKIWFGPKLYIAVSNPEHIEKVLNSPKALDKDYLYKFLSFAIGEGLITARGQKWKKHRKAIMPAFNQKILDNYQKVFAQKSAIFTNILLENSGNKELDLAALLANCTLDMICETALGLDMDMQKTNMDFFMNLDKLMEITCLRIFKVWHHLTFTFPLFPISKEYVKCLNTFLNFTSAIIKKKLDVFNSNLPRKGSLTIEDKNIKEKENLAFLDLIMENSNFTEEELREEVNLFLIAGTDTTASALACTFVMLGMHQDVQEKVLEEILETMGPDRPVLPSDLPHLVYTERVIKESLRLFPVAAFFLRKIAEDIQVGDVVFPAGSSVYFGSVHIMRNPKYWPDPLKFDPDRFLPENVAKRHPCTYIPFSYGPRKCIGGRYAIMNMKTVLASVLRKVRIFTEYKTVKEIKLKTNIVLRMKDGPKVWVEKR*

>Ah CYP4H10

MIFLIFVTVVLGLFVNWCFGIYRNNKLLSKIPGPLPIPFFSSRLPKDNYDICVLVDDLLQKYGKTFKIYFGPSLNVATCDKNLIEAVFKSTDHISKSSMYDLMSDYLLNGIIVSDGDKWVSRRKLCNPQFKFAHLKDFFENFKTKSNDIVKILSKVAAEDTVININDFTAKYQLDVLFLSLLGIDLKVQNDIKNEYISASHTFIGFAALRNFSLWKRFDLLFNLFSKDAVKNNTAVKYLNNFGKKIIADRRKERQLLSNKTKEETNENISTGKTLLDTLLETPYLTDDDILEEINNFLIAGYDTVGTGTAAMAVELSQHKDIQDKLYEDIYYTLAGDFNNLTFQNLQEMRYLDLFVKEVLRLRSPVPLIERVLKEDAVFENTLYPKGTTIKLLLYAMHHQSDYFPDPEKLIPERFAEENDTLPFRPFSKGLRNCIGQRYAIMAMKSLITRLVWEFEFLPVKDFKFEVGYGAVLLSKKGYVVKLQKRQR*

>Ah CYP4V2

LTPTLHLQNLKNFVNTFNKEANILVKNIKNLSNLNSCVTVLKLMSEFSLNAMAETTLGVEIRHDPKLEDYSHALHVISEVFCYRLTHPCLYVSCIYNFTKTARKEAAAIKELHSFSNKIIQKRK

>Ah CYP4P1

KNDTFNFTFQNVQNMQYLDQCVKELLRLRPSGPLIERLLKKDATLDNMFFPKGTTISMFIYFMHNNAEYYPNPEKFDPGRFSVENQNARSPFTYIPFGGGQRNCIGQKFATLQIKTIITRLLLEFEFLPVKNFNMKIGFGVVLLSKKHQFP

>Ah CYP349G1

MEALCFLNLFKNLHLKSTTEILLATTVFTFIAWSIQYTWKRRHLYKYGGKVNGPISLPIIGSSLYFLGSNADIFEKIFQLYDIYKPPFKLWFGTKLFYGISDAAQSEIILTRCLKKDSVYKKAEIVMGNGLFTADLDLWKKHRKMIMPTFNQQNLDSFVDIFFEQGVTLNKKLNKEVNKGAFDVFHYLSLYTLDIICETAMGVKVNAQNKNAEYPKWADRIMEVVLLRIFNLLYYIDYVFFRLPIGREVIQITKKMQAFSFQVVQDKKQAFEIKQQDTDTVKDYAEENEGKRRRKALLDYLLEATNDTNIKFTEKELREEVDTFIIAGFDTTASTNAFVLMMLGMFPQLQEKVYNEAIEILGPDGPPTPSDLSKLKYLELFIKETMRLFPVGAFIARTADKDIDLGDCILPKGASAFIAILKIHTDPKYWPDPYKFDPYRFLPENSKNRAPCTYLPFSYGPRNCVGLKYAMMAIKTLIVTIIRKYEISTPFKQVQDVKLKANMVIRPIDGYLISLTYRK*

>Tc CYP4G7

MVVVEESLNHSLNLGNSVLISLGVVAVILAVYHFWLQSLRYTKLGNKIPGYDPLPIIGNAHMVMNKNPTQVMELALRVASEKGSVVRFWFGSKLGVALLDPRDIELILGSNVHLEKSSEYRFFEPWLGDGLLISKGDKWRSHRKMIAPTFHQSILKTFVPVFNKNAMDLVEQLRNEALDQICDVHDYLSGATVDVLLETVMGVKKTKEARTSYKYAKAVMDMCTILHFRHVKLWLRSDWIFSFTKLFKEQTSLLRIIHNLTDRVIKQKKKETEEENLKIKNEQTFNFGSGLRDDLDENDENLGEKKRLAFLDFMVEASQTEGNKLNDEEIREEVNTIMFEGHDTTAAASSFFICILGVYPEIQEKVYQELRDIFQDSDRPITFNDTLQMKYLERVLLETLRMYPPVPIITRVINEEVKLASGDYTLPVGTTVGIGQFLVHRNPKYFPNPDKFDPDNFLPERCQQRHYYSFIPFSAGPRSCVGRKYAMLKLKILLASIVRNFKIKSVVKEKDFQLQADIILKRADGFRVILTSRT

>Tc CYP4G15

MVVVEESLNHSLNLGNSVLISLGVVAVILAVYHFWLQSLRYTKLGNKIPGYDPLPIIGNAHMVMNKNPTQVMELALRVASEKGSVVRFWFGSKLGVALLDPRDIELILGSNVHLEKSSEYRFFEPWLGDGLLISKGDKWRSHRKMIAPTFHQSILKTFVPVFNKNAMDLVEQLRNEALDQICDVHDYLSGATVDVLLETVMGVKKTKEARTSYKYAKAVMDMCTILHFRHVKLWLRSDWIFSFTKLFKEQTSLLRIIHNLTDRVIKQKKKAYFERVKDGDVSLYNNAVKETEEENLKIKNEQTFNFGSGLRDDLDENDENLGEKKRLAFLDFMVEASQTEGNKLNDEEIREEVNTIMFEGHDTTAAASSFFICILGVYPEIQEKVYQELRDIFQDSDRPITFNDTLQMKYLERVLLETLRMYPPVPIITRVINEEVKLASGDYTLPVGTTVGIGQFLVHRNPKYFPNPDKFDPDNFLPERCQQRHYYSFIPFSAGPRSCVGRKYAMLKLKILLASIVRNFKIKSVVKEKDFQLQADIILKRADGFRVILTSRT

>Tc CYP4G14

MSTTVPTPDISTPSGILSASNVFYFLLIPALLLWYAYWRISKRHMLELAAKIPGPPGLPILGNALDLVGKPHQVFSHVYQKSFEYKKVVKMWAGPKLLVFLTDPSDIELILSSYVHIDKSSEYRFFKPWLGDGLLISTGQKWKAHRKLIAPTFHLNVLKSFIDLFNANSRDVIRKLQKEIGKEFDCHDYMSEATVEMLLETAMGVSKKTQDQSGYDYAMAVMKMCDILHLRHTKFWLRPDIIFNQTKYAEYQKSLINTIHSLTRKVIKRKRADFDKGIRGSTAEVPPELQTKNYDKTESKTVVEGLSYGQSAGLKDDLDVDDNDIGEKKRMAFLDLMIEASQNGVVINDEEIKEQVDTIMFEGHDTTAAGSSFFLSMMGVHQDIQDKVVQELYDIFGDSDRPATFADTLEMKYLERCLMETLRMFPPVPIIARQLNQDLKLASGDYTVPAGCTVVIGTFKVHRLEEYYPNPDKFDPDNFLPERTANRHYYSFIPFSAGPRSCVGRKYAMLKLKILLSTILRNYRIYSDLKEKDFQLQGDIILKRAEGFKVRLEPRKMAKA

>Tc CYP4BR1

MILAIVIALISAPFIWWMLILSKYRSLSNVPGPKPSPVIGNSLHIGYRPQDLFNNIVKWAKEYGTVYKIFVANDVRVLITNPELTELVLSSNVHITKSNAYDLLKPWLGIGLLISTGKKWKSRRKLITPTFHFKILDQFLDVFNSCGNVLVQRLSSQVGKDSFDVYPFINLCSLDIICETSMGVKIKAQQGNNTEYVEAVRRFLDIFIIRTFSVWKGIDALFKFSSAYSVYKRLLKILHQFTINIIVQRRGEKAQQKTQNVTSDDGIKRKVALLEMLLESEDNNMLSNEDIREEIDTFMFEGHDTTTSGIAFAILCLAENPKVQEKLYEEVVAVIDNIENITMQQLQEMKYLEMVLKEAQRLYPSVPVIERRLEVDCNIGGYDFPKDTFLSLFIYGMHHNEKYFPEPEKFDPNRYLPENQAKRHNYAYVPFSAGPRNCIGQKFAMLEMKTTIAKIVKHFKILPVPDYKPDLGIAAILKSYNGVCVRLQHRHGN

>Tc CYP4E1

MILAIVIALISAPFIWWMLILSKYRSLSNVPGPKPSPVIGNSLHIGYRPQDLFNNIVKWAKEYGTVYKIFVANDVRVLITNPELTELVLSSNVHITKSNAYDLLKPWLGIGLLISTETSMGVKIKAQQGNNTEYVEAVRRFLDIFIIRTFSVWKGIDALFKFSSAYSVYKRLLKILHQFTINIIVQRRGEKAQQKTQNVTSDDGIKRKVALLEMLLESEDNNMLSNEDIREEIDTFMFEGHDTTTSGIAFAILCLAENPKVQEKLYEEVVAVIDNIENITMQQLQEMKYLEMVLKEAQRLYPSVPVIERRLEVDCNIGGYDFPKDTFLSLFIYGMHHNEKYFPEPEKFDPNRYLPENQAKRHNYAYVPFSAGPRNCIGQKFAMLEMKTTIAKIVKHFKILPVPDYKPDLGIAAILKSYNGVCVRLQHRHGN

>Tc CYP4H1

MNIILEFFSALAVIIVLSWWYLFLKNYKVGKFFKNFSGPPGVPLLGNALDFKNKTKGVLPAFLNYNKNFGGLVKVQIGPFRKLLLVSDYKFLEFVLSSTKIINKSQNYRTMLPWLGTGLLTSEGIKWKKHRRIITPTFHFKILEQFINSFDAAGDVMINKLRKKVGIESVDIYPFVTLCALDIICETAMGTTINAQNNEESEYVTSVKEMGRIIIERAIAPQKNNEFLFRFTKDYQLQKSALKVLHNYTNNVISKRREELLKDQASKVSENVIDMGIKKKMAFLDLLLQATVDGRPLTNEEIREEVDTFMFEGHDTTASGISFALYCLANNPEAQEKAYEEQVALFGKEKKPIVSYSDLQEMKYLELVIKEALRLYPSVPFYARETNQEVEFGDIKIPKGVNITIFAYGIHRDPKYFPEPDKFDPGRFETIDGKLPYAYIPFSAGPRNCIGQKFAMLEMKSTISKVLRNFKLCPATPHHTLDLVAETVLKSDNGVRLSLMERQ*

>Tc CYP4BN11

MEILFTLFILLLCLLLYFYNKYVYIFDKNLKDYPAPPQVPILGHTLDFLIFLGFLDVLLSYTHKYGDIVRIRPGPIRQLLLTSNYKLFEAVLSNAKITKKSADYKFFHRWLGTGLLTSDGAKWKKHRQIITPTFHFQILENFVDVFEKNGKILTKQLEKHLNEDSVNVYGFVNLCALDIICEAAMGTSVKAQENMNSEYVRSVKDLLDTLMGRIFSPYKMIDFIYFFTEDYKKEMKALQVIHSYTRNVIKSRQAAINSGEFEQKTKKNFLDLLLAANEQQMTLEEIREEVDTFMFAGHDTTASTISFALFCLANHPDEQARVYREQKDIFGDDFKRAVTFQDLKKMKYLEYVIKETLRLYPVGPFFSRELDKDVPFAGKVLPKGLTITLFIYAMHRNPEYFPDPEKFNPSRFETFDGKMPFAFVPFGAGPRNCLGQKFAMLEMLSVVSRVVRTYKILPSIPRHEINVAAQVVLISTNGMRMRFEKR

>Tc CYP4Q1

MLLSTTLVCAIGLLGVFHLLAWIKFHASNYMKMCVIPGPPQKGILIGNMTYLQTTPEKIFLRLREATKNFYPIYKLNALHKCAANILNPEDCELIMSNSAHNQKGQIYDLLRNWLKDGLLTSFGAKWQTRRKILTPAFHFSILQQFVQIFNEEAEILVEDLKKDCSKSYISISSHITKFTLKTIAETAMGSKLKFETQKEIDYYQAVYDVGKILLYRLTHPWFIFHYVNFFSPWYLQEVKVTKTLHNFTREVIKHREENFKDIELPTEEHEVYKGKKRLAMLDLLLSAKHKEGIVENDGIQEEVDTFMFKGHDTTSAALCFALMLIASHSEVQESIVAEMREVLGDLSKKPSYNDLQNLKYLERCIKETLRLYPSVHFISRTLGQDLITTGGYTLPKESNAIIHIYDVHHNADIYPDPEKFDPDRFLPENVQKRHPYAYLPFSAGPRNCIGQRFAMLELKTAICAILANFTLQPIDTPETIILVVDIILRTKEPIKIKFVPRS

>Tc CYP4Q2

MFASTLILTTIFAIVSVLAFKRIKYHVKNYILLNKLPGPPPDGLFVGNMPYLQTTPEDIFNRLREANKKFYPIYKLFAVHRMGANILSPEDSEIVLSNPIHNEKGYIYKLLHHWLKEGLLTSKGEKWQLRRKILTPAFHFNILQQFIMILNEEAEKLVEGLRKECHKPYINITPHISQFTLKSITETAMGTKLDFTTKKEIRYKEAVYKIGKILTYRITHPWFIEPLLNIFSPCFIQERRVTSTLHKFTKEVIEDREKNFKDFELPTEEHDVYKGKKRLAMLDLLLSAKKKDGIIDNKGIQEEVDTFMFEGHDTTAVALNFALMLIACHKDVQETILQEMRDVLGDIHAKPTYSDLQNLKYLERCIKESLRLYPSVHLISRALGEDVRTQKGYLIPKDTITIIHIYDLHHNPDIYPDPEKFDPDRFLPENCQNRHPFAYLPFSAGPRNCIGQRFAMLELKAAICAILANFVLEPIDTPETIVVVVDIVLRTKEGIKIRFVPREQ

>Tc CYP4Q3

MLLSSSVLFVISFFLVLYFFKKLKCVSAKLYNLGKLDGPSNHNIIIGNVLRLHAAPEVIFKKLRQWARDYYPVYQLRAIHIPAACILAPEDFEAILSNPRHIEKSMVYLLLHDWLGTGLLTSGGQKWATRRKILTPAFHFSILQQFITIFNEETDKLVDTFKQQCDQPIEITSHITQFTLKTIGETAMGTKFEFTTRKQIEYKQAILEIGEILLYRLLNSWLISDYLNFFSPHRLREKRLVKTLHTFTRDVITEREKTFEKFQLPTEEHDVYIGKKRLAMLDLLLTAKNEERTIDEDGIREEVDTFMFEGHDTTSAALSFALMLLANHKNVQDQIVDEMVTVLGDLHQKPTYNNLQEMKYLERAIKESLRLYPSVHFISRKLGEDFVTCNGLKLPKSTITHLHIYDLHHNPDIYPDPEKFDPERFRPENSQKRHPFAYLPFSAGPRNCIGQKFAMLELKAAICGILANFILEPVDTPESIVLVVDLVLRTKNGIKIGFIPRVK

>Tc CYP4Q4

MFLAPIILCLVLLALLVSSLRKKGRFWECLAPIPQPPAYPIIGNLFDIMRTPEQMFLADRERGLKYYPIYKLDVCGTGGVNLLNPEDVELVLTDTKQNTKSFIYHFLHSWLGTGLLTSRGPKWQNRRKILTPAFHFNILQEFIQIFNEETKRLVEDLEAESHKPYIDVVVPITQFTLLSIGETAMGIKLNASDNDKDGYKRAVYKIGQLLTYRAPRPWIHNETIYSLTPQGRKEQKVLKSLHSFSNNVIAERKKHFSSSSYSSRKRLAMLDLLLKYKSEGANIDDEGIREEVDTFMFEGHDTTSVSICYTLMLLANHREVQEEILKEMEAVLDEEPPTYAKLQELKFMDRVIKESLRLYPSVPFISRVSGSEIQTKTGYTIPKDCMVNLQIYDMHHNPNVFPDPEKFDPDRFLPENIQKRHPFAYIPFSAGSRNCIGQKFAMLEIKTVLCGILKKFILEAVDTRKDMAFVSDLVLRPKGSIKVKFVPRRAN

>Tc CYP4Q5

MIQSTLLLIFIVILSVIIYIWWYQNFSRFFSLLNKVPGPPGYPIIGNIIQFLATPEELFKIDRELGRRFYPIYKEWTLTYGAVNLLHPDDIELVLSNSKYNDKSAIYDFLHCWLGTGLLTSSGTKWQTRRKVLTPAFHFNILQQFLPIFNEETVKLIKNIKNDQPIDVIPPVTQFTLLSIAETSMGVKLDASPKSCDDYKNAIHVFGYALTYRLGRPWLHNPFVFFNLTRLGHLTKKSIKILHDFSRNVIEQKKRTFEGEKRGKRLAMLDLLLLAKHQGADIDDEGIAEEVDTFMFEGHDTTSIAICYTLLLLANHPDIQDELYSELKSVLSDPTQTPSYSDLKQLNLMERCIKESLRIFPSVPFISRLLTEDLTTASGYVIPRGSMAHIHIYDLHNNPEIYPDPKKFDPDRFLPENCQKRHPFAYLPFSAGPRNCIGQKFAMLELKVVLSGILGNFVLEAVDKPKDVTMITDLVLRCKGPIRVKFVPRYKIN

>Tc CYP4Q6

MLSFYLLLTGVGLILLLQTWRFFRKYFKNKTILKKIRGPPETPIFGHMYIFTQDPDLLFSRIRKYSREYYPIFRFSALYVDIVNFLEPNDIELILSTTKHLSKSKIYTFLNNWLGTGLLTSTGAKWHQRRKILTPAFHFNILQQFLNIFNDETEKLVLRLGEDCGRTIDVVPPVTNFTLQSIAETAMGLSNINELTQKEYKKAIYKIGHIFLKRLSKPWYRLDAIYNYSKLAEEENATVKILHDFSNGIISEREKERTTNLSQNLASYSKKKRMAMLDLLLAAKNEGADIDYEGIREEVDTFMFEGHDTTSMAISFILLTLANLQDVQTKVREEILSVVGKEKIPTYNDLQELKYTERCIKETLRLFPSVPFISRYASEDFVTKTGYTIPEGTVLHIHIFDLHRNAEIYPDPLKFDPDRFLPEKVNERHPFAYIPFSAGPRNCIGQKFAFLELKTVLCGILRKFKLEKVDDMYEIEFRPDLVLRPKNDVKVRIEKL

>Tc CYP4Q7

MLVLTTVLTAAAVFLLFYLFYCYKTIKQHIYSLISLSYLPGPPVNDIISGNILPLYTSAENIFKQLREWGRLYYPIYKLNAAHLAAANILSPEDCELVLSNPTHMEKSAIYNLLHDWLGTGLLTSTGLKWQTRRKILTPAFHFSILQQFVAIFNEETDKLVEVLKEECYKPFINVNAHVAQFTLKTIAETAMGTKLRFTTRKETIYKQSIVDMGEFLLYRLLRCWLISKCIYVFNPRYYLEKKVTRRLHRFTKSVIAERQENFKEIVVPETDEVYTGKRRLAMLDLLLTAKNKEGLIDDEGIREEVDTFMFEGHDTTAAALGFALMVLAGHKEVQDKIVEEMNEVLGDIKKKPTYQDLQEMKYLERCVKEVLRLYPSVHFISRKLGEDLVTHSGHKLAKGSIVNLHIYDLHHNPAIYPDPEKFDPDRFLPENCQKRHPFAYLPFSAGPRNCIGKKFAMLELKAAICGILANFTLEPIDIPETIVLVVDIVLRTKEGIKVRFIPRV

>Tc CYP4Q9

MIILLIIVALVLALFLKPLGHYISIIKYVQQLPGPPCENFLHGNITHLYGPPEKMFKTVRKWGLEYYPIYQNWIAHVAVANVMNPYDFEVILSNMKHTSKSMIYDMLHCWLGTGLLTSDGIKWQTRRKILTPAFHFNILQEFIKIFNEETETLVQQLKKHSNSSVDVTEYISAFTLNTIGETAMGTSFGTETSTGREYKKSIQEIGEILKYKLLRPWLLSKFVYVCDPNYWKEIKLVKILHNFTNNVIANRQSNFKPVEQKSDEFSYSRRKRMAMLDLLLTAKNEDNLIDDEGIREEVDTFMFEGHDTTAVAICFALMCIACHPDIQERIFEEIEETFSDDTKPDYKSLQELKYMERCIKEVLRLYPSVPFIARSLGEDIVTYSGHKLKAGSMVHLHIYDMHHNPQVYPDPEKFDPDRFLPENCLKRHNFAYVFPSAGPRNCIGQKFAILEMKAVLVGILKEFTLEPVDVDTPETLVMKTDLVLRAMNGVRVKFVPRICAK*

>Tc CYP4BN1

MGALALVVFAVTTLLFLIWFIRLVRKNNDYYKDIPGPRPLFFFGNVFEVSTTRDILNVMHKYALQFNGLYKIHLGPVRKLIIASDYKFLECVLSSMKILNKSEDYLYLRPWLGTGLLTSDGPKWKKHRKIITPAFHFQILEQFVEVFESGGNRFVQQLQKEVGKKSVDIYPYVTLCTLDIICESAMGISINAQFDDNSDYVRSVKAMCRITMERLFHLLEMNDVTYPLTKNYYTQKKSLNVLHNQTNSVINRRRKELENKTEAINVTNEDKADNNDTLFGRNKKAFLDLLLQATVDGRPLTQEEIREEVDTFMFEGHDTTASAISFAIYCLANNADVQAKAYEEQIALFGGNKSPAVTYSDLQSMKYLELVIKETLRLYPSVPMFARKTSEPVQYENIFIPEGVTVNIFAYGIHRDPKYFKDPEKFDPSRFETVDGKLPYAYIPFSAGPRNCIGQKFAMLEMKSTISKVLRNFELQPATPTHTVQLAAESVLKSANGVKIALKNRN

>Tc CYP4BN4

MCCCFPLALAALHWPRDARVDDCRLCYPMPMRDAGQQLLQHQKLSGTLRMDTQAAAKTPTLALRHDAMQSFNRPTFYLVLDIMMKYMKIYGGVVKFHGGPIDKTLLVSDYNFLENVLSSTKILNKTDDYKFLHSWLGTGLLTSAGPKWKKHRRILTPAFHFKILEQFIDVFESAGNKLVKKLEKVVGKDSVDIYPFVTLCTLDIICETAMGIKINAQDNGESEYVRSVKQMCKIIIERSFSLLQMINLTYPLTKNFYIEKNALKILHQQTNSVINQRRQELKNQSKNIEQENDLGTKTRKAFLDLILEATVDGRPLTDTEIREEVDTFMFEGHDTTASAISFALFCLATHPEVQARALEEQKALFGDTKNPTPTYTDLQNMKYLEQVIKEALRLYPSVPFHGRKTNEAVEFNNGTVVPKDVTITVFTYGIHRNPEYFKDPEKFDPSRFDTIDGKLPYSFIPFSAGPRNCIGQKFAMLELKSTLSKVVRKFELRPATPEHKLQLTAETVLKSVNGIKISLKLRQ

>Tc CYP4BR3

MFVVAFFVTLTVILCTPLIWWYLTLAKYSSLKKVPGPPPLPVIGNSNVIGKTTVDLLHSFMNLQDKYGTVVKVWLGPRLHLLITKPEMVEFFLNSTVHLNKSDGYDLFKPWLGDGLLVSTGSKWKTRRKLITPTFHFKILENFLETSFNKQINILLDVLLKEASQTDKSIEIHSLINLCSLDIICETAFGTELNAQAKCNPKYVEAVTGFLEIFTLRFFSAWLRHPLIFRLSDKYAKYMEYLKILHDFTNTIIKRRKEEFKLLQDNAKISEEGIKRRAALLDMLLEVSDNGKNLTDEDIREEVDTFMFEGHDTTTTSICFVLYAIAQNPDVQKKIYDELVSVLGPDCKKEITFSDIQELKYLDVVIKEAHRLYPPVPLIERSLEEDCTIDGLTIPKNTNISIFLYGMNYNKDVYPEPHVFDPDRFLPEKQGERHTFAYVPFSAGPRNCIGQKFALLELKTTIAKLLRCFEISPDPKNPPQVGMCSVLKSRNGIHMFLKKKN

>Tc CYP4BN5

MITIVIILIFILVSINFITKYLKKLKQNKDFYQNIRGPRGLPILGSALDFKTTKDVLQNLINYTRDYGEVVTAQIGPFHRYILVSNYDFLECVLSSTKLSSKSHNYNFLRPWLGTGLLTSDGAKWKTHRRILTPAFHFQILEQFIEVFEKCGNTLLEKLRNEVDKETCDIYPYVTMCTLDIICESIMGISINAQEDSTSDYVQSVKNVCRIMVERSISPLQMCPFMYPLTKNYWTEKKSLKILHKQTNDVINARRKELESSKEVSSYEENFTKKKKPFLDLLLETKIDNRLLTQEEIREEVDTFMFEGHDTTASAVSFTLFCLANNLESQQKAFEEQQAIFGNIQNVTASYTDLQNMKYLEQVIKEALRLYPSVPFYGREITENVEYDGKLLPKGDILLIFAFGIHRNEKYFPNPEKFDPDRFHNMDNKTPYAYIPFSAGPRNCIGQKFAMLEMKSTVSKVLRQYKLLPTTPQHELELVGETILKSTNGIKIRVTLRN

>Tc CYP4BN2

MILPILLCLIFSFLIWSLYQTCKKNSFYKNIPGPSGVPIFGIAFEFKSTQDVLINLTSYIKTYGDIVTAKIGPFRRYLLVSDYNFLECVLSSTKLIKKSHHYTFFQGWLGTGLLTADGAKWKTHRRILTPAFHFQILEQFIEVFEKCGDVLVKKFENEVGRKSFDIYPYVTLHTLDVICESIMGISVNAQNNSTSEYVNSVKNICKIFVERSVSPLQMCRFMYPFTKNYWAEQKALKILHQHTNSVIDARRKELHKAENGHNTNPKKSKKPFLDLLLETKIDGIPLTQEEIREEVDTFMFEGHDTTASAISFTLYSLANNLHVQEKAVDEQKKIFGERKDVTAAYADLQNMKYLENIIKESLRLYPSVPFYNREITDDIMFDNKLLPKGDTIMVFAFAIHRNAKYFDNPEQFNPDRFNDLENKLPYAYIPFSAGPRNCIGQKFAMLEMKSTISKILRKYKLLPADPQHELNLVSELILKSSNGIKISIEPRC

>Tc CYP349A2

VKIWKKHRRIIVPTLNQSILNTFPQIICQQCDILLEILEKKCDKGEIDHYKFVTNFAVDVVSETIFGVPLNAQITDANYSRTFDKIMEVVFMRIFRVDYHSDFLFSWTKEYKAEQENVKLVKETTRDLIERKKEQITCQLEVEEEKKRPFLDVLVGKYLNEELSYQELEDEVSTFLLAGSDTNATAGCFVLTLLGMHQDVQEKLYEEIIEVLGPEKYPTLDDLPKLKYTERVIKETLRLFPGAPFIARIASDDIDLGDYVIPRGSNIAVGYVHLHRSEKYWEEPLKFNPERFLPENVAKRHPYTWLPFSGGLRNCVGGKFGMMVMKIMISMIIRKFRVKSSVKSVGDIELTANIVLKPKNGFRLAFTLR

>Tc CYP349A1

MFLLLVFLSLCFIWCLQFHWKRFRLYKLSRKIPGPLNLPLIGCAHLFFTGNAAEIAKRFMQMFDDYPDLAKAWMGPELYYLITKPEYLEVVLNHNATLEKMDLYKFIRPIVGDGLISSPVKVWKRHRKIIAPTFNQKVLNEFPGVICEQVNFLIELLRKECDKGEIDHCRYVTNCAIDIVGETIFGVSIGAQTGGKSYSLIFDKWMEVVFMRIFRLDYQFEWIFSWTKASKIQEEVKRIIHTMTRDIIEKKKEQIGCQLEADDEKKKPFLNLLVEKHLNNELTLQELEDEVNTFLLAGSDTNATSGSFILTLLGMHQDVQDKLYEEVSKILGPERPPTLDDLPKLKYTERVIKESLRVFPGAPFVARVVEEDVNLGDVIVPKGANIGLGYLHLHRSEKYWKEPLKFDPDRFLPENSINRHPYTWLPFSGGSRNCIGWKYGMMVMKIMTAMVIRKFRVKSSIKSIGDIELTANVVLKPKNGFRLAFEMR

>Tc CYP351C1

MFVVSAFLVGIVVLLLLRKRVTLWYYSAKLPGPFGLPFLGSLHLLVKGPKEIHQTLANIYKSYPKVVKIWFGPWLYVSTTEPSDLKVILTHHLDKGEFYELMSEYFRRALAAAPVDLWKKHRKNINPTFNTTILNTFIGAFAKQAEILVKNLEKYQSDEDIFPIVWKCTLDSACETLADVDPIFIDTEACLRRVFRIEEILLERFFNPLCHLNILWKFSPLRKELAVLWKQNSTFITQMIDIMKQSDNLDKKRFLNHLIPSHDLNYTIEEAQIMFFVGTETSGVAISSVLLILGMFPQIQEKIFIEIDQVFGSTTGSTLDEINHLDYLERVIKETLRLLPPIPFVMRSLDENLKLSCGTFPAGSRVIVPIMMVHRREDFWPEPLKFDPDRFLEERPSGTYIPFSYGTRNCLGYKYAMLSMKVILATILRKYRVKSSNYKSIDEVVLLIHIIAKATNGYKIVLEKRNK*

>Tc CYP352A1

MLFLLLVILIICCLVKYHWDRRKLYLFAAKLQGPKAYPVVGNGPLFWCKNEEIFNNFMAATAPYPSPVRLWVGPKLVLFVKDPNQLQLILQSSKITTKSFLYRFLEPFLGKGLFTSSGPRQKHHRKLLQPLFSQRMIEGYCHLFQKHSKKFVEDLRKNANGPEFNISTFLQFTAFEATMDLLLEDQDTHSIDYNEIPNYVRKFYEIVFTRVKSFWLHLDIFFKLSSYYREQTKLQSLATTVINEITETNVPKIIEKIKAERKLADAEIRVPSMLESIAEMVMENPDCLTMQDCTDHLMTFMATSQDTQSSAVAFTCMMLGAYPHIQDLVVQELREVMGKKTSLDLTDVSQLKYLEMCLKESMRLFPVGPFIFRDTTEDFQFDKMVIPEGVTVILSIYHAHRSPEHWEKPDEFYPEHFAPEAMSKRHPCAFIPFSAGPRRCIAQHYSYTYMKILLATIVLNYEIECRFKAEDVKLIADISIRPQQGYLIKLKDRTN

>Tc CYP350C1

MQSTTILVALSAFTAFIYFLAEYFSKKQRLLRFHAAKLQGPPALPLIGSAYYLFGSNKSIVNNVIHLVKKYKSPWKIWLGRQLYIVVTEPEDIEVVLNKALEKAENYNFLACLLQEGLFTGPVEKWKRHRKVILSTFSMPILKSFVEIFSENSLRMLPKLDKFVGKGTFDVCPILTNTALEITCETAMGTKIYEQAHSDYAYNLSKALELVFMRMFQYWLHPNFIWSLSIYSKQLKQLSKQLEIFVQGIVTEKKVQYRDKKHDLCLETKRRKCFIDHLIEMSEDDNWHDHELLEEAQTMVAAGSESLGSVKSFTLIMLGMHPLIQDKVYNEMYNIFGPSDRTVTPDDLTEMTYLDMVIKETLRLFPVTAAVGRRVSQDIVTDRYTLPEGCECIVSILSAHRNPKIWPKPLDFNPDRFLPEEVAKRHPYSYLPFSNGPRNCIGFKYAMMAIKTVISTIVRRYKISTEFKSVPEIEFSPGVVLKSRKGYRTQLESRC

>Tc CYP350A1

MYFVTPGLIFTVIALWLLWSFLWKNFFATKIKGPWAFPLIGSAYVLFGGTHNILENTVRVVSKYRPLCKLWLGNKLTIFPSNPDDIQMILNRSLEKEEGYRYIKTVFGHGLFTSPVSEWKGHRKVIVSTLNQQVLNSFMDTYNIYTRELIEKLKIDTKDGQHIDMFPIMTQCTLDIICSSIMGTQMNAMKEESQFIMWMTRVAELAIIRMFNIYLWPELFWKLSPISKESHELDQKIYNYVKTVIHNKRHKGLKSHTGKKIFLDYLVELTDREGRWTDEELTNETRSVIMAGSDATALTLSYCLVMLAMFQDIQDKVYEELCSYLDRFIKETLRVFPVTSMIGRELTTDMTIDGHFIPKGTSIGFPILYIHRNPEYYPDPLKFDPDRFLPEEVAKRHPCTFIPFSFGPRNCIGYRYAMMTMKVILATLLRSFKMVHTPYKEISELKIKFDIATKVDEGYPVRMELRKNVAAGTK

>Tc CYP4BM1

MDFTYLLLFTFFTIGFVNFCKWLKDRKRIWDAIEPIGGEKWYPLIGTCLELIKTKRKDFYDVYCARNMKYGPFFRTWMGFIPAVHVMKPDHVERILSSTVNLTKGDNYRFVVPWLGEGLITGSNHKKWRVHRKLITPTFHFSILDNMMEVMAEKGQFLAEQLVPKANGQFFNIYPFLTLCELDIICETAMGVEVNAMKHSQSEYVRSVYGISDIMLYRMFHPYLHPDFVFNLSSKGRQHKKYLSILHGFTRKVIQERKEKLTSGRDVVQELSEEDKLLGKKKRLAFLDLLLEANKNSEGGLTDEDIREEVDTFMFAGHDTSTVTVGWTLFTLSNYPEYQEKVHQELDEIFQGEERPITPQDVLKMQYLDKVIKETQRLIPVVPVIARTLDQDLEIVASSRTIPAGVMVVIHLARLHKDPDQFPEPDRFDPERFLPENVSKRHPYSFVPFSAGPRNCLGQKFALRNTKVLLASILRKYKVRAEKKIDEMKYNIEIVLRPQGGLSVALEPRRR

>Tc CYP351A7

MKRDYIDITCALTWAFSALALVFIIKYNWSRRWLYYYGSKISGPFAWPIIGSAHHFIGGQKVFYKNMIKLFETQPPIFKFWLGKDLIVVTSRPEDVETVLSNCFGKPKFYDYSYKLFRDGLLIAPAKIWKDRRKIINPTFNPRILNSFLDIFNKHANRVVDVFAEGCGKESFDVFLKLFRCTLDIACETLADVDSDLIKGQDAYLQKAIRMEDVLAIRSFSVWLHPDIVWNNSSFGKEVAKASPEVFGFIKQIIGLKKLNPVPEDEFCKKKRFVNHLLTASETNPHFDELAVEEETQTILITGSETTAITIGMVLIILGIYPEIQEKIMDELDLVLGPDDRTITLEDINKMEYLERVIKETLRVLPIVPIILRSVDEDIKLDPYTIPAGSFVLVPIGHIGKKPEFWKEANKFDPDRFLPENNSNRHRCTFIPFSYGARNCVGFKYGMMSMKVLLAAILRKYNVKPAQYKSLEEIELIFGMVTKPKHGFKIKLEKKTKKIVNV*

>Tc CYP351B1

MKLPGPWALPILGSAHLFHGGYDDMYQAMLRLTKSQPPLFKIWLGQDLIFVTSRPEDCEIILSKCFTKGKFATFMDPIFWEGLLTAPAIKWKSHRKIINPSFNIQILNSFIGVFNKHSKKLVEKMRKHAGSDSVDVSYWLFRNTIDISCETLMDIDADLIKGQEEYIDDCIKAEKYSSTRMIGVWYHPEFIWKHSPLGKLTKATSDKILDFVRKIIKLKKLEPEPLLKNCGDFPDDSMRKKHFLNNLLKMSENMTDDDILEETQTMLVAASETTALTMATVLLTLAIFPQVQEKIYEELDAILWNTDEITLEHINKMVYLEAVIKETMRILPTVPFINRRMTEDLHLNDCVVPTGSNIIISIKNIHDSPLLWENPDKFDPERFLTERDPNRSRCAFMPFGYGPRNCIGFKFAMLSMKVMMASLLKNFTFEPAVYKSIAEIECFYNIVAKPKKGYKVKFSERKLYNNNDKL

>Tc CYP4BN3

MALLTDVFLPLVIILLIFWYWYRSQKNKKYYQNVATPPTVPILGNALDFTTTTELLGTFMRYRKDYGGLVKVHIGPLRHFLLVSDYKMLEYLLSSPKIVDKSEDYKFLSSWLGTGLLLADGGPKWKKHRKILTPAFHFQILEQFVDVFDSCSNVLIQKLDKEVGNTSVDVYPFVTLFTLDVICESTMGTKINAQDDVTSDYVQSVKHMCRIIIERSISPVQMYDFLFVFTRNYFIQRRALRILHDKADGVISQRLRELQAQTKTDSEGDSEGIKKKKAFLDLILEAKVDGKPLSQDDIRQEVETFMFAGHDTTASAISFTLYCLANYPEVQKMAYEEQLSIFEDNNEPDVTYANLQSMKYLELVIKETLRLYPSVPIIGRQSGEDFQFDNSWIPKGDTMLLFLYGIHRDPKYFKDPEVFDPNRFENPDNKMPYSYIPFSAGPRNCIGQKFAMLEMKCVLSKILRKFELQPAVPQHNLLLTAETVLKSANGIKIGIKLRK

>Tc CYP4C3

MALLTDVFLPLVIILLIFWYWYRSQKNKKYYQNVATPPTVPILGNALDFTTTTELLGTFMRYRKDYGGLVKVHIGPLRHFLLVSDYKMLEYLLSSPKIVDKSEDYKFLSSWLGTGLLLADGGPKWKKHRKILTPAFHFQILEQFVDVFDSCSNVLIQKLDKEVGNTSVDVYPFVTLFTLDVICESTMGTKINAQDDVTSDYVQSVKHMCRIIIERSISPVQMYDFLFVFTRNYFIQRRALRILHDKADGVISQRLRELQAQTKTDSEGDSEGIKKKKAFLDLILEAKVDGKPLSQDDIRQEVETFMFAGHDTTASAISFTLYCLANYPEVQKMAYEEQLSIFEDNNEPDVTYANLQSMKYLELVIKETLRLYPSVPIIGRQSGEDFQFGKVFIKNVFLQFSK

>Tc CYP4BN8

MFIFAALFFLIIFYLYHQNVKLNRILCTFPEPPKKFLLGHVLDITSTTDVLNVFSKYINNYGPTIKLRAATLFAGLGTTDYKLCELLLSNNRILSKSLNYQYARNWLGNGLLISDGDYWRRHRKILTPAFHFEILKQFVETFESVGKVFVEKLDISQGPSVDLPPLVTLCTLDVICETAMGTQINAQKGQNAKYVQSVREMCRILIDRGLSPLKILNNTYWMTKDYYIEKKSLKVLHSFTSSVIEKRKAERNERNCTKLAFLDLLLKFSNEGELLTDQELREEVDTFMFEGHDTTASSISFVLFCLANHPEVQEKVLREQNELFGDDKDPSVTYHELQKMKYLEQVIKETLRLYPAVPIIGRCTSEDITFGEHFIPKDTNIAIYIYGIHRNPEHFPEPETFNPDRFKNSNSLPPYAYIPFSAGPRNCIGQKFAMLEIKSIVSRVVRCFELRPAEPYHSLVLSAETVLKSANGIKIGIRKRI

>Tc CYP4BN7

MYLIAIVIVLISLYLIVKPVKKTGNIDDFPEPPSKVFFGHALDAKSTTEILNVTTNYVKKYGKIVKLRLGPFFKALVVTDYKLVEFFLSSNSLLTKSQNYAFARSWLGNGLILANGTHWKQHRKILTPAFHLEILREYLETFDSVGRIFIEKLKSLDNVQSVDLDALVPLCTLDVICETAMGTKINAQKGENSEYVSSVKEMCRIIVARFLSPLKILKCTYWMTHDFYTERKVVNILHSFTSKVIDGRKTNRNETEKSGKKKAFLDLLLKFSDIEKLSDRDIREEVDTFMFAGHDTTATGVCFALYLLANNPEVQEKVLSEQKELFGDEKNPCVTYQELQNMKYLEYVIKETLRLYPSVPVIGRYLKEDTTFGDRVISAKTNVAIFIYGIHRNPDYFPEPEKFIPERFENMTNLPPYAYIPFSAGPRNCIGQKFAMLEMKSLISKVIRHFELTPANPHHELVLAAETVLKSANGIKIGLRARSENKM

>Tc CYP4BN10

MSVLTEFFIALVALILLPFYFYYTKIRRIENFFKHFPSPAGVPILGVAPEFKTTSSVLDNFHKYAKAHGTMVHLKFGPFKHALLCTDTKFLEFLLSSNKLLKKSVNLRFLEPWIGTGLVTADGGPKWKTHRKLITPAFHFKILEQFVDIFESGGDILVKKLSEVSGSKSCDIYPYITKCTLDIICETAMGTKVDVQNTENSAYFESIKEMCRIFTVRTVGAFLSNDFIFRFSKDYKIQTKAVKVLHNHSNEVIVNKRKELKEKNNENTSDIDELGRKKKKVFLDLILSATTDGQSLSPEEIREEVDTFMFGGHDTTGTATSFILYCIANNKEAQEKILEEQKQLFGDERSPKVTYSTLQEMKYLENAIKEGLRLYSPVPIYGRLIDQDTEYNGTMIPKGVGVIIFAHGIHMNPKYYPNPEKFDPSRFENTTGKDPFTFIPFSAGPRNCIGQKYAMLEIKSLVSKVVRNFELFPASPTHEMHLAPETVLKSLNGVKIGLKMR

>Tc CYP4BN9

MIVFTGYLIAIVLILFPLYYYYAKCYALEKFYHQFPSPPKTPFFGNALDLKTTKDLFQSFHRYVKAYGTMVHLKIGPFDHMLLCSNPKFLEFLLTSNTLLKKSPNLRYLVPWIGTGVATAYGPKWKLHRKLVTPAFHYSVLSQFVNTFESSGNILVEKISKLCDGQKVDNFDLYPHITRCTFDIICETAMGTKSNVQTNETSVYFESIKEMCRIFMSRTMAVFQRYDFIFRFSKDFHLQKKALDIIHNYSNEVIATKRKELDENVKQKESVDDLGRKEKMVFLDMILNKAIDGHIFSQEAIRGEVDTFMFAGHDTTATAISFILLCIANHNEVQDKIIEEQERLFGCEKNPKVTFAHLQEMQFLENVIKEGLRLYTPVPLYGRQIDKNVEFEGMLIPKGVNVVIFNHGVHMNPEFYPNPEKFDPNRFESMEDKHPFAFIPFSAGPRVCIGKKFAILEIKSILSKIVRNFELFPASPSYELDLAAETVLKSLNGIRIGIKRRETF

>Tc CYP4AA1

MWTDICTYLVILAVMLMSIYFKNYIRSVYLAFQLSGPPAVPLIGNVLLMKNDRSKYQFFRGHILSEMYSPFMRCWLSIFPVFFIYEPKALQLIMGTNRYTSKNIFYNCLHNVVGDGLITSSGEIWKQHRKLIQPYFHISVLKVYFDIFYKWSMAMVQDLENETEVNITKYANKFVFDSQYWAFLGKDQSRLLIGGNLVQHIHQCQFLMLHRITRPWLLFNTIFKRTDFSKVEENQKNILQQYTKSILDGKKQNSHGTAMNNLMEMMMEISNMNPEFTDDDIVNETCTFMLAGQDSVGAALSFALYSLAAHQDIQEKVVQELNGIFKDGNQAATFEDVAEMKYLEQCIKETLRLYPSVPMITRKITEDVPLGKYTLPTGTNIVISPFVTHRLPHVFPDPLKFDPDRFSPENKAKIHPYGFIPFSAGPRNCIGYKFAIIELKTVLSQILRKYHVSLVPGREKLILSYRMTLKAKKGIWLRLKKRSV*

>Dm CYP4C3

MSSKVITSLMAESILLSKVGQVISGYSPITVFLLGSILIFLVVYNKRRSRLVKYIEKIPGPAAMPFLGNAIEMNVDHDELFNRVIGMQKLWGTRIGINRVWQGTAPRVLLFEPETVEPILNSQKFVNKSHDYDYLHPWLGEGLLTSTDRKWHSRRKILTPAFHFKILDDFIDVFNEQSAVLARKLAVEVGSEAFNLFPYVTLCTLDIVCETAMGRRIYAQSNSESEYVKAVYGIGSIVQSRQAKIWLQSDFIFSLTAEYKLHQSYINTLHGFSNMVIRERKAELAILQENNNNNNNNAPDAYDDVGKKKRLAFLDLLIDASKEGTVLSNEDIREEVDTFMFEGHDTTSAAISWTLFLLGCHPEYQERVVEELDSIFGDDKETPATMKNLMDMRYLECCIKDSLRLFPSVPMMARMVGEDVNIGGKIVPAGTQAIIMTYALHRNPRVFPKPEQFNPDNFLPENCAGRHPFAYIPFSAGPRNCIGQKFAILEEKAVISTVLRKYKIEAVDRREDLTLLGELILRPKDGLRVKITPRD

>Dm CYP4D1

MFLVIGAILASALFVGLLLYHLKFKRLIDLISYMPGPPVLPLVGHGHHFIGKPPHEMVKKIFEFMETYSKDQVLKVWLGPELNVLMGNPKDVEVVLGTLRFNDKAGEYKALEPWLKEGLLVSRGRKWHKRRKIITPAFHFKILDQFVEVFEKGSRDLLRNMEQDRLKHGDSGFSLYDWINLCTMDTICETAMGVSINAQSNADSEYVQAVKTISMVLHKRMFNILYRFDLTYMLTPLARAEKKALNVLHQFTEKIIVQRREELIREGSSQESSNDDADVGAKRKMAFLDILLQSTVDERPLSNLDIREEVDTFMFEGHDTTSSALMFFFYNIATHPEAQKKCFEEIRSVVGNDKSTPVSYELLNQLHYVDLCVKETLRMYPSVPLLGRKVLEDCEINGKLIPAGTNIGISPLYLGRREELFSEPNIFKPERFDVVTTAEKLNPYAYIPFSAGPRNCIGQKFAMLEIKAIVANVLRHYEVDFVGDSSEPPVLIAELILRTKEPLMFKVRERVY

>Dm CYP4D2

MLGVVGVLLLVAFATLLLWDFLWRRRGNGILPGPRPLPFLGNLLMYRGLDPEQIMDFVKKNQRKYGRLYRVWILHQLAVFSTDPRDIEFVLSSQQHITKNNLYKLLNCWLGDGLLMSTGRKWHGRRKIITPTFHFKILEQFVEIFDQQSAVMVEQLQSRADGMTPINIFPVICLTALDIIAETAMGTKINAQKNPNLPYVQAVNDVTNILIKRFIHAWQRVDWIFRLTQPTEAKRQDKAIKVMHDFTENIIRERRETLVNNSKETTPEEEVNFLGQKRRMALLDVLLQSTIDGAPLSDEDIREEVDTFMFEGHDTTTSAISFCLYEISRHPEVQQRLQQEIRDVLGEDRKSPVTLRDLGELKFMENVIKESLRLHPPVPMIGRWFAEDVEIRGKHIPAGTNFTMGIFVLLRDPEYFESPDEFRPERFDADVPQIHPYAYIPFSAGPRNCIGQKFAMLEMKSTVSKLLRHFELLPLGPEPRHSMNIVLRSANGVHLGLKPRA

>Dm CYP4D8

MLLFLLVVLLFGAGWIIHLGQADRRRKVANLPGPICPPLIGAMQLMLRLNPKTFIKVGREYVLKFGHLQRVWIFNRLLIMSGDAELNEQLLSSQEHLVKHPVYKVLGQWLGNGLLLSDGKVWHQRRKIITPTFHFSILEQFVEVFDQQSNICVQRLAQKANGNTFDVYRSICAAALDIIAETAMGTKIYAQANESTPYAEAVNECTALLSWRFMSVYLQVELLFTLTHPHLKWRQTQLIRTMQEFTIKVIEKRRQALEDQQSKLMDTADEDVGSKRRMALLDVLLMSTVDGRPLTNDEIREEVDTFMFEGHDTTTSALSFCLHELSRHPEVQAKMLEEIVQVLGTDRSRPVSIRDLGELKYMECVIKESLRMYPPVPIVGRKLQTDFKYTHSVHGDGVIPAGSEIIIGIFGVHRQPETFPNPDEFIPERHENGSRVAPFKMIPFSAGPRNCIGQKFAQLEMKMMLAKIVREYELLPMGQRVECIVNIVLRSETGFQLGMRKRKHN

>Dm CYP4D14

MYLELFAILLATALAWDYMRKRRHNKMYAEAGIRGPKSYPLVGNAPLLINESPKTIFDMQFRLIAEFGKNIKTQMLGESGFMTADSKMIEAIMSSQQTIQKNNLYSLLVNWLGDGLLISQGKKWFRRRKIITPAFHFKILEDFVEVFDQQSATMVQKLYDRADGKTVINMFPVACLCAMDIIAETAMGVKINAQLQPQFTYVQSVTTASAMLAERFMNPLQRLDFTMKLFYPKLLDKLNDAVKNMHDFTNSVITERRELLQKAIADGGDADAALLNDVGQKRRMALLDVLLKSTIDGAPLSNDDIREEVDTFMFEGHDTTTSSIAFTCYLLARHPEVQARVFQEVRDVIGDDKSAPVTMKLLGELKYLECVIKESLRLFPSVPIIGRYISQDTVLDGKLIPADSNVIILIYHAQRDPDYFPDPEKFIPDRFSMERKGEISPFAYTPFSAGPRNCIGQKFAMLEMKSTISKMVRHFELLPLGEEVQPVLNVILRSTTGINCGLKPRVY

>Dm CYP4D20

MWLTLITGALILLLTWDFGRKRQRVLAFEKSAIPGPISIPILGCGLQALHLGAENIIGWVGEKFDKYGKTFRFWILGESLIYTKDLQYFETILSSTTLLEKGQLYEYLRPFLNDGLLVSTGRKWHARRKIFTHAFHFKVLEHYVEIMDRHSSVMVDNLRKVADGKTAVDMLKYVSLAALDVITEAAMGVQVNAQNDPDFPYIKALKSVVYIQPDRMFRFSRRYNWLFPLAAPLLHRQLLSDIRVMHDFTDKVISERRETVRRAKADGTYRPLSLGDAEIGSKSQMALLDILLQSSINNQPLSDADIREEVDTFMFEGDDTTSSGVSHALYAIARHPEVQQRIFEELQRVLGPDASAPVTQAQLQDLKYLDCVIKETMRLYPPVPAIGRHAQKELEIGDKTIPANTSIYLVLYYAHRDANYFPDPLSFRPERFLEDQEQGHNTFAYVPFSAGPKNCIGQKFAVLEMKVLISKVLRFYELLPLGEELKPMLNFILRSASGINVGLRPRKALR

>Dm CYP4D21

MWILLGIAVLIMTLVWDNSRKQWRVNTFEKSRILGPFTIPIVGNGLQALTLRPENFIQRFGDYFNKYGKTFRLWILGECLIYTKDLKYFESILSSSTLLKKAHLYRFLRDFLGDGLLLSTGNKWTSRRKVLAPAFHFKCLENFVEIMDRNSGIMVEKLKNYADGKTCVDLFKFVSLEALDVTTETAMGVQVNAQNEPNFPYTKALKSVVYIESKRLASVSMRYNWLFPLAAPLVYRRLQKDIAIMQDFTDKVIRERRAILERARADGTYKPLIMGDDDIGGKAKMTLLDILLQATIDNKPLSDVDIREEVDVFIFAGDDTTTSGVSHALHAISRHPKVQECIYEELVSVLGPDPDASVTQTKLLELKYLDCVIKETMRLHPPVPILGRYIPEDLKIGEITIPGNTSILLMPYYVYRDPEYFPDPLVFKPERWMDMKTTSNTPPLAYIPFSSGPKNCIGQKFANLQMKALISKVIRHYELLPLGADLKATYTFILSSSTGNNVGLKPRTRVK

>Dm CYP4E1

MWIVLCAFLALPLFLVTYFELGLLRRKRMLNKFQGPSMLPLVGNAHQMGNTPTEILNRFFGWWHEYGKDNFRYWIGYYSNIMVTNPKYMEFILSSQTLISKSDVYDLTHPWLGLGLLTSTGSKWHKHRKMITPAFHFNILQDFHEVMNENSTKFIDQLKKVADGGNIFDFQEEAHYLTLDVICDTAMGVSINAMENRSSSVVQAFKDITYTIKMRAFSPWKRNKYLFHFAPEYPEYSKTLKTLQDFTNEIIAKRIEVRKSGLEVGIKADEFSRKKMAFLDTLLSSKVDGRPLTSQELYEEVSTFMFEGHDTTTSGVGFAVYLLSRHPDEQEKLFNEQCDVMGASGLGRDATFQEISTMKHLDLFIKEAQRLYPSVPFIGRFTEKDYVIDGDIVPKGTTLNLGLLMLGYNDRVFKDPHKFQPERFDREKPGPFEYVPFSAGPRNCIGQKFALLEIKTVVSKIIRNFEVLPALDELVSKDGYISTTLGLQPAEKKSRDAHNHKYDPILSASMTLKSENGLHLRMKQRLVCDST

>Dm CYP4E2

MWFVLYIFLALPLLLVAYLELSTFRRRRVLNKFNGPRGLPLMGNAHQMGKNPSEILDTVFSWWHQYGKDNFVFWIGTYSNVLVTSSKYLEFILSSQTLITKSDIYQLTHPWLGLGLLTSTGSKWHKHRKMITPAFHFNILQDFHEVMNENSTKFIKHLKTVAAGDNIFDFQEQAHYLTLDVICDTAMGVSINAMENRSSSIVQAFKDMCYNINMRAFHPLKRNELLYRLAPDYPAYSRTLKTLQDFTNEIIAKRIEAHKSGAVSTNAGDEFTRKKMAFLDTLLSSTIDGRPLNSKELYEEVSTFMFEGHDTTTSGVSFAVYLLSRHQDEQRKLFKEQREVMGNSELGRDATFQEISQMKYLDLFIKEAQRVYPSVPFIGRFTEKDYVIDGDLVPKGTTLNLGLVMLGYNEKVFKDPHKFRPERFELEKPGPFEYVPFSAGPRNCIGQKFALLEIKTVVSKIIRNFEVLPALDELVSKDGYISTTIGLPDAERKKRDPYRHKYDPILSAVLTLKSENGLYIRLKERH

>Dm CYP4E3

MWLAVLALLVLPLITLVYFERKASQRRQLLKEFNGPTPVPILGNANRIGKNPAEILSTFFDWWYDYGKDNFLFWIGYSSHIVMTNPKQLEYILNSQQLIQKSTIYDLLHPWLGHGLLTSFGSKWHKHRKMITPSFHFNILQDFHEVMNENSAKFMTQLKKASAGDTIIDFQEHANYLTLDVICDTAMGVPINAMEQRDSSIVQAFRDMCYNINMRAFHPFKRSNRVFSLTPEFSAYQKTLKTLQDFTYDIIEKRVYALQNGGSKEDHDPSLPRKKMAFLDTLLSSTIDGRPLTRQEIYEEVSTFMFEGHDTTTSGVSFSVYLLSRHPDVQRKLYREQCEVMGHDMNRSVSFQEIAKMKYLDLFIKEAQRVYPSVPFIGRYCDKDYDINGSIVPKGTTLNLALILLGYNDRIFKDPHHFRPERFEEEKPAPFEYLPFSAGPRNCIGQKFALLELKTVISKVVRSFEVLPAVDELVSTDGRLNTYLGLAPDEKLKREAGRHKYDPILSAVLTLKSDNGLHLRLRERRS*

>Dm CYP4G1

MAVEVVQETLQQAASSSSTTVLGFSPMLTTLVGTLVAMALYEYWRRNSREYRMVANIPSPPELPILGQAHVAAGLSNAEILAVGLGYLNKYGETMKAWLGNVLLVFLTNPSDIELILSGHQHLTKAEEYRYFKPWFGDGLLISNGHHWRHHRKMIAPTFHQSILKSFVPTFVDHSKAVVARMGLEAGKSFDVHDYMSQTTVDILLSTAMGVKKLPEGNKSFEYAQAVVDMCDIIHKRQVKLLYRLDSIYKFTKLREKGDRMMNIILGMTSKVVKDRKENFQEESRAIVEEISTPVASTPASKKEGLRDDLDDIDENDVGAKRRLALLDAMVEMAKNPDIEWNEKDIMDEVNTIMFEGHDTTSAGSSFALCMMGIHKDIQAKVFAEQKAIFGDNMLRDCTFADTMEMKYLERVILETLRLYPPVPLIARRLDYDLKLASGPYTVPKGTTVIVLQYCVHRRPDIYPNPTKFDPDNFLPERMANRHYYSFIPFSAGPRSCVGRKYAMLKLKVLLSTIVRNYIVHSTDTEADFKLQADIILKLENGFNVSLEKRQYATVA

>Dm CYP4G15

MEVLKKDAALGSPSSVFYFLLLPTLVLWYIYWRLSRAHLYRLAGRLPGPRGLPIVGHLFDVIGPASSVFRTVIRKSAPFEHIAKMWIGPKLVVFIYDPRDVELLLSSHVYIDKASEYKFFKPWLGDGLLISTGQKWRSHRKLIAPTFHLNVLKSFIELFNENSRNVVRKLRAEDGRTFDCHDYMSEATVEILLETAMGVSKKTQDKSGFEYAMAVMRMCDILHARHRSIFLRNEFVFTLTRYYKEQGRLLNIIHGLTTKVIRSKKAAFEQGTRGSLAQCELKAAALEREREQNGGVDQTPSTAGSDEKDREKDKEKASPVAGLSYGQSAGLKDDLDVEDNDIGEKKRLAFLDLMLESAQNGALITDTEIKEQVDTIMFEGHDTTAAGSSFFLSLMGIHQDIQDRVLAELDSIFGDSQRPATFQDTLEMKYLERCLMETLRMYPPVPLIARELQEDLKLNSGNYVIPRGATVTVATVLLHRNPKVYANPNVFDPDNFLPERQANRHYYAFVPFSAGPRSCVGRKYAMLKLKILLSTILRNYRVYSDLTESDFKLQADIILKREEGFRVRLQPRTS

>Dm CYP4P1

MIILWLILALSALLYWLHRANKDYHILSFFTKRIRLKDGTPVEIIAPIAKGKTIFGNTLDLYGRDHAGVFNYSRERAKEMGTSYIEYVFGKAIYNIIDADSAENVLNHPNLITKGLVYNFLHPFLRTGLLTSTGKKWHARRKMLTPTFHFNILNQFQEIFKTESQKFLLQFEGQDEVTITLHDVIPRFTLNSICETAMGVKLDEMAEKGDRYRENFSQIEECFIRRLSNPLLWGDKLFEMFAAKDFASALDVVHRFSSEIIAKRRDLLKDELDKSSSTADDDGFVSKKRFAMLDTLIYAEKDGLIDHIGICEEVDTLMFEGYDTTSIGLIFGLMNMSLNPDKQELCYQEIQEHIDDDLSNLDVGQLNKLKYLEYFMKETTRLFPSVPIMGREAVQETELANGLILPKGAQITIHVFDIHRNAKYWDSPEEFRPERFLPENVQDRHTYAYVPFSAGQRNCIGKKYAMQEMKTLMVVLLKQFKVLKAIDPQKIVFHTGITLRTQDKIRVKLVRRT

>Dm CYP4P2

MMICLLWISVAILVVIHWIYKVNKDYNILAFFARRVQTKDGKPLDSLVPMIKGRTVFANCFDLLGKDTDQVFTHLRQLAKNSGDSYLQYSMGFSNFNVIDAHNAANILNHPNLITKGVIYNFLHPFLRTGVLTATEKKWHTRRSMLTRTFHLDILNQFQEIFIAESLKFVSQFQGQNEVVVSLKDRISRFTLNSICETAMGIKLDEMAEKGDRYRANFHIIDEGLTRRIVNPLYWDDCVYNMFTGHKYNAALKVVHEFSREIIAKRRVLLEEELENRRATQTADDDICVIRKKRFAMLDTLICAEKDGLIDDIGISEEVDTLMAEGYDTTSIGLVFGLMNMSLYAAEQELCYQEIQEHILDDLSNLNLSQLSKLNYLGYFIKETMRLYPSIPIMGRQTLQETELENGLILPKRSQINIHVFDIHRNPKYWESPEEFRPERFLPQNCLKRHPYAYIPFSAGQRNCIGQKYAMQEMKTLMVVILKHFKILPVIDPKSIVFQVGITLRFKNKIKVKLVRRNCV

>Dm CYP4P3

MLILWLVGAFIVLIQWIYRLNRDYCILGFFAKRIRTKNGQNPESIAPLVKGSTIFANSFDLYGKDHSGVFEHSRDCAKKLGKSYAEYAMGTAIYNVIDADSAERVLNDPNLINKGTIYDFLHPFLRTGLLTSTGKKWHARRKMLSPTFHFNILNQFQEIFITESLKFLEQFKGNDEAIISLNEVIPRFTLNSICETAMGVKLDEMAEKGDRYRENFRQIEECFIRRMSNPLLWSDTLFKMFAEKDYASALDVVHGFSSEIIAKRRDQLNDEIDSRGNTQTAEDELFTSKKRFAMLDTLILAEKDGLIDHIGICEEVDTLMFEGYDTTSIGLMFGLMNMSLYPEEQEKCYQEIQANIDDELNILNIGQLNKLKNLEYFIKETMRLFPSVPAMGRETTRETELSNGLILPKGSQIFVHVFDIHRNPEYWDSPEEFRPERFLPENSQNRHTYAYIPFSAGQRNCIGQKFAMQEMKTLMVALLKQFQILPEIDPKTIVFQTGLTLRTKNQIHVNS

>Dm CYP4AA1

MHLRLLSPPQLERTTNLELCSILILLVISLSIYTFYATLNTYLRSVLLSLRLTGPPSLPFLGNCMLVTDKDLMRRCAGKAFDLYGSLVRIWVLLFPFFAVLEPEDLQVILSSKKHTNKVFFYRLMHNFLGDGLITSSGSKWSNHRRLIQPAFHHNLLEKFIDTFVDASQSLYENLDAEAVGTEINIAKYVNNCVLDILNEAVLGVPIKKRGQDVAMMEDSPFRQGKIMMPARFTQPWLLLDGIYHWTKMANDELNQKKRLNDFTRKMIQRRRQIQNNNNGNSERKCLLDHMIEISESNRDFTEEDIVNEACTFMLAGQDSVGAAVAFTLFLLTQNPECQDRCVLELATIFEDSNRAPTMTDLHEMRYMEMCIKEALRLYPSVPLIARKLGEEVRLAKHTLPAGSNVFICPYATHRLAHIYPDPEKFQPERFSPENSENRHPYAFLPFSAGPRYCIGNRFAIMEIKTIVSRLLRSYQLLPVTGKTTIAATFRITLRASGGLWVRLKERDHPLIAH

>Dm CYP4AC1

MWIALLGIPILLAVLTLLLKHINKTYFILSLTKRVRTEDGSPLESKVAIMPGKTRFGNNLDILNFTPASVFNFVRESTAKAKGQNYLWYFLYAPMYNVVRPEEAEEVFQSTKLITKNVVYELIRPFLGDGLLISTDHKWHSRRKALTPAFHFNVLQSFLGIFKEECKKFLNVLEKNLDAELELNQVIPPFTLNNICETALGVKLDDMSEGNEYRKAIHAIEEVLIQRVCNPLMYYNWYFFVYGDYRKHLQNLRIVHDFSSRIIERKRQQFQQKQLGEVDEFGRKQRYAMLDTLLAAEADGQIDHQGICDEVNTFMFEGYDTTSTCLIFTLLMLALHEDVQKKCYEEVENLPEDSDDISMFQFNKLVYLECVIKESLRMFPSVPFIGRQCVEETVVNGMVMPKDTQISIHIYDIMRDPRHFPKPDLFQPDRFLPENTVNRHPFAYVPFSAGQRNCIGQKFAILEMKVLLAAVIRNFKLLPATQLEDLTFENGIVLRTQENIKVKLSKRVK*

>Dm CYP4AC2

MFLEVLFAAPLVIFIFRKLWAHLNRTYFILSLCKRIRTEDGSLLESKIYVAPSKTRFGNNFDLVNFTSESIFNFMRDASAKAKGRNYLWYFFHAPMYNIVRAEEAEEILQSSKLITKNMIYELLKPFLGEGLLISTDQKWHSRRKALTPAFHFKVLQSFLIIFKEECNKLVKVLHQSVNMELELNQVIPQFTLNNVCETALGVKLDDLSEGIRYRQSIHAIEEVMQQRLCNPFFYNIVYFFLFGDYRKQVNNLKIAHEFSSNIIEKRRSLFKSNQLGQEDEFGKKQRYAMLDTLLAAEADGQIDHQGICDEVNTFMFEGYDTTSTCLIFTLLMLALHEDVQKKCYEEIKYLPDDSDDISVFQFNELVYMECVIKESLRLFPSVPFIGRRCVEEGVVNGLIMPKNTQINIHLYEIMRDARHFSNPKMFQPDRFFPENTVNRHPFAFVPFSAGQRNCIGQKFAILEIKVLLAAVIRNFKILPVTLLDDLTFENGIVLRTKQNIKVKLVHRENK

>Dm CYP4AC3

MWIALLGSSLLIGALWLLLRQLNKTYFILSLCKRVRTADGSPLESKVFVVPGKTRFGNNLDLLNLTPANIFSYIRESTAKANGQNYIWNFLFAPEYNIVRAEDAEEIFQSTKITTKNMSYELIRPFLGDGLLISIDQKWHTRRKTLTPAFHFNILQSFLSIFKEESKKFIKILDKNVGFELELNQIIPQFTLNNICETALGVKLDDMSEGNEYRKAIHDFEIVFNQRMCNPLMFFNWYFFLFGDYKKYSRILRTIHGFSSGIIQRKRQQFKQKQLGQVDEFGKKQRYAMLDTLLAAEAEGKIDHQGICDEVNTFMFGGYDTTSTSLIFTLLLLALHADVQERCYEELQDLPEDIDEVSMFQFNELIHLECVIKESLRLFPSAPIIGRTCIEESVMNGLVLPKNAQISIHIYDIMRDARHFPKPNQFLPERFLPENSVNRHPFAFVPFSAGPRNCIGQKFGVLEIKVLLAAVIRNFKLLPATQLEDLTFENGIVLRTQQNIKVKFEARVK

>Dm CYP4AD1

MFLIAIAIILATILVFKGVRIFNYIDHMAGIMEMIPGPTPYPFVGNLFQFGLKPAEYPKKVLQYCRKYDFQGFRSLVFLQYHMMLSDPAEIQNILSSSSLLYKEHLYSFLRPWLGDGLLTSSGARWLKHQKLYAPAFERSAIEGYLRVVHRTGGQFVQKLDVLSDTQEVFDAQELVAKCTLDIVCENATGQDSSSLNGETSDLHGAIKDLCDVVQERTFSIVKRFDALFRLTSYYMKQRRALSLLRSELNRIISQRRHQLAAENTCQQGQPINKPFLDVLLTAKLDGKVLKEREIIEEVSTFIFTGHDPIAAAISFTLYTLSRHSEIQQKAAEEQRRIFGENFAGEADLARLDQMHYLELIIRETLRLYPSVPLIARTNRNPIDINGTKVAKCTTVIMCLIAMGYNEKYFDDPCTFRPERFENPTGNVGIEAFKSVPFSAGPRRCIAEKFAMYQMKALLSQLLRRFEILPAVDGLPPGINDHSREDCVPQSEYDPVLNIRVTLKSENGIQIRLRKR

>Dm CYP4AE1

MLVVLLVALLVTRLVASLFRLALKELRHPLQGVVPSVSRVPLLGAAWQMRSFQPDNLHDKFAEYVKRFGRSFMGTVLGHVVMVTAEPRHIDALLQGQHQLKKGTMYFALRGWLGDGLLLSRGKEWHTMRKIITPTFHFSILEQFVEVFDRQSSILVERLRTLSYGNEVVNIYPLVGLAALDIITETAMGVNVDAQGADSEVVHAVKDLTNILATRFMRPHLLFPHLFRLCWPSGFRKQQAGVICLHEFTNGIIEQRRRLLAREANQDKPTKPHALLDTLLRATVDGQPLTDKQIRDEVNTFIFEGHDTTTSAVSFCLYLLSRHEAVQQKLFEELRMHYGQDLFRGVILSDFATLPYLSCVVKESLRLYPPIPAVARCLEKDLVIDEGYIPVGTNVVVLLWQLLRDEAIFTDPLVFQPERHLGEEAPRLSPYSYIPFSAGPRNCIGQKFALLEMKTMVTKVIRHYQLLPMGADVEPSIKIVLRSKSGVNVGLRPRLY

>Ld CYP412A1

MDIFTEKISSTNVRTYLVMSLITVFGVWYIRFLWNRRFLYIYTWNMEGPLSFPFLGCGYLFLGSTHELLKTLIKTQENSKTGIVRLWIGTKLAYLISEPTHIEKILNHPKALNKDDIYKIITSILGEGLLTAPVDTWKRHRKLMAPSFNQIVLSSSVDVFTEHCSIFAKELKAVLGKKNVDLYEWVIRTTFDIICQTALGVNLNLQKNRKEYPVLLERGLDITSDRMFSFWKPYKFLWYFSTDKKIFDDIYMNLHVLFQSLMRKKMSEYEKELEAERLDANVEEESIERLPFLNLMLKDPSFTMEEVIAEAKTFCLAGSETNASSTCFLLNLLGLYPEVQQKVYEEIIDILGPDRTILASDLPQMKYTERVIKENLRLFPVATIFGREIEEDIDLGNAILPKGSSVYFTPHYIHRNPKYWTNPLKFDPDRFLPDEIAKRHPCTFIPFSYGPRNCIGWKYAIFSMKIIIATIIREFKIFSEYKSIEEIEVKLYMLSKLKDRSKFWLELRQ

>Ld CYP412A2

MISSEDFSRYDVRTYLVVSLITAFAVWYIKFLWNRKYLYIYSWKVEGPLSFPFLGCGYLLLGNLINNITKQQAKSRTGIVRFWMGMNLVYLLSEPIHLQKVLSHPKALEKDYSYKYVTETIGEGLLSAPVNVWKRHRKLLAPSFNHRVLNTAVGIFTEYSVNFAKELQAVVGKKNFDLYAWTIRCTFDLICKTSMGINVETQKNRGDQGILFERAFEIMSYRMFRVWNHLDFVWYYSPNKKIFDGIYSKLYDLLHPLISKKFIEFKKELSGKHLDAEIEEESLYEKRLPFLDSMLLDPSITEKKVQDEAVAFALAGSETTASSICFLFSLLGLFPEVQQKVYEEMIDILGPDRNVEAFDLPKMKYTERVIKENLRLFPIAALVARRIEEDINLGDVILPAGSSVAFNLLYIHRNPKYWTEPLKFDPDRFLPEEIAKRHPCTFIPFLYGPRNCLGWKYAFLNMKIIIATVVREFKIFTEYKSIEEMEVVFHFTIQLKNGPKVWLERRR

>Ld CYP413A1

MSYCSPYDKTSFLYYVDENKLFSGITFFTFAVIGVFCYILRCYSFFEKHKIPRAVTPFAPYGNIKDILFRNTTMYVFLWKYYNKFKRKGYKFAGFYLFFKPGILAVHPSLTFKILSDDRGFDNVSRKKNVLDYKKVLRMFDDSILQQLIESSNAIKPSLEEQLAGKTPLKEVLYGFLAESSCLTFGFNKGSADVLTKSADEKIRSGFWHYLKLVLSFTKSKSSNEDLRNFIKDVCGYRKKHDVREKDLLQCFTDTLRDDRESEGIIDEIIEIAVENIIYSCSTVLFCLYELASNPDIQDDLIGEIRRFNKNNSTINLDNLHHLIYLEAVVKETLRKYPPVPLVTKKPKEDYNMNDFGLDLPKSCLIFATIFGVHHDPANFPDPEAFDPDRFFEDNIKYIKPNTYFPFGLDSRNTIGFRLTNVHVELCIFNILSSFRVDLTKKDHVLEFDNDKLVTYPKEPLPLRFELLTN

>Ld CYP4G57

GIGKSPTDVYDLVYDLYHKLNSDIVKLYFGPKLYIGICSAEDAELILGSSVHLEKPQEYRLFEPWLGDGLLISKGEKWRSHRKMIAPTFHTSILKSFMPVFNKNANNLVEQFKKEKKVFDVHNQMSAVTVDTLLETSMGVVKTDEDNTGFDYAMAVMDMCNILHLRHYKVWLRLDFLFRWTKMFTHQKNLLNIIHSLTRQ

>Ld CYP4G58

IAITDNEVIQQFFKDSIVKAGDYAFFKPWLGTGLLTNNGQSWKSRRKLLSQCFGSLGLMKDFIKVFETSGRHFMN

>Ld CYP4AA1

GQDSLGSAVAFTLFHLAKYHDIQNRVLKEISNYEEGTKLSNKELFGMKYLEQVIKETLRLAPSVPIFARVLTEDVVLDDVEFPRGTNIFISPFLTHRHPQIYPDPLKFDPDRFSTENVENRHPFAYLPFSLGPRKCIGYQFALMEMKTIIFTILKKYELSLVPGHEEYTLTYRTTLRAKGGIWIRLKARK

>Ld CYP4BN15

HVTPLQKARKNIIHRLRRKGEYFYSRLRFIEFVLGTHYILDKSRDYRFIRNWLGTGLLTSDGPKWKGRRRIATPAFHFSILEQFVEIFEKNGNIMINKLKKEVDKDSIDIYPFITLCALDIICETAMGVPVDAQLKENSQYVKNVKLMCKIGVLRSTSILKHNDFFYPLTLDYYREQKAVKQLHEVTNSVIESRMKHLESSGGKVKENADELGRKKKLAFLDILLQSTVDGKPLSIEDIREEVDTFMFEGHDTTASAMTFTSFLLAGHPEVQARALEEQTSVFADDLLR

>Ld CYP4CW1

FKMYLGSTLHVVTTDATFLEDLLRSNVHITKSHIYDMTRPWIGDGLLNSVGMKWKQRRKMLTPSVHFSVVSSYLPIFKRSAEILVQRIYKQLETESFIDIHPFVPTFTLDIAGATSFGYDFGSQEGRNHDFLKAVENFTKTLWIRNFSVWQRSYTLFKLFSSKYAAFKKDLDIIIGLSY

>Ld CYP4G15

MSAATASVDLENPTTLLTPKNIFYFLLIPALVLWYAYWKISRRHMVELASKIPGPEGLPLLGSALEFVGTSADIFKRMYAKSFEYGNTVKVWIGPKLLIFLVDPRDVEIILSSHVHIDKASEYRFFQPWLGDGLLISTGQKWRAHRKLIAPTFHLNVLKSFIDLFNANSREVVQKLKKEVGKEFDCHDYMSEATVEILLETAMGVSKKTQDQSGYDYAMAVMKMCDILHLRHTKVWLRPDFIFNLTNYAKKQEGLIGIIHSLTRKVIKRKRADFEKGIRGSTAEVPEELKTKNYDKNVSSKTVVEGLSYGQAAGLKDDLDVDDDVGEKKRMAFLDLMIEASQNGVVINDEEIKEQVDTIMFEGHDTTAAGSSFFLSMMGVHQDIQDKVVQEIDEIFGDSDRPATFADTLEMKYLERCLMETLRMYPPVPIIARQLRQDVKLASGDYTLPAGATIVIGTFKIHRQEDVYPNPDKFDPDNFLPERSANRHYYSFIPFSAGPRSCVGRKYAMLKLKILLSTILRNYRIYSTVEEKDFQLQGDIILKRADGFRIKLEPRKRVLKA

>Ld CYP4C3

MDSKVALEWSFLPPILFGIAITIFTYVWFSRREYLRKIEWVEEVPGLPFLGCILNLSKSTEILNVFSNYCRQYNGLARLNLFGKRFLLVSDYKFLEFVLNSNEILDKNDSYKFLNNWLGKGLLTVEGSKWKKSRKLLTPAFHFSIVDQFVEVFDNNANILVGLLEKEQENDFVDVFPYITMYTLDNICEATMGVSINAQTDTQSEYVFAVKEMCRITVDRTFSPIKMFNTLYPLTADYYKEKKYVNTLHEKTNSIIISRQKELIRKRAEEISSGDSVPREDEGTGIKKRMAFLDLLLQTPDETLTNEDIRQEVDTFMFAGHDTTASAVSFIIYCLAEHPEHQEKIVEELETIFGDDKSRPVTVADLQSMKYTEMVINETLRLYPSVPFTGRILNKDVKYEDGKILPEGLSLILYIYGANRNGQYYEDPNTFNPLRFESEKPAPYSYIPFSAGPRNCIGQKFAMLEMKATVAKLLLKFEIVSCGHVPILAAETVLFSKNGVKVTLKTRM

>Ld CYP4C1

MLYLKKYGPHIKVYNGPISTAVVSADEKFIEFFLSSPKLIDKADEYSFLHNWLGLGLLTSTGLKWKKRRKMLTPAFHFSILESFVDVFETVGDIFIKKLEEYVGKASFDIYPLVSLCTLDIICEAAMGVSINAQENSHSEYVRSVKEICAIVISRIFSPLDPRLYPLTWTSIREKIAVKKLHAHTNFIIERRMKEVAVNGIDDGIDDVGRKKKLAFLDMLLKSSVDGQSLSMADIREEVDTFMFEGHDTTSSAISFAIYCLAAHPEVQKKALEEQKHLFGNLKEVRPKAQDLINMKYLELVIKEVLRLYPSVPFLGRKVPEDFEWGGTIYPKGVNILLLIFASHRSPNYFPEPLKFIPERFENYDEKRPYLYVPFSAGPRNCIGQKFAMLEMKSTISKVLRHFEILPATPEHKLKLAPETILVSKNGVCISLRKRFELE

>Ld CYP4D2

MTLVFLAALVVLAPALYFGYLVYKHKDVKLPGPTPYPFYGLKSPTDIYGLFKMYCELTQKYGKTFKMYLGSTLHVVTTDATFLEDLLRSNVHITKSHIYDMTRPWIGDGLLNSVGMKWKQRRKMLTPSVHFSVVSSYLPIFKRSAEILVQRIYKQLETESVIDIHPFVPTFTLDIAGATSFGYDFGSQEGRNHDFLKAVENFTKTLWIRNFSVWQRSYTLFKLFSSKYAAFKKDLDIIIGLSYRVIDNKREYYEKHGKVQGIDSNENSYKKKVALIDALLQLPGLSDRDIQEEVNTFLFGGNDTLATALSFNLYELSKHPEIQAKLYEEVSHYSEIDPEMSLDTLQAMPYLHKVIKETLRLRTTVPIVERLLEEDAVIGGLKLPKGTVISMAVYWLHHNSEYYPNPEKFDPERFDPENDKKRPTYSFLAFSAGPKNCIGQRYAMLVLKNTISRIIKEFELLPVPDHKLELGMALVLKSKYGLPIRFKPRPKIEKDYCLMRKHSEKQINV

>Ld CYP4D8

MINQSFNMNLMERFMQVFNSASDALVKNLEKESGKDCTDILNYTNFSALEIACENLMGIKLKPEENAAEEEYIEKTKVMLRIVGVRFFSFWQRFETIFLMFSKHSKIYLDYVGTLKKFTLDVIERRSKTFKEERKEVGTENDFGIKKKAALMDILLEESERGTLTYEDIREEIDTFMVAGHETSAGILSFALFELARQPEAQQTLYEEIMAIAPEGDISMDQLNDMQYLECAVKEALRMYPQVPFMERQIIEEFELDGIKYPIGTVFSIPIINMQNDPDLFPDPGTYDPSRFLPENADKIPKYNYLPFSAGPRICIGYKYAMYSAKCSLAKVIREYEVLPVPGFNVQLEFQITLKSVTGINIRLKKRKHQR

>Ld CYP4AA1

MDFKLVTSTFQSLTQNMQIMKYLVASSAVVILFLIYIRTYLRSVYLALKLPGPKALPIIGNVLFLNDDREMEHLGNKAHELYGSFMRFWVSFLPCIVVHEPRHLNIILGTNRYSRKNIFYTLLHNFLGQGLITNNGYAWKKNRKMIQSLFHTNIQEIFIETFQDCAEAFVSKLNGLTDVKITALVNDCVLDILHNAILGVPLDKNSPYRKGQVILNDRLTKPWLLLETIFSRTTSAQKEIEQKTNLHSYTKKCMSMRTSTFAQKLLLGDYVFSFPGYQFALMEMKTIIFTILKKYELSLVPGHEEYTLTYRTTLRAKGGIWIRLKARK

>Ap CYP4G11

MAAASATGFSASSVFLSLLIPALILYFIYFRISRRHLLELAEKIPGPPALPLIGNALDLFGSPDAMFSQVLKKAENFKDVVKIWVGPKLVICLIDPRDVEIILSSNVYIDKSTEYRFFKPWLGDGLLISTGQKWRNHRKLIAPTFHLNVLKSFIDLFNANARSVVEKMRKENGKEFDCHNYMSELTVDILLETAMGVSKPTRDHNAFEYAMAVMKMCDILHLRHTKIWLRPDWLFNLTKYGKNQIKLLEIIHGLTKKVIQLKKEEYKSGKRNIIDNSAQKTESKTNNIVVEGVSFGQSVGLKDDLDIDDDVGEKKRQAFLDLLIEAGQNGVLLTDKEVKEQVDTIMFEGHDTTASGSSFFLAVMGCHPDIQEKVIQELDEIFGDSDRPATFQDTLEMKYLERCLLETLRMYPPVPLIAREIKTDLKLASGDYTIPAGCTVVIGTFKLHRQPHIYPNPDVFDPDNFLPEKTANRHYYAFVPFSAGPRSCVGRKYAMLKLKIVLSTILRNFRVRSDVKESEFRLQADIILKRADGFKIRLEPRKQVASTA

>Ap CYP4AA1

MVVTAQQIWEHTPGEAWVYLVILTSIYILYILRTYIRSIIYISQLNGPKTIPIIGNANSILKENFLYRLAYESQMYGRIVRIWLTIFPYVMFLEPEDIQLILNNAKHTQKVFFYKLLDNFLGKGLITRDIKSWRIHRRLLQPAFHLHVLEKFTNTFAKHADHLMNKILEKNNQEINITTFINDSVYNILSETVLGINRINRINEMDDLPFRKGQIMLLYRMIRPWLLIEWIYRLTKYGREEEKQRKNLFDTCFKMVKEKRDLLQSKDRISNNDIKKNKNISLLEYMVEINEKNPCFSDEDIVEECCTFMLAGQDSVGTATAMTIFLLANHPEWQNKCIEEIDEIFNGDTRFPTISDLKEMKCLEMCIKESLRLYPSVPIIGRTLGEDIKIGKHIIPAGCSVLISPYSTHHLPHHFPDPDTFKPERFNSENSEKRHPYAYIPFSAGPRNCIGYKFAMLEMKSIISAILRKCRLQSIPGKKEIRPKFRMTIRAQGGLWVKIIERDQILKSIAA

>Ap CYP4C1

MISAILFFIFLLATLHYFLLHHRKFGKMINLIPGPEPLPILGNIPTFHNISPSELWKFLTQLSKQYYPIYRMWTFLEAYVHICHPDDIETILGNIKFTKKGFGYKYLKPWFNTGLLTSSGHKWHVRRKILTSAFHFNVLRQFVDIFIEDAERLIKTLESEEGIFVENLLQLTSEHTLNVICETAMGTSLKNKEKFQYEYRKAVYNMGCIFANRIVKPWFYYDFFFNLSPEGWQQSKLLKILHNFTRKIIQERKEYHDKTNGRYLNDFHENINENDNNNDYNDCRIRRKRLAMLDLLIEAHRNNKIDDEGIREEVDTFMFRGHDTTAISFCFSIMLLAEHKEIQDRARAEIKAAIEENGGKLNITVLQNLPYLERCIKESLRLFPSVPRISRKLETSVKLSNYEIPSNTIINVNIFDTHRDPKFWPNPNKFDPDRFLPENSKKRHPYAYVPFSAGPRNCIGQRFAMLELKTYLGLLLYNYYFEPIDYLKDVTFVSGIVLRLENPVRMKFIPVKKIC

>Ap CYP4D2

MLEIVIATILLLFATPFIWWYLLLEKFKSLNDVPGPKPIPILGNANVVGNTTVSLLNSFMDLQKQYGGLYKLWLGTKLNLMVTKPEYVEYFTSSNTHLSKSDGYDLFKPWLGDSLLVTTGQKWRSRRKMITPTFHFQVLEKFMDVFNAQSNTLLSNLEKEVETNNNKRIEMHYFVNMCTLDIICETAFGTSIRAQHKGNPKYVEAVSSFLEIFTLRFFSPWMANDWLFRFSELYPRYKKHLKILHDFTNNVIKDRRAARKERKDNPISEDGIKIKAALLDMLLDASENGYDLTDEDIRAEVDTFMFEGHDTASTAVCFTLLALAQNPAIQHKVYEELVEVLGPESTDDVTCSQMNDLKYLDIVIKESMRLYSPVPLIARRLDTDCVLDGITIPKDTTINIFLYGMNHSTEVFPEPEKFDPERFLPEKQSSRHNFAYVPFAAGPRNCIGQKFAVLELKTIISKICRKFEIVPVENYKPEIGMCSVLKSRNGIVMGLKVRQ

Supplementary Table 3. Primers used in RT-PCR for determination expression levels of *Agasicles hygrophila* CYP genes.

| Gene name | Forward primers (5'-3') | Reverse primers (5'-3') | **Amplicon size (bp)** |
| --- | --- | --- | --- |
| *Actin* | ACGTAGGAGATGAAGCCC | GCGTACAAGGAAAGTA | 274 |
| *CoxI* | ATCATCTAATATTGCCCACGG | CCTCCCCCGACAGGGT | 282 |
| *AhCYP15A1* | AAGACTTCGATGGAAGGC | ACCAATCTGAAAGCGTCA | 355 |
| *AhCYP18A1* | ATATGGATCGCTCATTTC | ACTAACAGGAAATCTTCC | 291 |
| *AhCYP303A1* | AGTAATAGCCAACTTACACA | TTAAACGGCTTGGGTCC | 288 |
| *AhCYP305A1* | AGAGACTTGGAGACAGC | TACCATCATCAGGTGTTC | 505 |
| *AhCYP306A1* | ATTCGACAAATTAGC | TATATTTCAGCAATTTGTGG | 482 |
| *AhCYP307A1* | ATATGTTTTCTTTGGTAAC | TCAAATTTGTCCTGAATGAC | 298 |
| *AhCYP307A2* | ACTTGCAGAGGACGCG | GGAGGGGAATTGTAGTCC | 436 |
| *AhCYP12A2* | AGTCATCGAGAAAGTTG | TTGGGGACATTCTTAAAG | 279 |
| *AhCYP49A1* | ATGATAACGATGTTTGTC | TAAATACTGTTGCACCTG | 537 |
| *AhCYP301A1* | GATATTTCTATATTAGAAAC | CAGACTTTAACCACCTTTC | 439 |
| *AhCYP302A1* | AGGAAATTGTGCCTGG | TTCAATTAGCCACTC | 284 |
| *AhCYP314A1* | GAAGCTCAGATGGTGTAC | CTTTCTGCCGTGGCATC | 503 |
| *AhCYP315A1* | AACATCGGACCTACCAC | ATTTCTCAGCTAGTTTCGG | 464 |
| *AhCYP6A8* | AATATTCAATCCTATATAAACG | TCATATTTTTTTATGACATC | 293 |
| *AhCYP6A9* | AAAGTGGTGGGTAAAGC | TGTCCATTTAAGAGGGAGCC | 280 |
| *AhCYP6A13* | ACCTCATCTTCGCGATTAG | TAATTTGAGCCATACACTC | 376 |
| *AhCYP6A14* | ACAGTGGATGGAAACAC | GGATCCAATTATGTCAGTGG | 365 |
| *AhCYP6A20* | ACATTGGAAAAGTTTTAGAC | TACGCCATTGGAGGTATGC | 508 |
| *AhCYP6A23* | ATTTATTTGCTCTCGGAGG | TGATTGTATTTACATCGCC | 534 |
| *AhCYP6AS5* | ATAACGAAAATCTTACAATAC | TACATCGGGATCAAATTCT | 290 |
| *AhCYP6BQ7* | ATACCTGTTGACTTGGAG | CAACTATTTCTTTTGTCG | 619 |
| *AhCYP6BQ8* | AGTACTTTACCGATCATGG | AATCTTTCTTAACAATTCC | 507 |
| *AhCYP6BQ9* | ACCATGAAGGGTTTGTTAATG | TTAACAAATGCATAAAATCC | 527 |
| *AhCYP6BQ10* | AACTCCTGTATTCACACC | AACATCGGTGATCTCAATTG | 295 |
| *AhCYP6BQ11* | AGCAGGTTTTGAAACGTC | GGTATATCAATTTTAGTTCC | 288 |
| *AhCYP6BQ15* | AGTGGAGGAATCTTAGAGC | AGTTTTAAACAGCAACGC | 512 |
| *AhCYP6BQ16* | AGCTAAGGGTGAACGATATTG | TGCACCAGCCATCTTGGA | 510 |
| *AhCYP6BQ34* | ATGTTATGTATTACAATATTTC | CAACCCACCATTGTTTGA | 455 |
| *AhCYP6BQ36* | ACCGTATTTACCGAAAAGAC | TGATCACGTTTGTATTTGG | 420 |
| *AhCYP6BR2* | AGCCGCATTTAATTGTAATGG | TAAGAGTTCATTGTATCC | 295 |
| *AhCYP6BR3* | ATGGCTAAGTTTACGACCG | TTGTAACGATTCATAGC | 522 |
| *AhCYP6BS1* | AGTCGCAAGGCCTTGTTGCC | TATTTCAATTTCGCCATC | 288 |
| *AhCYP6BW2* | AAGCCTGTTTTTACTCC | TCGGTAGTGTCAATGGG | 290 |
| *AhCYP6CR2* | ATTGACTTTTCCAGCATAAC | ATCTTCTTAATTATTTCAG | 299 |
| *AhCYP6DG1* | ACTACGGTGGTGTGTATAC | TTTGGTCCACTAGATCACC | 285 |
| *AhCYP6EF1* | ACTTCACCGACAGAGCC | AATGGATTTTCTTCATC | 311 |
| *AhCYP6FP7* | AGCTCTTTATGGTGCTGG | TCTCCAAATGGCAAATAAG | 417 |
| *AhCYP6M7* | ATGGTATTTAAAAGAAAAT | CAACTCTAGTAGCAGTTGT | 352 |
| *AhCYP6J1* | ATGCTTATTACTTCATCGTG | TTACATTTAAACTAGTACC | 526 |
| *AhCYP6K1* | ACTGAGAGATGCCTTCACG | GCCTTCATGCAGGTCAATGC | 305 |
| *AhCYP9AC1* | GAATTAATATCAGAATGTA | TGTGGGCTGCAAAACTGAG | 448 |
| *AhCYP9E2* | AAGGATCTAGCAGCTCG | ATTAAAGAAGATACCGACTC | 446 |
| *AhCYP9F2* | AGCTACTGGAGTGATCGA | CACACCAAATGCTGTGGTC | 504 |
| *AhCYP9Z1* | TGCGTCCCATATTAAGTCC | ATCTGATTCTTTAACAGT | 515 |
| *AhCYP9Z4* | CACTCTTCCAGACTTCATGC | CATATCAAAAGCCTAACC | 522 |
| *AhCYP9Z19* | AGAAGAATGTGCCTCAAG | CACCAAAGGCGGTGGTGG | 489 |
| *AhCYP9Z62* | AAGTCCCTCCTTCACAAGC | TTACAATATTCCAAATACT | 275 |
| *AhCYP9Z63* | ATAGAAGAACTTACATTCC | GGATTTTCCAAAGAATTC | 295 |
| *AhCYP345B1* | ACCACATCGATCATCTGCG | AAGTGTTCTTGCTGTACGC | 340 |
| *AhCYP345C1* | AAGAAGTCTTCTTGCGC | TCGTTTCAACTTGTCCACA | 518 |
| *AhCYP345F1* | CGAGATTTTAAGAATTTCGA | GCTACTACATCTGTCATGTA | 289 |
| *AhCYP347A5* | AATGGCGTTCCTGGCCCG | AAGCATTTGCCTTCCAGACC | 503 |
| *AhCYP4AA1* | ATGGACTACTACCTAGGCC | TAACGGTTTCGTTACCTTCT | 484 |
| *AhCYP4BD1* | TATTGGCTGAACAGAAGG | ACCCTACTGTTATCCAGGA | 289 |
| *AhCYP4BD4V1* | ACAAGACGTACCGAGGCC | ATATTCAGAGGTTTCATTGT | 514 |
| *AhCYP4BD4V2* | ATAGGAGTAATATGCCTAC | TAGGGGTAGATATTAAAGC | 515 |
| *AhCYP4BN1* | ATCCGTTCGTTATTCGTTTC | TTACTTTCAGGCACTGAT | 545 |
| *AhCYP4BN15* | AGATGGAAACAACATCGG | TGTGAAGGGTTGAATAG | 295 |
| *AhCYP4BN28* | ATGGCTGCCCTGGATATT | AACGCCAGTCGTCTCTTGC | 311 |
| *AhCYP4BQ1* | ATGGTGATAAATGGCGTAC | CTGCTTTCTCTTTGTAATAG | 351 |
| *AhCYP4BR7* | ATGTGCTCCCCGTTG | TAGATCAAGGTGCTCTACC | 456 |
| *AhCYP4C1* | GCAATGTTGTCCCACTCA | ATTAGCAAGTAACATCAAC | 482 |
| *AhCYP4C3* | ATGGAATACGTTGAAAGC | TGGAAATAAAGTACCGTCG | 546 |
| *AhCYP4C39* | ATCAGTTCTTGGGGTTAAC | TTAATAAGCTCCTCTTGG | 351 |
| *AhCYP4D1* | GGATTTGTTCCTATGTTGC | AGAGCTCTTAAATACTCC | 201 |
| *AhCYP4D2* | GGTGTAAAAATGAACATCC | AAATAGAAAGTTATTAAGC | 357 |
| *AhCYP4D8* | ACTGGAGGTCCAAAGAG | AACTTTTAAGTTGAAGCA | 521 |
| *AhCYP4D14* | ATCAAACCCGGAATACGTC | TTCTTCATACACCTTGTGC | 424 |
| *AhCYP4E2* | AATTGTACTTGGGTATGATG | AAGTCGTAAGTCAAAATG | 189 |
| *AhCYP4G123* | ACACAGATGCTGATTGATCC | TCGATGTGATCATGACTGC | 350 |
| *AhCYP4G15* | ATGTATGCCAAATCTTTCG | TTCCACTGTAGCCTCAGAC | 357 |
| *AhCYP4Q9* | CCACAATGAGACAATGGAAC | CAATGGTAGTATATTTGTAC | 344 |
| *AhCYP4Q15* | AAGCGTTTGCCCATCGTTGG | TTCGTTAGTTTTCTCAACGC | 471 |
| *AhCYP4Q32* | ATGTATTGCCATTAATGTCAC | TCTGAATCGCCTGTTACGC | 523 |
| *AhCYP4Q33* | ATAAGTTTTATAGGTTCATG | AGATCCAATAGTGCAAGAC | 526 |
| *AhCYP4Q34* | ATACTGGAAGCACATTATGG | GCTTAGTTACATCTACGT | 434 |
| *AhCYP411A1* | AAGCAAACTACCATGGAC | TTGGTTTTAGTCCTGATGC | 285 |
| *AhCYP412A2* | AACAGGAAACATTCCATTGT | CGATATATAACTTAGGTCCA | 299 |
| *AhCYP4H10* | CGGATTATTTGTTAAATGG | AATTTCGTAGAGCAGCAAA | 324 |
| *AhCYP4V2* | AACACCTACTCTACATTTAC | TGTGAAATTGTAGATACATG | 295 |
| *AhCYP4P1* | ACGATACTTTTAATTTTAC | GGAAACTGATGCTTTTTAG | 448 |
| *AhCYP349G1* | CTGGTCTATTCAGTATACTT | TCAGCATTTTTATTCTGTGC | 543 |

Supplementary Table 4. Primers used in real-time PCR for determination expression levels of *Agasicles hygrophila* CYP genes.

| Gene name | Forward primers (5'-3') | Reverse primers (5'-3') | Amplicon size (bp) |
| --- | --- | --- | --- |
| *Actin* | ATGTATGTAGCCATCCAA | CATCTCCAGAGTCCAATA | 79 |
| *CoxI* | CCACTTCCATTATGTCCTATC | GCGGTATTCCTATCAATCC | 188 |
| *AhCYP15A1* | GATGATAAACGACTGAGAAA | GCATTAGGAGCAATATACC | 116 |
| *AhCYP18A1* | AGACAGAAGATTGCTAAG | ACAAGTAAGTATCAAGAAGA | 118 |
| *AhCYP303A1* | GGGAGTCACTGGCAAGAG | GGATGATCTGGTACTATGCTG | 85 |
| *AhCYP305A1* | TTCAGGAATAACATCAAC | CACTTCATCTCTTATCATT | 121 |
| *AhCYP306A1* | CGTAAGCCATCCATAGAAG | AGGTCTGATTCGCATAGT | 78 |
| *AhCYP307A1* | TTATGAACAACTACGAACTT | ATCCTCCAATTACACCAA | 189 |
| *AhCYP307A2* | TTGGCAGCATATTATATTCG | CTCCTGTAACCTCATCTATT | 146 |
| *AhCYP12A2* | CAGCAGGAGCAAATTCTA | CAATAACGGTCAGCATCT | 139 |
| *AhCYP49A1* | ACGAAGGTGTAATAGGAGTA | TTAGCAGTAAGAGGAGGTAA | 88 |
| *AhCYP301A1* | GTCCACAATATACCAGTT | GCTCTAAGATAAGGCATT | 132 |
| *AhCYP302A1* | TGAGGAACAGCAATACAGAT | CCACAGTTGAGGTCCATT | 200 |
| *AhCYP314A1* | TCTTGTTCGTTGGTTCTA | TAGGTATCTCTGGTAGCA | 119 |
| *AhCYP315A1* | TAGATGATACTGTTGTCCTT | ATCCTTACCGATGTTACC | 197 |
| *AhCYP6A8* | GGATTATACTACCAGTCTTG | GAGCACCTATACATATTCG | 150 |
| *AhCYP6A9* | CCAGCTTAGGTCACAGATT | TGTTTCAGTTTCATCAAGGT | 193 |
| *AhCYP6A13* | ATGCTTGAATGAATGTCT | TGAGGTATAGGTGTAGTC | 82 |
| *AhCYP6A14* | CCAATACACCGTTCAGAGA | GTAGAGTCACGCCAAGAA | 120 |
| *AhCYP6A20* | TGTTGGCTGATACTGGAA | CATAGTTGTTGCTGAGGTT | 113 |
| *AhCYP6A23* | AAGCGTATAGTCCGTTCA | ATCCAAGTCATCCTAATAGA | 124 |
| *AhCYP6AS5* | TTCAGGATAGAAGCACAA | CCGATATAACAGCCATCT | 111 |
| *AhCYP6BQ7* | TTCATACCTGTTGACTTGGA | ACTTCTTGCGGCTATCTT | 157 |
| *AhCYP6BQ8* | TCTGTTACCAATGATGAA | ATAAGTGTGGAAGATGTT | 134 |
| *AhCYP6BQ9* | TCACTTACGATGCTATAATG | GGTATCTGTTCCTGGTAT | 131 |
| *AhCYP6BQ10* | TCCTAATCAATATGCTTCCA | CTCTTCATCCAACAATCT | 194 |
| *AhCYP6BQ11* | AGAGCGGTTCAGTCCAGAA | GCAAGTACGAGGTCCTTC | 79 |
| *AhCYP6BQ15* | GGTAATAGCAGTTGTGTT | TGGTAGCAGAAGTATCAA | 94 |
| *AhCYP6BQ16* | TCCAATGATATGCCGAAT | TGCTACAGTCTTCTCAAC | 127 |
| *AhCYP6BQ34* | CATTACAGGCAGAAGAGAC | TCCAAATCAAAGACTACA | 126 |
| *AhCYP6BQ36* | TTACCATTCGGAGAAGGA | TCAGTTATATCAAGCCATACA | 191 |
| *AhCYP6BR2* | TTTGTTGCTGGTTATGAA | TGTGTAAGTAGAGGTGTT | 176 |
| *AhCYP6BR3* | CTCATCTACTTACTTACCATT | GGATTCTTCGTCTTCAATA | 183 |
| *AhCYP6BS1* | CAGTTATCTATGAGACAATG | TCTTCAGAGAATCTATCG | 199 |
| *AhCYP6BW2* | ATTGCTGCTATCTTCATT | GTAATTGGAACACAGAGA | 144 |
| *AhCYP6CR2* | TGAAGAAGATGTATGGTATTA | TGCTGCTATTATGTCTATGGT | 134 |
| *AhCYP6DG1* | CAATGCGTAACAACAACAAT | TCTTCCAAGTAGTAATCTC | 145 |
| *AhCYP6EF1* | CATCCAGCATACTTCACAGA | CCAACACTTCACCAGACATA | 99 |
| *AhCYP6FP7* | ACCTATGAAGCATTACAG | TATCAATACCAGCATATCTT | 165 |
| *AhCYP6M7* | GCCACATACAACACAGAT | GCATAAGTGAATAACCCATC | 147 |
| *AhCYP6J1* | TTGCTGCTTCTACTGATT | TGTGCTGTCGTATAGTTC | 183 |
| *AhCYP6K1* | ATATTCCACTATACGCTATTCA | GTTCACCTATACATTGTCTTG | 153 |
| *AhCYP9AC1* | ATCATTGACCAGACGATTAAG | ATCCTCCACTTGCTCTTC | 114 |
| *AhCYP9E2* | ATAGCACTCAACCAAGAC | TCCGAGACTACCATATCC | 128 |
| *AhCYP9F2* | GTTCACTCAGCCAATACT | GACCAATAATAATCCGAAG | 123 |
| *AhCYP9Z1* | CCAAGGAAGAACACAATG | TCACTGCTAACTCGTATG | 186 |
| *AhCYP9Z4* | CCGTTATTACTGATGATGAC | TTCTCTTGTATATCCTGGTTA | 127 |
| *AhCYP9Z19* | AGAACAGTCACGGTTAAGA | TTAGCAGAGCCTCATACG | 80 |
| *AhCYP9Z62* | TGGTATCTGATTGTGCTAAC | CAAGTTCTGTGCTGTCAT | 188 |
| *AhCYP9Z63* | TGTGATTCAACTATTGGTAG | TTGTGCTGTTATGTCTTC | 151 |
| *AhCYP345B1* | TCTTCGTTGCTCTATACT | TAGGTAAGGTGTCTTCATT | 176 |
| *AhCYP345C1* | GTAGTAGCCACATCATTA | TCACCTATACATACTCTTG | 187 |
| *AhCYP345F1* | GACGAAGATGATATTACG | CTAACGGAATAAGAACAG | 181 |
| *AhCYP347A5* | GGACATAGCCAATAGATTC | TGCGATACTTAATAGAGGAT | 164 |
| *AhCYP4AA1* | TCAGCAATACTATTAGCA | AATCGTTGGTAATAAGGA | 198 |
| *AhCYP4BD1* | CCTATGTCTACTGTCCTT | TAGTTGAAGTCCTTGTATG | 143 |
| *AhCYP4BD4V1* | AAGAAGAGGTAGATACTT | TCTTGTAGTTCTGTTATC | 175 |
| *AhCYP4BD4V2* | AAGTGTCAAGACTATGTG | GAATCCGTATGGTTATGTA | 132 |
| *AhCYP4BN1* | TTAGAGACCTACATTGAGATT | GCTTCGCATATTATATCCAA | 134 |
| *AhCYP4BN15* | TCTTCTTGGATATGCTGTTG | CGACCTCTTCTCTGATGT | 75 |
| *AhCYP4BN28* | ATCTAATGTGTCGTGTAAT | GTCAATATCCTCCTCATC | 191 |
| *AhCYP4BQ1* | CGCTGTATGGTAGGAATG | AACCGAGAAGGATTGAAC | 145 |
| *AhCYP4BR7* | ATCACAACAAGGAGTATTATC | GTATTCCATCAGCATTCG | 185 |
| *AhCYP4C1* | CTTGAACAGGCATCCATT | AATAGAGCGAATCGTTGAC | 78 |
| *AhCYP4C3* | ATTGCCATTAGACACTATGAA | TCACGAATTATCCGAGTTG | 162 |
| *AhCYP4C39* | CGACAATCCGTTCAAGTA | AATAAGGCGTCAAGCAATA | 143 |
| *AhCYP4D1* | CCTGTTAGTGTTGGTCTT | AATACTCCATCGCCTATC | 125 |
| *AhCYP4D2* | CGCTGTTATTGGTGACTA | GGCTCTATTGGCTTGATT | 145 |
| *AhCYP4D8* | TTGTGTTGGTCAGAAGTATG | CAGCGTTATTGATGGTAGG | 124 |
| *AhCYP4D14* | CATCGTGAAGAACAGAAG | CAGCAACATATCCAACAA | 106 |
| *AhCYP4E2* | CTCCACCAAGATCATAGA | TGCTAGTTAGAAGTCCTT | 77 |
| *AhCYP4G123* | GATGAGGATGTGCGATAT | CTGTGAATGGTATTGAGTAAT | 120 |
| *AhCYP4G15* | TTATCCTACGGTCAATCC | ATCAACAAGTCCAAGAATG | 95 |
| *AhCYP4Q9* | TAGCACAGGAAGTCAATG | GAGACAGCAATGGTAGTAT | 170 |
| *AhCYP4Q15* | CAGGTTAATGAGGATGTTA | TAGGCATAGTAAGGTCTC | 176 |
| *AhCYP4Q32* | GGATATGCTATACGAAGAA | GCTTGGATATAACCTCAG | 127 |
| *AhCYP4Q33* | GCAAGTGAACCAGAATATC | TCTCCAATAACTCTTCCAAT | 176 |
| *AhCYP4Q34* | GAACAGGCTTACTTACAA | TTATCGGCTTAGTTACATC | 183 |
| *AhCYP411A1* | CCTGATGACTCTTATGCTA | CTTGTAAGTATCGTCTAATGTT | 166 |
| *AhCYP412A2* | TATATCGCTGTGTCTAAC | ATCTTCTGATTGAATGCT | 179 |
| *AhCYP4H10* | GTTGCTGCTGAAGATACTG | TCCAAGAAGTGACAAG | 84 |
| *AhCYP4V2* | AAGGAAGCGAATATACTGG | ATGTAGAGCGTGACTGT | 171 |
| *AhCYP4P1* | GCAGTATCTTGACCAATGTG | GTTATCCAATGTGGCAT | 97 |
| *AhCYP349G1* | GAATTATGGAGGTTGTCTTG | TGTATCTGTATCTTGCTG | 182 |

Supplementary Table 5. Cytochrome P450 monooxygenase genes identified in the *Agasicles hygrophila* adult transcriptome.

| **Gene name** | **Accession**  **number** | **ORF(bp)** | **Completeness** | **Best Blastx hit** | | | | | |
| --- | --- | --- | --- | --- | --- | --- | --- | --- | --- |
|  |  |  |  | **Gene annotation** | **Species** | **Protein ID** | **Score** | *E*-value | Identity |
| *CYP15A1*-partial | MH158318 | 507 | NO(5’/3’-lost | cytochrome P450 15A1 | *Tribolium castaneum* | EFA01264.1 | 216 | 5e-65 | 72% |
| *CYP18A1*-partial | MH158319 | 1203 | NO(3’-lost) | cytochrome P450 18A1 | *Anopophora glabripenni*s | XP_018564416.1 | 654 | 0.0 | 84% |
| *CYP303A1*-partial | MH158320 | 309 | NO(5’/3’-lost) | probable cytochrome P450 303a1 | *Anopophora glabripennis* | XP_018572226.1 | 155 | 9e-43 | 66% |
| *CYP305A1*-partial | MH158321 | 1341 | NO(3’-lost) | cytochrome P450 305a1 | *Leptinotarsa decemlineata* | AGT57832.1 | 416 | 2e-134 | 56% |
| *CYP306A1*-partial | MH158322 | 1299 | NO(3’-lost) | cytochrome P450 305a1 | *Leptinotarsa decemlineata* | AGT57833.1 | 512 | 3e-172 | 54% |
| *CYP307A1*-partial | MH158323 | 1056 | No(5’-lost) | cytochrome P450 307a1 | *Tribolium castaneum* | XP_974280.1 | 498 | 5e-171 | 63% |
| *CYP307A2*-partial | MH158324 | 1455 | NO(3’-lost) | cytochrome P450 307a2 | *Nilaparvata lugens* | AIW79977.1 | 557 | 0.0 | 55% |
| *CYP12A2*-partial | MH158325 | 825 | No(5’-lost) | cytochrome P450 CYP12A2 | *Tribolium castaneum* | XP_008190935.1 | 248 | 6e-68 | 49% |
| *CYP49A1*-partial | MH158326 | 708 | No(3’-lost) | cytochrome P450 49A1 | *Tribolium castaneum* | EFA07521.1 | 313 | 4e-100 | 71% |
| *CYP301A1*-partial | MH158327 | 672 | No(5’-lost) | cytochrome P450 301A1 | *Tribolium castaneum* | XP_974014.1 | 231 | 4e-68 | 43% |
| *CYP302A1*-partial | MH158328 | 783 | No(5’-lost) | cytochrome P450 302a1, mitochondrial-like | *Leptinotarsa decemlineata* | [XP_023028420.1](https://www.ncbi.nlm.nih.gov/protein/XP_023028420?report=genbank&log$=protalign&blast_rank=2&RID=2M0PN70D014) | 314 | 2e-98 | 64% |
| *CYP314A1*-partial | MH158329 | 1476 | No(3’-lost) | probable cytochrome 314A1 | *Leptinotarsa decemlineata* | AGT57843.1 | 649 | 0.0 | 62% |
| *CYP315A1* | MH158330 | 1410 | Yes | cytochrome 315A1, mitochondrial | *Leptinotarsa decemlineata* | AGT57844.1 | 529 | 9e-151 | 47% |
| *CYP6A8*-partial | MH158331 | 1504 | No(3’-lost) | cytochrome P450 6a8-like | *Leptinotarsa decemlineata* | XP_023023338.1 | 472 | 1e-159 | 48% |
| *CYP6A9*-partial | MH158332 | 397 | NO(5’/3’-lost) | cytochrome P450 6a9-like | *Anoplophora glabripennis* | XP_018571884.1 | 62.8 | 9e-09 | 35% |
| *CYP6A13* | MH158333 | 1509 | Yes | probable cytochrome P450 6a13 | *Leptinotarsa decemlineata* | XP_023024821.1 | 507 | 3e-153 | 44% |
| *CYP6A14* | MH158334 | 1533 | Yes | probable cytochrome P450 6a14 | *Anoplophora glabripennis* | XP_023311070.1 | 642 | 0.0 | 58% |
| *CYP6A20*-partial | MH158335 | 777 | No(5’-lost) | probable cytochrome P450 6a20 | *Tribolium castaneum* | XP_015834510.1 | 294 | 1e-93 | 56% |
| *CYP6A23*-partial | MH158336 | 1239 | No(5’-lost) | probable cytochrome P450 6a23 | *Leptinotarsa decemlineata* | XP_023029782.1 | 505 | 2e-172 | 53% |
| *CYP6AS5*-partial | MH158337 | 381 | No(5’/3’-lost) | probable cytochrome P450 6AS5 | *Apis mellifera* | NP_001035324.1 | 126 | 2e-31 | 47% |
| *CYP6BQ7*-partial | MH158338 | 1020 | No(5’-lost) | cytochrome P450 6BQ7 | *Tribolium castaneum* | EFA02821.2 | 273 | 4e-86 | 37% |
| *CYP6BQ8* | MH158339 | 1536 | Yes | cytochrome P450 6BQ8 | *Tribolium castaneum* | NP_001280532.1 | 420 | 3e-138 | 44% |
| *CYP6BQ9* | MH158340 | 1518 | Yes | cytochrome P450 6BQ9 | *Tribolium castaneum* | NP_001177722.1 | 530 | 0.0 | 50% |
| *CYP6BQ10* | MH158341 | 1527 | Yes | cytochrome P450 6BQ10 | *Tribolium castaneum* | NP_001164249.1 | 473 | 2e-156 | 46% |
| *CYP6BQ11*-partial | MH158342 | 750 | No(5’-lost) | cytochrome P450 6BQ11 | *Tribolium castaneum* | NP_001280531.1 | 305 | 3e-97 | 56% |
| *CYP6BQ15* | MH158343 | 1551 | Yes | cytochrome P450 6BQ15, partial | *Leptinotarsa decemlineata* | AGT57849.1 | 561 | 0.0 | 50% |
| *CYP6BQ16*-partial | MH158344 | 798 | No(5’/3’-lost) | cytochrome P450 6BQ16, partial | *Leptinotarsa decemlineata* | AGT57850.1 | 236 | 2e-73 | 43% |
| *CYP6BQ34*-partial | MH158345 | 861 | No(3’-lost) | cytochrome P450 monooxygenase 6BQ34 | *Tenebrio molitor* | AKZ17693.1 | 228 | 5e-67 | 40% |
| *CYP6BQ36*-partial | MH158346 | 1122 | No(5’-lost) | cytochrome P450 monooxygenase 6BQ36 | *Tenebrio molitor* | AKZ17695.1 | 439 | 4e-148 | 56% |
| *CYP6BR2*-partial | MH158347 | 1058 | No(3’-lost) | cytochrome P450 CYP6BR2 | *Tribolium castaneum* | EFA12628.2 | 267 | 6e-82 | 38% |
| *CYP6BR3*-partial | MH158348 | 963 | No(5’-lost) | cytochrome P450 CYP6BR3 | *Tribolium castaneum* | EFA12627.2 | 281 | 1e-106 | 45% |
| *CYP6BS1* | MH158349 | 1512 | Yes | cytochrome P450 CYP6BS1 | *Tribolium castaneum* | EEZ99243.2 | 587 | 0.0 | 72% |
| *CYP6BW2* | MH158350 | 1530 | Yes | cytochrome P450 CYP6BW2 | *Dendroctonus ponderosae* | AFI45025.1 | 445 | 1e-147 | 46% |
| *CYP6CR2* | MH158351 | 1551 | Yes | cytochrome P450 CYP6CR2 | *Dendroctonus ponderosae* | AFI45030.1 | 503 | 3e-166 | 48% |
| *CYP6DG1*-partial | MH158352 | 1497 | No(5’-lost) | cytochrome P450 CYP6DG1 | *Dendroctonus ponderosae* | AFI45036.1 | 399 | 1e-129 | 43% |
| *CYP6EF1*-partial | MH158353 | 1470 | No(5’-lost) | cytochrome P450 CYP6EF1,partial | *Leptinotarsa decemlineata* | AGT57858.1 | 339 | 7e-108 | 37% |
| *CYP6FP7*-partial | MH158354 | 597 | No(5’/3’-lost) | cytochrome P450 CYP6FP7 | *Propylea japonica* | AHU88028.1 | 166 | 1e-44 | 38% |
| *CYP6M7*-partial | MH158355 | 426 | No(5’/3’-lost) | cytochrome P450 CYP6M7 | *Anopheles funestus* | AIE17429.1 | 85.5 | 1e-16 | 34% |
| *CYP6J1* | MH158356 | 1500 | Yes | cytochrome P450 6J1-like | *Zootermopsis nevadensis* | XP_021930152.1 | 333 | 1e-104 | 36% |
| *CYP6K1* | MH158357 | 1512 | Yes | cytochrome P450 6K1-like | *Anoplophora glabripennis* | XP_018561043.1 | 378 | 9e-123 | 38% |
| *CYP9AC1*-partial | MH158358 | 633 | No(5’/3’-lost) | cytochrome P450 9AC1 | *Tribolium castaneum* | EFA01242.2 | 441 | 1e-113 | 54% |
| *CYP9E2*-partial | MH158359 | 1179 | No(5’-lost) | cytochrome P450 9E2-like isoform X4 | *Aethina tumida* | XP_019875693.1 | 452 | 2e-123 | 50% |
| *CYP9F2*-partial | MH158360 | 840 | No(3’-lost) | cytochrome P450 isoform 9F2 | *Tribolium castaneum* | NP_001127706.1 | 265 | 6e-77 | 52% |
| *CYP9Z1*-partial | MH158361 | 1446 | No(5’/3’-lost) | cytochrome P450 9Z1 | *Tribolium castaneum* | EFA09239.2 | 531 | 0.0 | 55% |
| *CYP9Z4* | MH158362 | 1566 | Yes | cytochrome P450 9Z4 | *Tribolium castaneum* | NP_001164248.1 | 513 | 7e-165 | 47% |
| *CYP9Z19* | MH158363 | 1566 | Yes | cytochrome P450 CYP9Z19 | *Dendroctonus ponderosae* | AFI45046.1 | 479 | 7e-161 | 48% |
| *CYP9Z62*-partial | MH158364 | 1023 | No(5’/3’-lost) | cytochrome P450 monooxygenase CYP9Z62 | *Tenebrio molitor* | [AKZ17700.1](https://www.ncbi.nlm.nih.gov/protein/AKZ17700?report=genbank&log$=protalign&blast_rank=13&RID=6FZEA3CK01R) | 305 | 1e-94 | 47% |
| *CYP9Z63* | MH158365 | 1578 | Yes | cytochrome P450 monooxygenase CYP9Z63 | *Tenebrio molitor* | AKZ17701.1 | 528 | 5e-100 | 49% |
| *CYP345B1*-partial | MH158366 | 1497 | No(5’/3’-lost) | cytochrome P450 monooxygenase CYP345B1 | *Tenebrio molitor* | AKZ17703.1 | 353 | 3e-112 | 38% |
| *CYP345C1* | MH158367 | 1521 | Yes | cytochrome P450 345C1 | *Tribolium castaneum* | KYB27493.1 | 330 | 2e-103 | 37% |
| *CYP345F1*-partial | MH158368 | 1494 | No(5’/3’-lost) | cytochrome P450 345F1 | *Dendroctonus ponderosae* | AFI45009.1 | 528 | 2e-180 | 53% |
| *CYP347A5*-partial | MH158369 | 1497 | No(5’-lost) | cytochrome P450 monooxygenase CYP347A5 | *Tenebrio molitor* | AKZ17705.1 | 474 | 1e-160 | 46% |
| *CYP4AA1*-partial | MH158370 | 1452 | No(5’/3’-lost) | Probable cytochrome P450 4AA1 isoform X1 | *Anoplophora*  *glabripennis* | XP_018572295.1 | 491 | 1e-168 | 56% |
| *CYP4BD1*-partial | MH158371 | 513 | No(5’-lost) | CYP4BD1 | *Ips paraconfusus* | ABF06546.1 | 200 | 4e-57 | 56% |
| *CYP4BD4V1*-partial | MH158372 | 1386 | No(5’/3’-lost) | cytochrome P450 CYP4BD4V1 | *Dendroctonus ponderosae* | AFI45016.1 | 464 | 6e-156 | 49% |
| *CYP4BD4V2*-partial | MH158373 | 987 | No(3’-lost) | cytochrome P450 CYP4BD4V2 | *Dendroctonus ponderosae* | AFI45017.1 | 267 | 5e-109 | 46% |
| *CYP4BN1*-partial | MH158374 | 1458 | No(5’-lost) | cytochrome P450 CYP4BN1 | *Tribolium castaneum* | NP_001123993.1 | 441 | 6e-147 | 46% |
| *CYP4BN15*-partial | MH158375 | 561 | No(5’/3’-lost) | cytochrome P450 CYP4BN15 | *Leptinotarsa decemlineata* | AGT57866.1 | 199 | 1e-60 | 53% |
| *CYP4BN28*-partial | MH158376 | 1128 | No(5’-lost) | cytochrome P450 monooxygenase CYP4BN28 | *Tenebrio molitor* | AKZ17708.1 | 372 | 1e-122 | 48% |
| *CYP4BQ1*-partial | MH158377 | 1446 | No(5’-lost) | cytochrome P450 CYP4BQ1 | *Dendroctonus ponderosae* | AFI45019.1 | 409 | 1e-133 | 45% |
| *CYP4BR7* | MH158378 | 1458 | Yes | cytochrome P450 monooxygenase CYP4BR7 | *Tenebrio molitor* | AKZ17709.1 | 286 | 1e-86 | 33% |
| *CYP4C1* | MH158379 | 1521 | Yes | cytochrome P450 4C1-like | *Anoplophora glabripennis* | [XP_018562118.1](https://www.ncbi.nlm.nih.gov/protein/1080042156?report=genbank&log$=protalign&blast_rank=1&RID=2J2TGBR6015) | 911 | 0.0 | 60% |
| *CYP4C3*-partial | MH158380 | 906 | No(5’-lost) | cytochrome P450 4C3-like isoform X2 | *Dendroctonus ponderosae* | XP_019761610.1 | 248 | 7e-75 | 42% |
| *CYP4C39* | MH158381 | 1476 | Yes | cytochrome P450 enzyme, enzyme, CYP4C39 | *Scylla serrata* | pirllJC8026 | 311 | 2e-96 | 36% |
| *CYP4D1*-partial | MH158382 | 201 | No(5’/3’-lost) | cytochrome P450 4D1 | *Aedes aegypti* | XP_001652926.1 | 57.8 | 4e-08 | 38% |
| *CYP4D2* | MH158383 | 1485 | Yes | cytochrome P450 4D2-like | *Leptinotarsa decemlineata* | XP_023015850.1 | 392 | 3e-128 | 43% |
| *CYP4D8*-partial | MH158384 | 696 | No(5’/3’-lost) | cytochrome P450 4d8 | *Culex quinquefasciatus* | XP_001870443.1 | 180 | 1e-50 | 42% |
| *CYP4D14*-partial | MH158385 | 1500 | No(3’-lost) | Probable cytochrome P450 4d14 isoform X3 | *Musca domestica* | XP_005184046.1 | 382 | 8e-124 | 41% |
| *CYP4E2*-partial | MH158386 | 192 | No(5’/3’-lost) | cytochrome P450 4E2-like | *Dendroctonus ponderosae* | XP_019761611.1 | 55.1 | 9e-07 | 40% |
| *CYP4G123* | MH158387 | 1683 | Yes | cytochrome P450 monooxygenase CYP4G123 | *Tenebrio molitor* | AKZ17710.1 | 842 | 0.0 | 72% |
| *CYP4G15* | MH158388 | 1686 | Yes | cytochrome P450 4G15 | *Leptinotarsa decemlineata* | XP_023013049.1 | 972 | 0.0 | 87% |
| *CYP4Q9*-partial | MH158389 | 909 | No(5’/3’-lost) | cytochrome P450 monooxygenase CYP4Q9 | *Tribolium castaneum* | NP_001107850.1 | 209 | 2e-60 | 40% |
| *CYP4Q15* | MH158390 | 1677 | Yes | cytochrome P450 4g15-like | *Anoplophora glabripennis* | XP_018572314.1 | 716 | 0.0 | 64% |
| *CYP4Q32* | MH158391 | 1521 | Yes | cytochrome P450 monooxygenase CYP4Q32 | *Tenebrio molitor* | AKZ17711.1 | 450 | 7e-150 | 45% |
| *CYP4Q33* | MH158392 | 1518 | Yes | cytochrome P450 monooxygenase CYP4Q33 | *Tenebrio molitor* | AKZ17712.1 | 469 | 4e-157 | 47% |
| *CYP4Q34* | MH158393 | 1482 | Yes | cytochrome P450 monooxygenase CYP4Q34 | *Tenebrio molitor* | AKZ17713.1 | 489 | 3e-164 | 48% |
| *CYP411A1* | MH158394 | 1479 | Yes | cytochrome P450 CYP411A1 | *Dendroctonus ponderosae* | AFI45014.1 | 318 | 3e-99 | 35% |
| *CYP412A2* | MH158395 | 1554 | Yes | cytochrome P450 412A2 | *Leptinotarsa decemlineata* | AGT57837.1 | 414 | 4e-136 | 43% |
| *CYP4H10* | MH158396 | 1467 | Yes | cytochrome P450 monooxigenase CYP4H10 | *Tribolium castaneum* | NP_001107836.1 | 291 | 9e-89 | 35% |
| *CYP4V2*-partial | MH158397 | 372 | No(5’/3’-lost) | cytochrome P450 4V2 | *Parasteatoda tepidariorum* | XP_021001216.1 | 80.5 | 4e-15 | 35% |
| *CYP4P1*-partial | MH158398 | 453 | No(5’/3’-lost) | cytochrome P450 4P1-like | *Bactrocera dorsalis* | XP_011207610.1 | 135 | 1e-34 | 45% |
| *CYP349G1* | MH158399 | 1560 | Yes | cytochrome P450 monooxigenase CYP349G1 | *Tenebrio molitor* | AKZ17714.1 | 448 | 3e-149 | 44% |
